# A novel and robust heterogeneous Cu catalyst using modified lignosulfonate as support for the synthesis of nitrogen-containing heterocycles

**DOI:** 10.3762/bjoc.16.238

**Published:** 2020-11-26

**Authors:** Bingbing Lai, Meng Ye, Ping Liu, Minghao Li, Rongxian Bai, Yanlong Gu

**Affiliations:** 1 Key Laboratory of Material Chemistry for Energy Conversion and Storage, Ministry of Education. Hubei Key Laboratory of Material Chemistry and Service Failure, School of Chemistry and Chemical Engineering, Huazhong University of Science and Technology, 1037 Luoyu Road, Hongshan District, Wuhan 430074, P. R. China; 2 School of Chemistry and Chemical Engineering, the Key Laboratory for Green Processing of Chemical Engineering of Xinjiang Bingtuan, Shihezi University, Shihezi City, 832004, China; 3 State Key Laboratory for Oxo Synthesis and Selective Oxidation, Lanzhou Institute of Chemical Physics, Chinese Academy of Sciences, Lanzhou 730000, P. R. China

**Keywords:** biomass, heterogeneous catalyst, immobilized copper catalyst, lignosulfonate, nitrogen-containing heterocycles, solid acid

## Abstract

A waste biomass, sodium lignosulfonate, was treated with sodium 2-formylbenzenesulfonate, and the phenylaldehyde condensation product was then used as a robust supporting material to immobilize a copper species. The so-obtained catalyst was characterized by many physicochemical methods including FTIR, EA, FSEM, FTEM, XPS, and TG. This catalyst exhibited excellent catalytic activity in the synthesis of nitrogen-containing heterocycles such as tricyclic indoles bearing 3,4-fused seven-membered rings, 2‑arylpyridines, aminonaphthalenes and 3-phenylisoquinolines. In addition, this catalyst showed to be recyclable and could be reused several times without significant loss in activity during the course of the reaction process.

## Introduction

Heterogeneous metal catalysts have been continuously receiving considerable attention in the field of organic synthesis owing to the advantages of easy separation and recycling [1–4]. However, most of them often encounter the issues of poor stability and metal leaching [5], especially when the substrates and/or products have a powerful coordinating ability with the immobilized metal [6–7]. For instance, nitrogen-containing substrates or target products sometimes may lead to the fast deactivation of catalysts, which consequently impair the recyclability of the catalysts [8–11]. Therefore, special efforts should be paid to enhancing the robustness of heterogeneous metal catalysts.

Sodium lignosulphonate (LS) is a waste from the paper-making industry, containing aryl- and sodium sulfonate groups [12]. As a category of polyanionic material, LS can easily load metal ions via an ion exchange process [13]. Given the desirable property, an array of heterogeneous metal catalysts using LS as support were successfully designed and utilized to catalyze some typical organic reactions in our previous work. In 2014 [14], LS were used by our group, for the first time, as a solid support of cationic catalysts. The obtained catalysts were then successfully applied to many organic transformations, in which the catalysts showed not only high activity but also good recyclability. However, the stability and durability of LS was very limited especially in polar solvents such as H_2_O, EtOH and at harsh conditions. In a further study of our previous work [15], a LS/dicationic ionic liquid composite was prepared via an ion exchange process, and then used as catalyst support for preparing a heterogeneous Cu-based catalyst, the thereby obtained catalyst displayed remarkable performance in the Glaser hetero-coupling reaction. Combining two successful attempts, a further study concerning improvement of the robustness of catalysts and active sites when using LS as support is urgently needed.

In this work, we present a novel heterogeneous Cu catalyst using modified LS as support by a consecutive process involving the phenol–aldehyde condensation of LS with 2-formylbenzenesulfonic acid sodium (FAS), ion exchange and acidification. Special interest is given in the application of the prepared catalyst for synthesizing nitrogen-containing heterocycles. The results showed that the grafting of FAS on LS provided the support with more ion exchange sites, significantly increasing the loading capability of the Cu species. The acidification process could transform the –SO_3_Na group left in the catalyst after ion exchange into –SO_3_H, enabling the catalyst to catalyze the model reactions without the addition of protonic acid. The catalyst demonstrated impressive catalytic performance in the synthesis of nitrogen-containing heterocycles, and there was no deactivation even after 6 times of recycling, exhibiting enhanced stability compared to that without grafting of FAS. It is expected that this research would shed light on the design of heterogeneous metal catalysts with high activity and stability.

## Results and Discussion

**Figure 1:**
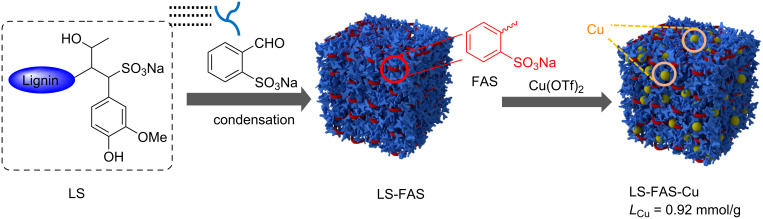
Schematic illustration for the preparation of the catalyst in this work.

The whole preparation process of the catalyst is depicted in Figure 1. Firstly, the support was prepared through phenol–formaldehyde condensation reaction of LS and FAS. The FAS was chosen to embellish LS in consideration of the following reason: FAS skeleton consists of both aldehyde and sulfonic groups, so the grafting of FAS and LS can be easily realized via phenol–formaldehyde condensation reaction, and therefore equips the generated polymeric support with more sulfonic groups. The heterogeneous Cu catalyst (LS-FAS-Cu) was finally obtained through refluxing of LS-FAS with Cu(OTf)_2_ in ethanol, and the loading capacity of Cu was confirmed to be 0.92 mmol/g by means of ICP. For comparison, two controlled heterogeneous catalysts, namely Resin-Cu and LS-FM-Cu were prepared using commercially available Amberlyst-15 and the material [13] was synthesized by condensation of formaldehyde and LS as supports, respectively (see Supporting Information File 1, Figure S1 and Figure S2), and the loading capacity of Cu was 0.45 mmol/g and 0.56 mmol/g, respectively.

### Characterization of the prepared materials

**Figure 2:**
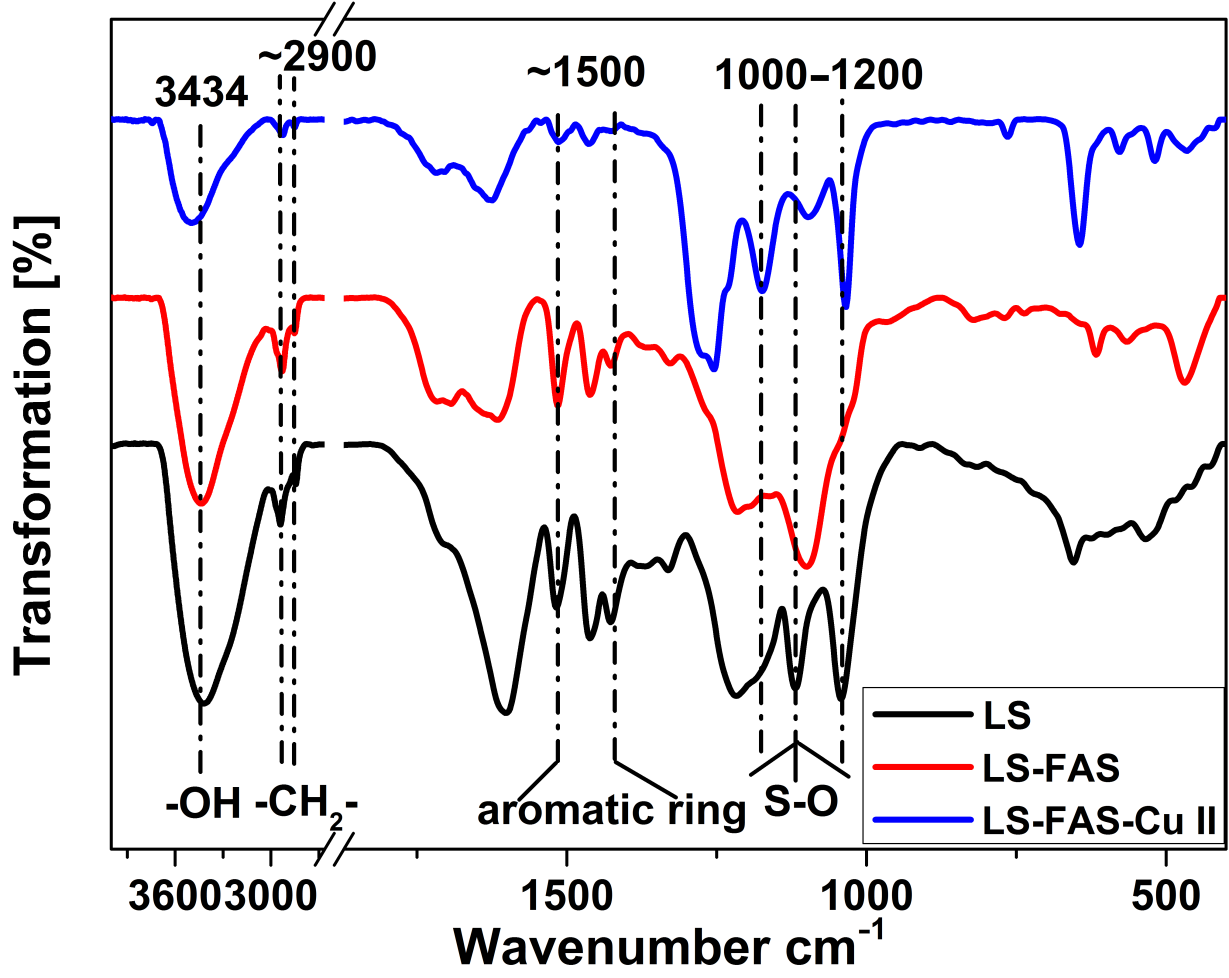
FTIR spectra of LS, LS-FAS, and LS-FAS-Cu.

The primary purpose of using FAS to modify LS was to increase the amount of –SO_3_Na groups in the supporting material. For the verification of this assumption, elemental analysis was firstly conducted and the results are listed in Table S1 (see Supporting Information File 1). From the results, it was found that with introduction of the FAS moiety, LS-FAS showed a higher content of C, H and S elements compared to pristine LS, indicating the successful grafting of FAS on LS. According to the increment of the S element, the increment of –SO_3_Na groups was 1.26 mmol/g. The chemical compositions on the surfaces of LS, LS-FAS, LS-FAS-Cu were further characterized by FTIR. As shown in Figure 2, the broad and strong absorption peak at around 3000 cm^−1^ was associated with the stretching vibration of the –OH group in the skeleton of LS [16]. The tiny peak at approximately 2900 cm^−1^ was assigned to the stretching vibration of the −CH_2_− moiety. The characteristic peak of the aromatic benzene ring appeared at about 1500 cm^−1^ [17]. The vibration bands at 1000−1200 cm^−1^ were attributed to the −SO_3_ and O−S−O stretching of the −SO_3_Na (or −SO_3_H) group [18–19]. Grafting of the FAS moiety changed the peak pattern of –SO_3_Na in LS-FAS material, and the immobilization of the Cu complex shifted the corresponding peaks to a higher wavenumber position, implying the successful coordination of LS-FAS to the Cu complex.

**Figure 3:**
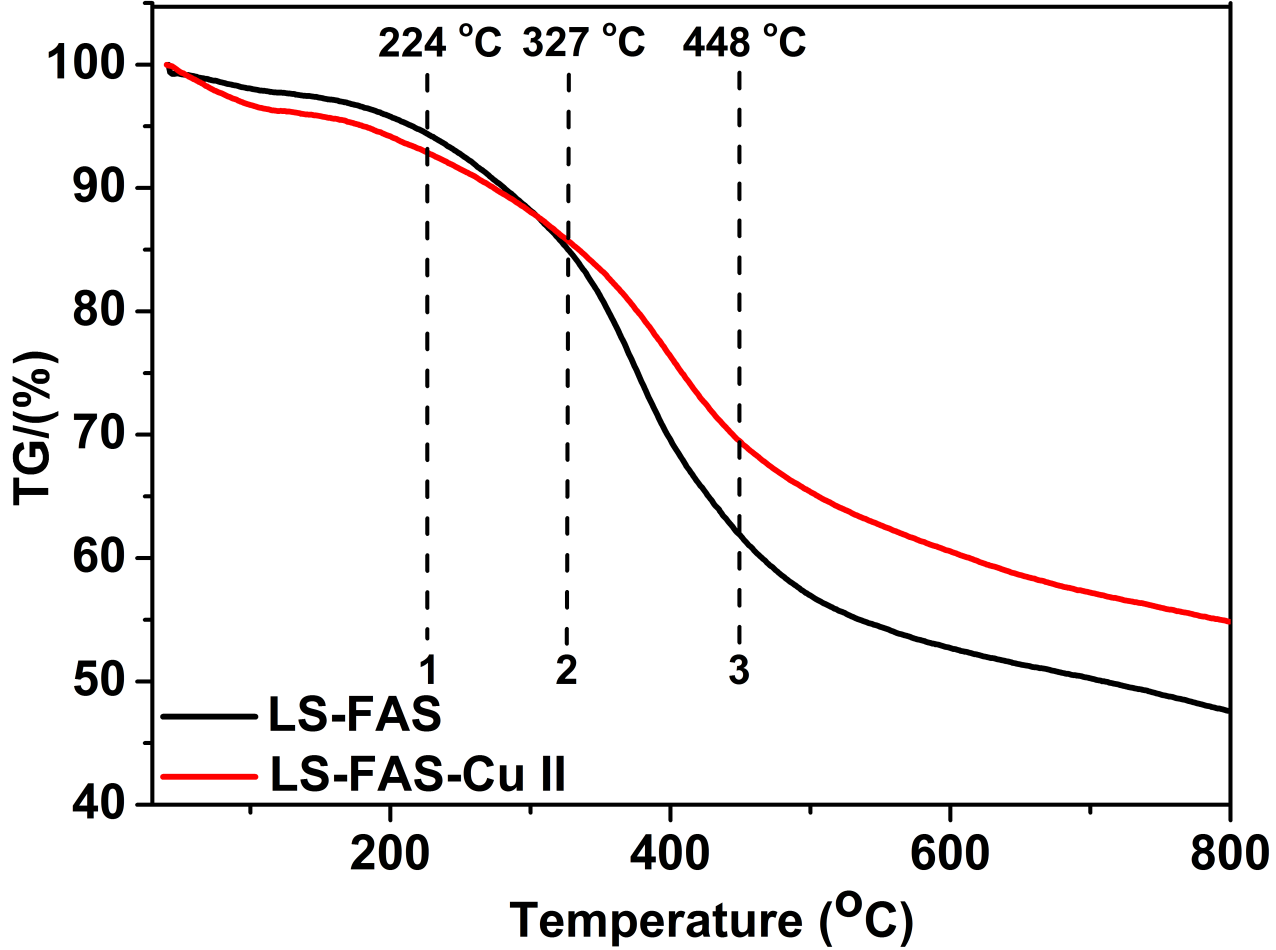
Thermogravimetric weight loss of the obtained materials LS-FAS and LS-FAS-Cu.

The thermal behavior of LS-FAS and LS-FAS-Cu was investigated by TG in a temperature range of 40–800 °C (Figure 3). When the temperature was lower than 200 °C, both of them exhibited good stability with a slight drop of the curves, possibly due to the loss of a trace amount of absorbed water [20–21]. Two sharp weight losses were identified on the TG curves as temperature rose. The first loss within 224–327 °C may be caused by decomposition and elimination of the –SO_3_Na groups and the introduced small organic species in the materials [22], while the second loss at higher temperature of 327–448 °C may be attributed to decomposition of the support skeleton [23]. The thermal stability of referential Resin-Cu catalyst was also investigated by TG analysis (Supporting Information File 1, Figure S3), showing a high thermal stability as well [24–26]. The above results indicated that all prepared materials could remain stable in the system when used to catalyze organic reactions.

**Figure 4:**
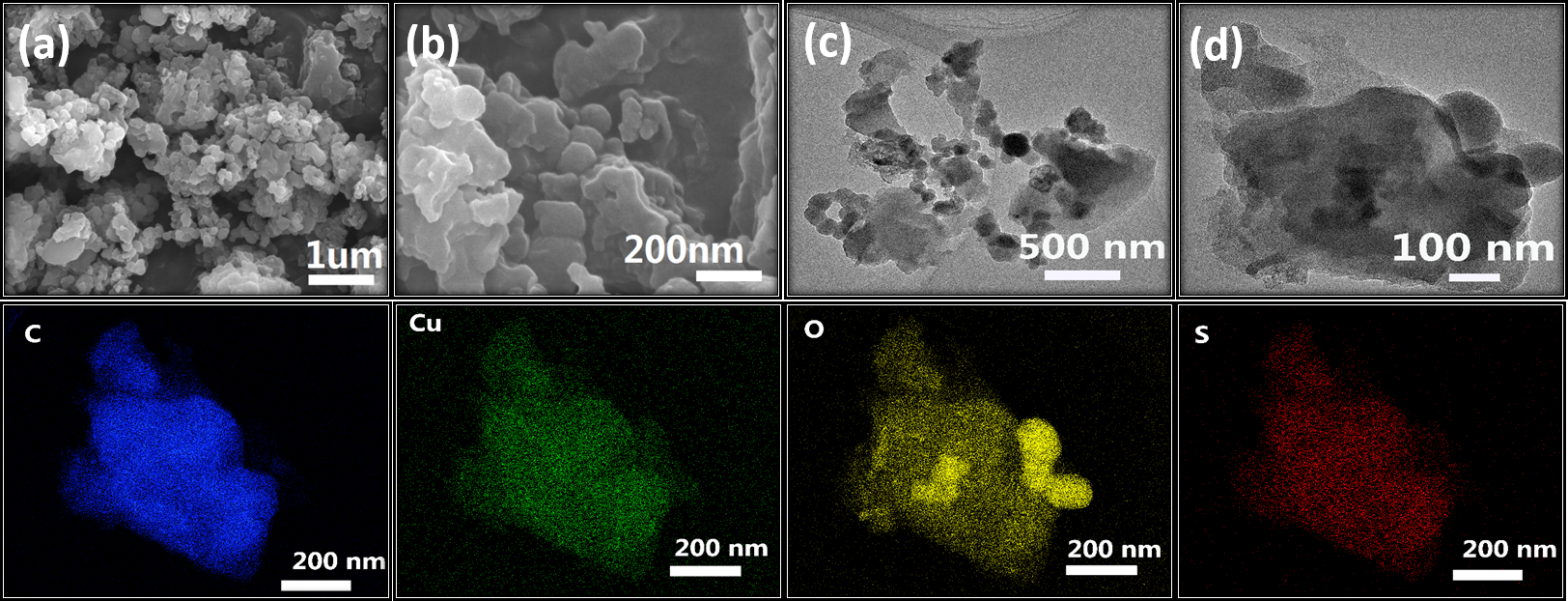
FSEM imagine of LS-FAS-Cu in different scale label a) 1 μm, b) 200 nm; FTEM images of LS-FAS-Cu in 500 nm (c) and 100 nm (d); and the elemental mapping of LS-FAS-Cu for C, Cu, O and S elements.

FSEM (field emission scanning electron microscopy) and FTEM (field emission transmission electron microscopy) were used to observe the surface morphologies of different catalysts, and the results are presented in Figure 4. The LS-FAS-Cu catalyst featured irregular and blocky morphology with uneven size from 100 nm to 400 nm (Figure 4a–d). In addition, the elemental mapping images clearly revealed the presence and uniform distribution of C, Cu, O, and S in LAS-FAS-Cu.

**Figure 5:**
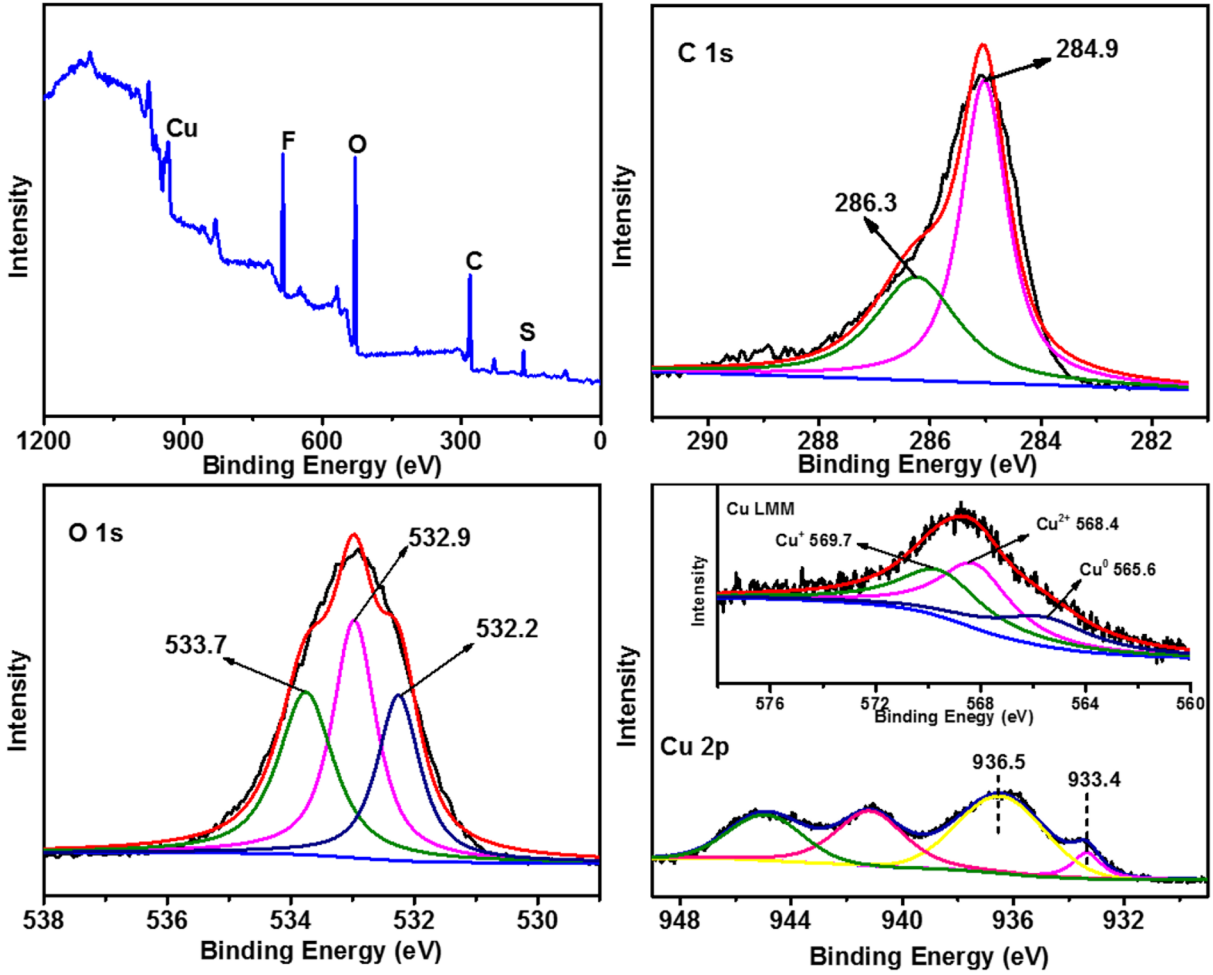
XPS spectra of LS-FAS-Cu in the regions of C 1s, O 1s, Cu 2p_3/2_ and Cu LMM (inset).

XPS was further utilized to analyze the chemical states of the elements on the surface of the catalyst (Figure 5). The wide survey spectrum of LS-FAS-Cu showed that all of the essential elements could be detected. In the high-resolution spectrum of C 1s, the peak at 284.9 eV was ascribed to C–C, and the peak at 286.3 eV corresponded to C–O/C–S [27–28]. The O 1s spectrum clearly evidenced the presence of oxygen atoms with three kinds of chemical environments: the peaks at 532.9 eV and 532.2 eV were attributed to –OH and –C–O– groups, respectively, while the peak at 533.7 eV was attributed to –SO_3_ [12]. In the spectrum of Cu 2p_3/2_, the peak at ≈936 eV was assigned to Cu^2+^ in the spinel, accompanied by the characteristic Cu^2+^ shakeup satellite peaks at 938−948 eV, while the peak at ≈933 eV suggested the presence of Cu^+^ and/or Cu^0^ species. Because Cu 2p_3/2_ XPS cannot differentiate between Cu^+^ and Cu^0^, Auger Cu LMM spectra were further recorded, and the results confirmed the presence of Cu^+^ at ≈570 eV, while Cu^0^ at 565.6 eV [29–31], meaning that Cu^2+^ species were partially reduced during the course of immobilization.

### Catalytic activity of the catalysts

With the catalysts in hand, we investigated their catalytic activity in organic reactions. Tricyclic indole alkaloids bearing 3,4-fused seven-membered rings have attracted much attention because of their interesting molecular architectures and important biological activities [32–33]. Here the three-component reaction of 4-aminoindole (**1a**), 4-methylbenzaldehyde (**2a**) and diethyl acetylenedicarboxylate (**3a**) was performed to construct the seven-membered indole ring system with the aid of the LS-FAS-Cu catalyst, and the results are summarized in Table 1. At the beginning, the three-component reaction was conducted without the presence of any catalyst, but no products were formed (Table 1, entry 1). After screening different kind of solvents (Table 1, entries 2–8) at 60 °C, EtOH was found to be the best one, and the target product **4a** was obtained in 86% yield (Table 1, entry 8). The referential catalysts LS-FM-Cu and Resin-Cu showed inferior catalytic activity for this reaction, probably attributed to the low content of Cu species in the materials (Table 1, entries 9 and 10). Further investigation revealed that the reaction was also affected by the dosage of catalyst and the temperature. The yield was decreased significantly with the catalyst dosage decreasing (Table 1, entry 8 and 13). Decreasing the temperature will result in a significant loss of yields (Table 1, entry 11), prolong the time could increase the yield but still lower than that at 60 °C (Table 1, entry 12). Thus the optimal conditions were confirmed to be 20 mol % of LS-FAS-Cu (the Cu loading with reference to the substrates **1a**), 60 °C and 7 h. To our delight, the reaction could be easily scaled up to 10 mmol without significant loss of the efficiency and selectivity (Table 1, entry 14).

**Table 1:** Three-component reaction of **1a**, **2a**, and **3a** to synthesis of **4a**^a^.

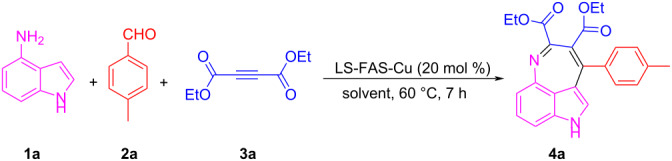		

Entry	Solvent	Yield [%]^b^

1^c^	EtOH	0
2	AcOH	0
3	MeOH	59
4	THF	21
5	DMSO	trace
6	H_2_O	0
7	CH_3_NO_2_	35
8	EtOH	86
9^d^	EtOH	62
10^e^	EtOH	57
11^f^	EtOH	49
12^g^	EtOH	71
13^h^	EtOH	37
14^i^	EtOH	84

^a^Reaction conditions: **1a**/**2a**/**3a** = 1.5:1:1.5; 1.0 mL. ^b^Isolated yields. ^c^Without the catalyst. ^d^LS-FM-Cu was used. ^e^Resin-Cu was used. ^f^40 °C. ^g^40 °C, 24 h. ^h^10 mol % LS-FAS-Cu was used. ^i^10 mmol scale reaction.

**Scheme 1:**
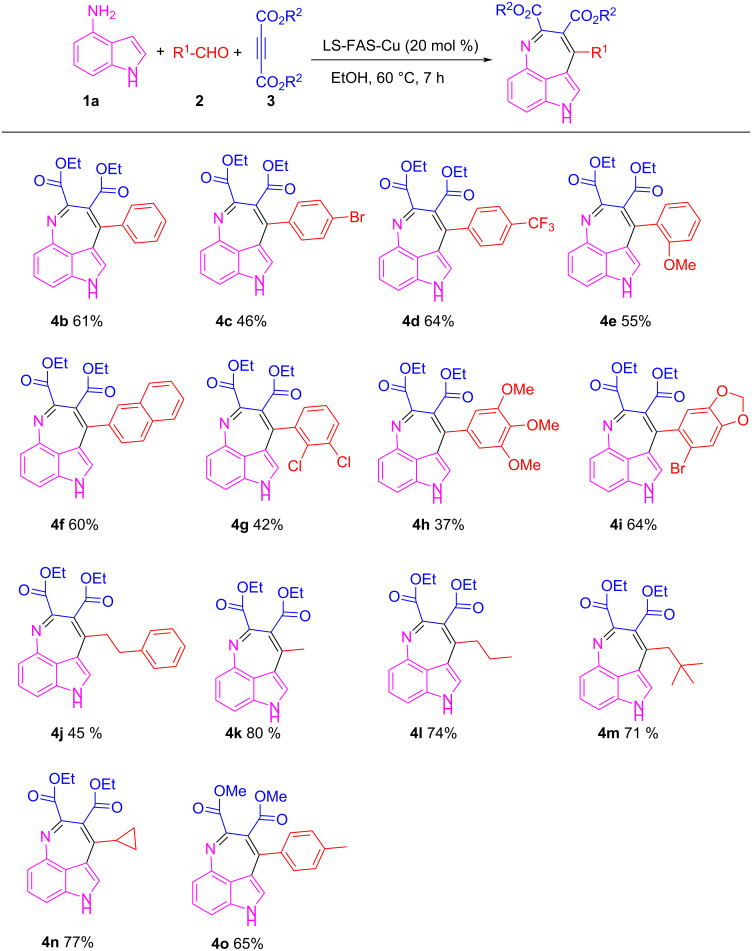
Substrate scope of LS-FAS-Cu catalyzed three-component reactions of 4-aminoindoles, alkynes and aldehydes.

Under the optimal conditions, the substrate scope of the model reaction was extended and the results were shown in Scheme 1. Aldehydes **2** with different functional groups on the benzene ring could react smoothly with compounds **1a** and **3a**, producing the corresponding 3,4-fused tricyclic indoles **4b**–**d** with yields ranging from 46% to 64%. *o*-Anisaldehyde, with steric-hindrance effect, also reacted efficiently in this reaction and gave product **4e** in 55% yield. 2-Naphthaldehyde also proceeded well with **1a** and **3a** and the product **4f** was isolated in 60% yield. Aldehydes with multi-substituted functional groups also worked well in this reaction and the corresponding products were obtained in moderate yields (**4g** and **4h**). It should be noted that heterocyclic aldehydes such as 2-bromo-4,5-methylenedioxybenzaldehyde (**2i**) could also successfully engage in this reaction and the yield of product **4i** was up to 64%. In the following investigation, the aliphatic aldehydes **2j–m** were also successfully reacted, and the products **4j**–**m** were obtained in good to excellent yields. The acid-labile cyclopropanecarboxaldehyde (**2n**) could also participate well, and the product **4n** was obtained in 77% yield without damage of the cyclopropane structure. Dimethyl acetylenedicarboxylate (**3b**), **1a** and **2a** could tolerate the LS-FAS-Cu-promoted conditions as well, and gave the product **4o** in 65% yield. The successful attempts in the three-component reaction of 4-aminoindoles (**1a**), alkynes and aldehydes indicate that the heterogeneous catalyst LS-FAS-Cu is competent for catalyzing nitrogen-containing heterocyclic compounds without significant damage of the Cu species.

The pyridine-containing moiety, such as arylpyridines, widely exist in natural products, pharmaceutical agents and functional materials [34–37]. Traditional methods for synthesizing pyridine-containing derivatives include condensation reactions, cross-coupling, ring-closing, metathesis, cycloadditions, radical reactions and microwave-assisted reactions [38–40]. In this work, we attempt to develop a greener, simpler, more efficient and recyclable system to synthesize arylpyridine derivatives. Initially, the reaction of acetophenone (**5a**) and 1,3-diaminopropane (**6a**) was conducted with the aid of LS-FAS-Cu and TsOH·H_2_O in a variety of solvents at 100 °C for 24 h under an oxygen atmosphere (Table 2, entries 1–6). After comparing the yield, EtOH was found to be the optimal solvent, generating the target product 2-arylpyridine **7a** in 75% yield. Other kinds of solvents were ineffective (Table 2, entries 1–5 vs entry 6). Only the addition of TsOH·H_2_O showed a reluctant activity in this organic transformation (Table 2, entry 7). When homogeneous Cu(OTf)_2_ was used, only 20% of **7a** was isolated (Table 2, entry 8). The two referential catalysts LS-FM-Cu and Resin-Cu exhibited low catalytic efficiencies in the model reaction, only 60% and 48% of **7a** were obtained, respectively (Table 2, entries 9 and 10). The results indicated that the yields were also greatly affected by the catalyst loading and proper acid additives. In the same solvent, the yield decreased with the decreasing of the catalyst loading (Table 2, entry 6 vs entries 9 and 10).

**Table 2:** Optimizing the reaction condition of acetophenones and 1,3-diaminopropane to synthesis 2‑arylpyridine derivatives.^a^

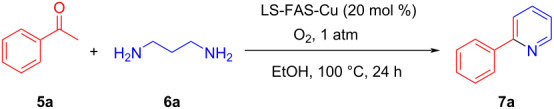		

Entry	Solvent	Yield [%]^b^

1	CH_3_CN	30
2	toluene	5
3	CH_2_Cl_2_	NR
4	H_2_O	trace
5^c^	THF	trace
6	EtOH	75
7^d^	EtOH	NR
8^e^	EtOH	20
9^f^	EtOH	60
10^g^	EtOH	48
11^h^	EtOH	74

^a^Reaction conditions: **5a** (0.2 mmol), **6a** (0.6 mmol), solvent (1.0 mL), 0.6 equiv TsOH·H_2_O, O_2_ (1 atm), 24 h. ^b^Isolated yield. ^c^60 °C. ^d^Only 0.5 equiv TsOH·H_2_O was used. ^e^Cu(OTf)_2_ (20 mol %) was used. ^f^Referential catalyst LS-FM-Cu (20 mol %) was used. ^g^Referential catalyst Resin-Cu (20 mol %) was used. ^h^Catalyst: LSA-FAS-Cu [Cu (40 mol %), –SO_3_H (0.5 equiv)], 26 h.

In order to utilize the residual –SO_3_Na groups in LS-FAS-Cu after immobilization of the Cu species, LS-FAS-Cu were further acidized by sulfuric acid solution (2 M) and denoted as LSA-FAS-Cu. The amount of –SO_3_H was determined through acid-base titration and elemental analysis, respectively (Table 3). The results showed that the density of –SO_3_H determined by acid-base titration was much lower than that confirmed by elemental analysis, indicating that a portion of sulfur existed in the form of Cu complex and –SO_3_Na. Afterwards, the catalytic activity of LSA-FAS-Cu in the model reaction was investigated under the optimal conditions except for the absence of TsOH**^.^**H_2_O. Considering that the density of –SO_3_H in LSA-FAS-Cu was a half of TsOH**^.^**H_2_O, so the amount of LSA-FAS-Cu should be doubled. To our delight, although the reaction time was prolonged a little, LSA-FAS-Cu (Cu 40 mol %, –SO_3_H 0.5 equiv) could also promote the reaction smoothly and generated **7a** in 74% yield (Table 2, entry 11), suggesting that the acidification process enabled the catalyst to catalyze the model reaction without addition of TsOH**^.^**H_2_O.

**Table 3:** Acid density of catalyst.

Sample	Totally S content^a^ (mmol/g)	SO_3_H density^b^ (mmol/g)

LSA-FAS-Cu	2.08	1.16

^a^Determined by EA. ^b^Determined by acid−base titration.

Using LSA-FAS-Cu as catalyst, the substrate scope of the reaction was subsequently investigated, and it was found that the reaction could tolerate a wide range of functionalities, including fluoro, chloro, iodo, cyclohexane and benzyloxy moieties. Acetophenones with an electron-donating group in the *para*-position of the aromatic ring afforded the target products in better yields than the electron-withdrawing groups (Table 4, **7b**−**j** and **7k**–**m**). 3'-Methylacetophenone (**5n**) and 2-acetonaphthone (**5o**) could also react with **6a**, and give the desired product **7n** and **7o** in 65% and 58% yield, respectively. Some disubstituted and trisubstituted acetophenones were also examined, and most of them could generate the desired products in moderate yields (**7p**−**t**). It was noted that heterocycles-substituted ketones also showed high reactivity in this reaction, and the corresponding products were obtained in good yields (**7u**–**w**).

**Table 4:** Substrate scope of the ketones catalyzed by LSA-FAS-Cu.

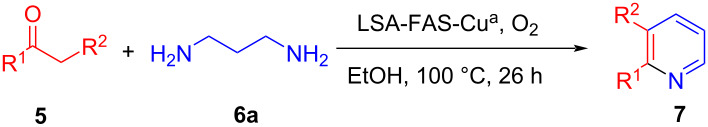					

Entry	Product	Yield^b^ (%)	Entry	Product	Yield^b^ (%)

1	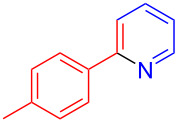 **7b**	71	12	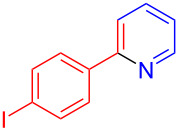 **7m**	39
2	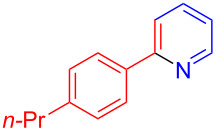 **7c**	70	13	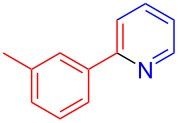 **7n**	65
3	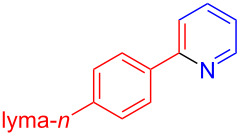 **7d**	68	14	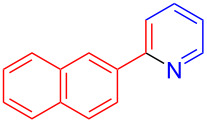 **7o**	58
4	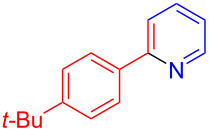 **7e**	63	15	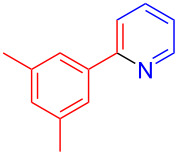 **7p**	68
5	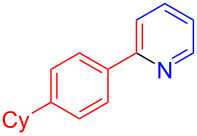 **7f**	60	16	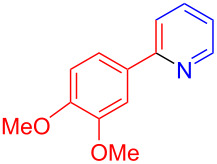 **7q**	56
6	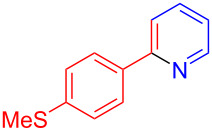 **7g**	82	17	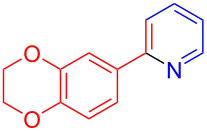 **7r**	64
7	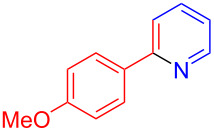 **7h**	65	18	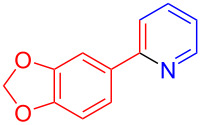 **7s**	71
8	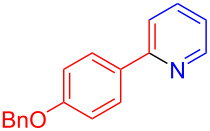 **7i**	67	19	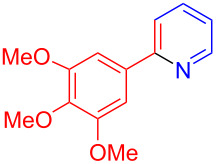 **7t**	73
9	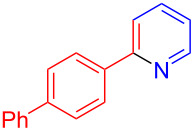 **7j**	63	20	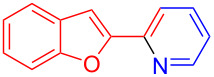 **7u**	76
10	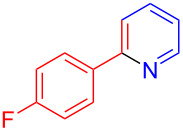 **7k**	45	21	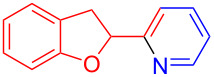 **7v**	75
11	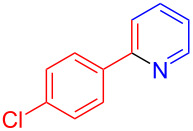 **7l**	40	22	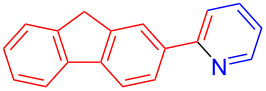 **7w**	69

^a^LSA-FAS-Cu [Cu (40 mol %), –SO_3_H (0.5 equiv). ^b^Isolated yields.

In the following investigation, the LS-FAS-Cu was also found to be an efficient catalyst for the synthesis of aminonaphthalene derivatives (Table 5). The substituent effect of aniline was examined systematically, and the results showed that anilines bearing electron-donating groups such as Me, OMe and *t*-Bu at the *para*-position could convert smoothly and give the corresponding products in excellent yields (**10a**–**c**). The anilines with electron-withdrawing substituents such as 4-bromoaniline (**9d**) worked sluggishly and only a moderate yield of **10d** was obtained. Disubstituted anilines also tolerated the catalytic system, generating the product **10e** in 66% yield. Naphthylamine (**9f**) also reacted positively with 2-(phenylethynyl)acetophenone (**8a**) and 70% of **10f** was obtained. The attempts of aliphatic amines were also successful, obtaining the corresponding products **10g**–**i** in good to excellent yields. It should be noted that secondary amines such as morpholine (**9j**) also showed high reactivity in this reaction and **10j** was obtained in 81% yield. The substituent effect of 2-(phenylethynyl)acetophenone was studied subsequently, and the results showed that fluoro, chloro, aliphatic chain and cycloolefin-substituted 2-(phenylethynyl)acetophenone could react smoothly, generating the target products **10k**–**o** in excellent yields. Unfortunately, aniline substituted with a strong electron-withdrawing group at the *para*-position and 2-(phenylethynyl)acetophenone substituted with an electron-donating group were reluctant to react under this catalytic system, and no desired products were determined (**10p** and **10q**).

**Table 5:** LS-FAS-Cu catalyzed synthesis of aminonaphthalene derivatives.^a^

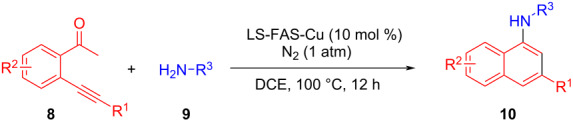					

Entry	R^1^	R^2^	R^3^	Product	Yield^b^ (%)

1	Ph	H	4-MeC_6_H_4_	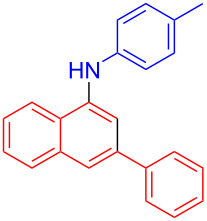 **10a**	81, (65)^c^, (58)^d^
2	Ph	H	4-OMeC_6_H_4_	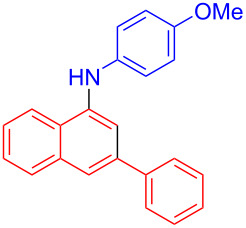 **10b**	85
3	Ph	H	4-*t*-BuC_6_H_4_	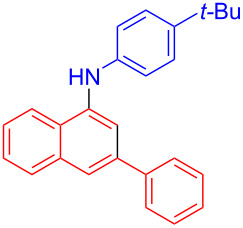 **10c**	79
4	Ph	H	4-BrC_6_H_4_	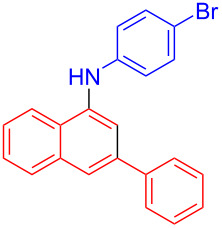 **10d**	55
5	Ph	H	3,4-OMeC_6_H_3_	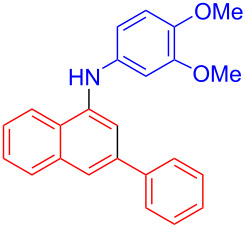 **10e**	66
6	Ph	H	1-naphthalene	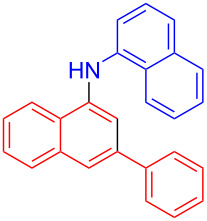 **10f**	70
7	Ph	H	Bn	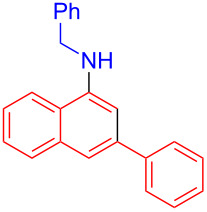 **10g**	61
8	Ph	H	*n*-Bu	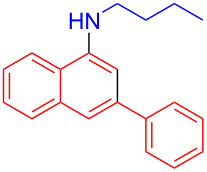 **10h**	83
9	Ph	H	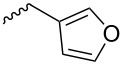	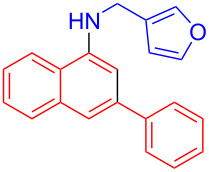 **10i**	88
10	Ph	H	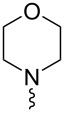	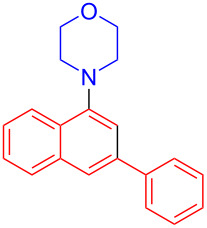 **10j**	81
11	Ph	Cl	4-MeC_6_H_4_	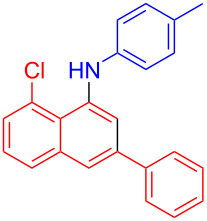 **10k**	78
12	Ph	F	4-MeC_6_H_4_	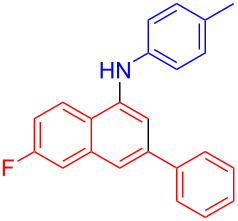 **10l**	63
13	4-FC_6_H_4_	H	4-MeC_6_H_4_	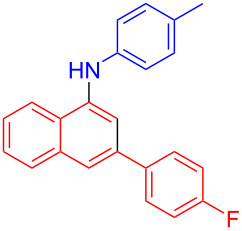 **10m**	71
14	*n*-hexyl	H	4-MeC_6_H_4_	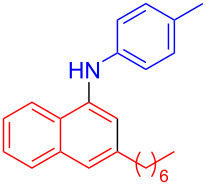 **10n**	54
15	cyclohexenyl	H	4-MeC_6_H_4_	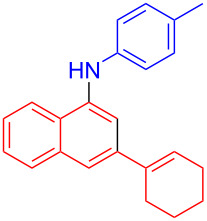 **10o**	42
16	Ph	H	4-NO_2_C_6_H_4_	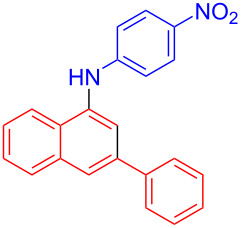 **10p**	n.d.
17	4-OMeC_6_H_4_	H	4-MeC_6_H_4_	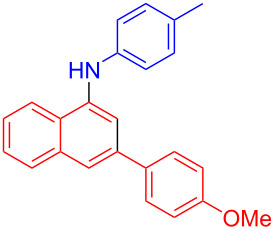 **10q**	n.d.

^a^Reaction conditions: **8** (0.2 mmol), **9** (0.24 mmol), DCE (1.0 mL), N_2_, 12 h. ^b^Isolated yields. ^c^LS-FM-Cu (10 mol % was used) ^d^Resin-Cu (10 mol %) was used.

Substituted isoquinoline derivatives are considered as an important class of N-heterocyclic compounds, showing attractive physiological, biological and pharmacological activities [41–45]. Therefore, the feasibility of synthesizing isoquinoline derivatives using LSA-FAS-Cu as catalyst was investigated. As shown in Table 6, LS-FAS-Cu could promote 2-(phenylethynyl)benzaldehyde (**11a**) and urea (**12a**) to generate 3-phenylisoquinoline (**13a**) in excellent yield, while the referential catalysts showed inferior catalytic activity, which may be ascribed to the low loading capacity of Cu species (Table 6, entry 1 vs entries 2 and 3). In addition, by increasing the amount of catalyst, the yields could reach the ideal level (Table 6, entries 4 and 5).

**Table 6:** Synthesis of the 3-phenylisoquinoline from **11a** and urea (**12a**).^a^

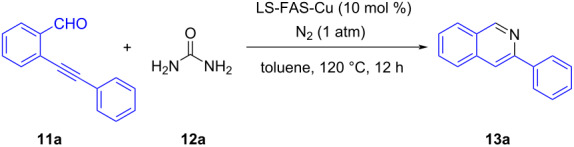		

Entry	Catalyst	Yield [%]^b^

1	LS-FAS-Cu	85%, (84%)^3rd cycle^
2	LS-FM-Cu	74%
3	Resin-Cu	65%
4^c^	LS-FM-Cu	81%
5^c^	Resin-Cu	79%

^a^**11a**:**12a** = 1:1.2. ^b^Isolated yield. ^c^20 mol % of catalyst was used.

**Figure 6:**
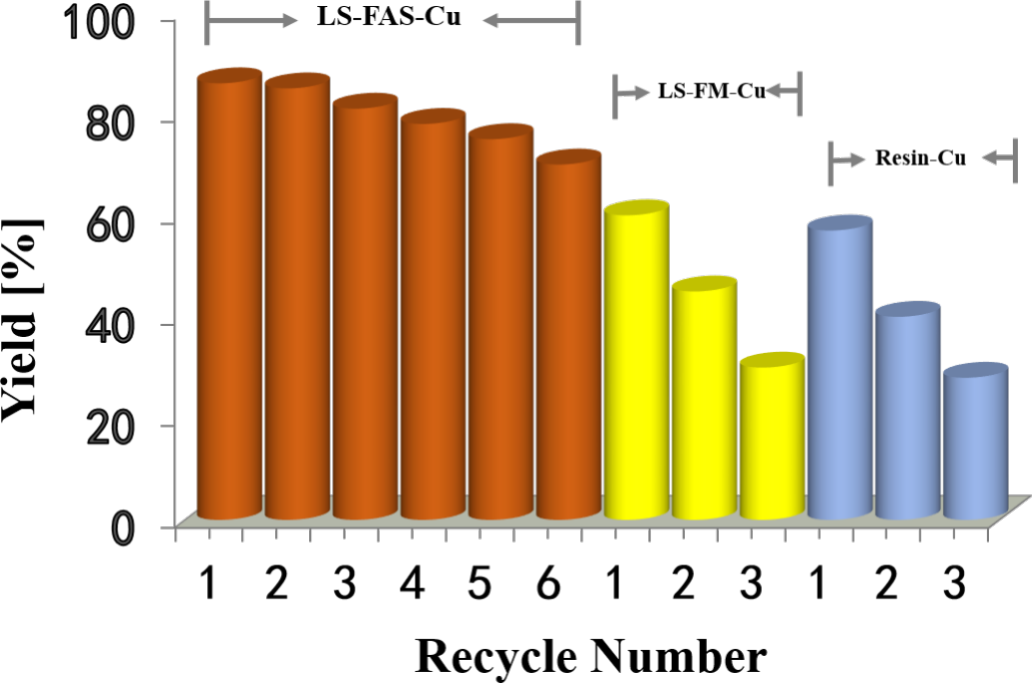
Recyclability of LS-FAS-Cu, LS-FM-Cu and Resin-Cu in the reaction between compounds **1a**, **2a** and **3a**.

The recyclability of LS-FAS-Cu was investigated based on the three-component reaction of 4-aminoindole (**1a**), 4-methylbenzaldehyde (**2a**) and diethyl acetylenedicarboxylate (**3a**). In order to confirm the heterogeneity of this reaction, we investigated the Cu leaching during the reaction process. The reaction mixture (including the catalyst) was allowed to stir for a period of time firstly, and then the catalyst was isolated by hot filtration. The remaining liquid mixture was divided into two portions, one was isolated and used to calculate the target product yield, another one stirred for a period of time once again, and the latter did not show an increase in the yield. The Cu content in the reaction mixture after catalyst separation was confirmed to be 2.78 ppm by ICP. Besides, also with the aid of ICP-MS analysis, we found that there was no obvious change in the Cu content of the catalyst before and after the reaction (0.920 mmol/g vs 0.918 mmol/g). The above results not only verified the heterogeneous property of LS-FAS-Cu catalyst, but also implied that the loaded Cu species did not leach into the reaction system during the reaction process. The results associated with the recyclability of LS-FAS-Cu and two referential catalysts were summarized in Figure 6. After six runs of recycling (Figure 6a), LS-FAS-Cu was still capable of catalyzing the model reaction in 70% yield, indicating that the catalyst was robust and stable under the reaction conditions and could be recycled without obvious loss of catalytic activity. The slight decreasing of yield maybe caused by the mass loss of the catalyst during the recovery process (mass recovery 97.5%). In stark contrast, the two referential catalysts showed inferior recyclability (Figure 6b and 6c) not only because the mass loss but also the low stability and capacity of the metals.

## Conclusion

A robust heterogeneous Cu catalyst was successfully prepared through immobilizing Cu on a novel and ecofriendly supporting material synthesized by the phenyl–aldehyde condensation reaction of FAS and LS. This catalyst could be used for the synthesis of several nitrogen-containing heterocycles and exhibited excellent catalytic activity. ICP-MS data showed that grafting of FAS on LS greatly increased the loading capability of the Cu species, which was considered to be responsible for the enhanced catalytic performance of the catalyst. This catalyst demonstrated a satisfying recyclability and could be reused several times without significant loss in activity. It is anticipated that this catalyst would have a broad application prospect considering the low cost and availability of the raw materials, as well as the facile preparation, multi-functionalities and recyclability of the catalyst.

## Experimental

### Experimental instrumentation

The chemical composition of the samples war characterized by Fourier transform infrared spectroscopy (FTIR, EQUINOX 55, Bruker) in the wavenumber range of 4000–400 cm^−1^ and X-ray photoelectron spectroscopy (XPS, AXIS-ULTRA DLD-600W, SHIMADZU) at a base pressure of 2 × 10^−9^ Pa. Elemental analyses (EA) were conducted using a Vario Micro cube Elemental Analyzer (Elementar, Germany). Thermogravimetric analyses (TGA) were performed under N_2_ atmosphere by heating the materials from room temperature to 800 °C at a rate of 10 °C·min^−1^. Before testing, all samples were degassed at 110 °C for 8 h under vacuum (10^−5^ bar) conditions. ICP-MS data were recorded on ELAN DRC-e device. The morphologies of samples were observed by scanning electron microscopy (SEM, Sirion 200, Holland) equipped with an energy dispersive X-ray (EDX) spectroscopy and transmission electron microscopy (TEM, Talos F200X), respectively. ^1^H and ^13^C NMR spectra were recorded on Bruker AV-400 spectrometer.

### Catalyst preparation procedure

Typically, 3.0 g of LS were dissolved in 5.0 mL of deionized water, followed by adding 0.9 g of FAS. After the addition of 3.0 mL of concentrated HCl (37 wt %), the solution was continuously stirred at 90 °C for 8 h. Subsequently, the so-obtained support (denoted as LS-FAS) was filtered off, washed to be neutral and dried at 110 °C for 10 h.

## Supporting Information

File 1 Characterization data, copies of NMR spectra and the preparation of the referential catalysts.
