# Supplementary material for: A novel and robust heterogeneous Cu catalyst using modified lignosulfonate as support for the synthesis of nitrogen-containing heterocycles
Source: Beilstein J Org Chem. 2020 Nov 26;16:2888–902. doi: 10.3762/bjoc.16.238 (PMC7705867; doi:10.3762/bjoc.16.238)
Supplement: File 1 — Characterization data, copies of NMR spectra and the preparation of the referential catalysts. [file Beilstein_J_Org_Chem-16-2888-s001.pdf]

BEILSTEIN JOURNAL OF ORGANIC CHEMISTRY

Supporting Information

for

A novel and robust heterogeneous Cu catalyst using modified lignosulfonate as support for the synthesis of nitrogen-containing heterocycles

Bingbing Lai, Meng Ye, Ping Liu, Minghao Li, Rongxian Bai and Yanlong Gu

Beilstein J. Org. Chem. **2020,** *16,* 2888–2902. doi:10.3762/bjoc.16.238

Characterization data, copies of NMR spectra and the preparation of the referential catalysts

License and Terms: This is a supporting information file under the terms of the Creative Commons Attribution License (https://creativecommons.org/licenses/by/4.0). Please note that the reuse, redistribution and reproduction in particular requires that the authors and source are credited.
The license is subject to the *Beilstein Journal of Organic Chemistry* terms and conditions: (https://www.beilstein-journals.org/bjoc)

1. General remarks

Sodium lignosulfonate, o-anisaldehyde, propionaldehyde and 1,3-diaminopropane were purchased from Aladdin Industrial Corporation. 2-Formylbenzenesulfonic acid sodium salt, 4-(trifluoromethyl)benzaldehyde, 1-(4-propylphenyl)ethan-1-one and 3,4-dimethoxyaniline were purchased from Adamas Reagent, Ltd. Copper(II) trifluoromethanesulfonate, benzaldehyde, formaldehyde, acetophenone, 1-aminonaphthalene, butylamine and morpholine were purchased from Sinopharm Chemical Reagent Co., Ltd. 4-Aminoindole, 4'-*n*-amylacetophenone, 6-acetyl-1,4-benzodioxane and 3,4-methylenedioxyacetophenone were purchased from Bide Pharmatech Ltd. p-Tolualdehyde, 4'-n-amylacetophenone, 4-fluoroacetophenone, 5-acetyl-2,3-dihydrobenzo(*b*)furan and 4-bromoaniline were purchased from Accela Chem Bio Co., Ltd. Dimethyl acetylenedicarboxylate, 4-bromobenzaldehyde, 2-naphthaldehyde, 2,3-dichlorobenzaldehyde, 3,4,5-trimethoxybenzaldehyde, 2-bromo-4,5-methylenedioxybenzaldehyde, phenylpropylaldehyde, 3,3-dimethylbutyraldehyde, cyclopropanecarboxaldehyde, dimethyl acetylenedicarboxylate, 4'-methylacetophenone, 4'-cyclohexylacetophenone, 4'-methylthioacetophenone, 4'-methoxyacetophenone, 4'-benzyloxyacetophenone, 4-acetylbiphenyl, 4'-chloroacetophenone, 4`-iodoacetophenone, 3'-methylacetophenone, 3,4-dimethoxyacetophenone, 3',4',5'-trimethoxyacetophenone, 2-acetylfluorene, *p*-anisidine and urea were purchased from Energy Chemical Co., Ltd. 2-Acetonaphthone, 2',5'-dimethylacetophenone, 2-acetylbenzofuran, benzylamine and furfurylamine were purchased from Alfa Aesar Chemical Company. *p*-Toluidine was purchased from Jinshan Chemical Co., Ltd. 4-*tert*-Butylaniline was purchased from Heowns Biochem Technologies Co., Ltd.

2. Supplementary experiment section

2.1 Preparation of the referential catalyst LS-FM-Cu

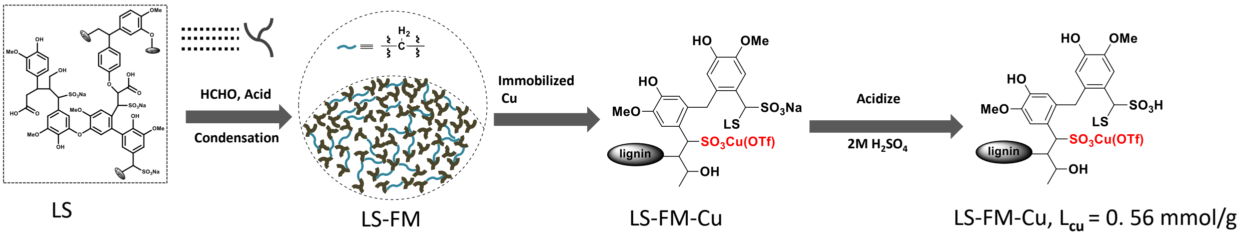

Fig. S1 Preparation of the referential catalyst LS-FM-Cu in this work.

2.2 Preparation of the referential catalyst Resin-Cu

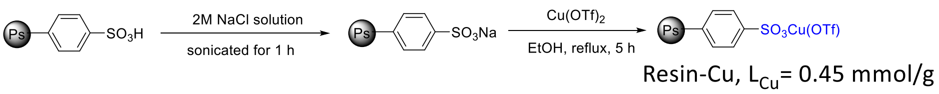

Fig. S2 Preparation of the referential catalyst Resin-Cu in this work.

2.3 Elemental analysis of the composites in this work

Table S1 Elemental analysis of the composite in this work.

Composite	Weight [mg]	Method	C [%]	H [%]	S [%]
LS	2.1080	2mg80s	30.84	3.658	2.626
LS-FAS	2.3860	2mg80s	53.79	4.851	6.650

2.4 Thermo gravimetric weight loss of the obtained referential catalyst Resin-Cu

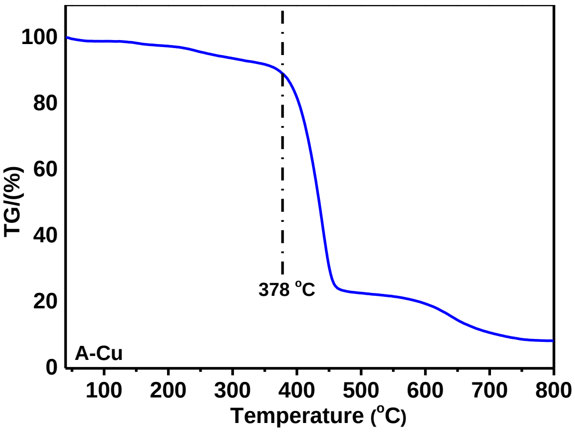

Fig. S3. Thermogravimetric weight loss of the obtained referential catalyst Resin-Cu.

2.5 Typical procedure for the synthesis of tricyclic indole alkaloids bearing 3,4-fused seven-membered rings (4a as an example)

In a V-type reaction flask which was equipped with magnetic stirring, 4-aminoindole (**1a**, 0.3 mmol, 39.6 mg), 4-methylbenzaldehyde (**2a**, 0.2 mmol, 24.0mg), diethyl acetylenedicarboxylate (**3a**, 0.3 mmol, 51.0 mg) and the catalyst LS-FAS-Cu (20 mol %, 43.5 mg) were added to 1.0 mL EtOH and the temperature of the mixture was allowed to increase to 60 °C. The reaction process was monitored by PTLC, after its completion the solution was centrifuged. The organic phase was further purified by preparative TLC using a mixture of ethyl acetate and petroleum ether as eluent (EA/PE 1:5 v/v), thus the product **4a** (69.2 mg, 86%) was obtained.

2.6 Typical procedure for the synthesis of 2-arylpyridine derivatives (7a as an example) [1]

In a 10 mL V-type reaction flask which was equipped with magnetic stirring, acetophenone **5a** (0.2 mmol, 24.0 mg), 1,3-diaminopropane (**6a**, 0.6 mmol, 44.5 mg), LS-FAS-Cu (20 mol %, 43.5 mg) and TsOH·H_2O (0.12 mmol, 22.8 mg) were added into 1.0 mL EtOH solution. The mixture was stirred for 24 hours at 100 °C under oxygen atmosphere. The reaction process was monitored by PTLC, after its completion the solution was centrifuged. The organic phase was further purified by preparative TLC using a mixture of ethyl acetate and petroleum ether as eluent (EA/PE 1:3 v/v), thus the product **7a** (23.3 mg, 75%) was obtained. In the substrate scope extending section, the catalyst was changed to LSA-FAS-Cu [Cu (40 mol %), -SO_3H (0.5 equiv)] without the presence of TsOH.H_2O.

2.7 Typical procedure for the synthesis of aminonaphthalenes derivatives (10a as an example) [2]

In a 10 mL V-type reaction flask which was equipped with magnetic stirring, 1-(2-(phenylethynyl)phenyl)ethan-1-one (**8a**, 0.2 mmol, 44.1 mg), *p*-toluidine (**9a**, 0.24 mmol, 25.7 mg) and catalyst LS-FAS-Cu (10 mol %, 21.7 mg) were added to 1.0 mL DCE solvent. The mixture solution was stirred for 12 hours at 100 °C under N_2 atmosphere. The reaction process was monitored by PTLC, after its completion the solution was centrifuged. The organic phase was further purified by preparative TLC using a mixture of ethyl acetate and petroleum ether as eluent (EA/PE 1:3 v/v), thus the product **10a** (50.1 mg, 81%) was obtained.

2.8 Typical procedure for the synthesis of 3-phenylisoquinolines derivatives 13a [3]

In a 10 mL V-type reaction flask which was equipped with magnetic stirring, 2-(phenylethynyl)benzaldehyde (**11a**, 0.2 mmol, 41.2 mg), urea (**12a**, 0.4 mmol, 24.0 mg) and the catalyst LS-FAS-Cu (10 mol %, 21.7 mg) were added to 1.0 mL Toluene solvent. The mixture solution was stirred for 12 hours at 120 °C under N_2 atmosphere. The reaction process was monitored by PTLC, after its completion the solution was centrifuged. The organic phase was further purified by preparative TLC using a mixture of ethyl acetate and petroleum ether as eluent (EA/PE 1:3 v/v), thus the product **13a** (34.9 mg, 85%) was obtained.

3. Spectroscopic data for the compounds

Diethyl 4-(*p*-tolyl)-6*H*-azepino[4,3,2-*cd*]indole-2,3-dicarboxylate (4a) Yellow solid, mp: 167–169 °C, ^{1}H NMR (400 MHz, $CDCl_3$, 25 °C, TMS): δ = 9.16 (d, *J* = 8.8 Hz, 1H), 7.65 (dd, *J* = 8.4, 7.0 Hz, 3H), 7.53 (d, *J* = 9.0 Hz, 1H), 7.37 (s, 1H), 7.26 (d, *J* = 8.3 Hz, 2H), 7.23–7.16 (m, 1H), 4.53 (q, *J* = 7.1 Hz, 2H), 4.17 (q, *J* = 7.1 Hz, 2H), 2.41 (s, 3H), 1.43 (t, *J* = 7.1 Hz, 3H), 1.08 ppm (t, *J* = 7.1 Hz, 3H). ^{13}C NMR (100 MHz, $CDCl_3$, 25 °C) δ = 168.5, 167.4, 155.6, 144.5, 139.8, 138.7, 137.8, 135.6, 129.0, 128.8, 124.0, 123.6, 121.3, 118.5, 117.2, 115.5, 103.7, 62.4, 61.8, 21.4, 14.1, 13.7 ppm. IR: v = 3369, 2980, 1728, 1550, 1248, 1027, 747, 501 cm^{-1}; HRMS (TOF, ESI): *m/z* calcd for $C_{24}H_{22}N_2O_4$, [M + H]$^+$402.1579, found 402.1601.

Diethyl 4-phenyl-6*H*-azepino[4,3,2-*cd*]indole-2,3-dicarboxylate (4b) Yellow solid, mp: 150–152 °C, ^{1}H NMR (400 MHz, $CDCl_3$, 25 °C, TMS): δ = 9.04 (s, 1H), 7.76–7.72 (m, 2H), 7.69 (d, *J* = 9.0 Hz, 1H), 7.59 (d, *J* = 9.0 Hz, 1H), 7.46 (q, *J* = 5.7 Hz, 3H), 7.39 (s, 1H), 7.25 (dd, *J* = 5.2, 2.5 Hz, 1H), 4.53 (q, *J* = 7.1 Hz, 2H), 4.14 (q, *J* = 7.1 Hz, 2H), 1.43 (t, *J* = 7.1 Hz, 3H), 1.02 ppm (t, *J* = 7.1 Hz, 3H). ^{13}C NMR (100 MHz, $CDCl_3$, 25 °C) δ = 168.3, 167.3, 155.7, 144.5, 140.7, 140.0, 135.7, 128.9, 128.7, 128.2, 124.1, 123.6, 121.4, 118.7, 118.6, 117.4, 115.5, 103.9, 103.8, 62.4, 61.8, 14.1, 13.6 ppm. IR: ν = 3380, 2980, 1717, 1549, 1246, 1213, 1017, 737, 696, 597, 499 cm^{-1}; HRMS (TOF, ESI): m/z calcd for $C_{23}H_{20}N_2O_4$, [M + H]$^+$ 388.1423, found 388.1426.

Diethyl 4-(4-bromophenyl)-6*H*-azepino[4,3,2-*cd*]indole-2,3-dicarboxylate (4c) Yellow solid, mp: 172–174 °C, ^{1}H NMR (400 MHz, $CDCl_3$, 25 °C, TMS): δ = 8.96 (d, *J* = 11.2 Hz, 1H), 7.70 (d, *J* = 9.0 Hz, 1H), 7.66–7.58 (m, 5H), 7.39 (d, *J* = 1.9 Hz, 1H), 7.33–7.27 (m, 1H), 4.54 (q, *J* = 7.1 Hz, 2H), 4.18 (q, *J* = 7.1 Hz, 2H), 1.44 (t, *J* = 7.1 Hz, 3H), 1.10 ppm (t, *J* = 7.1 Hz, 3H). ^{13}C NMR (100 MHz, $CDCl_3$, 25 °C) δ = 168.04, 167.15, 154.42, 144.47, 140.18, 139.62, 135.68, 131.39, 130.55, 124.07, 123.65, 123.22, 121.07, 118.70, 117.51, 115.68, 103.87, 62.44, 61.91, 14.11, 13.67 ppm. IR: v = 3372, 2971, 1735, 1707, 1254, 1215, 1007, 746, 582, 446 cm^{-1}; HRMS (TOF, ESI): *m/z* calcd for $C_{23}H_{19}BrN_2O_4$, [M + H]$^+$ 466.0528, found 466.0540.

Diethyl 4-(4-(trifluoromethyl)phenyl)-6*H*-azepino[4,3,2-*cd*]indole-2,3-dicarboxylate (4d) Yellow solid, mp: 126–128 °C, ^{1}H NMR (400 MHz, $CDCl_3$, 25 °C, TMS): δ = 9.11 (s, 1H), 7.84 (d, *J* = 7.9 Hz, 2H), 7.71 (dd, *J* = 16.0, 8.5 Hz, 3H), 7.62 (d, *J* = 9.0 Hz, 1H), 7.37 (s, 1H), 7.26 (d, *J* = 2.9 Hz, 1H), 4.55 (q, *J* = 7.1 Hz, 2H), 4.16 (q, *J* = 7.1 Hz, 2H), 1.44 (t, *J* = 7.1 Hz, 3H), 1.04 ppm (t, *J* = 7.1 Hz, 3H). ^{13}C NMR (100 MHz, $CDCl_3$, 25 °C) δ = 167.8, 167.2, 154.3, 144.5, 144.3, 140.4, 135.8, 130.8, 130.5, 129.3, 125.6, 125.2, 125.1, 124.1, 123.8, 122.9, 121.0, 118.6, 117.7, 116.0, 103.8, 62.5, 62.0, 14.1, 13.5 ppm.

IR: ν = 3368, 2981, 1739, 1708, 1550, 1324, 1260, 748, 586, 449 cm^{-1}; HRMS (TOF, ESI): *m/z* calcd for $C_{24}H_{19}F_3N_2O_4$, $[M + H]^+$ 456.1296, found 456.1305.

Diethyl 4-(2-methoxyphenyl)-6*H*-azepino[4,3,2-*cd*]indole-2,3-dicarboxylate (4e) Yellow solid, mp: 125–127 °C, ^1H NMR (400 MHz, $CDCl_3$, 25 °C, TMS): δ = 9.19 (s, 1H), 7.73 (dd, *J* = 7.4, 1.4 Hz, 1H), 7.61 (d, *J* = 9.0 Hz, 1H), 7.56 (d, J = 9.0 Hz, 1H), 7.42–7.31 (m, 2H), 7.21–7.17 (m, 1H), 7.13 (t, *J* = 7.4 Hz, 1H), 6.87 (d, *J* = 8.2 Hz, 1H), 4.52 (q, *J* = 7.1 Hz, 2H), 4.15–4.07 (m, 2H), 3.66 (s, 3H), 1.42 (t, *J* = 7.1 Hz, 3H), 1.04 ppm (t, *J* = 7.1 Hz, 3H). ^{13}C NMR (100 MHz, $CDCl_3$, 25 °C) δ = 167.9, 167.3, 156.7, 154.0, 144.8, 140.4, 135.7, 131.3, 130.3, 130.1, 124.0, 123.6, 121.7, 121.0, 118.7, 118.6, 117.5, 115.4, 110.1, 103.8, 62.2, 61.2, 55.1, 14.1, 13.6 ppm. IR: ν = 3372, 2979, 1728, 1550, 1247, 1027, 751, 489 cm^{-1}; HRMS (TOF, ESI): *m/z* calcd for $C_{24}H_{22}N_2O_4$, $[M + H]^+$ 418.1528, found 418.1550.

Diethyl 4-(naphthalen-2-yl)-6*H*-azepino[4,3,2-*cd*]indole-2,3-dicarboxylate (4f) Yellow solid, mp: 199–201 °C, ^1H NMR (400 MHz, $CDCl_3$, 25 °C, TMS): δ = 8.95 (s, 1H), 8.21 (s, 1H), 7.98–7.86 (m, 4H), 7.72 (d, *J* = 9.0 Hz, 1H), 7.64 (d, *J* = 9.0 Hz, 1H), 7.56–7.48 (m, 2H), 7.45 (s, 1H), 7.30 (s, 1H), 4.56 (q, *J* = 7.2 Hz, 2H), 4.12 (q, *J* = 7.1 Hz, 2H), 1.45 (t, *J* = 7.1 Hz, 3H), 0.93 ppm (t, *J* = 7.1 Hz, 3H). ^{13}C NMR (100 MHz, $CDCl_3$, 25 °C) δ = 168.4, 167.3, 155.5, 144.5, 140.1, 138.0, 135.7, 133.4, 133.2, 128.5, 128.5, 127.9, 127.7, 126.6, 126.5, 126.3, 124.1, 123.6, 121.6, 118.7, 117.4, 115.5, 104.0, 62.4, 61.8, 14.1, 13.6 ppm. IR: ν = 3381, 2980, 1727, 1550, 1243, 1021, 750, 479 cm^{-1}; HRMS (TOF, ESI): *m/z* calcd for $C_{27}H_{22}N_2O_4$, $[M + H]^+$ 438.1579, found 438.1568.

Diethyl 4-(2,3-dichlorophenyl)-6*H*-azepino[4,3,2-*cd*]indole-2,3-dicarboxylate (4g) Yellow solid, mp: 172–174 °C, ^1H NMR (400 MHz, $CDCl_3$, 25 °C, TMS): δ = 9.18 (s, 1H), 7.67 (s, 2H), 7.52 (d, *J* = 7.9 Hz, 1H), 7.42 (d, *J* = 7.2 Hz, 1H), 7.33 (d, *J* = 7.8 Hz, 1H), 7.28 (dd, *J* = 9.3, 6.8 Hz, 2H), 4.56 (q, *J* = 7.1 Hz, 2H), 4.12 (dd, *J* = 13.7, 6.8 Hz, 2H), 1.45 (t, *J* = 7.1 Hz, 3H), 1.00 ppm (t, *J* = 7.1 Hz, 3H). ^{13}C NMR (100 MHz, $CDCl_3$, 25 °C) δ = 167.6, 166.2, 154.2, 144.7, 142.5, 141.7, 135.9, 132.8, 131.5, 130.1, 129.1, 127.2, 124.0, 123.9, 120.1, 118.9, 118.2, 116.0, 104.0, 62.4, 61.7, 29.7, 14.1, 13.4 ppm. IR: ν = 3379, 2981, 1724, 1549, 1412, 1246, 1031, 744, 515 cm^{-1}; HRMS (TOF, ESI): *m/z* calcd for $C_{23}H_{18}Cl_2N_2O_4$, $[M + H]^+$ 456.0643, found 456.0626.

Diethyl 4-(3,4,5-trimethoxyphenyl)-6*H*-azepino[4,3,2-*cd*]indole-2,3-dicarboxylate (4h) Yellow solid, mp: 200–202 °C, ^1H NMR (400 MHz, $CDCl_3$, 25 °C, TMS): δ = 8.89 (s, 1H), 7.74 (d, *J* = 9.0 Hz, 1H), 7.68 (d, *J* = 9.0 Hz, 1H), 7.46 (s, 1H), 7.36 (d, *J* = 2.4 Hz, 1H), 6.99 (s, 2H), 4.54 (q, *J* = 7.1 Hz, 2H), 4.18 (q, *J* = 7.1 Hz, 2H), 3.91 (d, *J* = 4.5 Hz, 9H), 1.45 (t, *J* = 7.2 Hz, 3H), 1.09 ppm (t, *J* = 7.1 Hz, 3H). ^{13}C NMR (100 MHz, $CDCl_3$, 25 °C) δ = 168.5, 167.1, 155.3, 153.2, 144.3, 139.8, 136.2, 135.7, 124.1, 123.5, 121.7, 118.8, 117.5, 115.4, 106.2, 104.0, 62.4, 61.9, 61.0, 56.2, 14.1, 13.7 ppm. IR: ν = 3351, 2979, 1730, 1587, 1416, 1240, 1126, 1022, 873, 746, 531, 455 cm^{-1}; HRMS (TOF, ESI): *m/z* calcd for $C_{26}H_{26}N_2O_7$, $[M + H]^+$ 478.1740, found 478.1749.

Diethyl 4-(6-bromobenzo[*d*][1,3]dioxol-5-yl)-6*H*-azepino[4,3,2-*cd*]indole-2,3-dicarboxylate (4i) Yellow solid, mp: 160–162 °C, ^1H NMR (400 MHz, $CDCl_3$, 25 °C, TMS): δ = 9.85 (s, 1H), 7.66 (q, *J* = 9.0 Hz, 2H), 7.35 (s, 1H), 7.28 (s, 1H), 7.04 (s, 1H), 7.00 (s, 1H), 6.00 (s, 2H), 4.55 (q, *J* = 7.0 Hz, 2H), 4.18 (q, *J* = 7.0 Hz, 2H), 1.45 (t, *J* = 7.1 Hz, 3H), 1.12 ppm (t, *J* = 7.1 Hz, 3H). ^{13}C NMR (100 MHz, $CDCl_3$, 25 °C) δ = 167.6, 166.5, 155.6, 148.4, 147.1, 144.5, 141.2, 136.1, 135.3, 124.0, 123.8, 120.6, 118.5, 118.0, 116.2, 113.4, 112.3, 110.9, 103.6, 101.9, 62.3, 61.7, 14.1, 13.7 ppm. IR: ν = 3377, 2979, 2900, 1726, 1549, 1479, 1238, 1037, 932, 876, 745, 444 cm^{-1}; HRMS (TOF, ESI): *m/z* calcd for $C_{24}H_{19}BrN_2O_6$, $[M + H]^+$ 510.0426, found 510.0409.

Diethyl 4-phenethyl-6*H*-azepino[4,3,2-*cd*]indole-2,3-dicarboxylate (4j) Yellow solid, mp: 172–174 °C, ^1H NMR (400 MHz, $CDCl_3$, 25 °C, TMS): δ = 8.86 (s, 1H), 7.68 (d, *J* = 9.0 Hz, 1H), 7.61 (d, *J*

= 9.0 Hz, 1H), 7.42 (s, 1H), 7.37–7.27 (m, 5H), 7.21 (d, *J* = 6.8 Hz, 1H), 4.51 (q, *J* = 7.1 Hz, 2H), 4.40 (dd, *J* = 14.3, 7.1 Hz, 2H), 3.52 (dd, *J* = 9.8, 6.6 Hz, 2H), 3.25 (dd, *J* = 9.7, 6.6 Hz, 2H), 1.42 (dt, *J* = 21.2, 7.1 Hz, 6H) ppm. ^{13}C NMR (100 MHz, $CDCl_3$, 25 °C) δ = 167.6, 167.5, 157.7, 144.6, 142.2, 139.8, 135.6, 128.6, 128.4, 125.9, 123.8, 123.3, 121.0, 119.0, 117.2, 114.8, 103.8, 62.1, 61.9, 39.0, 35.6, 14.2, 14.1 ppm. IR: v = 3369, 2982, 1721, 1555, 1242, 1019, 742, 508 cm^{-1}; HRMS (TOF, ESI): *m/z* calcd for $C_{25}H_{24}N_2O_4$, $[M + H]^+$ 416.1736, found 416.1763.

Diethyl 4-methyl-6*H*-azepino[4,3,2-*cd*]indole-2,3-dicarboxylate (4k) Yellow solid, mp: 164–166 °C, ^1H NMR (400 MHz, $CDCl_3$, 25 °C, TMS): δ = 9.18 (s, 1H), 7.62 (d, *J* = 4.9 Hz, 2H), 7.39 (s, 1H), 7.32 (s, 1H), 4.52 (q, *J* = 7.1 Hz, 2H), 4.43 (q, *J* = 7.1 Hz, 2H), 2.95 (s, 3H), 1.43 ppm (q, *J* = 7.4 Hz, 6H). ^{13}C NMR (100 MHz, $CDCl_3$, 25 °C) δ = 167.6, 167.5, 155.4, 144.6, 140.2, 135.7, 123.5, 120.6, 118.9, 117.1, 114.8, 103.5, 62.1, 61.8, 25.1, 14.1 ppm. IR: v = 3381, 2983, 1727, 1559, 1246, 1204, 1039, 742, 516 cm^{-1}; HRMS (TOF, ESI): *m/z* calcd for $C_{18}H_{18}N_2O_4$, $[M + H]^+$ 326.1266, found 326.1258.

Diethyl 4-butyl-6*H*-azepino[4,3,2-*cd*]indole-2,3-dicarboxylate (4l) Yellow oil, ^1H NMR (400 MHz, $CDCl_3$, 25 °C, TMS): δ = 9.05 (s, 1H), 7.65 (d, *J* = 9.0 Hz, 1H), 7.56 (d, *J* = 9.0 Hz, 1H), 7.38 (s, 1H), 7.29 (s, 1H), 4.50 (q, *J* = 7.1 Hz, 2H), 4.42 (q, *J* = 7.1 Hz, 2H), 3.24–3.13 (m, 2H), 1.90 (dd, *J* = 15.3, 7.6 Hz, 2H), 1.42 (q, *J* = 7.2 Hz, 6H), 1.04 ppm (t, *J* = 7.3 Hz, 3H). ^{13}C NMR (100 MHz, $CDCl_3$, 25 °C) δ = 167.9, 167.6, 158.7, 144.6, 139.7, 135.6, 123.7, 123.3, 121.1, 118.9, 117.0, 114.7, 103.7, 62.2, 61.8, 39.3, 23.2, 14.2, 14.2, 14.1 ppm. IR: v = 2924, 2961, 2865, 2730, 1725, 1630, 1595, 1414, 1378, 1310, 1124, 736, 553 cm^{-1}. HRMS (TOF, ESI): *m/z* calcd for $C_{20}H_{22}N_2O_4$, $[M + H]^+$ 354.1580, found 354.1581.

Diethyl 4-neopentyl-6*H*-azepino[4,3,2-*cd*]indole-2,3-dicarboxylate (4m) Yellow solid, mp: 110–112 °C, ^1H NMR (400 MHz, $CDCl_3$, 25 °C, TMS): δ = 9.16 (s, 1H), 7.67 (d, *J* = 9.0 Hz, 1H), 7.60 (d, *J* = 9.0 Hz, 1H), 7.37 (s, 1H), 7.31 (s, 1H), 4.49 (q, *J* = 7.1 Hz, 2H), 4.39 (q, *J* = 7.1 Hz, 2H), 3.24 (s, 2H), 1.42 (dt, *J* = 11.4, 7.2 Hz, 6H), 1.02 (s, 9H) ppm. ^{13}C NMR (100 MHz, $CDCl_3$, 25 °C) δ = 168.3, 167.6, 156.7, 144.1, 139.1, 135.4, 123.9, 123.2, 122.4, 118.7, 116.7, 114.8, 103.6, 62.1, 61.7, 47.9, 33.1, 29.9, 14.2, 14.1 ppm. IR: v = 3375, 2958, 1716, 1552, 1247, 1206, 1019, 747, 588, 521 cm^{-1}; HRMS (TOF, ESI): *m/z* calcd for $C_{22}H_{26}N_2O_4$, $[M + H]^+$ 382.1892, found 382.1923.

Diethyl 4-cyclopropyl-6*H*-azepino[4,3,2-*cd*]indole-2,3-dicarboxylate (4n) Yellow solid, mp: 130–132 °C, ^1H NMR (400 MHz, $CDCl_3$, 25 °C, TMS): δ = 8.79 (s, 1H), 7.63 (d, *J* = 9.0 Hz, 1H), 7.50 (d, *J* = 9.0 Hz, 1H), 7.26 (dd, *J* = 8.8, 2.4 Hz, 2H), 4.47 (dq, *J* = 24.1, 7.1 Hz, 4H), 2.60 (td, *J* = 8.1, 4.1 Hz, 1H), 1.42 (dd, *J* = 13.3, 7.0 Hz, 8H), 1.12–1.02 ppm (m, 2H). ^{13}C NMR (100 MHz, $CDCl_3$, 25 °C) δ = 168.0, 167.6, 158.6, 144.7, 138.9, 135.5, 123.6, 123.1, 121.4, 119.0, 116.6, 114.2, 103.5, 62.1, 61.9, 15.4, 14.2, 11.0 ppm. IR: v = 3381, 2983, 1726, 1556, 1418, 1248, 1181, 1023, 865, 744, 667, 533 cm^{-1}; HRMS (TOF, ESI): *m/z* calcd for $C_{20}H_{20}N_2O_4$, $[M + H]^+$ 352.1423, found 352.1432.

Dimethyl 4-(*p*-tolyl)-6*H*-azepino[4,3,2-*cd*]indole-2,3-dicarboxylate (4o) Yellow oil, ^1H NMR (400 MHz, DMSO-d_6, 25 °C): δ = 11.97 (s, 1H), 7.86 (d, *J* = 9.0 Hz, 1H), 7.60 (dd, *J* = 14.8, 8.4 Hz, 4H), 7.34 (d, *J* = 7.7 Hz, 2H), 7.20 (s, 1H), 4.00 (s, 3H), 3.70 (s, 3H), 2.39 ppm (s, 3H). ^{13}C NMR (100 MHz, $CDCl_3$, 25 °C) δ = 168.4, 167.4, 154.5, 144.3, 139.7, 138.9, 137.4, 136.2, 129.5, 128.9, 125.6, 123.8, 120.6, 117.9, 117.2, 116.5, 102.7, 53.7, 53.2, 21.3 ppm. IR (KBr) v = 3383, 2924, 1735, 1351, 1252, 1141, 1028, 749, 497 cm-1; HRMS (ESI, TOF) m/z: calcd for $C_{22}H_{19}N_2O_4^+$, $[M + H]^+$ 375.1339, found 375.1339.

2-Phenylpyridine (7a) Yellow oil, ^1H NMR (400 MHz, $CDCl_3$, 25 °C, TMS): δ = 8.70 (d, *J* = 4.7 Hz, 1H), 7.99 (d, *J* = 7.5 Hz, 2H), 7.78–7.68 (m, 2H), 7.48 (t, *J* = 7.4 Hz, 2H), 7.41 (t, *J* = 7.2 Hz, 1H), 7.25–7.18 ppm (m, 1H). ^{13}C NMR (100 MHz, $CDCl_3$, 25 °C) δ = 157.5, 149.7, 139.4, 136.8, 129.0, 128.8, 126.9, 122.1, 120.6 ppm.

2-(*p*-Tolyl)pyridine (7b) Yellow oil, ^1H NMR (400 MHz, $CDCl_3$, 25 °C, TMS): δ = 8.68 (d, *J* = 8.0 Hz, 1H), 7.89

(d, J = 8.0 Hz, 2H), 7.74–7.72 (m, 2H), 7.28 (t, J = 8.0 Hz, 2H), 7.22–7.19 (m, 1H), 2.41 ppm (s, 3H). ^{13}C NMR (100 MHz, $CDCl_3$, 25 °C) δ = 157.5, 149.6, 139.0, 136.7, 136.6, 129.5, 126.8, 121.8, 120.3, 21.3 ppm.

2-(4-Propylphenyl)pyridine (7c) Yellow oil, ^{1}H NMR (400 MHz, $CDCl_3$, 25 °C, TMS): δ = 8.67 (d, J = 4.6 Hz, 1H), 7.91 (d, J = 8.1 Hz, 2H), 7.76–7.66 (m, 2H), 7.28 (d, J = 8.1 Hz, 2H), 7.19 (dd, J = 8.9, 4.4 Hz, 1H), 2.64 (t, J = 7.6 Hz, 2H), 1.68 (dd, J = 15.0, 7.5 Hz, 2H), 0.96 (t, J = 7.3 Hz, 3H). ^{13}C NMR (100 MHz, $CDCl_3$, 25 °C) δ = 157.6, 149.6, 143.7, 136.9, 136.7, 128.9, 126.8, 121.8, 120.3, 37.8, 24.5, 13.8 ppm.

2-(4-Pentylphenyl)pyridine (7d) Yellow oil, ^{1}H NMR (400 MHz, $CDCl_3$, 25 °C, TMS): δ = 8.67 (d, J = 4.6 Hz, 1H), 7.90 (d, J = 8.1 Hz, 2H), 7.73–7.71 (m, 2H), 7.29 (d, J = 12.0 Hz, 2H), 7.20–7.18 (m, 1H), 2.66 (t, J = 4.0 Hz, 2H), 1.67–1.63 (m, 2H), 1.35–1.33 (m, 4H), 0.90 ppm (t, J = 4.0 Hz, 3H). ^{13}C NMR (100 MHz, $CDCl_3$, 25 °C) δ = 157.6, 149.6, 144.0, 136.8, 136.7, 128.9, 126.8, 121.8, 120.3, 35.7, 31.5, 31.1, 22.6, 14.1 ppm.

2-(4-(*tert*-Butyl)phenyl)pyridine (7e) Yellow oil, ^{1}H NMR (400 MHz, $CDCl_3$, 25 °C, TMS): δ = 8.69 (d, J = 4.1 Hz, 1H), 7.93 (d, J = 8.1 Hz, 2H), 7.74 (d, J = 7.0 Hz, 2H), 7.50 (d, J = 8.2 Hz, 2H), 7.22 (t, J = 5.0 Hz, 1H), 1.36 ppm (s, 9H). ^{13}C NMR (100 MHz, $CDCl_3$, 25 °C) δ =157.4, 152.3, 149.4, 136.9, 136.3, 126.7, 125.8, 121.9, 120.4, 34.7, 31.3 ppm.

2-(4-Cyclohexylphenyl)pyridine (7f) Yellow oil, ^{1}H NMR (400 MHz, $CDCl_3$, 25 °C, TMS): δ = 8.68 (d, J = 4.0 Hz, 1H), 7.91 (d, J = 8.0 Hz, 2H), 7.73–7.71 (m, 2H), 7.32 (d, J = 8.0 Hz, 2H), 7.21–7.18 (m, 1H), 2.58–2.53 (m, 1H), 1.92–1.84 (m, 4H), 1.48–1.39 (m, 4H), 1.29–1.25 ppm (m, 2H). ^{13}C NMR (100 MHz, $CDCl_3$, 25 °C) δ = 157.6, 149.6, 149.1, 137.0, 136.7, 127.3, 126.9, 121.8, 120.3, 44.4, 34.4, 26.9, 26.2 ppm.

2-(4-(Methylthio)phenyl)pyridine (7g) Yellow oil, ^{1}H NMR (400 MHz, $CDCl_3$, 25 °C, TMS): δ = 8.67 (d, J = 4.4 Hz, 1H), 7.93 (d, J = 8.5 Hz, 2H), 7.77–7.65 (m, 2H), 7.34 (d, J = 8.4 Hz, 2H), 7.24–7.17 (m, 1H), 2.53 ppm (s, 3H). ^{13}C NMR (100 MHz, $CDCl_3$, 25 °C) δ = 156.8, 149.7, 139.9, 136.8, 136.0, 128.8, 127.2, 126.4, 122.0, 120.1, 15.6 ppm.

2-(4-Methoxyphenyl)pyridine (7h) Yellow oil, ^{1}H NMR (400 MHz, $CDCl_3$, 25 °C, TMS): δ = 8.65 (d, J = 4.4 Hz, 1H), 7.95 (d, J = 8.6 Hz, 2H), 7.75–7.60 (m, 2H), 7.22–7.13 (m, 1H), 7.00 (d, J = 8.6 Hz, 2H), 3.86 (s, 3H) ppm. ^{13}C NMR (100 MHz, $CDCl_3$, 25 °C) δ = 160.5, 157.1, 149.5, 136.7, 132.0, 128.2, 121.4, 119.8, 114.1, 55.4 ppm.

2-(4-(Benzyloxy)phenyl)pyridine (7i) Yellow oil, ^{1}H NMR (400 MHz, $CDCl_3$, 25 °C, TMS): δ = 8.65 (d, J = 4.2 Hz, 1H), 7.95 (d, J = 8.6 Hz, 2H), 7.69 (dt, J = 15.8, 7.8 Hz, 2H), 7.46 (d, J = 7.2 Hz, 2H), 7.40 (t, J = 7.3 Hz, 2H), 7.36–7.30 (m, 1H), 7.20–7.13 (m, 1H), 7.07 (d, J = 8.6 Hz, 2H), 5.13 ppm (s, 2H). ^{13}C NMR (100 MHz, $CDCl_3$, 25 °C) δ = 159.7, 157.1, 149.6, 136.8, 136.7, 132.3, 128.6, 128.2, 128.0, 127.5, 121.5, 119.8, 115.1, 70.1 ppm.

2-([1,1'-Biphenyl]-4-yl)pyridine (7j) Yellow oil, ^{1}H NMR (400 MHz, $CDCl_3$, 25 °C, TMS): δ = 8.72 (d, J = 4.0 Hz, 1H), 8.09 (d, J = 8.0 Hz, 2H), 7.77–7.75 (m, 2H), 7.72 (d, J = 8.0 Hz, 2H), 7.66 (d, J = 8.0 Hz, 2H), 7.46 (t, J = 8.0 Hz, 2H), 7.38–7.35 (m, 1H), 7.25–7.22 ppm (m, 1H). ^{13}C NMR (100 MHz, $CDCl_3$, 25 °C) δ = 157.1, 149.8, 141.7, 140.6, 138.3, 136.8, 128.9, 127.6, 127.5, 127.3, 127.1, 122.2, 120.5 ppm.

2-(4-Fluorophenyl)pyridine (7k) Yellow oil, ^{1}H NMR (400 MHz, $CDCl_3$, 25 °C, TMS): δ = 8.70 (d, J = 3.0 Hz, 1H), 8.05–7.94 (m, 2H), 7.80 (d, J = 7.2 Hz, 1H), 7.72 (d, J = 7.8 Hz, 1H), 7.27 (s, 1H), 7.18 ppm (t, J = 8.5 Hz, 2H). ^{13}C NMR (100 MHz, $CDCl_3$, 25 °C) δ = 156.4, 149.8, 138.9, 137.9, 136.9, 128.7, 122.5, 120.3, 95.3 ppm.

2-(4-Chlorophenyl)pyridine (7l) Yellow oil, ^{1}H NMR (400 MHz, $CDCl_3$, 25 °C, TMS): δ = 8.70 (d, J = 3.0 Hz, 1H), 8.05–7.95 (m, 2H), 7.80 (d, J = 7.2 Hz, 1H), 7.72 (d, J = 7.8 Hz, 1H), 7.26–7.25 (m, 1H), 7.18 ppm (t, J = 8.5 Hz, 2H). ^{13}C NMR (100 MHz, $CDCl_3$, 25 °C) δ = 156.4, 149.8, 138.9, 137.9, 136.9, 128.7, 122.5, 120.3, 95.3 ppm.

2-(4-Iodophenyl)pyridine (7m) Yellow oil, ^{1}H NMR (400 MHz, $CDCl_3$, 25 °C, TMS): δ = 8.69 (d, J = 4.3 Hz, 1H), 7.81 (d, J = 8.5 Hz, 2H), 7.77–7.67 (m, 4H), 7.25 ppm (d, J = 8.8 Hz, 1H). ^{13}C NMR (100 MHz, $CDCl_3$, 25 °C) δ = 156.4, 149.8, 138.9, 137.9, 136.9, 128.7, 122.5, 120.3, 95.3 ppm.

2-(*m*-Tolyl)pyridine (7n) Yellow oil, ^{1}H NMR (400 MHz, $CDCl_3$, 25 °C, TMS): δ = 8.69 (d, J = 4.4 Hz, 1H), 7.84 (s, 1H), 7.74 (dd, J = 8.1, 6.9 Hz, 3H), 7.37 (t, J = 7.6 Hz, 1H), 7.29–7.18 (m, 2H), 2.44 ppm (s, 3H). ^{13}C NMR (100

MHz, $CDCl_3$, 25 °C) δ = 157.7, 149.6, 139.4, 138.4, 136.7, 129.7, 128.7, 127.7, 124.1, 122.0, 120.7, 21.5 ppm.

2-(Naphthalen-2-yl)pyridine (7o) Yellow oil, ^{1}H NMR (400 MHz, $CDCl_3$, 25 °C, TMS): δ = 8.75 (d, J = 4.0 Hz, 1H), 8.48 (s, 1H), 8.14 (d, J = 8.5 Hz, 1H), 7.94 (d, J = 7.7 Hz, 2H), 7.87 (d, J = 8.0 Hz, 2H), 7.78 (dd, J = 10.9, 4.4 Hz, 1H), 7.50 (dd, J = 6.0, 3.3 Hz, 2H), 7.25 ppm (t, J = 5.8 Hz, 1H). ^{13}C NMR (100 MHz, $CDCl_3$, 25 °C) δ = 157.4, 149.8, 136.8, 136.7, 133.6, 133.5, 128.7, 128.5, 127.7, 126.5, 126.4, 126.3, 124.6, 122.2, 120.8 ppm.

2-(3,5-Dimethylphenyl)pyridine (7p) Yellow oil, ^{1}H NMR (400 MHz, $CDCl_3$, 25 °C, TMS): δ = 8.69 (d, J = 4.4 Hz, 1H), 7.73 (td, J = 7.7, 1.5 Hz, 1H), 7.39 (d, J = 7.8 Hz, 1H), 7.26–7.20 (m, 2H), 7.17 (d, J = 7.7 Hz, 1H), 7.11 (d, J = 7.8 Hz, 1H), 2.36 (s, 3H), 2.32 ppm (s, 3H). ^{13}C NMR (100 MHz, $CDCl_3$, 25 °C) δ = 160.1, 149.2, 140.2, 136.0, 135.3, 132.5, 130.7, 130.3, 129.0, 124.1, 121.5, 20.9, 19.8 ppm.

2-(3,5-Dimethoxyphenyl)pyridine (7q) Yellow oil, ^{1}H NMR (400 MHz, $CDCl_3$, 25 °C, TMS): δ = 8.67 (d, J = 4.0 Hz, 1H), 7.73–7.67 (m, 3H), 7.51 (d, J = 8.0 Hz, 1H), 7.21–7.17 (m, 1H), 6.96 (d, J = 8.0 Hz, 1H), 4.00 (s, 3H), 3.94 ppm (s, 3H). ^{13}C NMR (100 MHz, $CDCl_3$, 25 °C) δ = 157.1, 150.0, 149.5, 149.3, 136.7, 132.3, 121.6, 120.0, 119.4, 111.1, 109.9, 56.0 ppm.

2-(2,3-Dihydrobenzo[*b*][1,4]dioxin-6-yl)pyridine (7r) Yellow oil, ^{1}H NMR (400 MHz, $CDCl_3$, 25 °C, TMS): δ = 8.64 (d, J = 8.0 Hz, 1H), 7.70 (t, J = 8.0 Hz, 1H), 7.63 (d, J = 8.0 Hz, 1H), 7.54–7.49 (m, 2H), 7.19–7.16 (m, 1H), 6.95 ppm (d, J = 8.0 Hz, 1H), 4.30 ppm (s, 4H). ^{13}C NMR (100 MHz, $CDCl_3$, 25 °C) δ = 156.8, 149.5, 144.6, 143.8, 136.7, 133.0, 121.6, 120.1, 119.9, 117.5, 115.9, 64.6, 64.4 ppm.

2-(Benzo[*d*][1,3]dioxol-5-yl)pyridine (7s) Yellow oil, ^{1}H NMR (400 MHz, $CDCl_3$, 25 °C, TMS): δ = 8.64 (d, J = 4.2 Hz, 1H), 7.71 (td, J = 7.8, 1.7 Hz, 1H), 7.63 (d, J = 8.0 Hz, 1H), 7.53–7.47 (m, 2H), 7.18 (dd, J = 6.4, 5.0 Hz, 1H), 6.90 (d, J = 8.1 Hz, 1H), 6.02 ppm (s, 2H). ^{13}C NMR (100 MHz, $CDCl_3$, 25 °C) δ = 156.9, 149.5, 148.5, 148.3, 136.8, 133.9, 121.7, 120.9, 120.0, 108.5, 107.4, 101.3 ppm.

2-(3,4,5-Trimethoxyphenyl)pyridine (7t) Yellow oil, ^{1}H NMR (400 MHz, $CDCl_3$, 25 °C, TMS): δ = 8.67 (d, J = 4.0 Hz, 1H), 7.75 (t, J = 8.0 Hz, 1H), 7.69 (d, J = 8.0 Hz, 1H), 7.24–7.22 (m, 3H), 3.97 (s, 6H), 3.91 ppm (s, 3H). ^{13}C NMR (100 MHz, $CDCl_3$, 25 °C) δ = 157.1, 153.5, 149.5, 139.1, 136.8, 135.0, 122.0, 120.4, 104.2, 61.0, 56.3 ppm.

2-(Benzofuran-2-yl)pyridine (7u) Yellow oil, ^{1}H NMR (400 MHz, $CDCl_3$, 25 °C, TMS): δ = 8.69 (d, J = 4.6 Hz, 1H), 7.91 (d, J = 7.9 Hz, 1H), 7.80 (dd, J = 7.7, 1.5 Hz, 1H), 7.66 (d, J = 7.6 Hz, 1H), 7.57 (d, J = 8.2 Hz, 1H), 7.44 (s, 1H), 7.37 – 7.31 (m, 1H), 7.26 ppm (dd, J = 8.7, 6.1 Hz, 2H). ^{13}C NMR (100 MHz, $CDCl_3$, 25 °C) δ = 155.3, 155.0, 149.8, 149.2, 136.9, 128.8, 128.3, 125.2, 123.9, 123.3, 123.2, 122.9, 121.7, 119.9, 113.1, 112.5, 111.5, 104.9, 100.0 ppm.

2-(2,3-Dihydrobenzofuran-2-yl)pyridine (7v) Yellow oil, ^{1}H NMR (400 MHz, $CDCl_3$, 25 °C, TMS): δ = 8.63 (d, J = 4.1 Hz, 1H), 7.89 (s, 1H), 7.76–7.67 (m, 2H), 7.64 (d, J = 8.0 Hz, 1H), 7.19–7.12 (m, 1H), 6.86 (d, J = 8.3 Hz, 1H), 4.63 (t, J = 8.7 Hz, 2H), 3.27 ppm (t, J = 8.7 Hz, 2H). ^{13}C NMR (100 MHz, $CDCl_3$, 25 °C) δ = 161.1, 157.5, 149.5, 136.7, 132.2, 127.8, 127.2, 123.8, 121.3, 119.9, 109.4, 71.7, 29.6 ppm.

2-(9*H*-Fluoren-2-yl)pyridine (7w) Yellow oil, ^{1}H NMR (400 MHz, $CDCl_3$, 25 °C, TMS): δ = 8.71 (d, J = 4.3 Hz, 1H), 8.22 (s, 1H), 8.00 (d, J = 7.9 Hz, 1H), 7.85 (dd, J = 16.0, 7.7 Hz, 2H), 7.77 (dt, J = 7.1, 4.7 Hz, 2H), 7.56 (d, J = 7.3 Hz, 1H), 7.39 (t, J = 7.3 Hz, 1H), 7.32 (t, J = 7.3 Hz, 1H), 7.22 (dd, J = 8.4, 3.3 Hz, 1H), 3.98 (s, 2H) ppm. ^{13}C NMR (100 MHz, $CDCl_3$, 25 °C) δ = 157.7, 149.7, 143.9, 143.9, 142.6, 141.3, 138.0, 136.8, 127.0, 126.9, 125.8, 125.1, 123.6, 121.9, 120.6, 120.2, 120.1, 37.0 ppm.

3-Phenyl-*N*-(*p*-tolyl)naphthalen-1-amine (**10a**) White solid, ^{1}H NMR (400 MHz, $CDCl_3$, 25 °C, TMS): δ = 7.99 (d, J = 8.2 Hz, 1H), 7.89 (d, J = 8.0 Hz, 1H), 7.70 (s, 1H), 7.65 (d, J = 7.6 Hz, 2H), 7.57 (s, 1H), 7.53–7.39 (m, 4H), 7.34 (t, J = 7.3 Hz, 1H), 7.10 (d, J = 8.1 Hz, 2H), 7.01 (d, J = 8.2 Hz, 2H), 2.31 ppm (s, 3H).^{13}C NMR (100 MHz, $CDCl_3$, 25 °C) δ = 154.8, 142.7, 141.2, 138.5, 137.1, 135.2, 129.4, 129.0, 127.9, 127.3, 126.9, 125.3, 125.0, 122.8, 122.4, 117.3, 115.1, 108.7, 21.3 ppm.

***N*-(4-Methoxyphenyl)-3-phenylnaphthalen-1-amine (10b)** Light yellow oil, ^{1}H NMR (400 MHz, DMSO-d$_6$, 25 °C): δ = 8.28 (d, J = 8.1 Hz, 1H), 8.12 (s, 1H), 7.92 (d, J = 7.6 Hz, 1H), 7.64 (d, J = 6.9 Hz, 3H), 7.54– .42 (m, 4H), 7.34 (dd, J = 15.1, 7.7 Hz, 2H), 7.21 (d, J = 8.8 Hz, 2H), 6.94 (d, J = 8.8 Hz, 2H), 3.74 ppm (s, 3H). ^{13}C NMR (100 MHz, DMSO-d$_6$, 25 °C) δ = 154.8, 142.7, 141.2, 138.5, 137.1, 135.2, 129.4, 129.0, 127.9, 127.3, 126.9, 125.3, 125.0, 122.8, 122.4, 117.3, 115.1, 108.7, 55.7 ppm.
***N*-(4-(*tert*-Butyl)phenyl)-3-phenylnaphthalen-1-amine (10c)** White solid, ^{1}H NMR (400 MHz, $CDCl_3$, 25 °C, TMS): δ = 7.99 (d, J = 8.2 Hz, 1H), 7.88 (d, J = 7.9 Hz, 1H), 7.70 (s, 1H), 7.68–7.61 (m, 3H), 7.52–7.40 (m, 4H), 7.35 (d, J = 7.4 Hz, 1H), 7.30 (d, J = 8.5 Hz, 2H), 7.03 (d, J = 8.5 Hz, 2H), 1.32 ppm (s, 9H). ^{13}C NMR (100 MHz, $CDCl_3$, 25 °C) δ = 143.9, 141.6, 141.3, 139.8, 139.0, 135.0, 128.9, 128.8, 127.5, 127.4, 126.6, 126.3, 125.6, 121.4, 120.1, 117.8, 113.8, 34.2, 31.6, 29.8 ppm.
***N*-(4-Bromophenyl)-3-phenylnaphthalen-1-amine (10d)** Light yellow solid, ^{1}H NMR (400 MHz, DMSO-d$_6$, 25 °C): δ = 8.49 (s, 1H), 8.13 (d, J = 8.2 Hz, 1H), 8.00 (d, J = 7.9 Hz, 1H), 7.88 (s, 1H), 7.74 (d, J = 7.4 Hz, 2H), 7.62 (s, 1H), 7.58–7.46 (m, 4H), 7.42–7.34 (m, 3H), 7.06 ppm(d, J = 8.7 Hz, 2H). ^{13}C NMR (100 MHz, DMSO-d$_6$, 25 °C) δ = 145.0, 140.6, 139.8, 138.3, 135.3, 132.3, 129.5, 129.2, 128.1, 127.4, 127.2, 126.9, 125.9, 123.1, 120.3, 119.1, 114.4, 110.7 ppm.
***N*-(3,4-Dimethoxyphenyl)-3-phenylnaphthalen-1-amine (10e)** Yellow oil, ^{1}H NMR (400 MHz, $CDCl_3$, 25 °C, TMS) δ = 8.00 (d, J = 8.2 Hz, 1H), 7.89 (d, J = 8.0 Hz, 1H), 7.68–7.61 (m, 3H), 7.54–7.39 (m, 5H), 7.34 (t, J = 7.3 Hz, 1H), 6.83 (d, J = 8.5 Hz, 1H), 6.79 (d, J = 2.2 Hz, 1H), 6.68 (dd, J = 8.5, 2.3 Hz, 1H), 3.88 (s, 3H), 3.82 ppm (s, 3H). ^{13}C NMR (100 MHz, $CDCl_3$) δ = 149.8, 141.3, 141.1, 139.0, 137.3, 134.9, 129.0, 128.8, 127.4, 127.3, 126.5, 125.5, 125.3, 121.0, 119.1, 112.4, 112.1, 111.7, 105.3, 56.3, 55.9 ppm.
***N*-(Naphthalen-1-yl)-3-phenylnaphthalen-1-amine (10f)** Light yellow oil, ^{1}H NMR (400 MHz, DMSO-d$_6$, 25 °C): δ = 8.47 (s, 1H), 8.22 (d, J = 8.3 Hz, 2H), 8.01 (d, J = 8.1 Hz, 1H), 7.94 (d, J = 8.0 Hz, 1H), 7.82 (s, 1H), 7.60–7.45 (m, 8H), 7.41 (dd, J = 15.0, 7.4 Hz, 3H), 7.36–7.32 (m, 1H), 7.11 (s, 1H), 6.97 ppm (d, J = 7.4 Hz, 1H). ^{13}C NMR (100 MHz, DMSO-d$_6$, 25 °C) δ = 142.5, 141.4, 140.8, 138.4, 135.2, 134.8, 129.4, 129.2, 128.7, 127.9, 127.4, 127.1, 127.0, 126.8, 126.6, 126.2, 125.7, 125.7, 123.5, 123.4, 122.3, 119.3, 116.4, 114.1 ppm.
***N*-((3-Phenylnaphthalen-1-yl)methyl)aniline (10g)** Yellow oil, ^{1}H NMR (400 MHz, DMSO-d$_6$, 25 °C): δ = 8.28 (d, J = 7.9 Hz, 1H), 7.84 (d, J = 7.6 Hz, 1H), 7.59 (d, J = 7.5 Hz, 2H), 7.45 (dq, J = 15.1, 7.5 Hz, 6H), 7.34 (dd, J = 15.7, 8.3 Hz, 4H), 7.22 (d, J = 7.2 Hz, 1H), 7.11 (t, J = 5.6 Hz, 1H), 6.65 (s, 1H), 4.62 ppm (d, J = 5.5 Hz, 2H). ^{13}C NMR (100 MHz, DMSO-d$_6$, 25 °C) δ = 144.6, 141.6, 140.6, 138.7, 134.9, 129.3, 129.0, 128.8, 127.7, 127.5, 127.2, 127.1, 126.6, 124.7, 123.0, 122.0, 113.9, 103.3, 46.9 ppm.
***N*-Butyl-3-phenylnaphthalen-1-amine (10h)** Light yellow solid, ^{1}H NMR (400 MHz, DMSO-d$_6$, 25 °C): δ = 8.20 (d, J = 8.3 Hz, 1H), 7.83 (d, J = 7.9 Hz, 1H), 7.76 (d, J = 7.5 Hz, 2H), 7.51–7.43 (m, 3H), 7.42–7.33 (m, 3H), 6.73 (s, 1H), 6.19 (t, J = 4.8 Hz, 1H), 3.30 (dd, J = 12.4, 6.6 Hz, 2H), 1.78–1.66 (m, 2H), 1.47 (dd, J = 14.8, 7.4 Hz, 2H), 0.96 ppm (t, J = 7.3 Hz, 3H). ^{13}C NMR (100 MHz, DMSO-d$_6$, 25 °C) δ = 145.2, 141.9, 139.0, 134.9, 129.3, 128.9, 127.7, 127.4, 126.5, 124.5, 122.9, 122.1, 113.6, 102.2, 43.4, 30.8, 20.6, 14.4 ppm.
***N*-(Furan-3-ylmethyl)-3-phenylnaphthalen-1-amine (10i)** Yellow oil, ^{1}H NMR (400 MHz, DMSO-d$_6$, 25 °C): δ = 8.22 (d, J = 8.2 Hz, 1H), 7.85 (d, J = 7.8 Hz, 1H), 7.73 (d, J = 7.5 Hz, 2H), 7.59 (s, 1H), 7.52 – 7.34 (m, 7H), 6.90 (d, J = 7.0 Hz, 2H), 6.40 (d, J = 2.1 Hz, 2H), 4.60 ppm (d, J = 5.5 Hz, 2H). ^{13}C NMR (100 MHz, DMSO-d$_6$, 25 °C) δ = 153.7, 144.4, 142.4, 141.7, 138.7, 134.8, 129.3, 129.0, 127.8, 127.3, 126.6, 124.8, 123.0, 122.0, 114.3, 110.9, 107.5, 103.2 ppm.

4-(3-Phenylnaphthalen-1-yl)morpholine (10j) Yellow oil, ^{1}H NMR (400 MHz, DMSO-d_6, 25 °C): δ = 8.18–8.12 (m, 1H), 8.00–7.95 (m, 1H), 7.91 (s, 1H), 7.81 (d, J = 7.5 Hz, 2H), 7.52 (dd, J = 12.4, 5.4 Hz, 4H), 7.44–7.36 (m, 2H), 3.92–3.84 (m, 4H), 3.11 ppm (s, 4H). ^{13}C NMR (100 MHz, DMSO-d_6, 25 °C) δ = 150.3, 140.8, 138.2, 135.1, 129.4, 129.3, 128.0, 127.7, 127.5, 126.8, 126.1, 123.7, 121.3, 114.4, 67.1, 53.6 ppm.

8-Chloro-3-phenyl-*N*-(*p*-tolyl)naphthalen-1-amine (10k) Yellow oil, ^{1}H NMR (400 MHz, DMSO-d_6, 25 °C): δ = 8.34 (s, 1H), 8.29 (s, 1H), 7.97 (d, J = 8.8 Hz, 1H), 7.73 (s, 1H), 7.65 (d, J = 7.6 Hz, 2H), 7.56–7.50 (m, 2H), 7.46 (t, J = 7.6 Hz, 2H), 7.36 (t, J = 7.3 Hz, 1H), 7.16 – 7.08 (m, 4H), 2.25 ppm (s, 3H). ^{13}C NMR (100 MHz, DMSO-d_6, 25 °C) δ = 141.6, 141.0, 140.7, 139.0, 133.7, 131.2, 130.3, 130.2, 130.2, 129.4, 128.1, 127.4, 127.3, 126.2, 122.0, 119.4, 118.0, 111.4, 20.8 ppm.

6-Fluoro-3-phenyl-*N*-(*p*-tolyl)naphthalen-1-amine (10l) Light yellow solid, ^{1}H NMR (400 MHz, DMSO-d_6, 25 °C): δ = 8.35–8.27 (m, 2H), 7.72–7.67 (m, 2H), 7.65 (d, J = 7.6 Hz, 2H), 7.46 (dd, J = 13.0, 5.0 Hz, 3H), 7.40–7.34 (m, 2H), 7.12 (t, J = 5.1 Hz, 4H), 2.25 ppm (s, 3H). ^{13}C NMR (100 MHz, DMSO-d_6, 25 °C) δ = 162.3, 159.8, 141.9, 141.7, 140.8, 139.8, 136.5, 136.4, 130.2, 130.1, 129.4, 128.1, 127.3, 126.2, 126.1, 122.8, 119.6, 117.6, 115.2, 115.0, 111.9, 111.7, 110.1, 20.8 ppm.

3-(4-Fluorophenyl)-*N*-(*p*-tolyl)naphthalen-1-amine (10m) Yellow oil, ^{1}H NMR (400 MHz, DMSO-d_6, 25 °C): δ = 8.35–8.27 (m, 2H), 7.72–7.67 (m, 2H), 7.65 (d, J = 7.6 Hz, 2H), 7.46 (dd, J = 13.0, 5.0 Hz, 3H), 7.40–7.34 (m, 2H), 7.12 (t, J = 5.1 Hz, 4H), 2.25 ppm (s, 3H). ^{13}C NMR (100 MHz, DMSO-d_6, 25 °C) δ = 162.3, 159.8, 141.9, 141.7, 140.8, 139.8, 136.5, 136.4, 130.2, 130.1, 129.4, 128.1, 127.3, 126.2, 126.1, 122.8, 119.6, 117.6, 115.2, 115.0, 111.9, 111.7, 110.1, 20.8 ppm.

3-Heptyl-*N*-(*p*-tolyl)naphthalen-1-amine (10n) Yellow oil, ^{1}H NMR (400 MHz, $CDCl_3$, 25 °C, TMS): δ = 7.94 (d, J = 8.3 Hz, 1H), 7.77 (d, J = 8.0 Hz, 1H), 7.45 (t, J = 7.4 Hz, 1H), 7.41 – 7.36 (m, 1H), 7.30 (s, 1H), 7.17 (s, 1H), 7.08 (d, J = 8.1 Hz, 2H), 6.93 (d, J = 8.2 Hz, 2H), 2.67 (t, J = 7.7 Hz, 2H), 2.31 (s, 3H), 1.64 (dd, J = 14.7, 7.2 Hz, 2H), 1.39 – 1.22 (m, 8H), 0.88 ppm (t, J = 6.4 Hz, 3H). ^{13}C NMR (100 MHz, $CDCl_3$, 25 °C) δ = 142.1, 141.0, 139.2, 134.9, 130.2, 129.9, 128.1, 126.1, 125.7, 124.7, 121.4, 120.8, 118.2, 116.0, 36.3, 31.8, 31.3, 29.7, 29.1, 22.7, 20.7, 14.1 ppm.

3-(Cyclohex-1-en-1-yl)-*N*-(*p*-tolyl)naphthalen-1-amine (10o) Yellow oil, ^{1}H NMR (400 MHz, $CDCl_3$, 25 °C, TMS): δ = 7.93 (d, J = 8.3 Hz, 1H), 7.81 (d, J = 8.1 Hz, 1H), 7.49–7.35 (m, 4H), 7.08 (d, J = 8.1 Hz, 2H), 6.94 (d, J = 8.3 Hz, 2H), 6.19 (s, 1H), 2.49 (d, J = 1.6 Hz, 2H), 2.31 (s, 3H), 2.25 – 2.17 (m, 2H), 1.80 (dd, J = 7.6, 3.9 Hz, 2H), 1.72 – 1.63 ppm (m, 2H). ^{13}C NMR (100 MHz, $CDCl_3$, 25 °C) δ 141.1, 139.2, 138.0, 135.3, 133.8, 129.0, 128.9, 127.7, 125.4, 125.2, 124.4, 124.0, 120.4, 116.9, 116.9, 111.7, 28.7, 26.3, 25.0, 22.1, 21.2, 19.6 ppm.

3-Phenylisoquinoline (13a) White solid, mp: 94–96 °C ^{1}H NMR (400 MHz, $CDCl_3$, 25 °C, TMS): δ = 9.34 (s, 1H), 8.12 (d, J = 7.3 Hz, 2H), 8.06 (s, 1H), 7.98 (d, J = 8.1 Hz, 1H), 7.86 (d, J = 8.2 Hz, 1H), 7.68 (dd, J = 11.1, 4.0 Hz, 1H), 7.57 (dd, J = 11.1, 4.0 Hz, 1H), 7.51 (t, J = 7.5 Hz, 2H), 7.42 ppm (t, J = 7.3 Hz, 1H). ^{13}C NMR (100 MHz, $CDCl_3$, 25 °C) δ = 152.5, 151.4, 139.7, 136.7, 130.6, 128.9, 128.6, 127.9, 127.6, 127.14, 127.11, 127.0, 116.6ppm.

References

[1] Xi, L.; Zhang, R.; Liang, S.; Chen, S.; Yu, X. *Org. Lett.* **2014**, *16*, 5269−5271.

[2] Guo, B.; Hua, R. *Tetrahedron* **2016**, *72*, 4608−4615.

[3] Ju, J,; Hua, R. *Curr. Org. Synth.* **2013**, *10*, 328-332.

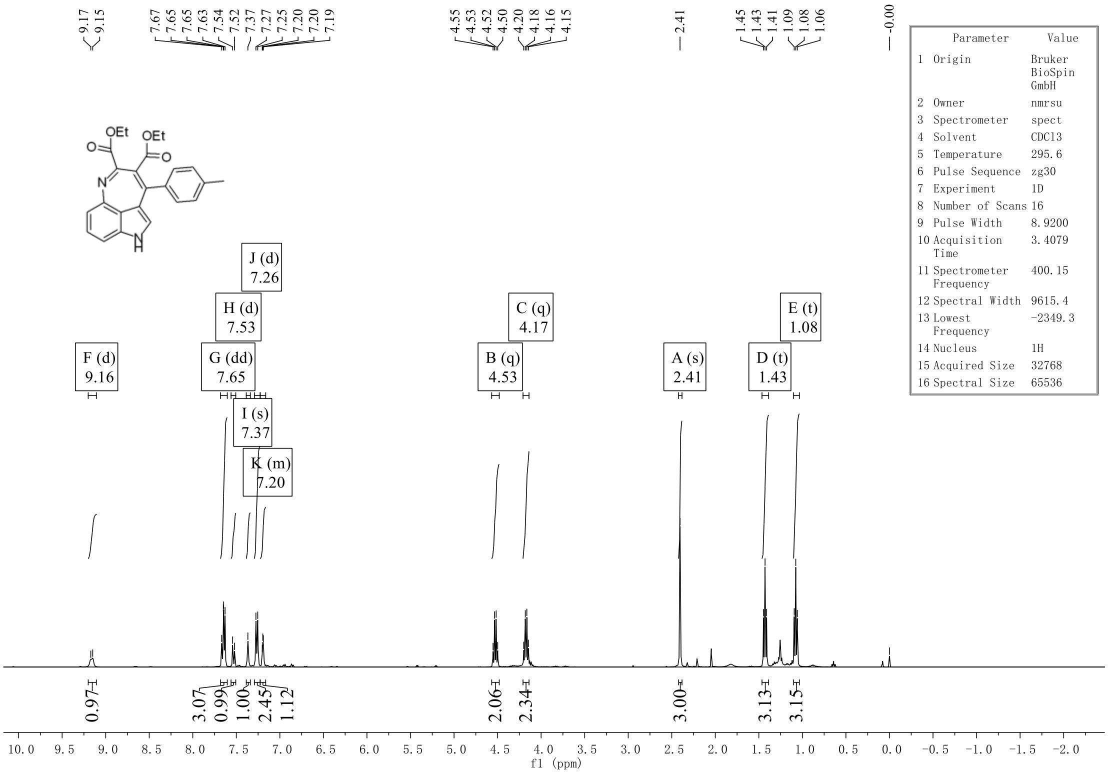

Parameter	Value
1 Origin	Bruker BioSpin GmbH
2 Owner	nmrsu
3 Spectrometer	spect
4 Solvent	CDCl3
5 Temperature	295.6
6 Pulse Sequence	zg30
7 Experiment	1D
8 Number of Scans	16
9 Pulse Width	8.9200
10 Acquisition Time	3.4079
11 Spectrometer Frequency	400.15
12 Spectral Width	9615.4
13 Lowest Frequency	-2349.3
14 Nucleus	1H
15 Acquired Size	32768
16 Spectral Size	65536

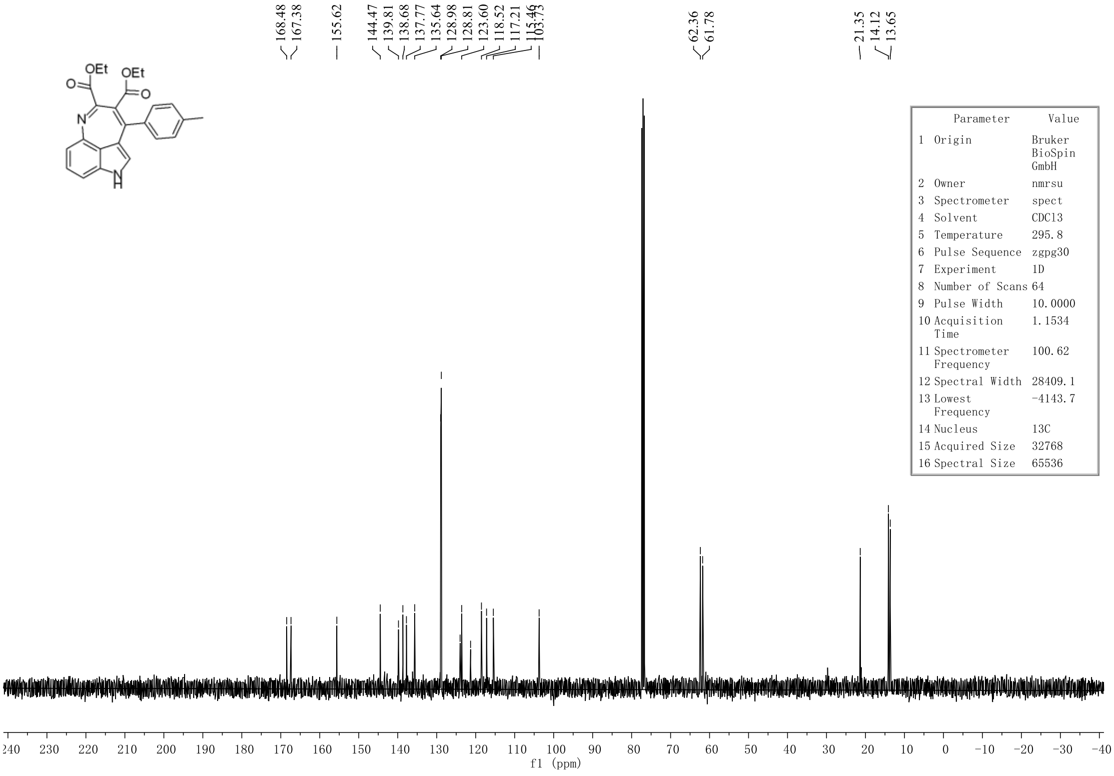

Parameter	Value
1 Origin	Bruker BioSpin GmbH
2 Owner	nmrsu
3 Spectrometer	spect
4 Solvent	CDCl3
5 Temperature	295.8
6 Pulse Sequence	zgpg30
7 Experiment	1D
8 Number of Scans	64
9 Pulse Width	10.0000
10 Acquisition Time	1.1534
11 Spectrometer Frequency	100.62
12 Spectral Width	28409.1
13 Lowest Frequency	-4143.7
14 Nucleus	13C
15 Acquired Size	32768
16 Spectral Size	65536

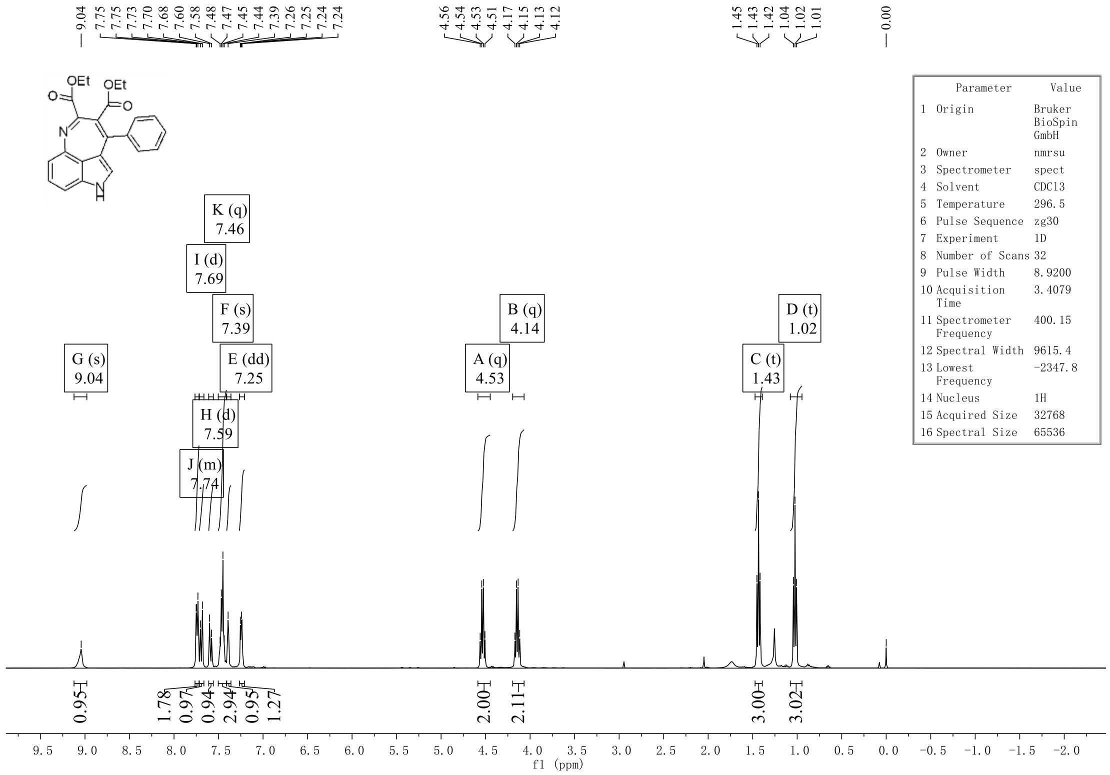
9.04
7.75 7.75 7.73 7.70 7.68 7.60 7.58 7.48 7.47 7.45 7.44 7.39 7.26 7.25 7.24 7.24
4.56 4.54 4.53 4.51 4.17 4.15 4.13 4.12
1.45 1.43 1.42 1.04 1.02 1.01
0.00
OEt
OEt
N
H
K (q) 7.46
I (d) 7.69
F (s) 7.39
B (q) 4.14
D (t) 1.02
G (s) 9.04
E (dd) 7.25
A (q) 4.53
C (t) 1.43
H (d) 7.59
J (m) 7.74
0.95 1.78 0.97 0.94 2.94 0.95 1.27 2.00 2.11 3.00 3.02
f1 (ppm)
Parameter Value
1 Origin Bruker BioSpin GmbH
2 Owner nmrsu
3 Spectrometer spect
4 Solvent CDCl3
5 Temperature 296.5
6 Pulse Sequence zg30
7 Experiment 1D
8 Number of Scans 32
9 Pulse Width 8.9200
10 Acquisition Time 3.4079
11 Spectrometer Frequency 400.15
12 Spectral Width 9615.4
13 Lowest Frequency -2347.8
14 Nucleus 1H
15 Acquired Size 32768
16 Spectral Size 65536

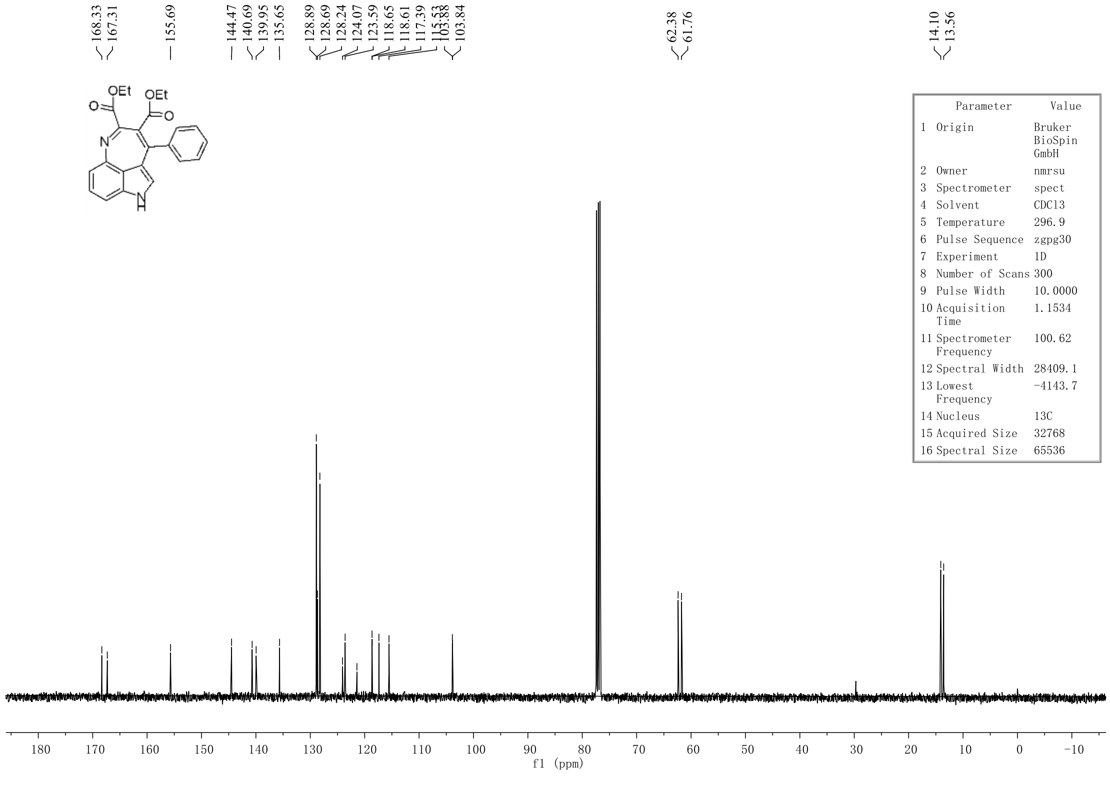
168.33 167.31
155.69
144.47 140.69 139.95 135.65
128.89 128.69 128.24 124.07 123.59 118.65 118.61 117.39 115.53 103.88 103.84
62.38 61.76
14.10 13.56
OEt
OEt
N
H
f1 (ppm)
Parameter Value
1 Origin Bruker BioSpin GmbH
2 Owner nmrsu
3 Spectrometer spect
4 Solvent CDCl3
5 Temperature 296.9
6 Pulse Sequence zgpg30
7 Experiment 1D
8 Number of Scans 300
9 Pulse Width 10.0000
10 Acquisition Time 1.1534
11 Spectrometer Frequency 100.62
12 Spectral Width 28409.1
13 Lowest Frequency -4143.7
14 Nucleus 13C
15 Acquired Size 32768
16 Spectral Size 65536

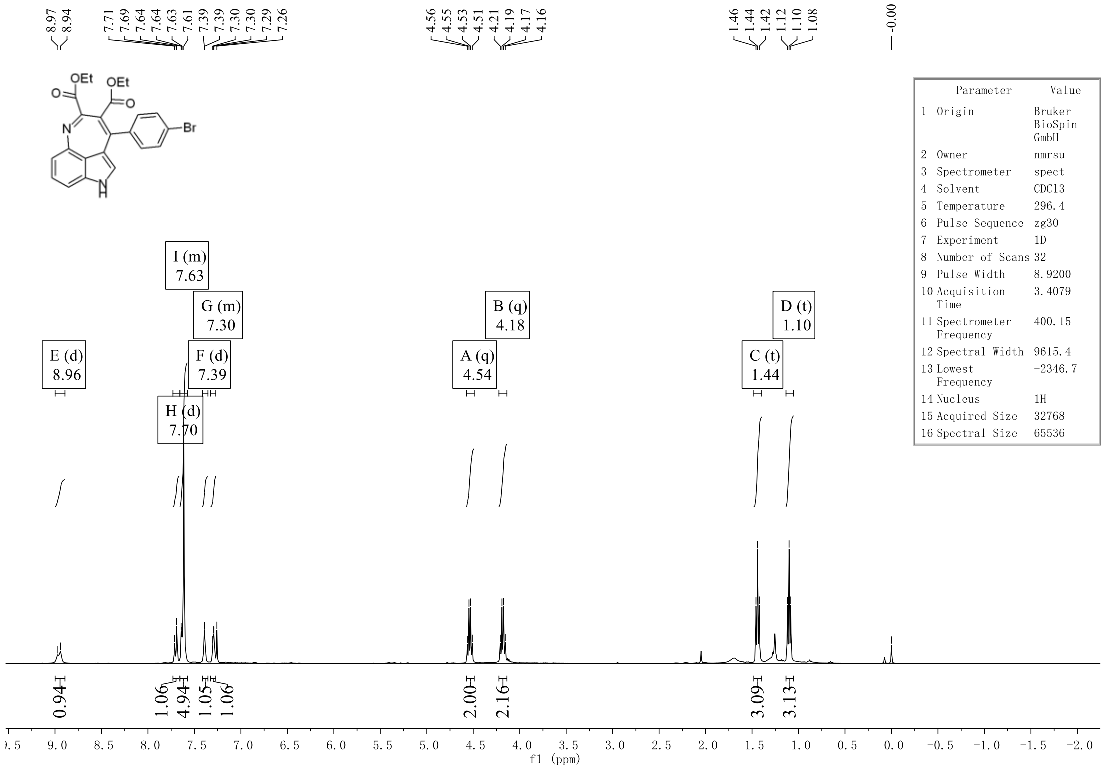
8.97
8.94
7.71
7.69
7.64
7.64
7.63
7.61
7.39
7.39
7.30
7.30
7.29
7.26
4.56
4.55
4.53
4.51
4.21
4.19
4.17
4.16
1.46
1.44
1.42
1.12
1.10
1.08
-0.00
OEt
OEt
O
O
N
Br
NH
I (m) 7.63
G (m) 7.30
B (q) 4.18
D (t) 1.10
E (d) 8.96
F (d) 7.39
A (q) 4.54
C (t) 1.44
H (d) 7.70
0.94
1.06
4.94
1.05
1.06
2.00
2.16
3.09
3.13
f1 (ppm)
Parameter Value
1 Origin Bruker BioSpin GmbH
2 Owner nmrsu
3 Spectrometer spect
4 Solvent CDCl3
5 Temperature 296.4
6 Pulse Sequence zg30
7 Experiment 1D
8 Number of Scans 32
9 Pulse Width 8.9200
10 Acquisition Time 3.4079
11 Spectrometer Frequency 400.15
12 Spectral Width 9615.4
13 Lowest Frequency -2346.7
14 Nucleus 1H
15 Acquired Size 32768
16 Spectral Size 65536

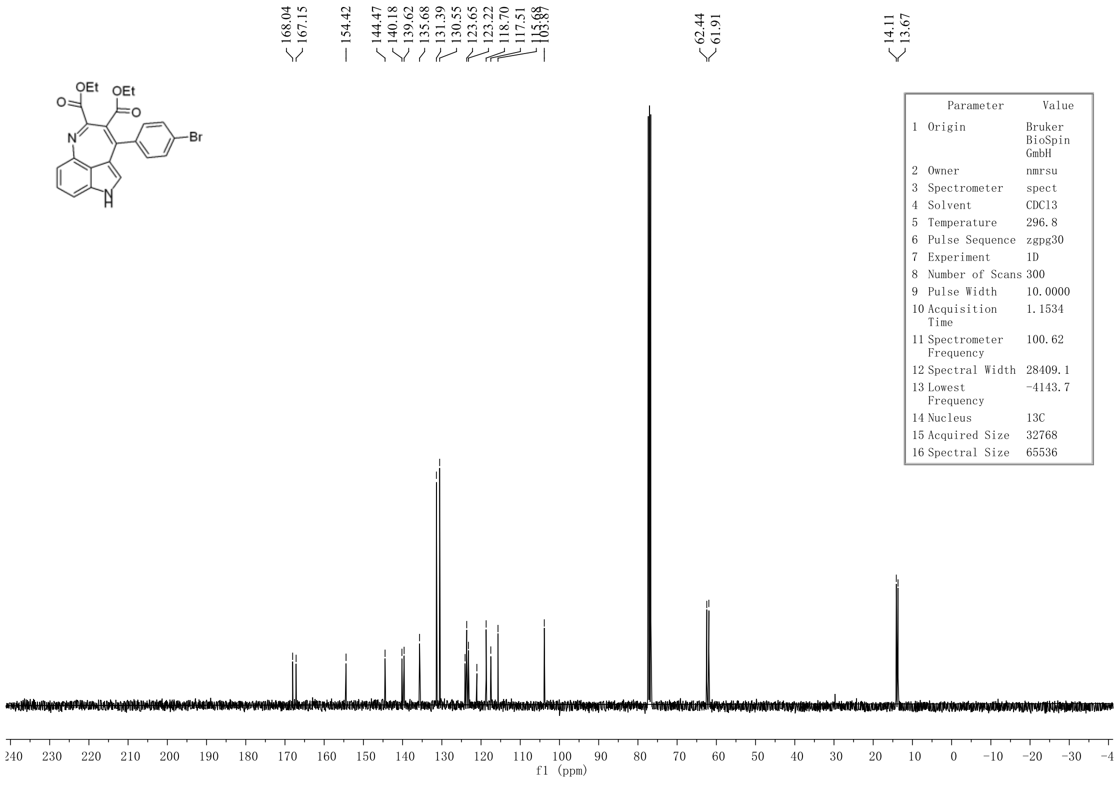
168.04
167.15
154.42
144.47
140.18
139.62
135.68
131.39
130.55
123.65
123.22
118.70
117.51
115.68
103.87
62.44
61.91
14.11
13.67
OEt
OEt
O
O
N
Br
NH
f1 (ppm)
Parameter Value
1 Origin Bruker BioSpin GmbH
2 Owner nmrsu
3 Spectrometer spect
4 Solvent CDCl3
5 Temperature 296.8
6 Pulse Sequence zgpg30
7 Experiment 1D
8 Number of Scans 300
9 Pulse Width 10.0000
10 Acquisition Time 1.1534
11 Spectrometer Frequency 100.62
12 Spectral Width 28409.1
13 Lowest Frequency -4143.7
14 Nucleus 13C
15 Acquired Size 32768
16 Spectral Size 65536

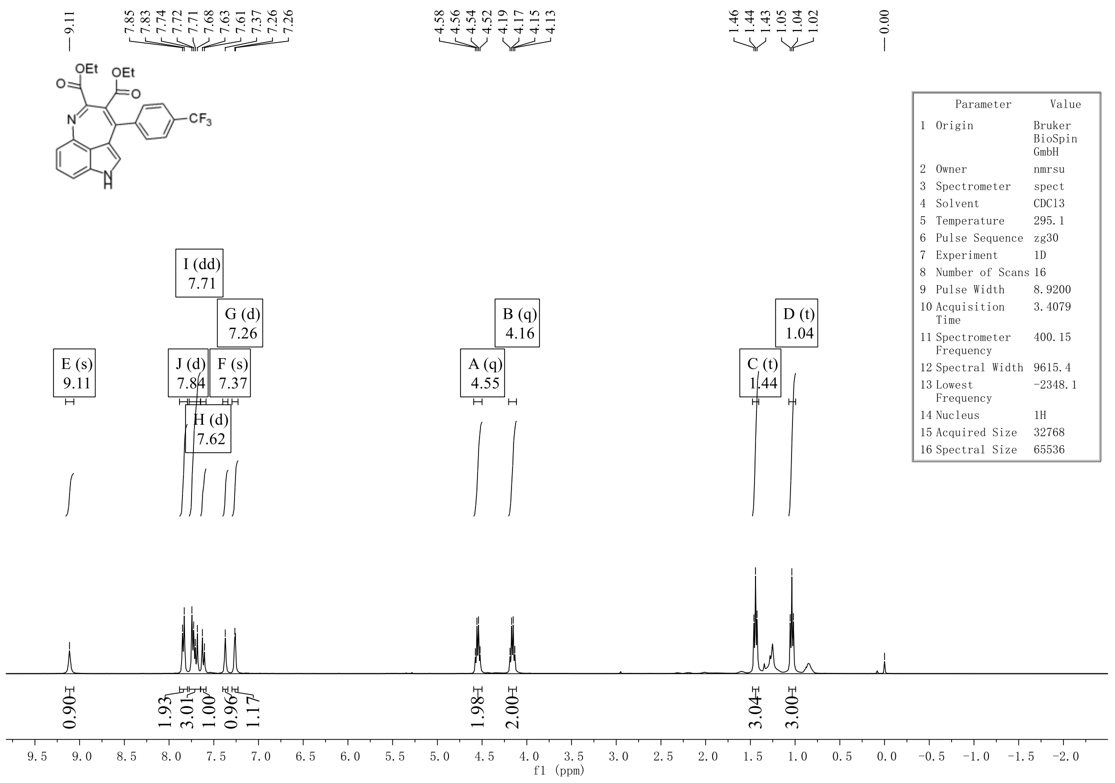

Parameter	Value
1 Origin	Bruker BioSpin GmbH
2 Owner	nmrsu
3 Spectrometer	spect
4 Solvent	CDCl3
5 Temperature	295.1
6 Pulse Sequence	zg30
7 Experiment	1D
8 Number of Scans	16
9 Pulse Width	8.9200
10 Acquisition Time	3.4079
11 Spectrometer Frequency	400.15
12 Spectral Width	9615.4
13 Lowest Frequency	-2348.1
14 Nucleus	1H
15 Acquired Size	32768
16 Spectral Size	65536

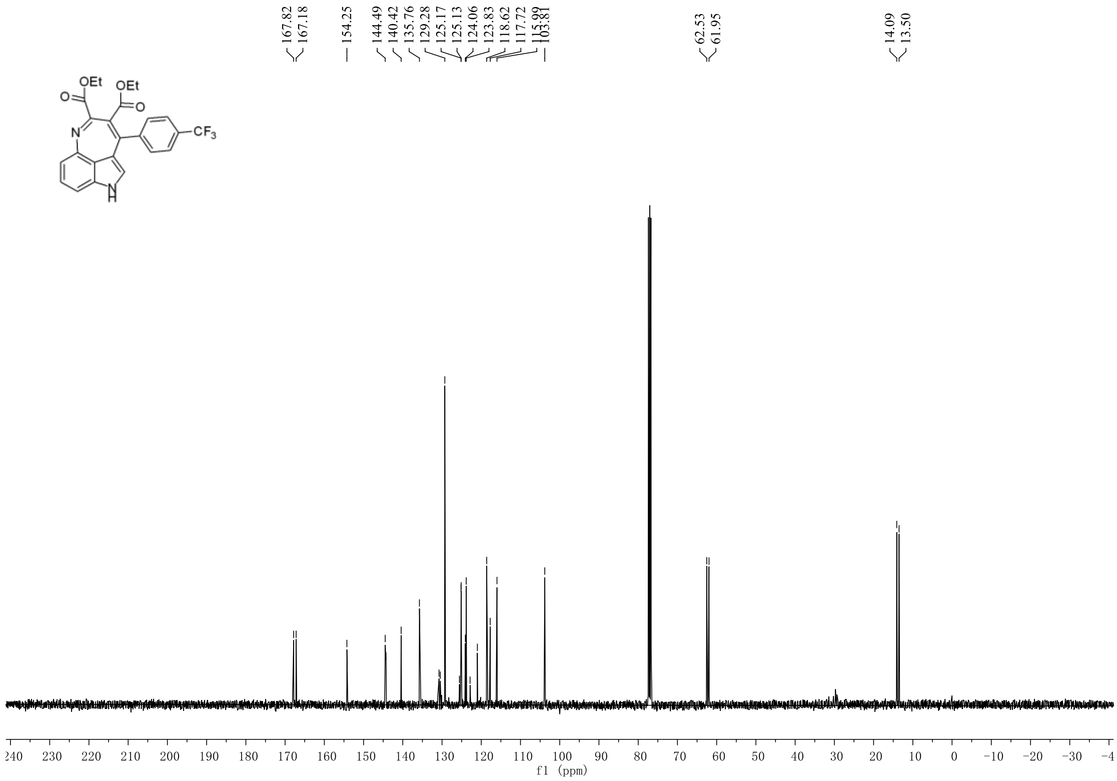

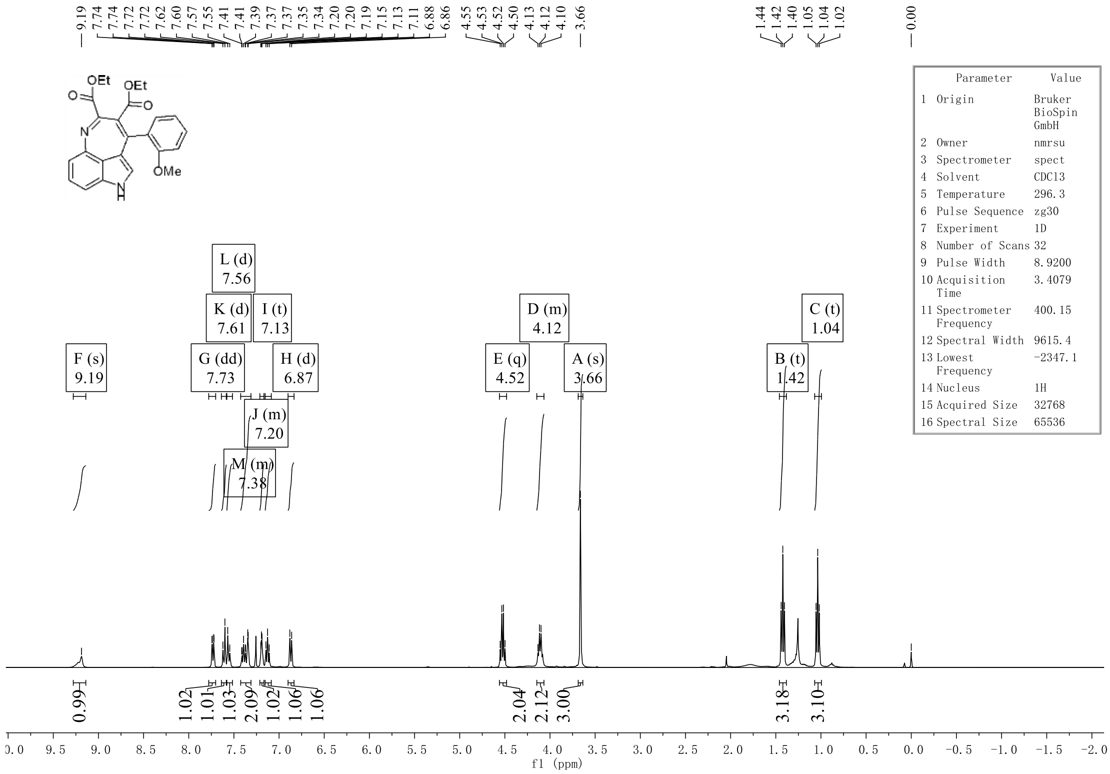

Parameter	Value
1 Origin	Bruker BioSpin GmbH
2 Owner	nmrsu
3 Spectrometer	spect
4 Solvent	CDCl3
5 Temperature	296.3
6 Pulse Sequence	zg30
7 Experiment	1D
8 Number of Scans	32
9 Pulse Width	8.9200
10 Acquisition Time	3.4079
11 Spectrometer Frequency	400.15
12 Spectral Width	9615.4
13 Lowest Frequency	-2347.1
14 Nucleus	1H
15 Acquired Size	32768
16 Spectral Size	65536

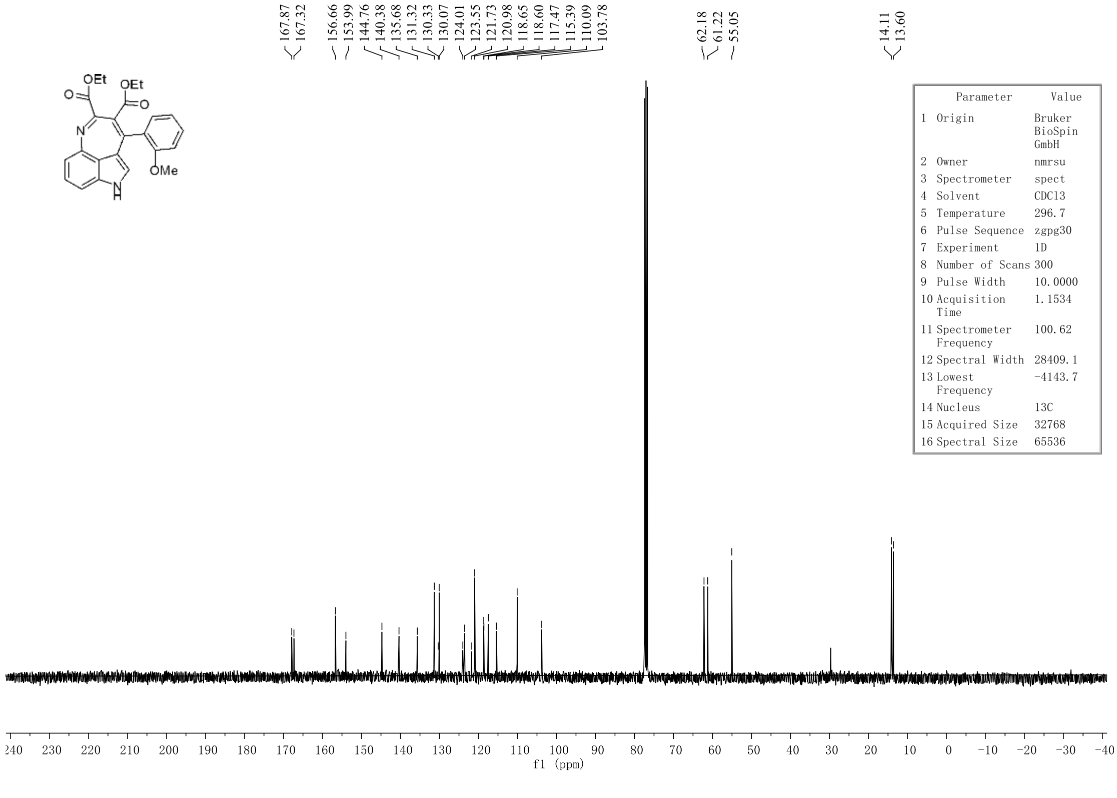

Parameter	Value
1 Origin	Bruker BioSpin GmbH
2 Owner	nmrsu
3 Spectrometer	spect
4 Solvent	CDCl3
5 Temperature	296.7
6 Pulse Sequence	zgpg30
7 Experiment	1D
8 Number of Scans	300
9 Pulse Width	10.0000
10 Acquisition Time	1.1534
11 Spectrometer Frequency	100.62
12 Spectral Width	28409.1
13 Lowest Frequency	-4143.7
14 Nucleus	13C
15 Acquired Size	32768
16 Spectral Size	65536

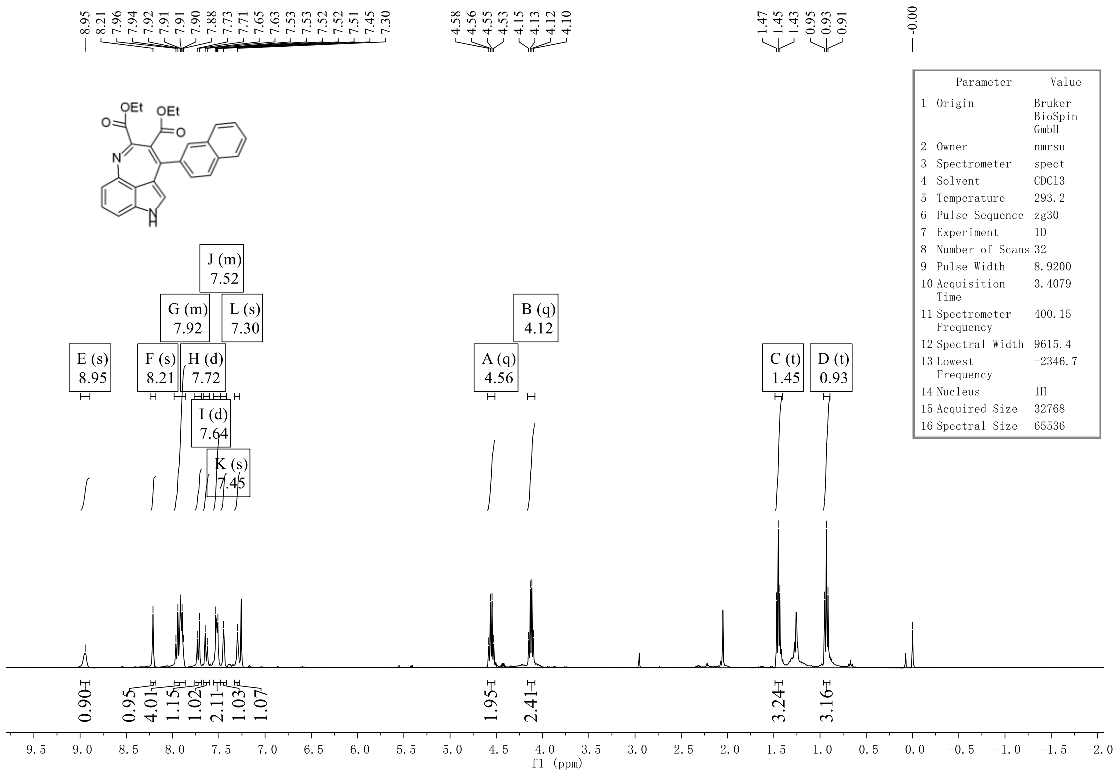
OEt
OEt
N
H
E (s) 8.95
F (s) 8.21
G (m) 7.92
H (d) 7.72
I (d) 7.64
J (m) 7.52
K (s) 7.45
L (s) 7.30
A (q) 4.56
B (q) 4.12
C (t) 1.45
D (t) 0.93
Parameter Value
1 Origin Bruker BioSpin GmbH
2 Owner nmrsu
3 Spectrometer spect
4 Solvent CDCl3
5 Temperature 293.2
6 Pulse Sequence zg30
7 Experiment 1D
8 Number of Scans 32
9 Pulse Width 8.9200
10 Acquisition Time 3.4079
11 Spectrometer Frequency 400.15
12 Spectral Width 9615.4
13 Lowest Frequency -2346.7
14 Nucleus 1H
15 Acquired Size 32768
16 Spectral Size 65536
f1 (ppm)

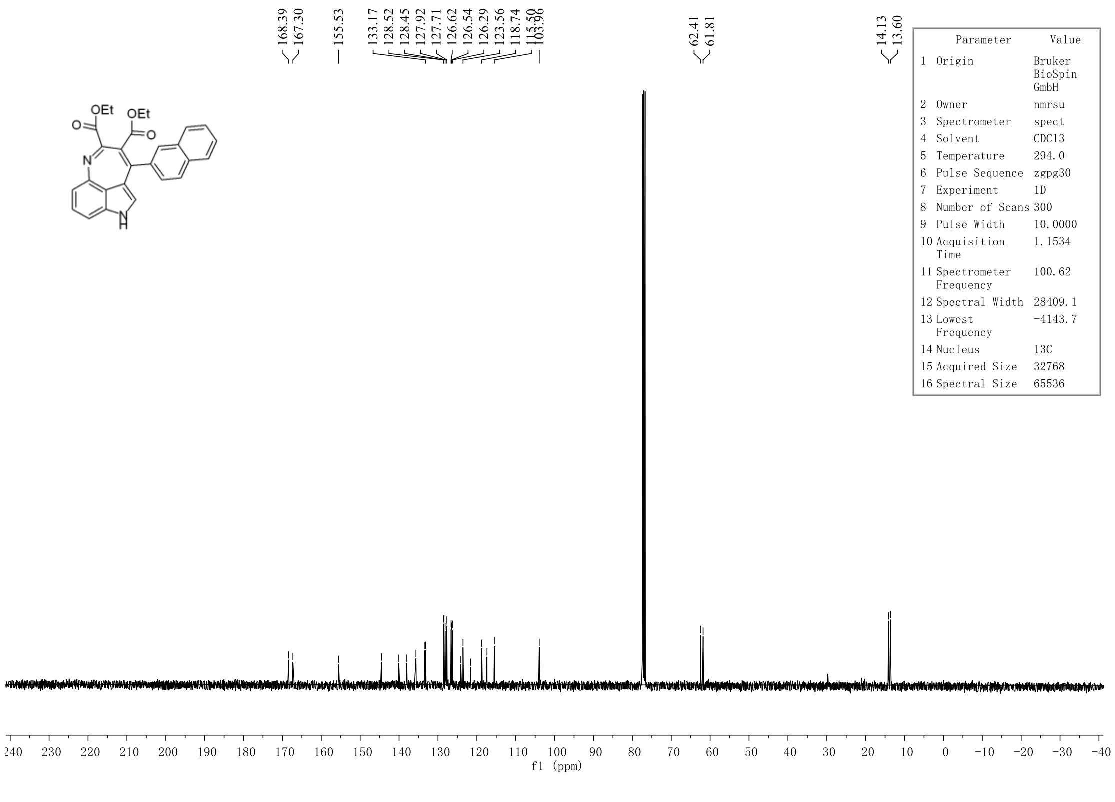
168.39
167.30
155.53
133.17
128.52
128.45
127.92
127.71
126.62
126.54
126.29
123.56
118.74
115.50
103.96
62.41
61.81
14.13
13.60
OEt
OEt
N
H
Parameter Value
1 Origin Bruker BioSpin GmbH
2 Owner nmrsu
3 Spectrometer spect
4 Solvent CDCl3
5 Temperature 294.0
6 Pulse Sequence zgpg30
7 Experiment 1D
8 Number of Scans 300
9 Pulse Width 10.0000
10 Acquisition Time 1.1534
11 Spectrometer Frequency 100.62
12 Spectral Width 28409.1
13 Lowest Frequency -4143.7
14 Nucleus 13C
15 Acquired Size 32768
16 Spectral Size 65536
f1 (ppm)

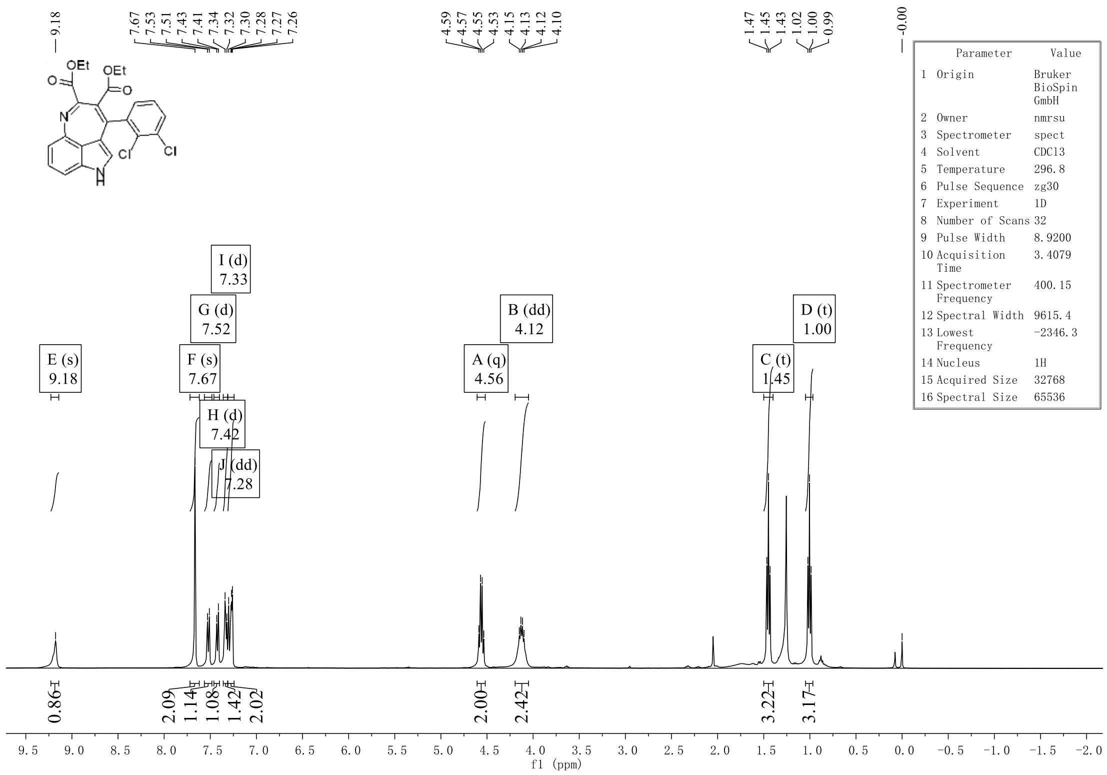
9.18
7.67 7.53 7.51 7.43 7.41 7.34 7.32 7.30 7.28 7.27 7.26
4.59 4.57 4.55 4.53 4.15 4.13 4.12 4.10
1.47 1.45 1.43 1.02 1.00 0.99
-0.00
OEt
OEt
N
Cl
Cl
H
E (s) 9.18
F (s) 7.67
G (d) 7.52
I (d) 7.33
H (d) 7.42
J (dd) 7.28
A (q) 4.56
B (dd) 4.12
C (t) 1.45
D (t) 1.00
0.86
2.09 1.14 1.08 1.42 2.02
2.00
2.42
3.22
3.17
f1 (ppm)
Parameter Value
1 Origin Bruker BioSpin GmbH
2 Owner nmrsu
3 Spectrometer spect
4 Solvent CDCl3
5 Temperature 296.8
6 Pulse Sequence zg30
7 Experiment 1D
8 Number of Scans 32
9 Pulse Width 8.9200
10 Acquisition Time 3.4079
11 Spectrometer Frequency 400.15
12 Spectral Width 9615.4
13 Lowest Frequency -2346.3
14 Nucleus 1H
15 Acquired Size 32768
16 Spectral Size 65536

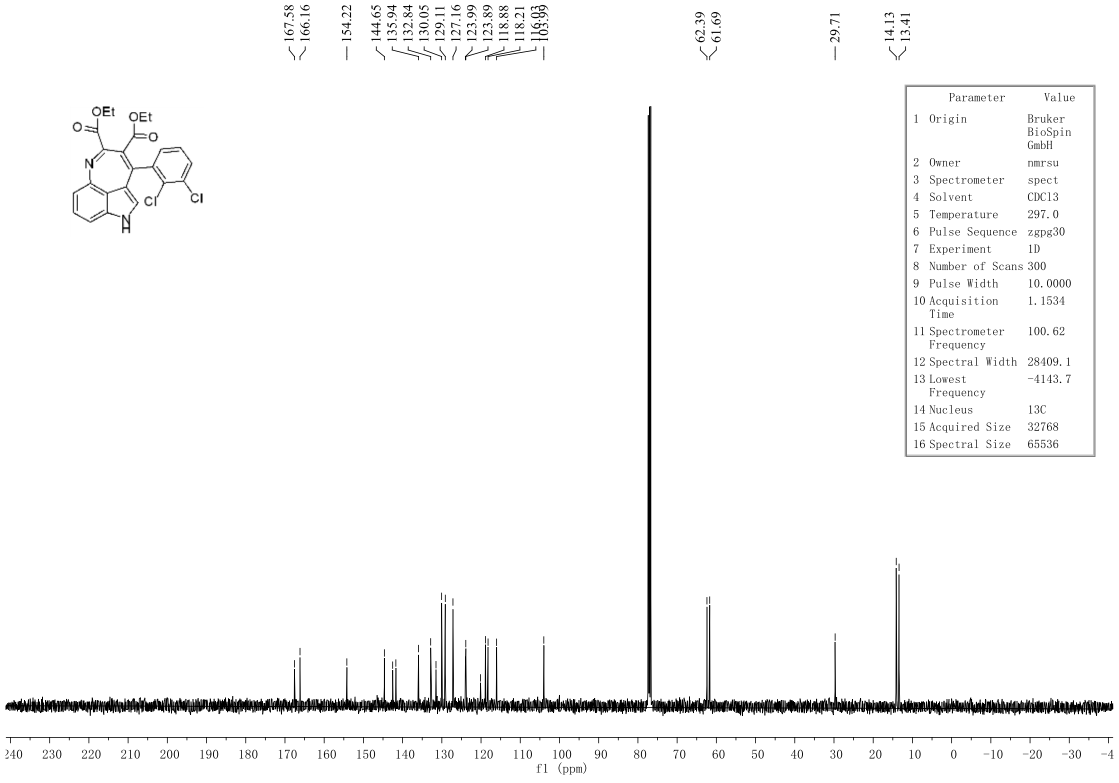
167.58 166.16
154.22
144.65 135.94 132.84 130.05 129.11 127.16 123.99 123.89 118.88 118.21 116.03 103.99
62.39 61.69
29.71
14.13 13.41
OEt
OEt
N
Cl
Cl
H
f1 (ppm)
Parameter Value
1 Origin Bruker BioSpin GmbH
2 Owner nmrsu
3 Spectrometer spect
4 Solvent CDCl3
5 Temperature 297.0
6 Pulse Sequence zgpg30
7 Experiment 1D
8 Number of Scans 300
9 Pulse Width 10.0000
10 Acquisition Time 1.1534
11 Spectrometer Frequency 100.62
12 Spectral Width 28409.1
13 Lowest Frequency -4143.7
14 Nucleus 13C
15 Acquired Size 32768
16 Spectral Size 65536

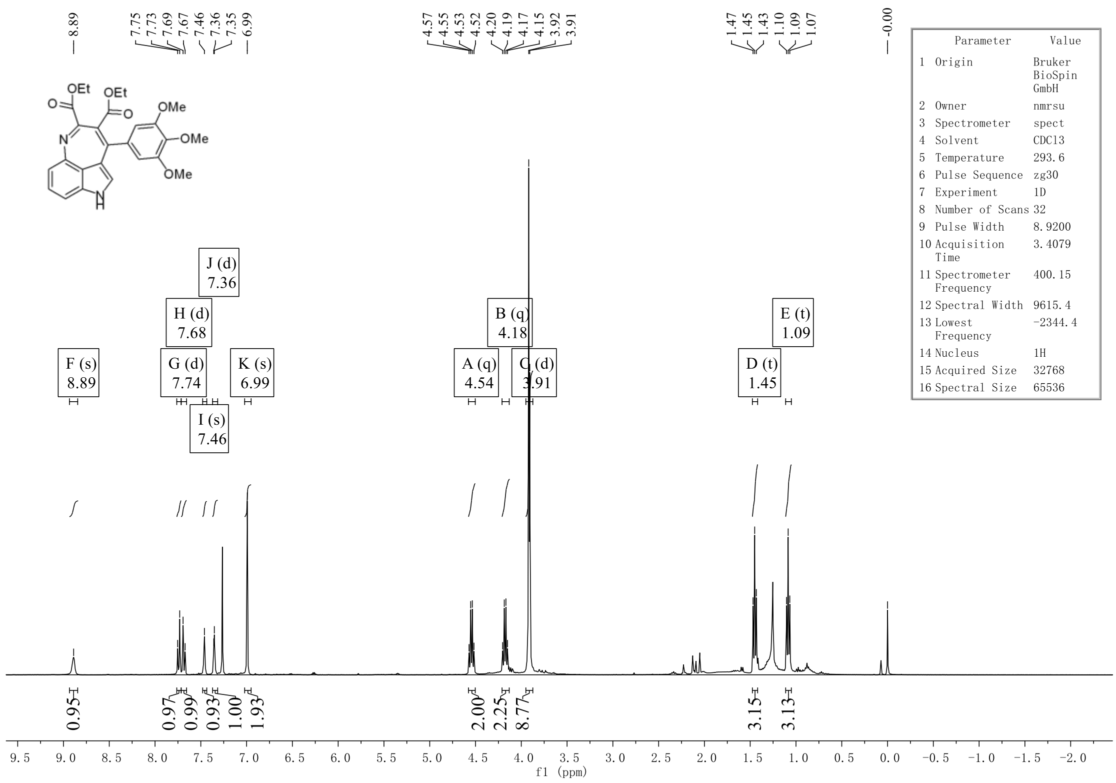

Parameter	Value
1 Origin	Bruker BioSpin GmbH
2 Owner	nmrsu
3 Spectrometer	spect
4 Solvent	CDCl3
5 Temperature	293.6
6 Pulse Sequence	zg30
7 Experiment	1D
8 Number of Scans	32
9 Pulse Width	8.9200
10 Acquisition Time	3.4079
11 Spectrometer Frequency	400.15
12 Spectral Width	9615.4
13 Lowest Frequency	-2344.4
14 Nucleus	1H
15 Acquired Size	32768
16 Spectral Size	65536

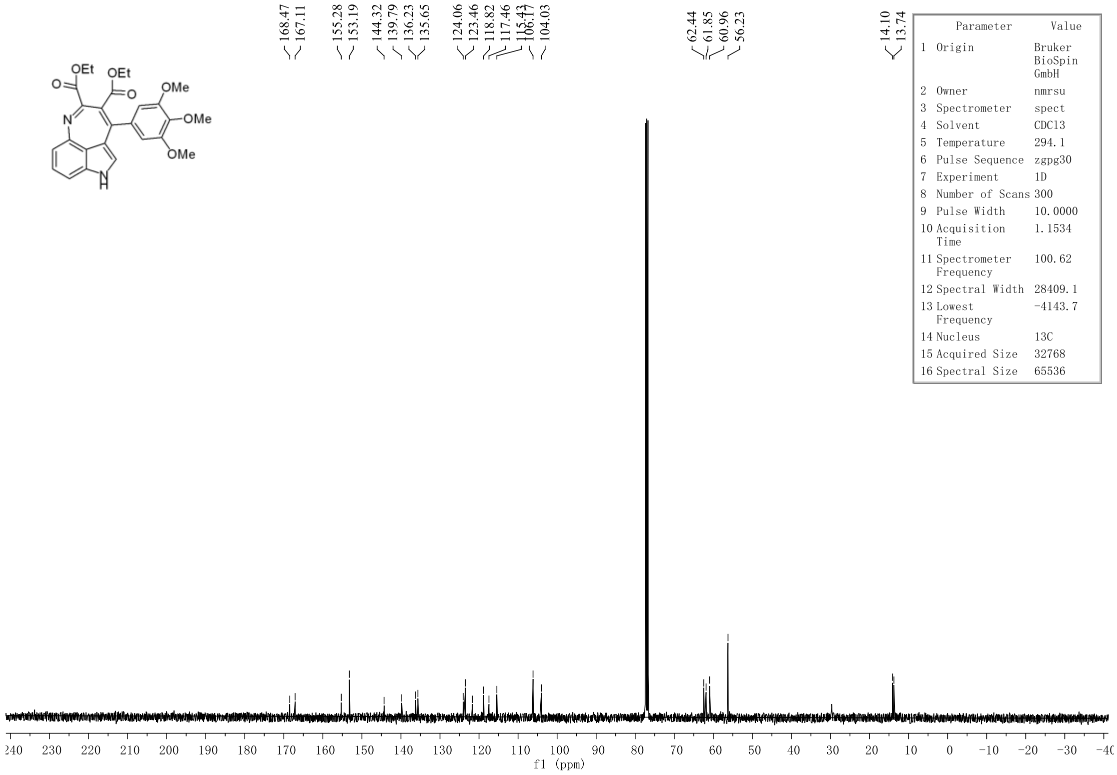

Parameter	Value
1 Origin	Bruker BioSpin GmbH
2 Owner	nmrsu
3 Spectrometer	spect
4 Solvent	CDCl3
5 Temperature	294.1
6 Pulse Sequence	zgpg30
7 Experiment	1D
8 Number of Scans	300
9 Pulse Width	10.0000
10 Acquisition Time	1.1534
11 Spectrometer Frequency	100.62
12 Spectral Width	28409.1
13 Lowest Frequency	-4143.7
14 Nucleus	13C
15 Acquired Size	32768
16 Spectral Size	65536

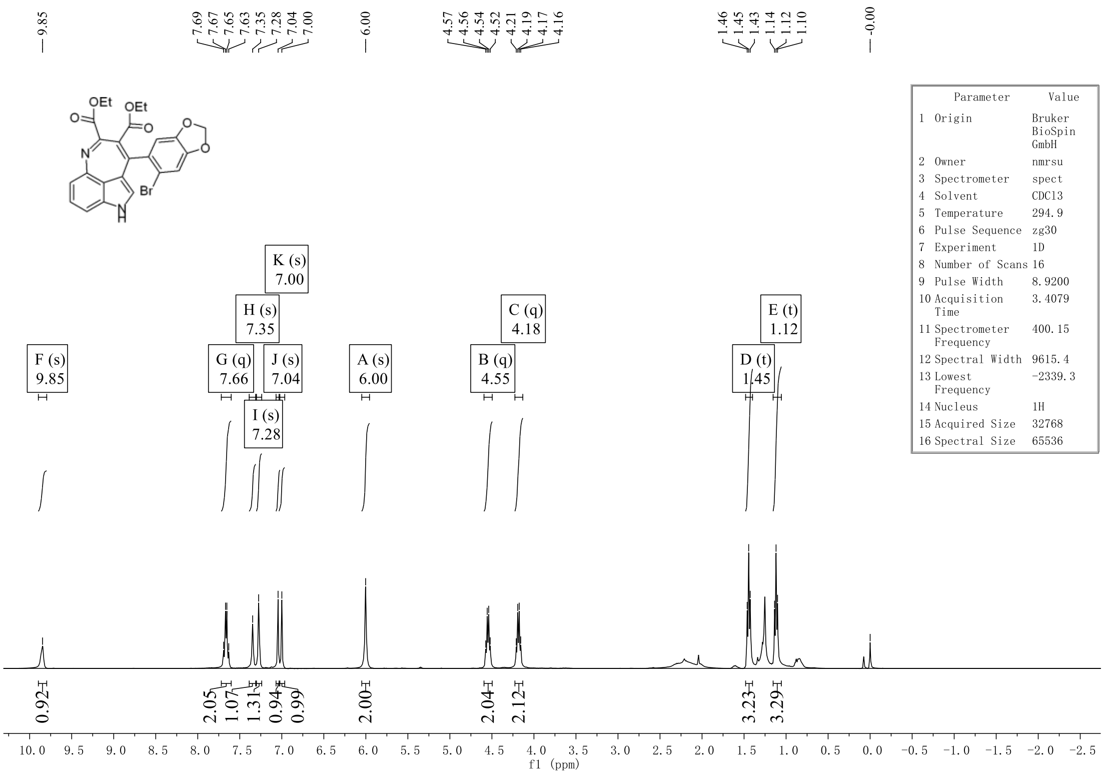

	Parameter	Value
1	Origin	Bruker BioSpin GmbH
2	Owner	nmrsu
3	Spectrometer	spect
4	Solvent	CDCl3
5	Temperature	294.9
6	Pulse Sequence	zg30
7	Experiment	1D
8	Number of Scans	16
9	Pulse Width	8.9200
10	Acquisition Time	3.4079
11	Spectrometer Frequency	400.15
12	Spectral Width	9615.4
13	Lowest Frequency	-2339.3
14	Nucleus	1H
15	Acquired Size	32768
16	Spectral Size	65536

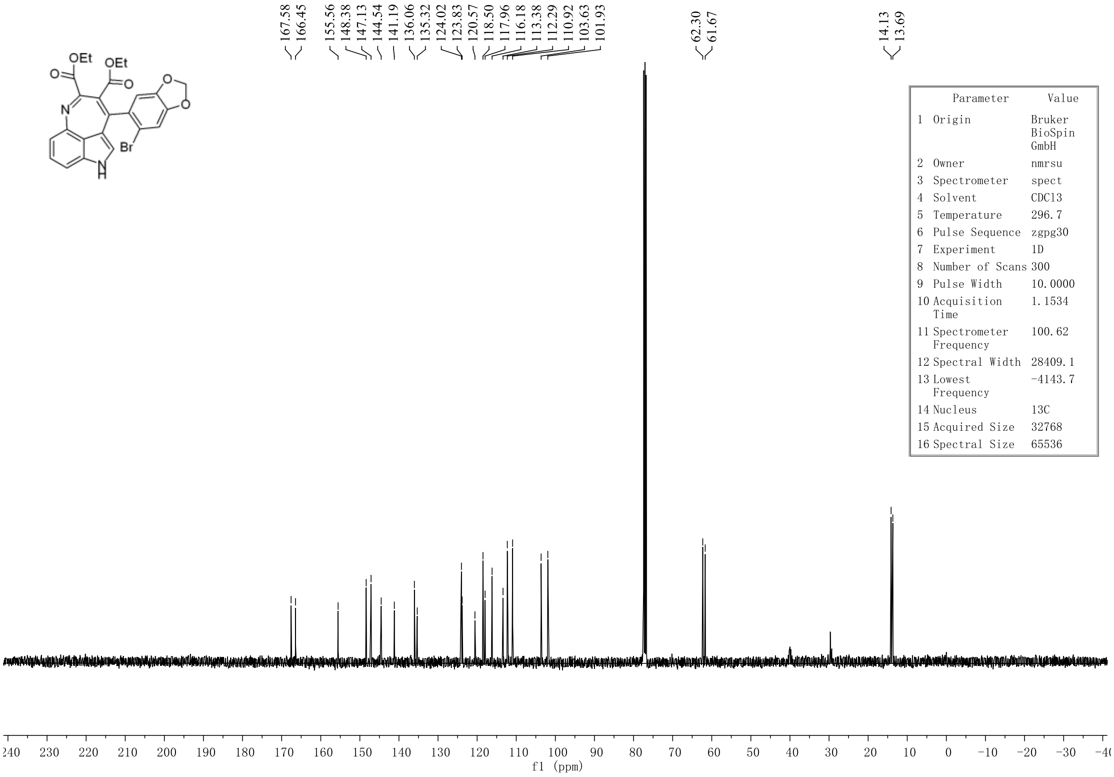

	Parameter	Value
1	Origin	Bruker BioSpin GmbH
2	Owner	nmrsu
3	Spectrometer	spect
4	Solvent	CDCl3
5	Temperature	296.7
6	Pulse Sequence	zgpg30
7	Experiment	1D
8	Number of Scans	300
9	Pulse Width	10.0000
10	Acquisition Time	1.1534
11	Spectrometer Frequency	100.62
12	Spectral Width	28409.1
13	Lowest Frequency	-4143.7
14	Nucleus	13C
15	Acquired Size	32768
16	Spectral Size	65536

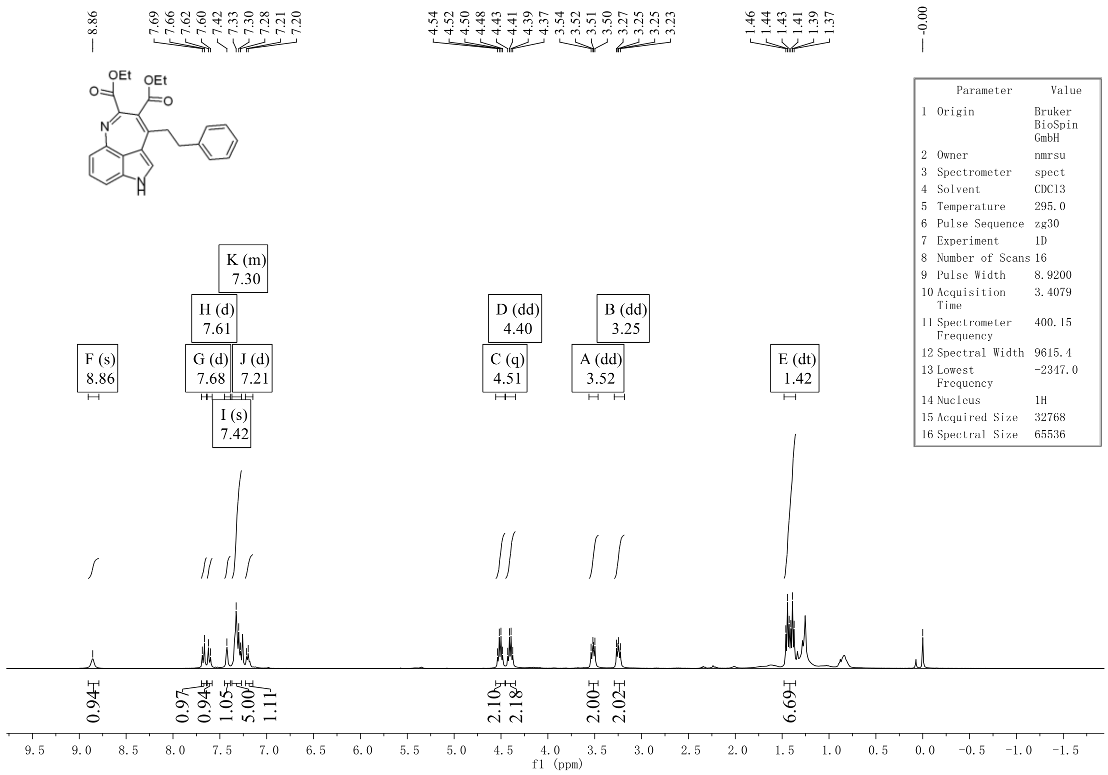

Parameter	Value
1 Origin	Bruker BioSpin GmbH
2 Owner	nmrsu
3 Spectrometer	spect
4 Solvent	CDCl3
5 Temperature	295.0
6 Pulse Sequence	zg30
7 Experiment	1D
8 Number of Scans	16
9 Pulse Width	8.9200
10 Acquisition Time	3.4079
11 Spectrometer Frequency	400.15
12 Spectral Width	9615.4
13 Lowest Frequency	-2347.0
14 Nucleus	1H
15 Acquired Size	32768
16 Spectral Size	65536

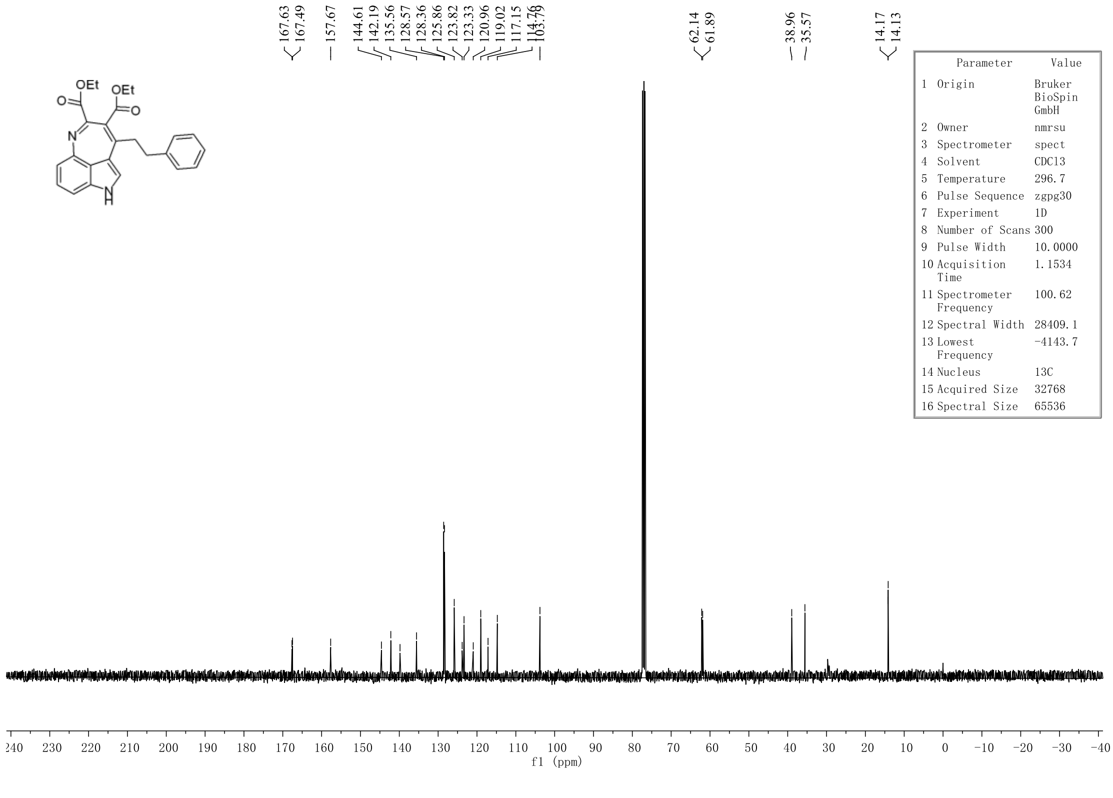

Parameter	Value
1 Origin	Bruker BioSpin GmbH
2 Owner	nmrsu
3 Spectrometer	spect
4 Solvent	CDCl3
5 Temperature	296.7
6 Pulse Sequence	zgpg30
7 Experiment	1D
8 Number of Scans	300
9 Pulse Width	10.0000
10 Acquisition Time	1.1534
11 Spectrometer Frequency	100.62
12 Spectral Width	28409.1
13 Lowest Frequency	-4143.7
14 Nucleus	13C
15 Acquired Size	32768
16 Spectral Size	65536

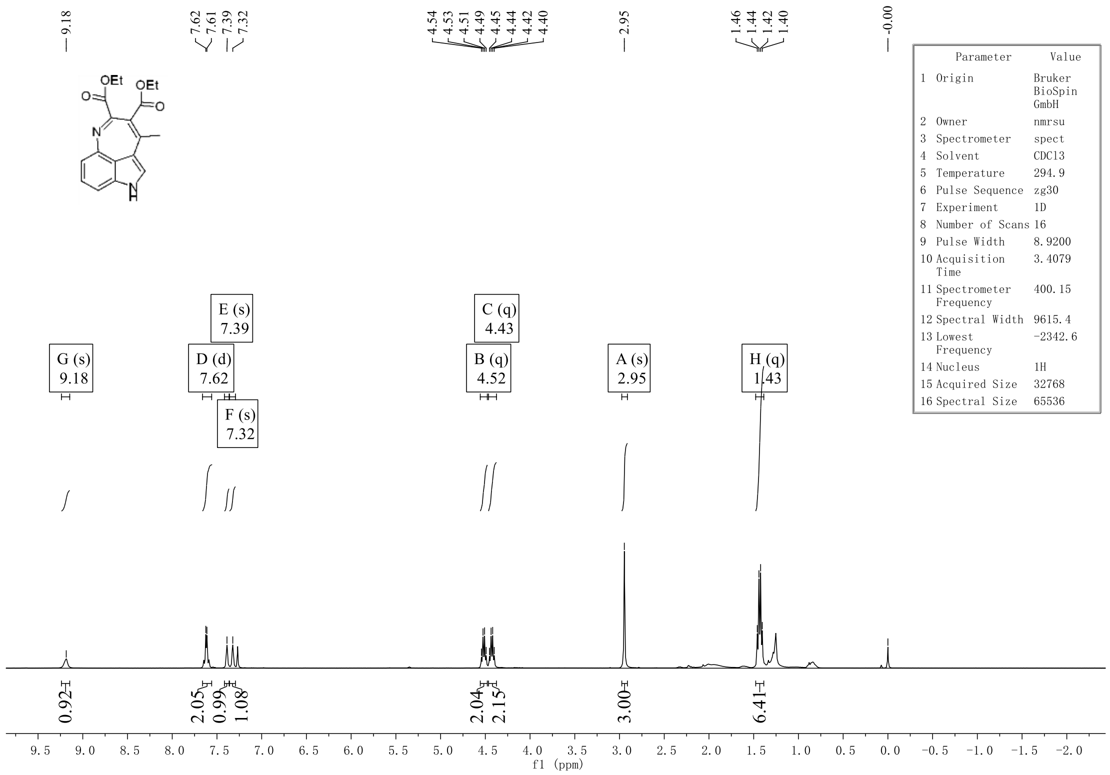

Parameter	Value
1 Origin	Bruker BioSpin GmbH
2 Owner	nmrsu
3 Spectrometer	spect
4 Solvent	CDCl3
5 Temperature	294.9
6 Pulse Sequence	zg30
7 Experiment	1D
8 Number of Scans	16
9 Pulse Width	8.9200
10 Acquisition Time	3.4079
11 Spectrometer Frequency	400.15
12 Spectral Width	9615.4
13 Lowest Frequency	-2342.6
14 Nucleus	1H
15 Acquired Size	32768
16 Spectral Size	65536

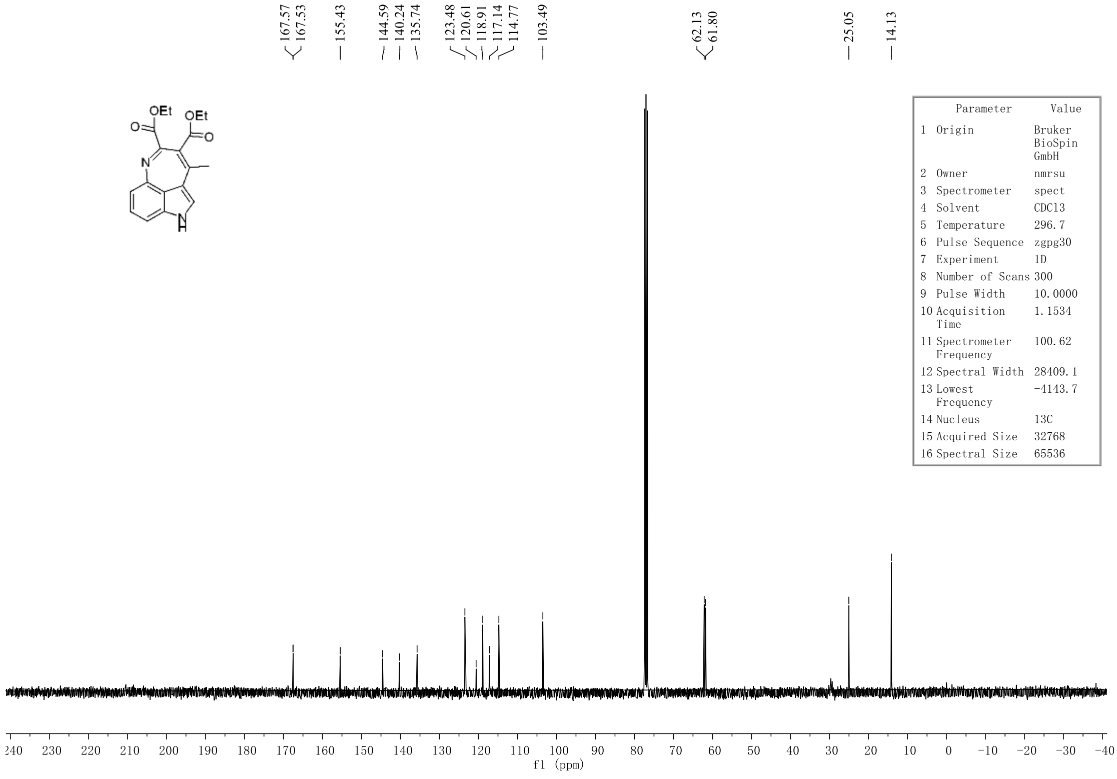

Parameter	Value
1 Origin	Bruker BioSpin GmbH
2 Owner	nmrsu
3 Spectrometer	spect
4 Solvent	CDCl3
5 Temperature	296.7
6 Pulse Sequence	zgpg30
7 Experiment	1D
8 Number of Scans	300
9 Pulse Width	10.0000
10 Acquisition Time	1.1534
11 Spectrometer Frequency	100.62
12 Spectral Width	28409.1
13 Lowest Frequency	-4143.7
14 Nucleus	13C
15 Acquired Size	32768
16 Spectral Size	65536

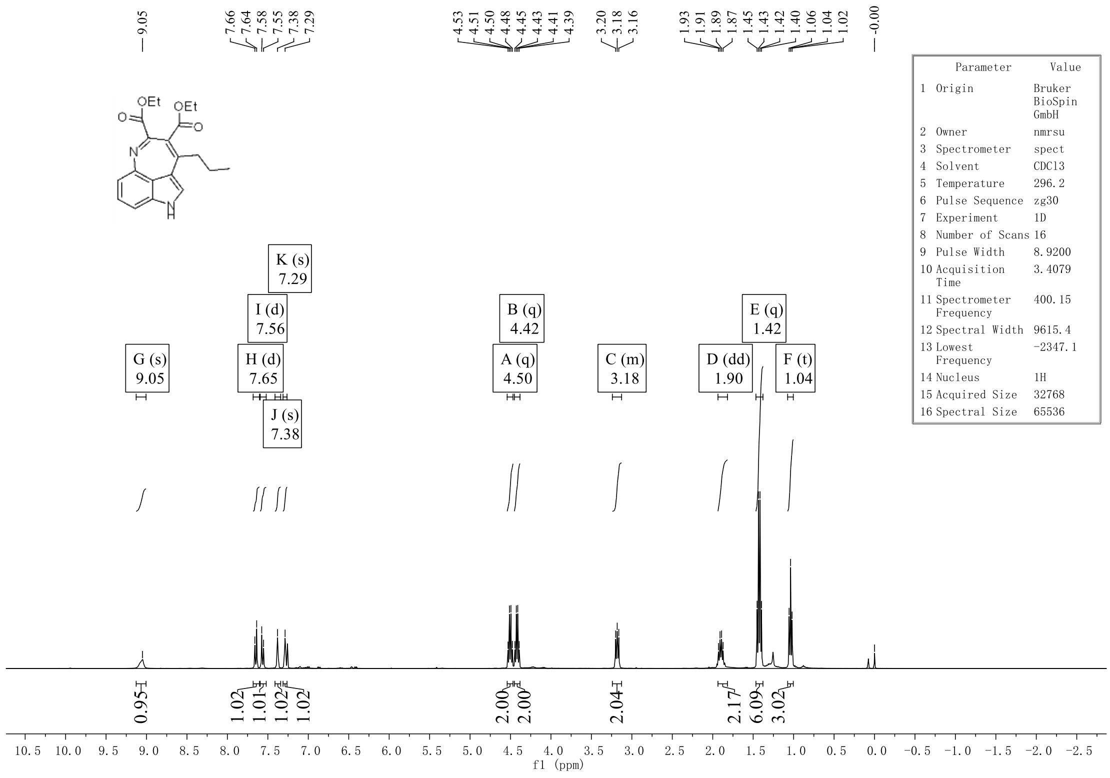

9.05
7.66
7.64
7.58
7.55
7.38
7.29
4.53
4.51
4.50
4.48
4.45
4.43
4.41
4.39
3.20
3.18
3.16
1.93
1.91
1.89
1.87
1.45
1.43
1.42
1.40
1.06
1.04
1.02
-0.00
OEt
OEt
N
H
K (s) 7.29
I (d) 7.56
H (d) 7.65
G (s) 9.05
J (s) 7.38
B (q) 4.42
A (q) 4.50
C (m) 3.18
D (dd) 1.90
E (q) 1.42
F (t) 1.04
0.95
1.02
1.01
1.02
1.02
2.00
2.00
2.04
2.17
6.09
3.02
f1 (ppm)
Parameter Value
1 Origin Bruker BioSpin GmbH
2 Owner nmrsu
3 Spectrometer spect
4 Solvent CDCl3
5 Temperature 296.2
6 Pulse Sequence zg30
7 Experiment 1D
8 Number of Scans 16
9 Pulse Width 8.9200
10 Acquisition Time 3.4079
11 Spectrometer Frequency 400.15
12 Spectral Width 9615.4
13 Lowest Frequency -2347.1
14 Nucleus 1H
15 Acquired Size 32768
16 Spectral Size 65536

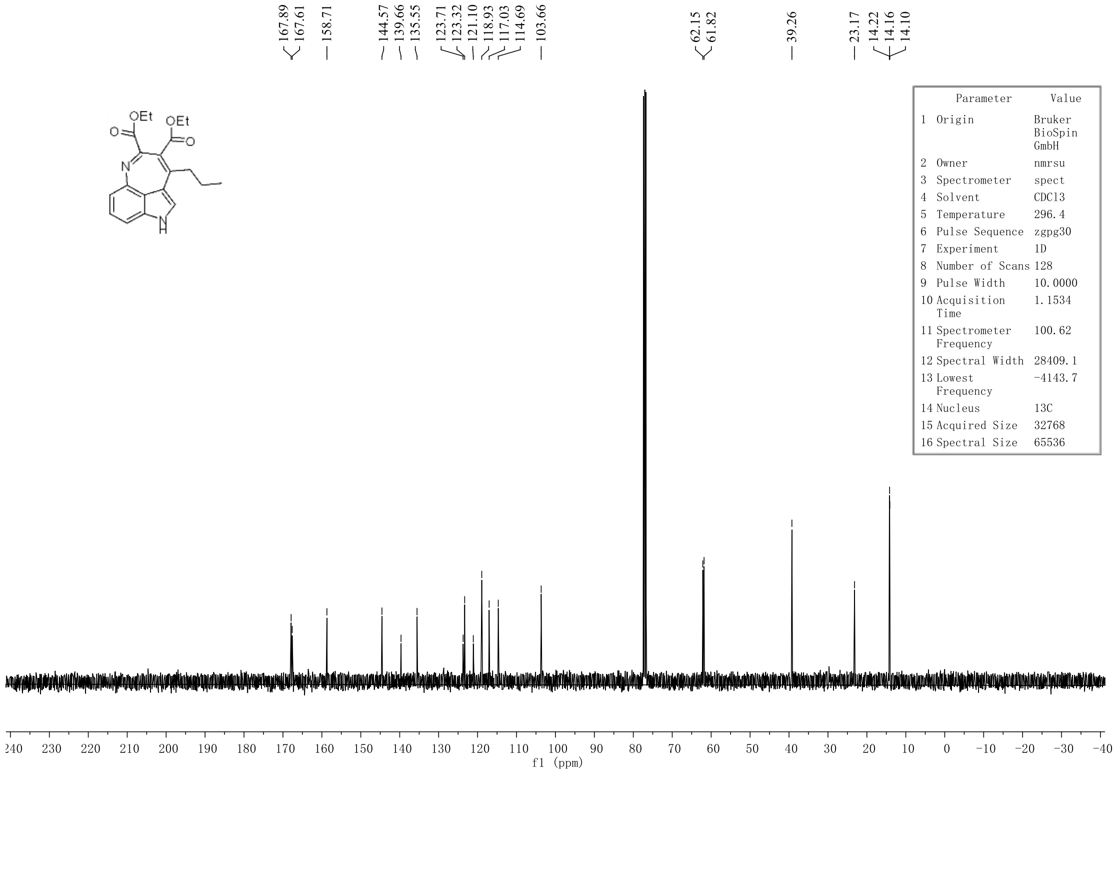

167.89
167.61
158.71
144.57
139.66
135.55
123.71
123.32
121.10
118.93
117.03
114.69
103.66
62.15
61.82
39.26
23.17
14.22
14.16
14.10
OEt
OEt
N
H
f1 (ppm)
Parameter Value
1 Origin Bruker BioSpin GmbH
2 Owner nmrsu
3 Spectrometer spect
4 Solvent CDCl3
5 Temperature 296.4
6 Pulse Sequence zgpg30
7 Experiment 1D
8 Number of Scans 128
9 Pulse Width 10.0000
10 Acquisition Time 1.1534
11 Spectrometer Frequency 100.62
12 Spectral Width 28409.1
13 Lowest Frequency -4143.7
14 Nucleus 13C
15 Acquired Size 32768
16 Spectral Size 65536

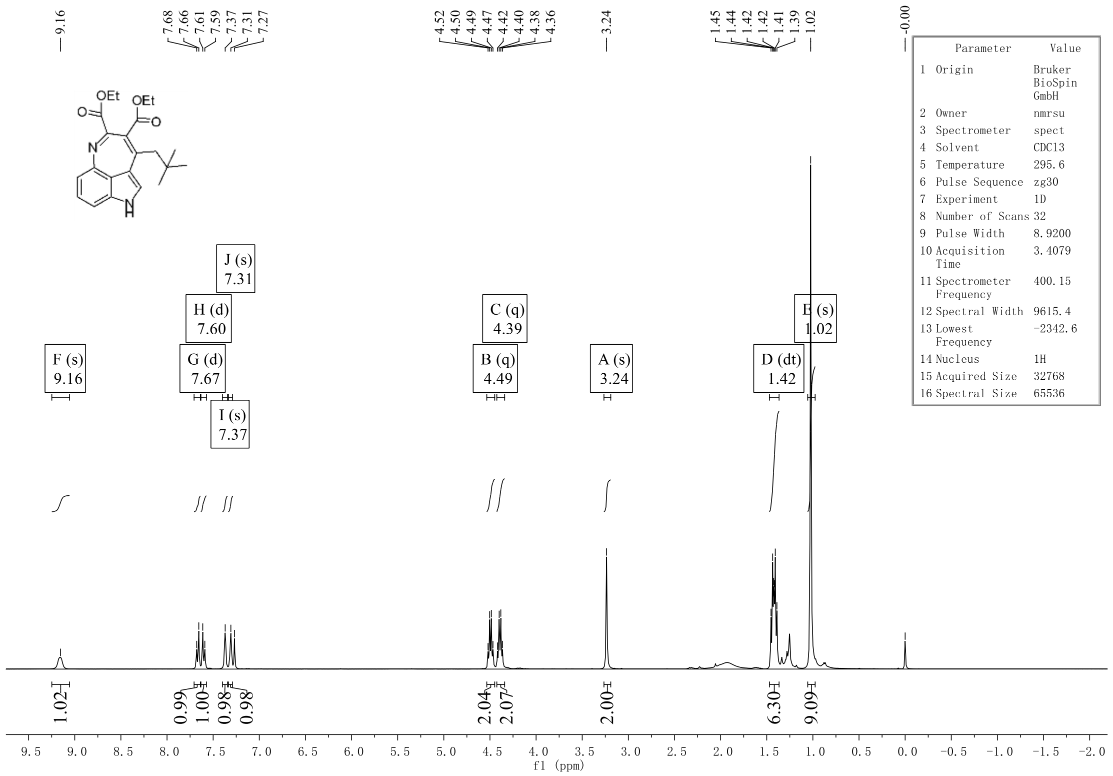
9.16
7.68
7.66
7.61
7.59
7.37
7.31
7.27
4.52
4.50
4.49
4.47
4.42
4.40
4.38
4.36
3.24
1.45
1.44
1.42
1.42
1.41
1.39
1.02
-0.00
OEt
OEt
O
O
N
H
J (s) 7.31
H (d) 7.60
C (q) 4.39
E (s) 1.02
F (s) 9.16
G (d) 7.67
B (q) 4.49
A (s) 3.24
D (dt) 1.42
I (s) 7.37
1.02
0.99
1.00
0.98
0.98
2.04
2.07
2.00
6.30
9.09
f1 (ppm)
Parameter Value
1 Origin Bruker BioSpin GmbH
2 Owner nmrsu
3 Spectrometer spect
4 Solvent CDCl3
5 Temperature 295.6
6 Pulse Sequence zg30
7 Experiment 1D
8 Number of Scans 32
9 Pulse Width 8.9200
10 Acquisition Time 3.4079
11 Spectrometer Frequency 400.15
12 Spectral Width 9615.4
13 Lowest Frequency -2342.6
14 Nucleus 1H
15 Acquired Size 32768
16 Spectral Size 65536

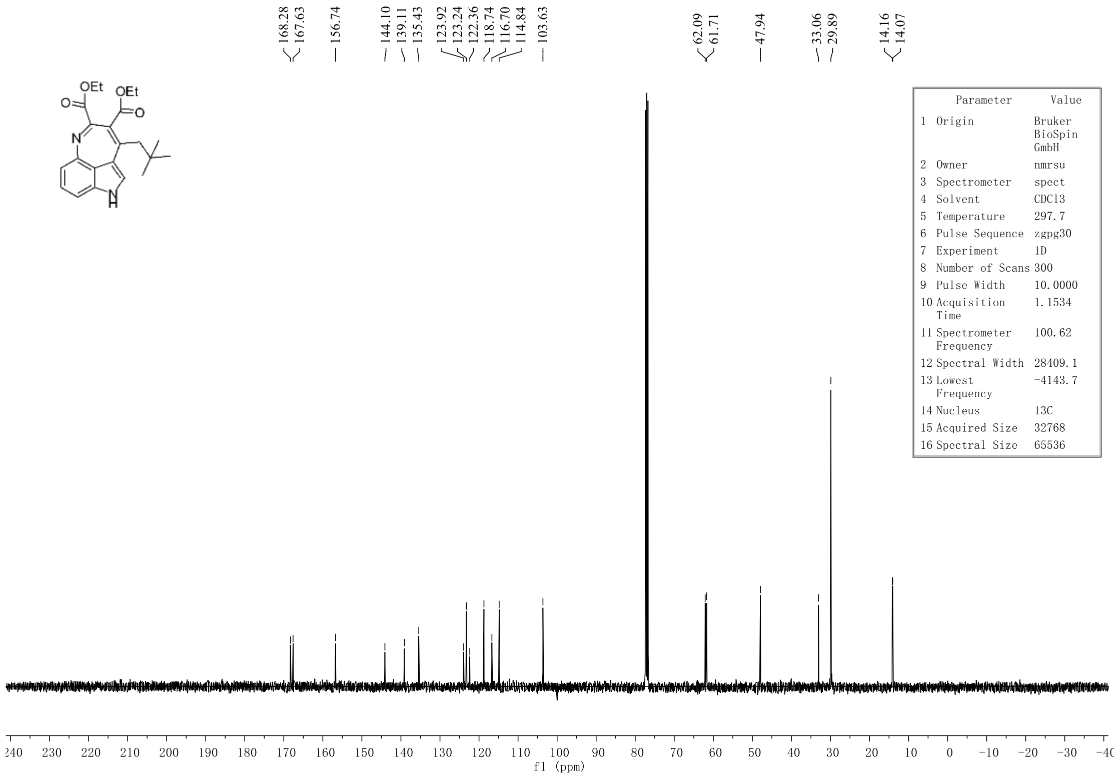
168.28
167.63
156.74
144.10
139.11
135.43
123.92
123.24
122.36
118.74
116.70
114.84
103.63
62.09
61.71
47.94
33.06
29.89
14.16
14.07
OEt
OEt
O
O
N
H
f1 (ppm)
Parameter Value
1 Origin Bruker BioSpin GmbH
2 Owner nmrsu
3 Spectrometer spect
4 Solvent CDCl3
5 Temperature 297.7
6 Pulse Sequence zgpg30
7 Experiment 1D
8 Number of Scans 300
9 Pulse Width 10.0000
10 Acquisition Time 1.1534
11 Spectrometer Frequency 100.62
12 Spectral Width 28409.1
13 Lowest Frequency -4143.7
14 Nucleus 13C
15 Acquired Size 32768
16 Spectral Size 65536

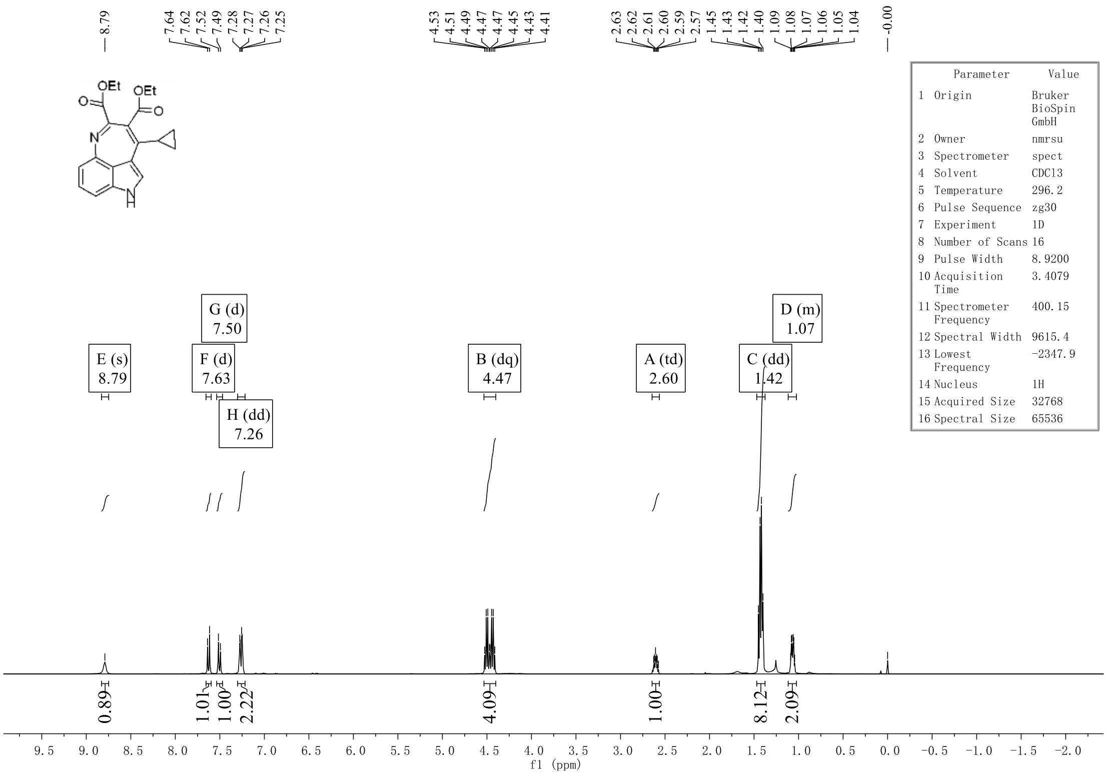
OEt
OEt
N
H
E (s) 8.79
F (d) 7.63
G (d) 7.50
H (dd) 7.26
B (dq) 4.47
A (td) 2.60
C (dd) 1.42
D (m) 1.07
0.89
1.01
1.00
2.22
4.09
1.00
8.12
2.09
f1 (ppm)
Parameter Value
1 Origin Bruker BioSpin GmbH
2 Owner nmrsu
3 Spectrometer spect
4 Solvent CDCl3
5 Temperature 296.2
6 Pulse Sequence zg30
7 Experiment 1D
8 Number of Scans 16
9 Pulse Width 8.9200
10 Acquisition Time 3.4079
11 Spectrometer Frequency 400.15
12 Spectral Width 9615.4
13 Lowest Frequency -2347.9
14 Nucleus 1H
15 Acquired Size 32768
16 Spectral Size 65536

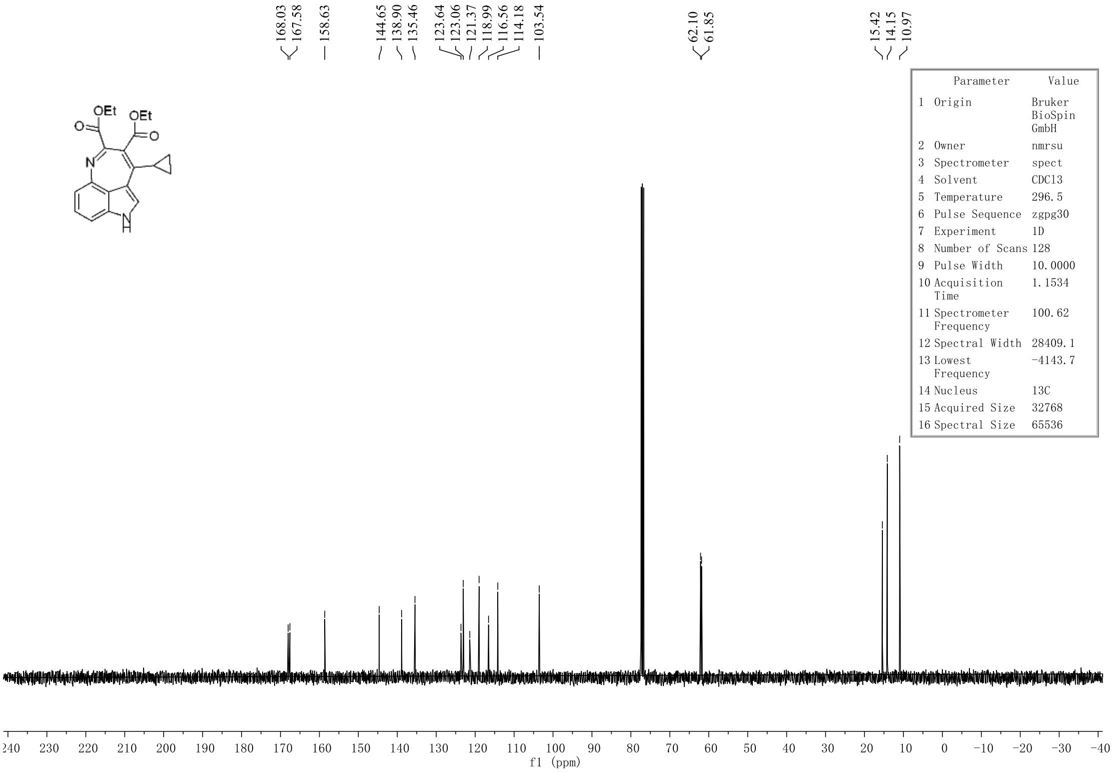
OEt
OEt
N
H
168.03
167.58
158.63
144.65
138.90
135.46
123.64
123.06
121.37
118.99
116.56
114.18
103.54
62.10
61.85
15.42
14.15
10.97
f1 (ppm)
Parameter Value
1 Origin Bruker BioSpin GmbH
2 Owner nmrsu
3 Spectrometer spect
4 Solvent CDCl3
5 Temperature 296.5
6 Pulse Sequence zgpg30
7 Experiment 1D
8 Number of Scans 128
9 Pulse Width 10.0000
10 Acquisition Time 1.1534
11 Spectrometer Frequency 100.62
12 Spectral Width 28409.1
13 Lowest Frequency -4143.7
14 Nucleus 13C
15 Acquired Size 32768
16 Spectral Size 65536

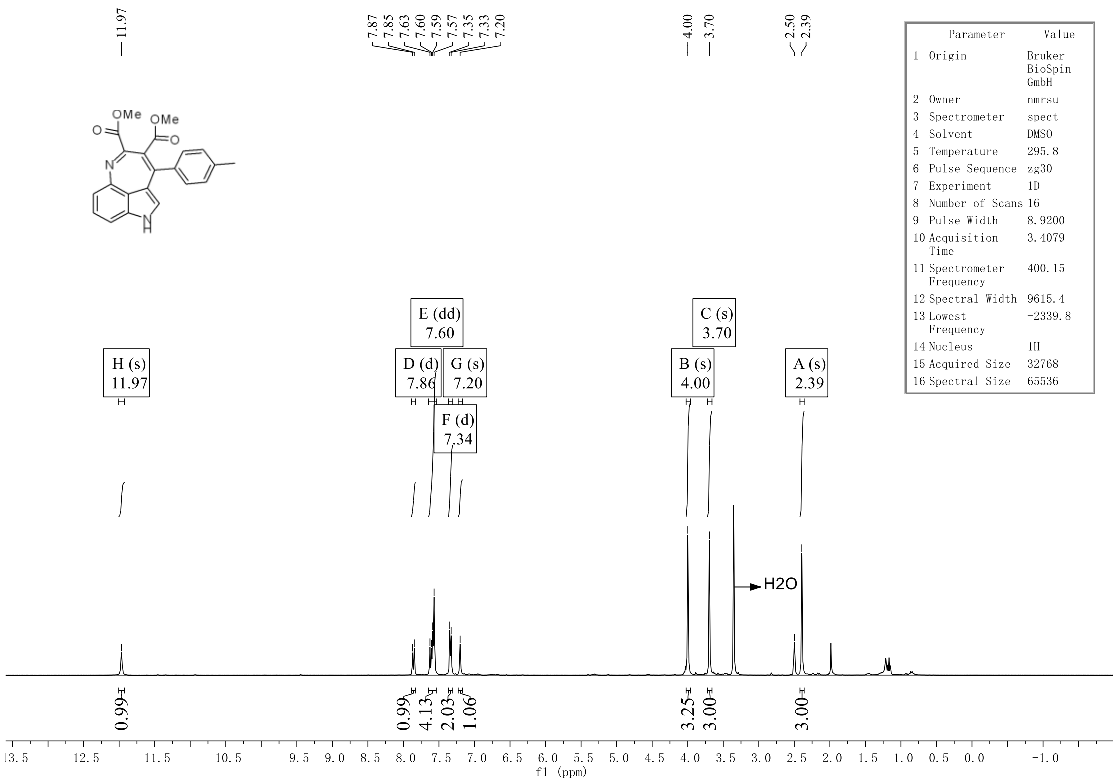
11.97
7.87 7.85 7.63 7.60 7.59 7.57 7.35 7.33 7.20
4.00 3.70
2.50 2.39
OMe
O
OMe
O
N
NH
Parameter Value
1 Origin Bruker BioSpin GmbH
2 Owner nmrsu
3 Spectrometer spect
4 Solvent DMSO
5 Temperature 295.8
6 Pulse Sequence zg30
7 Experiment 1D
8 Number of Scans 16
9 Pulse Width 8.9200
10 Acquisition Time 3.4079
11 Spectrometer Frequency 400.15
12 Spectral Width 9615.4
13 Lowest Frequency -2339.8
14 Nucleus 1H
15 Acquired Size 32768
16 Spectral Size 65536
H (s) 11.97
E (dd) 7.60
D (d) 7.86
G (s) 7.20
F (d) 7.34
C (s) 3.70
B (s) 4.00
A (s) 2.39
H2O
0.99
0.99
4.13
2.03
1.06
3.25
3.00
3.00
f1 (ppm)

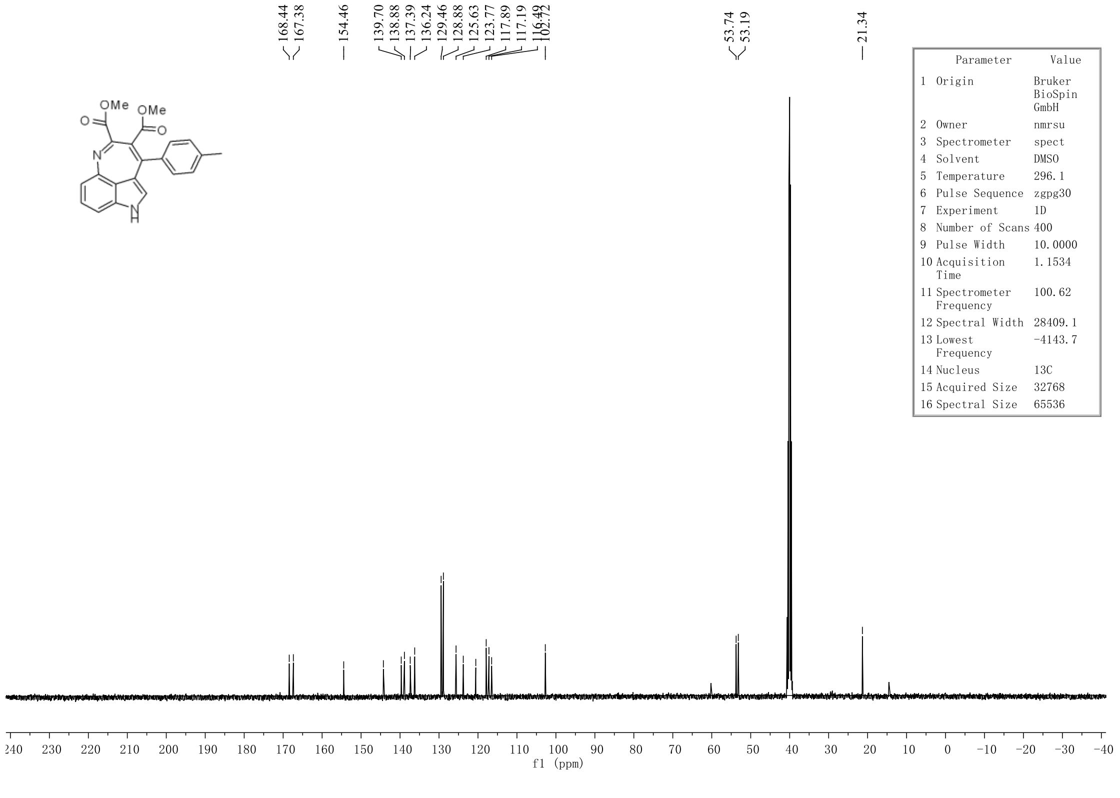
168.44 167.38
154.46
139.70 138.88 137.39 136.24 129.46 128.88 125.63 123.77 117.89 117.19 116.49 102.72
53.74 53.19
21.34
OMe
O
OMe
O
N
NH
Parameter Value
1 Origin Bruker BioSpin GmbH
2 Owner nmrsu
3 Spectrometer spect
4 Solvent DMSO
5 Temperature 296.1
6 Pulse Sequence zgpg30
7 Experiment 1D
8 Number of Scans 400
9 Pulse Width 10.0000
10 Acquisition Time 1.1534
11 Spectrometer Frequency 100.62
12 Spectral Width 28409.1
13 Lowest Frequency -4143.7
14 Nucleus 13C
15 Acquired Size 32768
16 Spectral Size 65536
f1 (ppm)

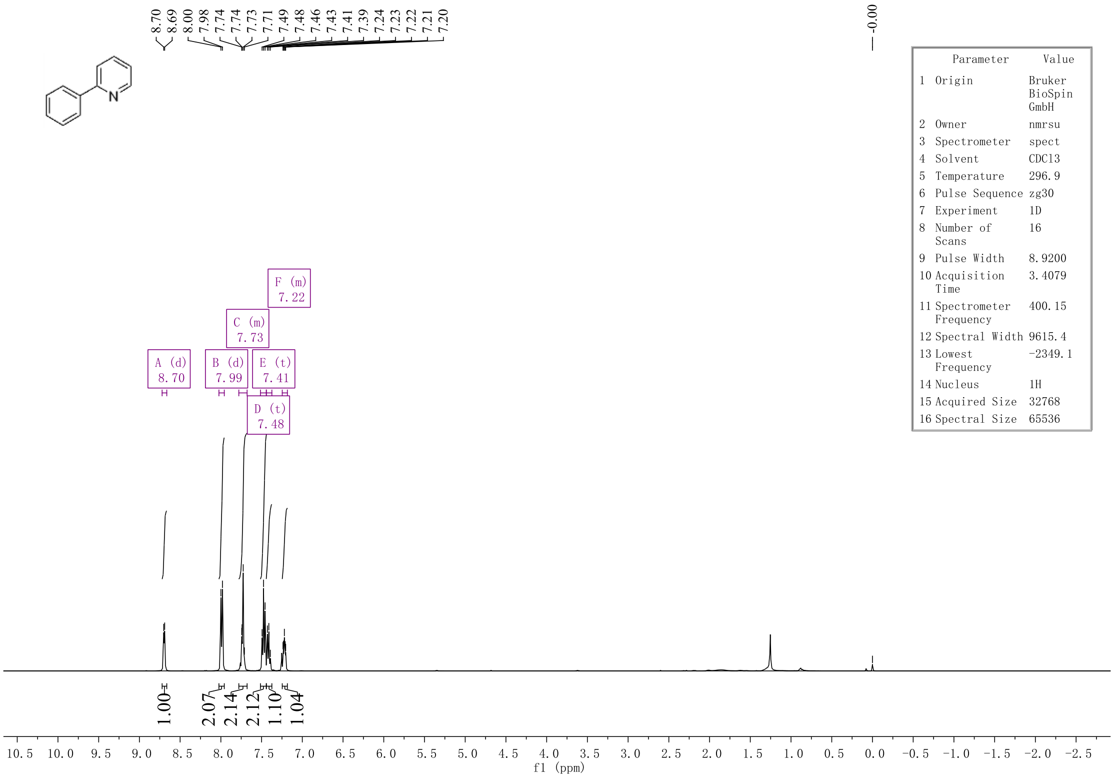

	Parameter	Value
1	Origin	Bruker BioSpin GmbH
2	Owner	nmrsu
3	Spectrometer	spect
4	Solvent	CDCl3
5	Temperature	296.9
6	Pulse Sequence	zg30
7	Experiment	1D
8	Number of Scans	16
9	Pulse Width	8.9200
10	Acquisition Time	3.4079
11	Spectrometer Frequency	400.15
12	Spectral Width	9615.4
13	Lowest Frequency	-2349.1
14	Nucleus	1H
15	Acquired Size	32768
16	Spectral Size	65536

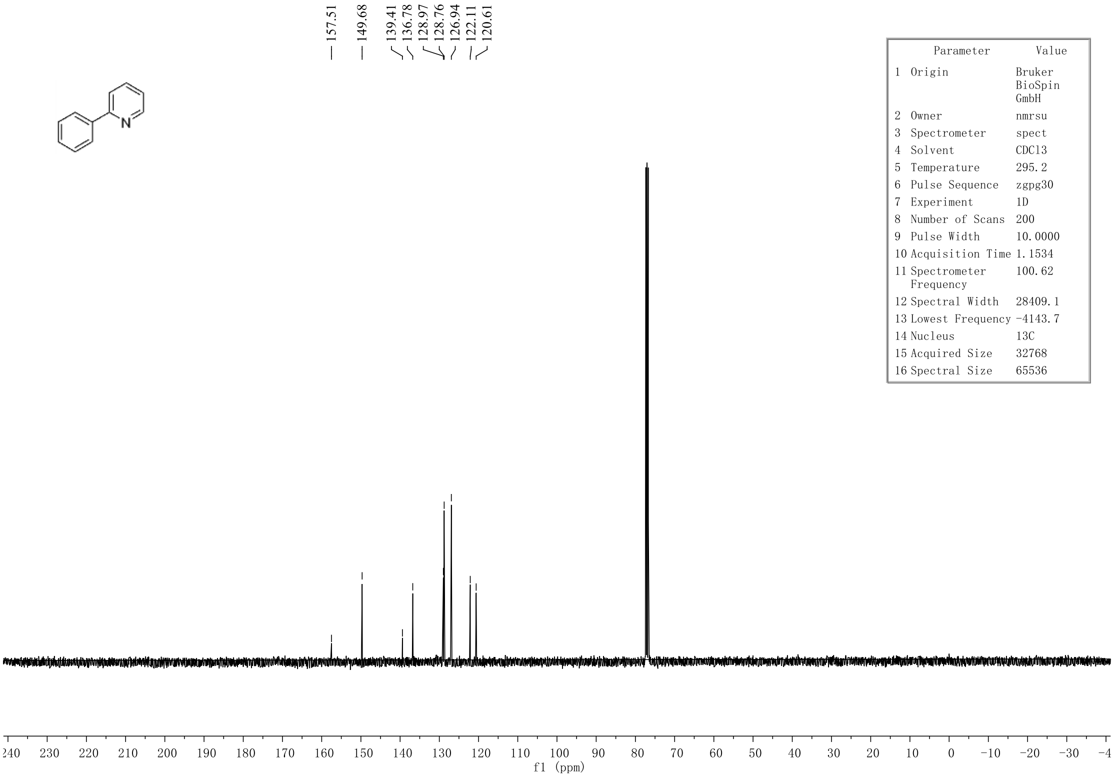

	Parameter	Value
1	Origin	Bruker BioSpin GmbH
2	Owner	nmrsu
3	Spectrometer	spect
4	Solvent	CDCl3
5	Temperature	295.2
6	Pulse Sequence	zgpg30
7	Experiment	1D
8	Number of Scans	200
9	Pulse Width	10.0000
10	Acquisition Time	1.1534
11	Spectrometer Frequency	100.62
12	Spectral Width	28409.1
13	Lowest Frequency	-4143.7
14	Nucleus	13C
15	Acquired Size	32768
16	Spectral Size	65536

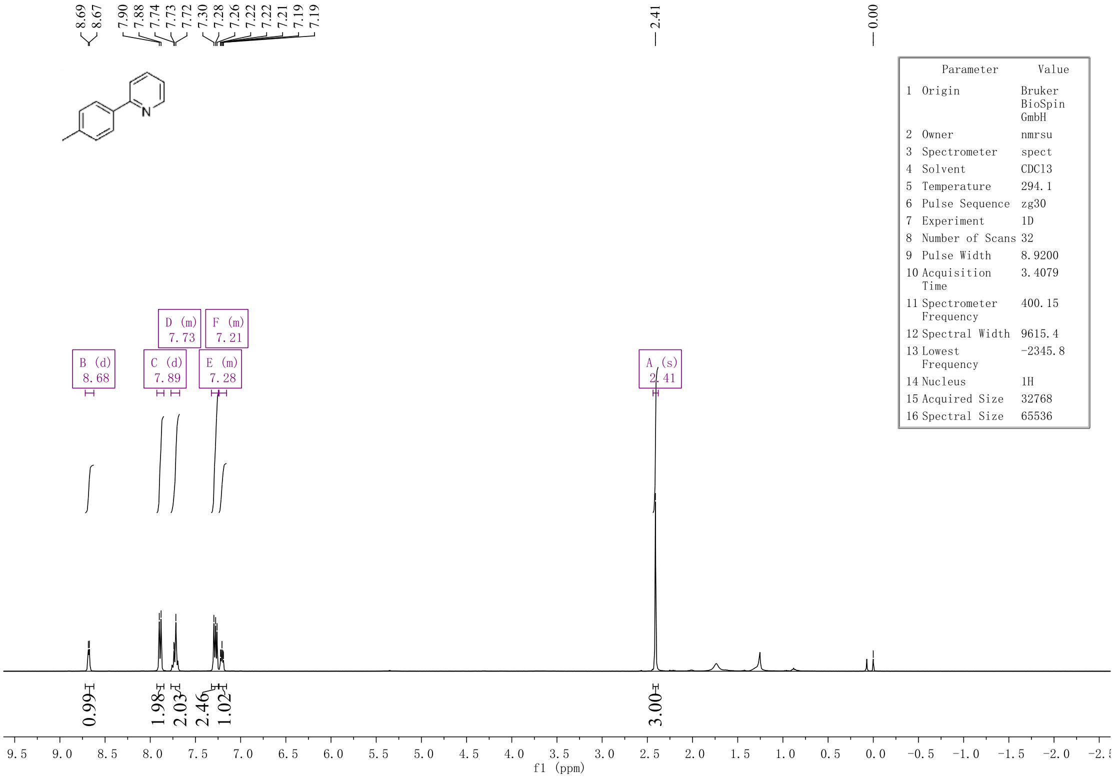
8.69
8.67
7.90
7.88
7.74
7.73
7.72
7.30
7.28
7.26
7.22
7.22
7.21
7.19
7.19
2.41
0.00
N
Parameter Value
1 Origin Bruker BioSpin GmbH
2 Owner nmrsu
3 Spectrometer spect
4 Solvent CDCl3
5 Temperature 294.1
6 Pulse Sequence zg30
7 Experiment 1D
8 Number of Scans 32
9 Pulse Width 8.9200
10 Acquisition Time 3.4079
11 Spectrometer Frequency 400.15
12 Spectral Width 9615.4
13 Lowest Frequency -2345.8
14 Nucleus 1H
15 Acquired Size 32768
16 Spectral Size 65536
D (m) 7.73
F (m) 7.21
B (d) 8.68
C (d) 7.89
E (m) 7.28
A (s) 2.41
0.99
1.98
2.03
2.46
1.02
3.00
f1 (ppm)

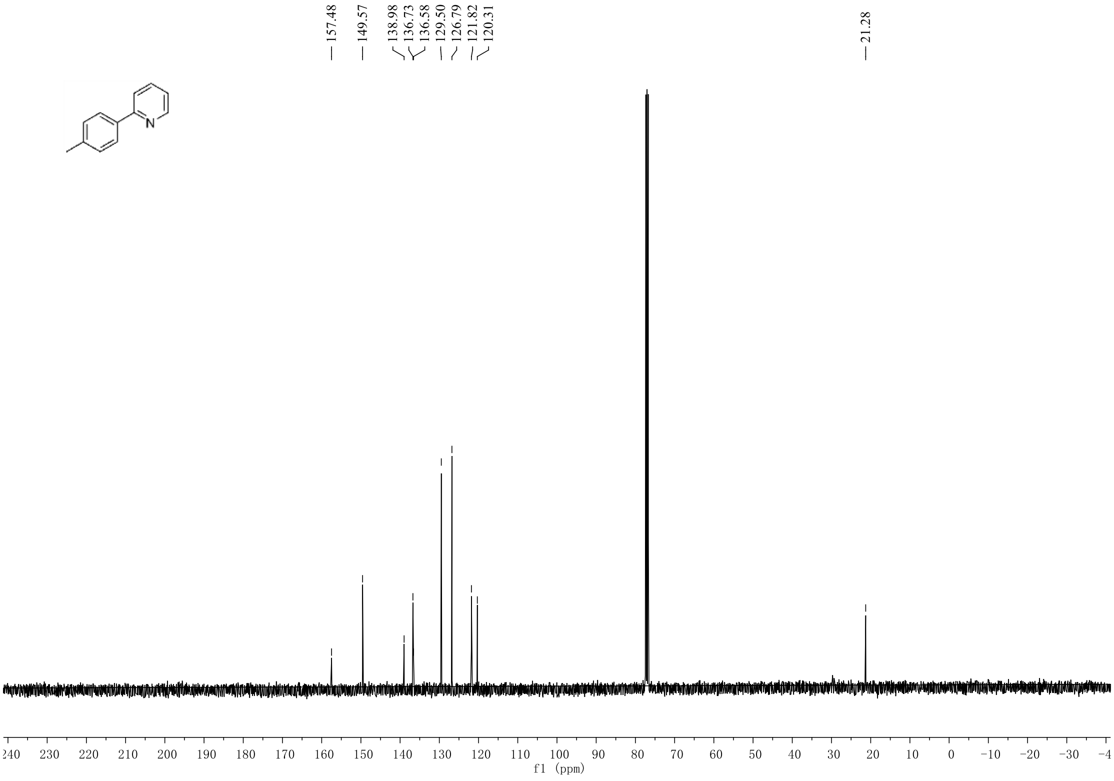
157.48
149.57
138.98
136.73
136.58
129.50
126.79
121.82
120.31
21.28
N
f1 (ppm)

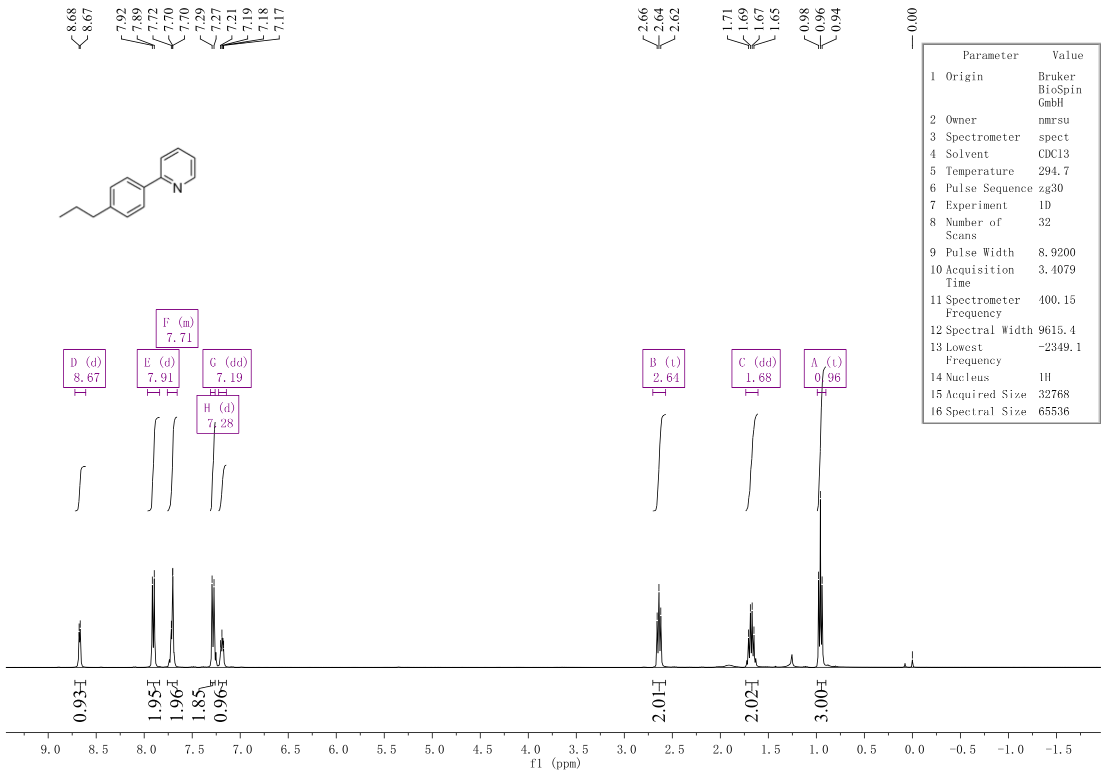

	Parameter	Value
1	Origin	Bruker BioSpin GmbH
2	Owner	nmrsu
3	Spectrometer	spect
4	Solvent	CDCl3
5	Temperature	294.7
6	Pulse Sequence	zg30
7	Experiment	1D
8	Number of Scans	32
9	Pulse Width	8.9200
10	Acquisition Time	3.4079
11	Spectrometer Frequency	400.15
12	Spectral Width	9615.4
13	Lowest Frequency	-2349.1
14	Nucleus	1H
15	Acquired Size	32768
16	Spectral Size	65536

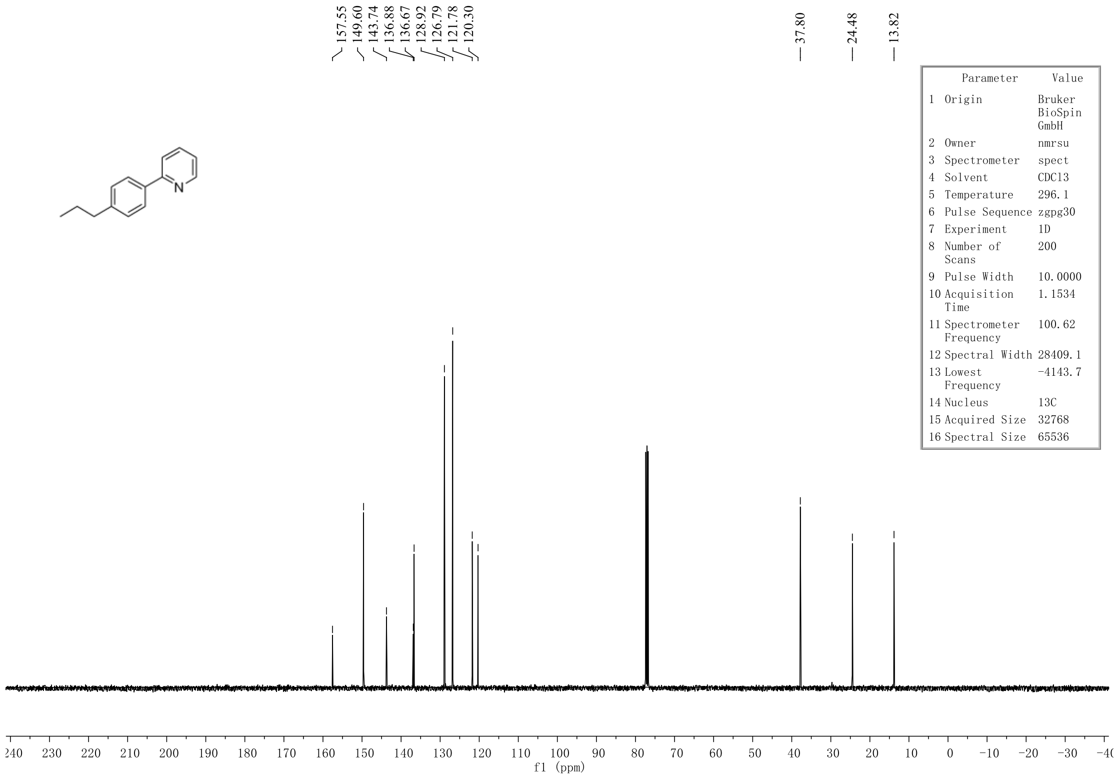

	Parameter	Value
1	Origin	Bruker BioSpin GmbH
2	Owner	nmrsu
3	Spectrometer	spect
4	Solvent	CDCl3
5	Temperature	296.1
6	Pulse Sequence	zgpg30
7	Experiment	1D
8	Number of Scans	200
9	Pulse Width	10.0000
10	Acquisition Time	1.1534
11	Spectrometer Frequency	100.62
12	Spectral Width	28409.1
13	Lowest Frequency	-4143.7
14	Nucleus	13C
15	Acquired Size	32768
16	Spectral Size	65536

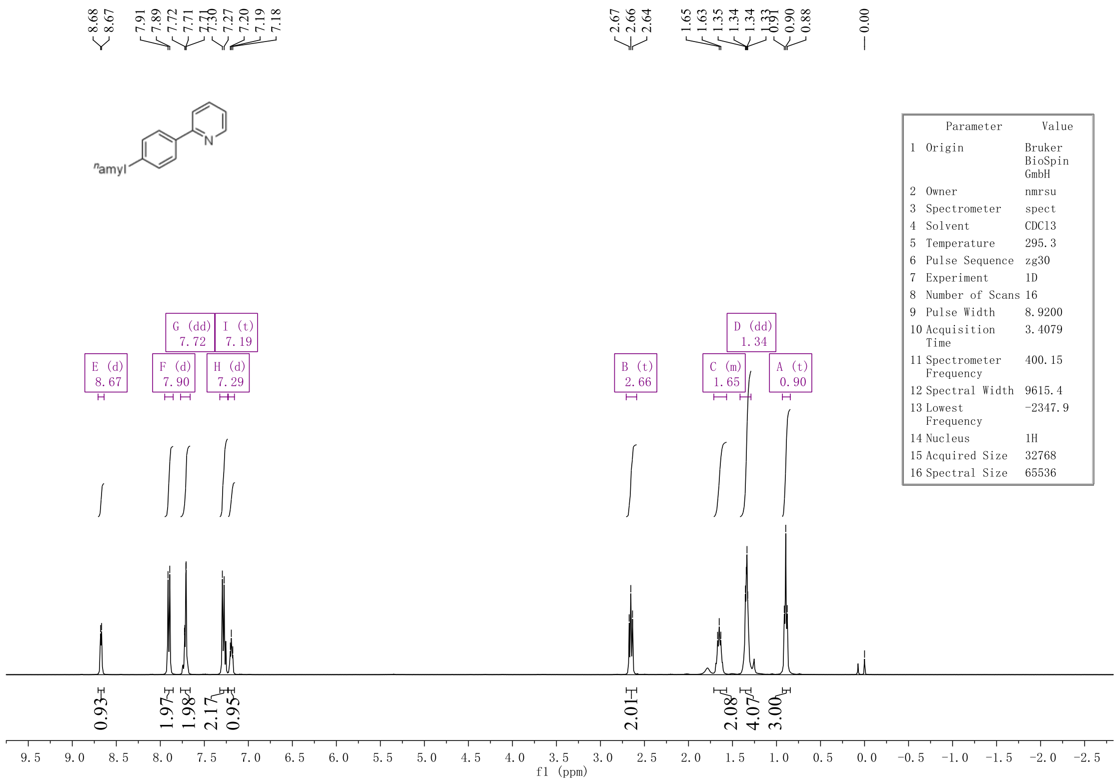

	Parameter	Value
1	Origin	Bruker BioSpin GmbH
2	Owner	nmrsu
3	Spectrometer	spect
4	Solvent	CDCl3
5	Temperature	295.3
6	Pulse Sequence	zg30
7	Experiment	1D
8	Number of Scans	16
9	Pulse Width	8.9200
10	Acquisition Time	3.4079
11	Spectrometer Frequency	400.15
12	Spectral Width	9615.4
13	Lowest Frequency	-2347.9
14	Nucleus	1H
15	Acquired Size	32768
16	Spectral Size	65536

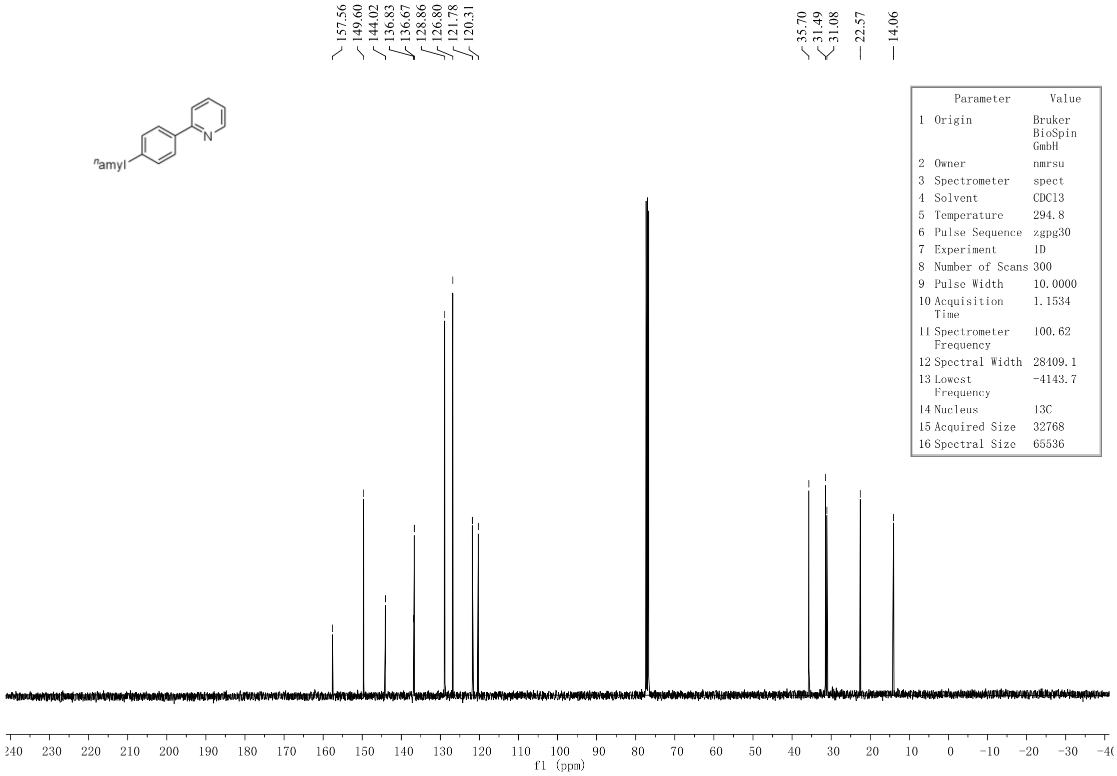

	Parameter	Value
1	Origin	Bruker BioSpin GmbH
2	Owner	nmrsu
3	Spectrometer	spect
4	Solvent	CDCl3
5	Temperature	294.8
6	Pulse Sequence	zgpg30
7	Experiment	1D
8	Number of Scans	300
9	Pulse Width	10.0000
10	Acquisition Time	1.1534
11	Spectrometer Frequency	100.62
12	Spectral Width	28409.1
13	Lowest Frequency	-4143.7
14	Nucleus	13C
15	Acquired Size	32768
16	Spectral Size	65536

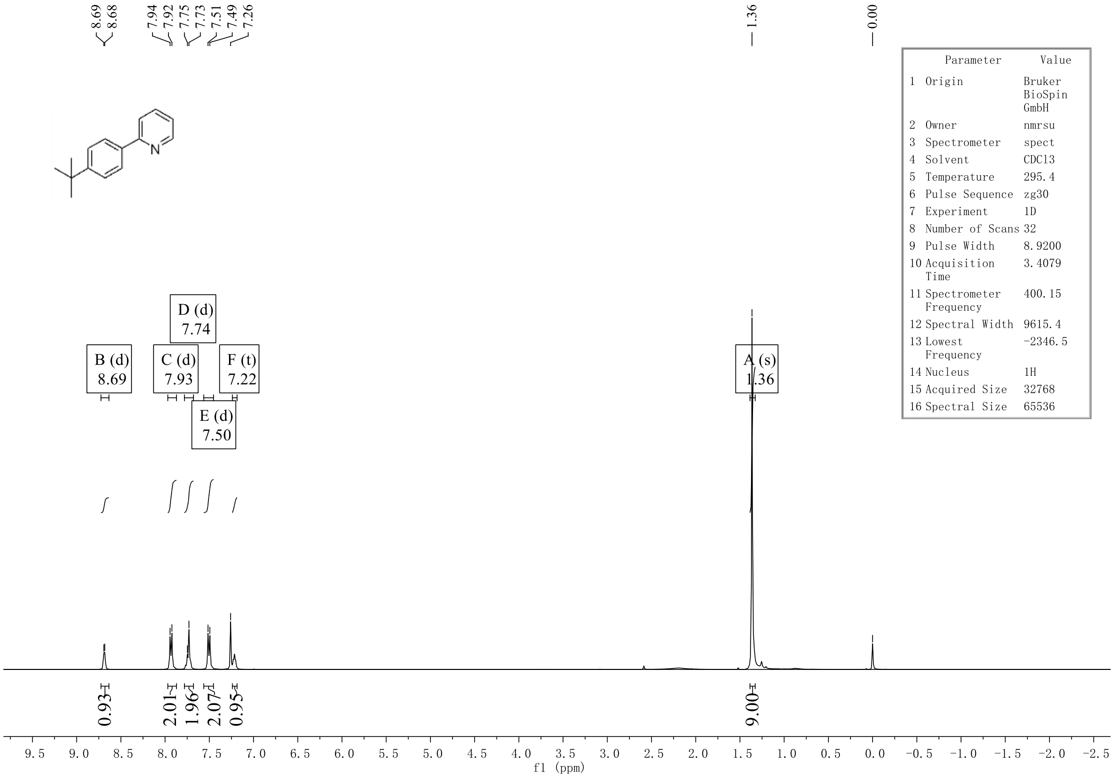

Parameter		Value
1	Origin	Bruker BioSpin GmbH
2	Owner	nmrsu
3	Spectrometer	spect
4	Solvent	CDCl3
5	Temperature	295.4
6	Pulse Sequence	zg30
7	Experiment	1D
8	Number of Scans	32
9	Pulse Width	8.9200
10	Acquisition Time	3.4079
11	Spectrometer Frequency	400.15
12	Spectral Width	9615.4
13	Lowest Frequency	-2346.5
14	Nucleus	1H
15	Acquired Size	32768
16	Spectral Size	65536

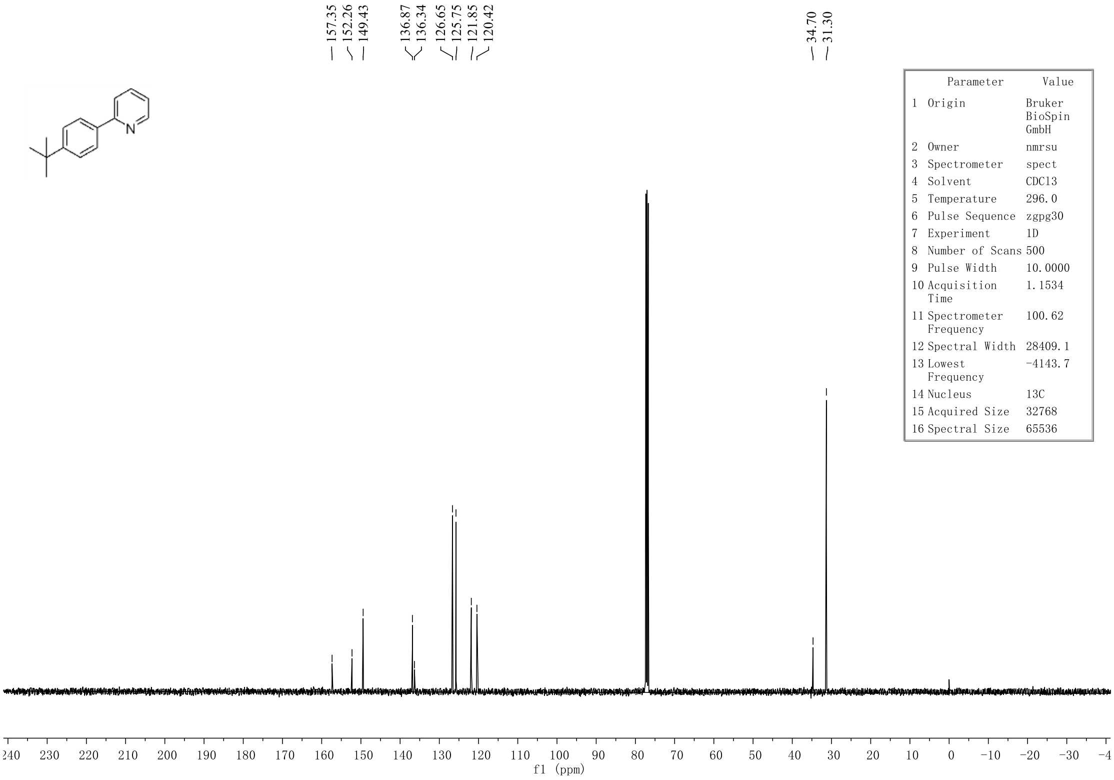

Parameter		Value
1	Origin	Bruker BioSpin GmbH
2	Owner	nmrsu
3	Spectrometer	spect
4	Solvent	CDCl3
5	Temperature	296.0
6	Pulse Sequence	zgpg30
7	Experiment	1D
8	Number of Scans	500
9	Pulse Width	10.0000
10	Acquisition Time	1.1534
11	Spectrometer Frequency	100.62
12	Spectral Width	28409.1
13	Lowest Frequency	-4143.7
14	Nucleus	13C
15	Acquired Size	32768
16	Spectral Size	65536

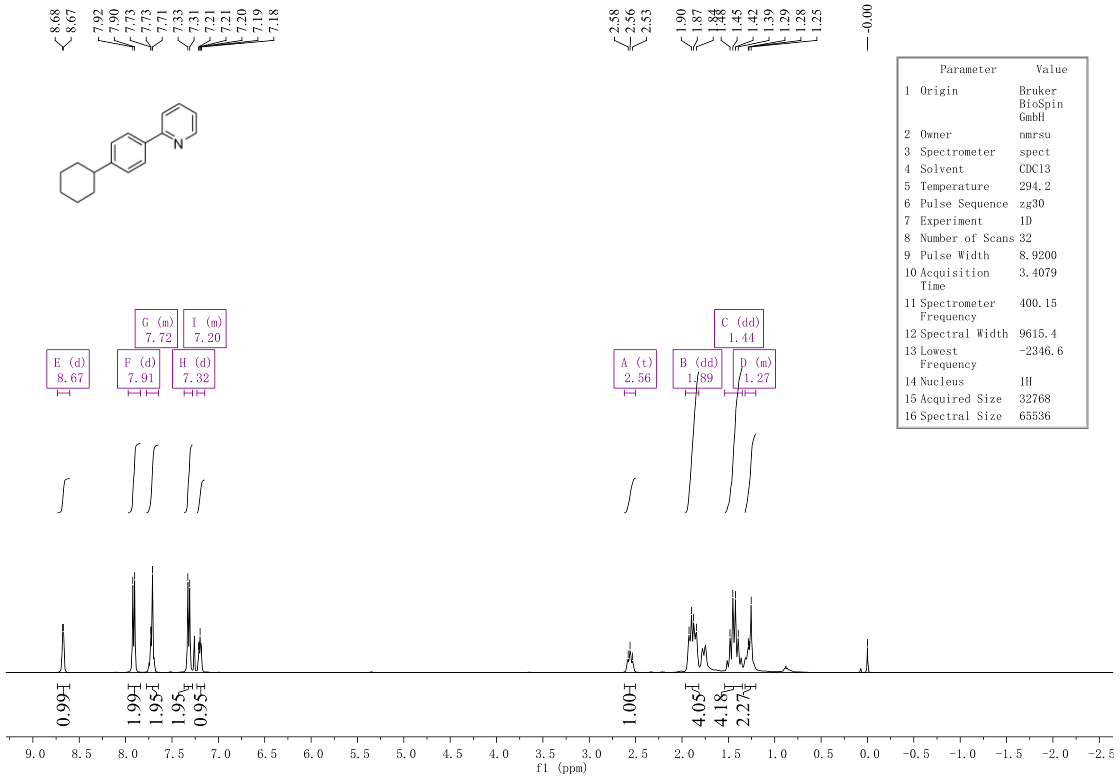

Parameter		Value
1	Origin	Bruker BioSpin GmbH
2	Owner	nmrsu
3	Spectrometer	spect
4	Solvent	CDCl3
5	Temperature	294.2
6	Pulse Sequence	zg30
7	Experiment	1D
8	Number of Scans	32
9	Pulse Width	8.9200
10	Acquisition Time	3.4079
11	Spectrometer Frequency	400.15
12	Spectral Width	9615.4
13	Lowest Frequency	-2346.6
14	Nucleus	1H
15	Acquired Size	32768
16	Spectral Size	65536

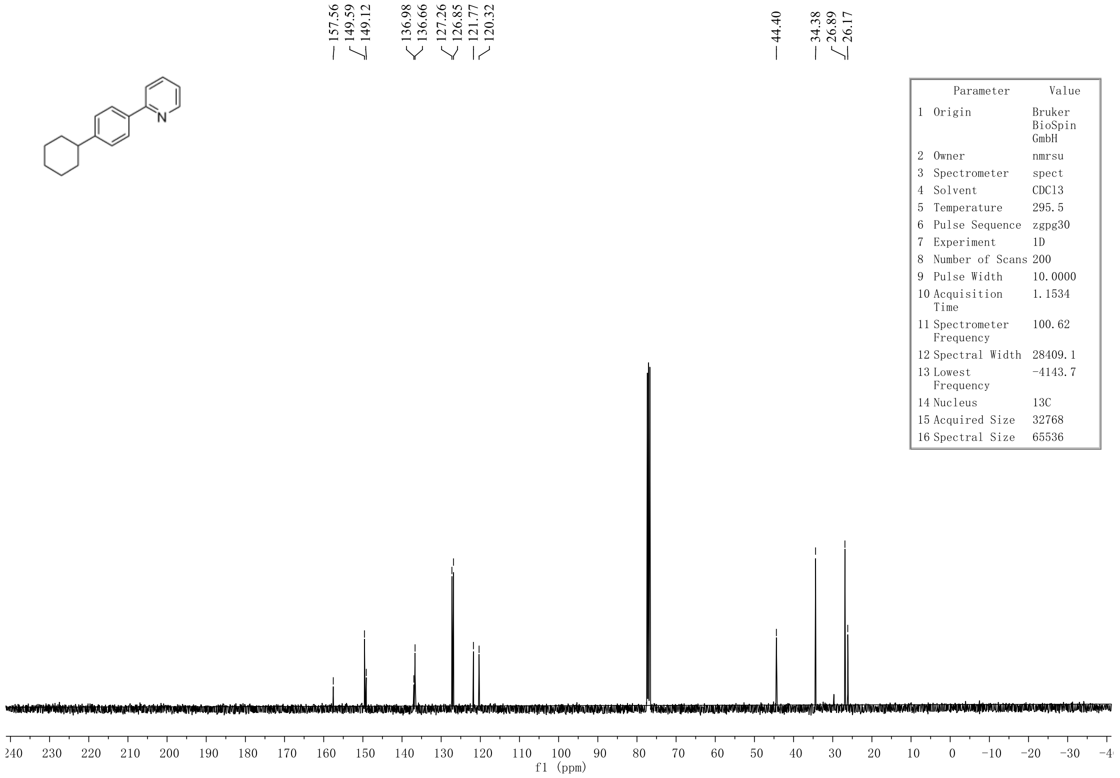

Parameter		Value
1	Origin	Bruker BioSpin GmbH
2	Owner	nmrsu
3	Spectrometer	spect
4	Solvent	CDCl3
5	Temperature	295.5
6	Pulse Sequence	zgpg30
7	Experiment	1D
8	Number of Scans	200
9	Pulse Width	10.0000
10	Acquisition Time	1.1534
11	Spectrometer Frequency	100.62
12	Spectral Width	28409.1
13	Lowest Frequency	-4143.7
14	Nucleus	13C
15	Acquired Size	32768
16	Spectral Size	65536

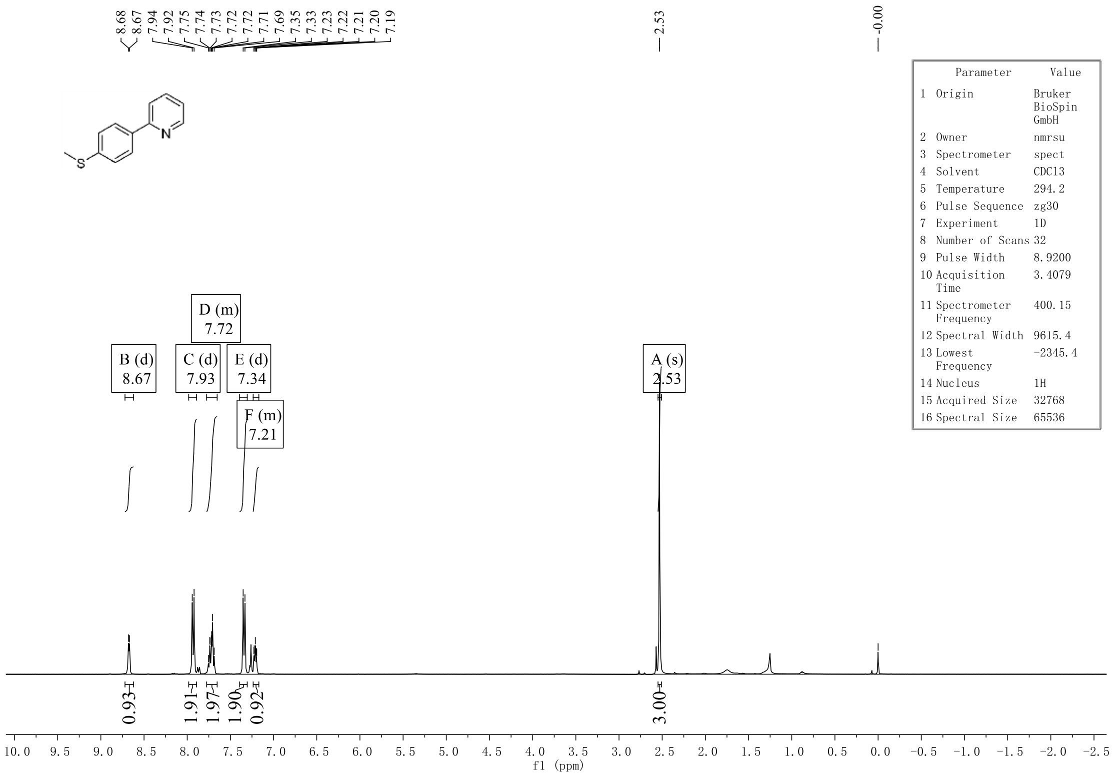

Parameter		Value
1	Origin	Bruker BioSpin GmbH
2	Owner	nmrsu
3	Spectrometer	spect
4	Solvent	CDCl3
5	Temperature	294.2
6	Pulse Sequence	zg30
7	Experiment	1D
8	Number of Scans	32
9	Pulse Width	8.9200
10	Acquisition Time	3.4079
11	Spectrometer Frequency	400.15
12	Spectral Width	9615.4
13	Lowest Frequency	-2345.4
14	Nucleus	1H
15	Acquired Size	32768
16	Spectral Size	65536

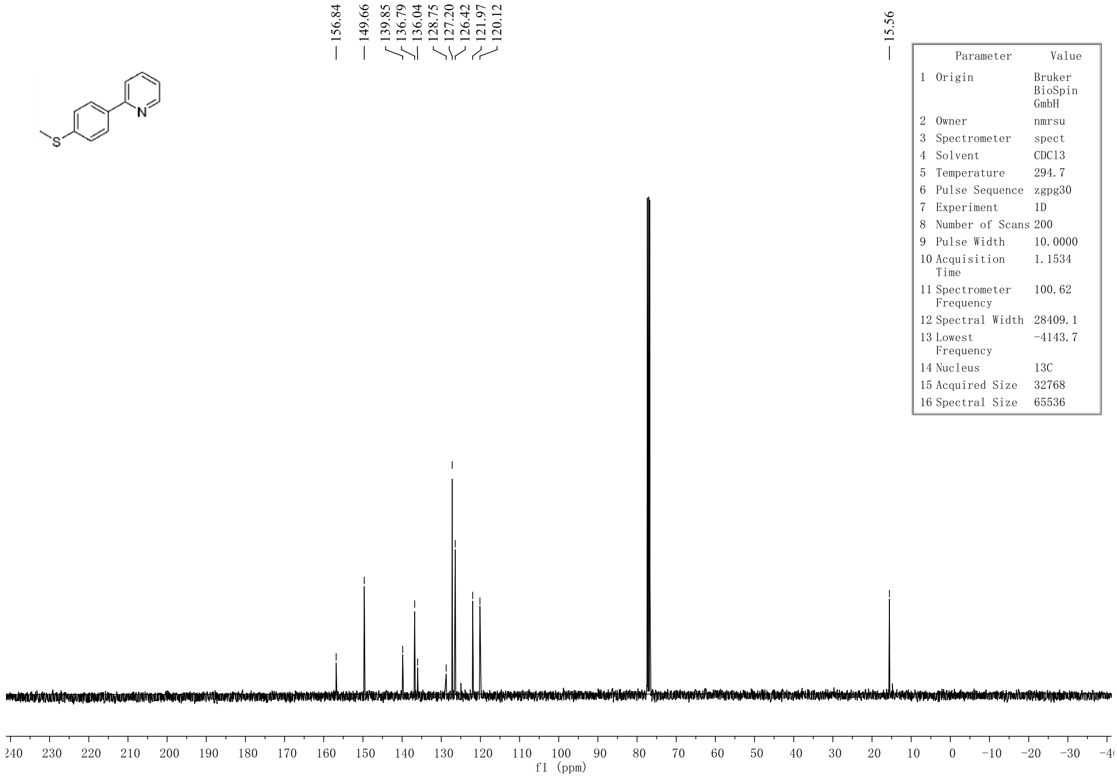

Parameter		Value
1	Origin	Bruker BioSpin GmbH
2	Owner	nmrsu
3	Spectrometer	spect
4	Solvent	CDCl3
5	Temperature	294.7
6	Pulse Sequence	zgpg30
7	Experiment	1D
8	Number of Scans	200
9	Pulse Width	10.0000
10	Acquisition Time	1.1534
11	Spectrometer Frequency	100.62
12	Spectral Width	28409.1
13	Lowest Frequency	-4143.7
14	Nucleus	13C
15	Acquired Size	32768
16	Spectral Size	65536

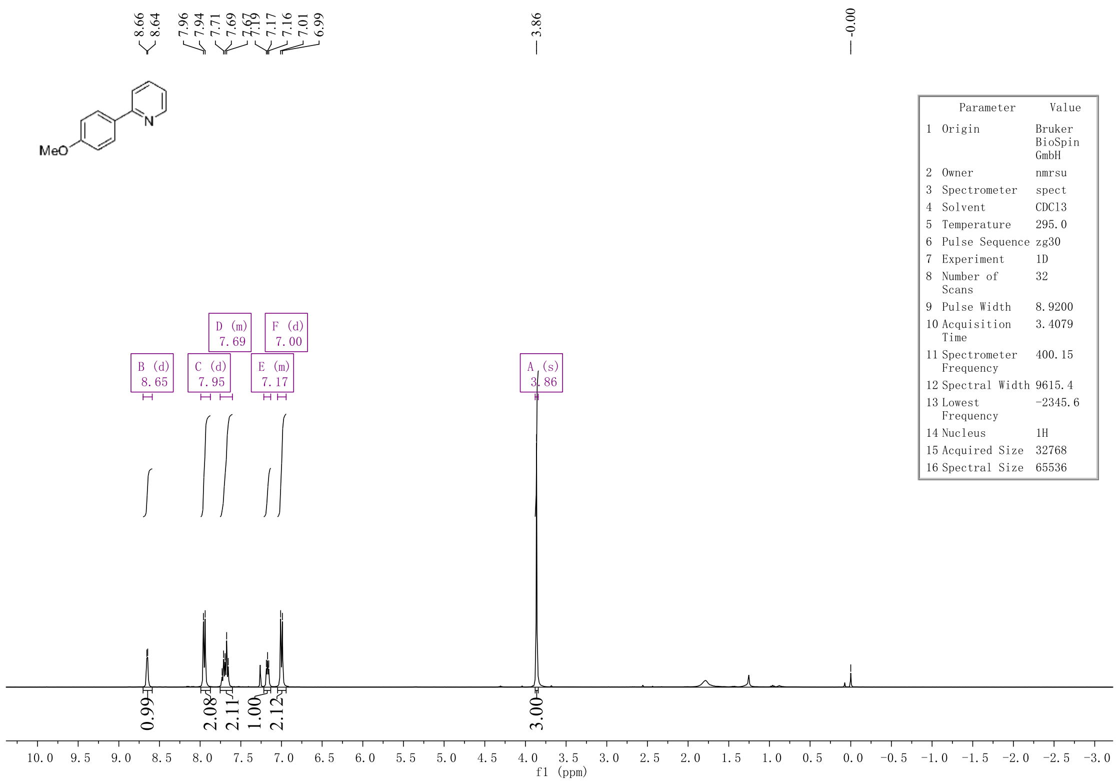

Parameter	Value
1 Origin	Bruker BioSpin GmbH
2 Owner	nmrsu
3 Spectrometer	spect
4 Solvent	CDCl3
5 Temperature	295.0
6 Pulse Sequence	zg30
7 Experiment	1D
8 Number of Scans	32
9 Pulse Width	8.9200
10 Acquisition Time	3.4079
11 Spectrometer Frequency	400.15
12 Spectral Width	9615.4
13 Lowest Frequency	-2345.6
14 Nucleus	1H
15 Acquired Size	32768
16 Spectral Size	65536

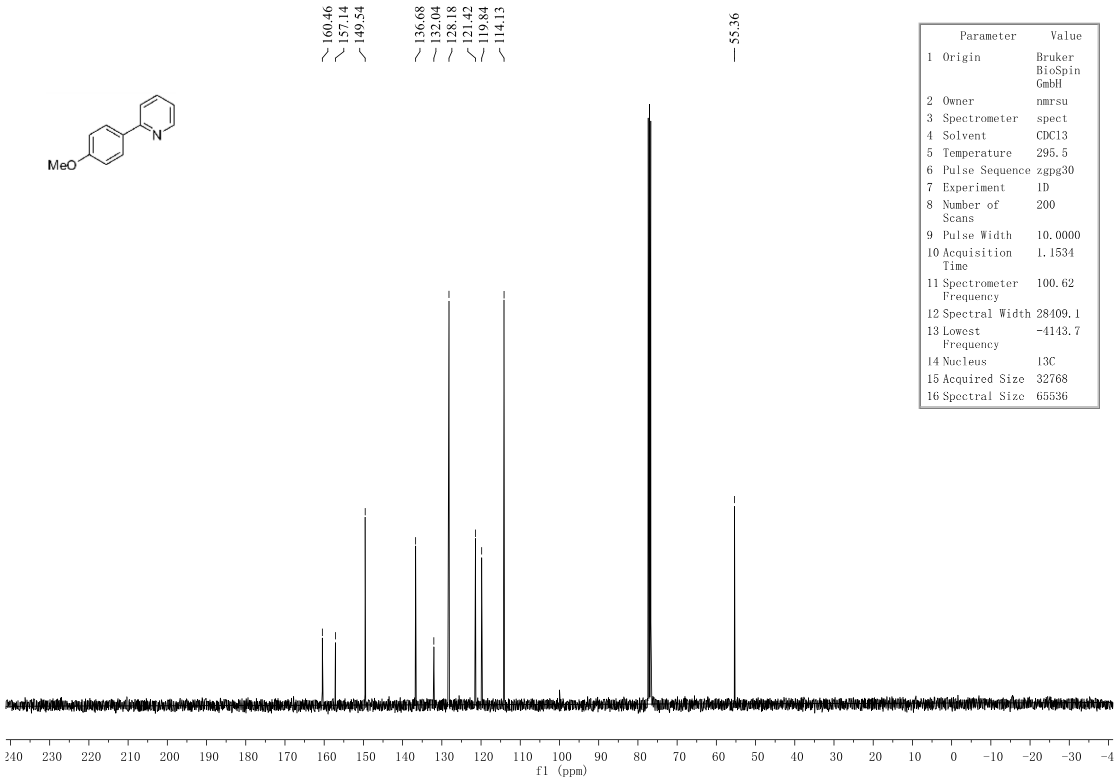

Parameter	Value
1 Origin	Bruker BioSpin GmbH
2 Owner	nmrsu
3 Spectrometer	spect
4 Solvent	CDCl3
5 Temperature	295.5
6 Pulse Sequence	zgpg30
7 Experiment	1D
8 Number of Scans	200
9 Pulse Width	10.0000
10 Acquisition Time	1.1534
11 Spectrometer Frequency	100.62
12 Spectral Width	28409.1
13 Lowest Frequency	-4143.7
14 Nucleus	13C
15 Acquired Size	32768
16 Spectral Size	65536

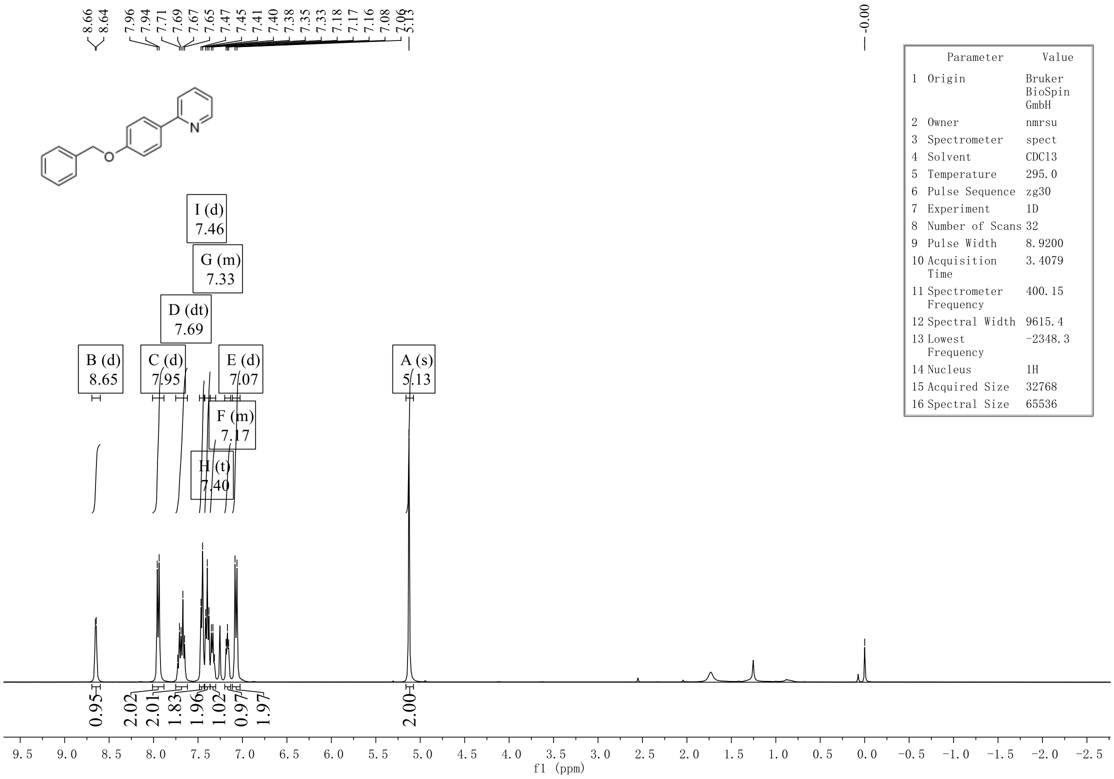

8.66
8.64
7.96
7.94
7.71
7.69
7.67
7.65
7.47
7.45
7.41
7.40
7.38
7.35
7.33
7.18
7.17
7.16
7.08
7.06
5.13
-0.00
N
O
I (d) 7.46
G (m) 7.33
D (dt) 7.69
B (d) 8.65
C (d) 7.95
E (d) 7.07
A (s) 5.13
F (m) 7.17
H (t) 7.40
0.95
2.02
2.01
1.83
1.96
1.02
0.97
1.97
2.00
f1 (ppm)
Parameter Value
1 Origin Bruker BioSpin GmbH
2 Owner nmrsu
3 Spectrometer spect
4 Solvent CDCl3
5 Temperature 295.0
6 Pulse Sequence zg30
7 Experiment 1D
8 Number of Scans 32
9 Pulse Width 8.9200
10 Acquisition Time 3.4079
11 Spectrometer Frequency 400.15
12 Spectral Width 9615.4
13 Lowest Frequency -2348.3
14 Nucleus 1H
15 Acquired Size 32768
16 Spectral Size 65536

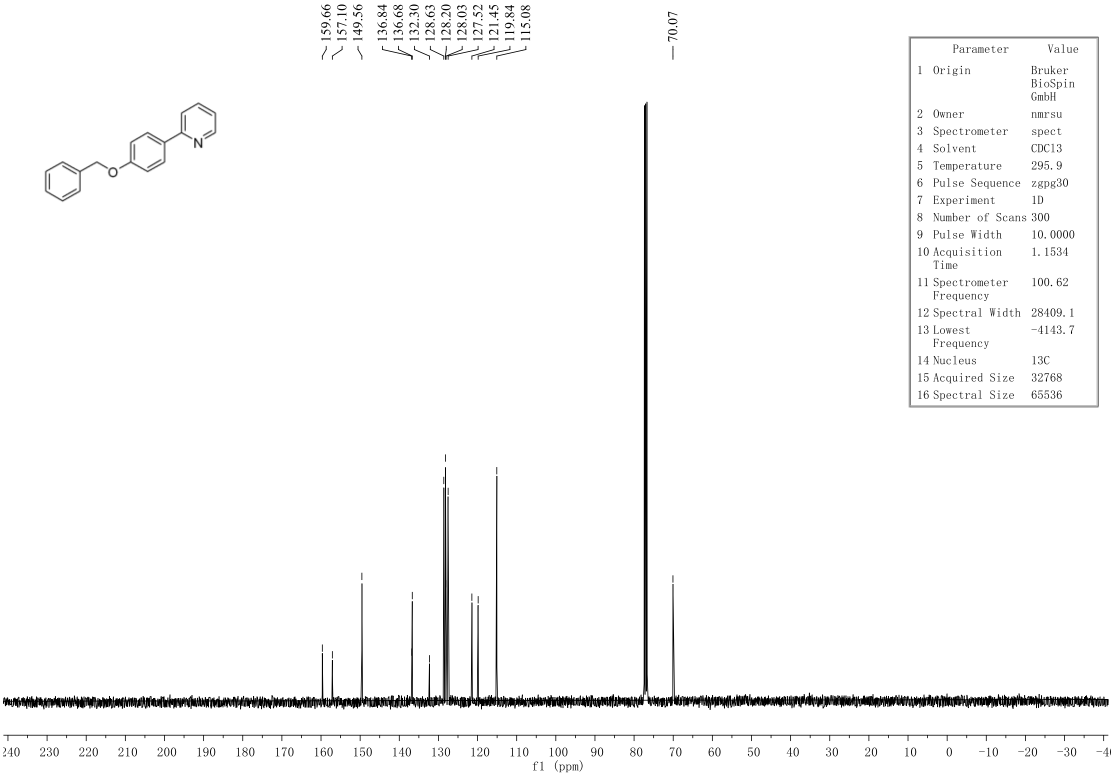

159.66
157.10
149.56
136.84
136.68
132.30
128.63
128.20
128.03
127.52
121.45
119.84
115.08
70.07
N
O
f1 (ppm)
Parameter Value
1 Origin Bruker BioSpin GmbH
2 Owner nmrsu
3 Spectrometer spect
4 Solvent CDCl3
5 Temperature 295.9
6 Pulse Sequence zgpg30
7 Experiment 1D
8 Number of Scans 300
9 Pulse Width 10.0000
10 Acquisition Time 1.1534
11 Spectrometer Frequency 100.62
12 Spectral Width 28409.1
13 Lowest Frequency -4143.7
14 Nucleus 13C
15 Acquired Size 32768
16 Spectral Size 65536

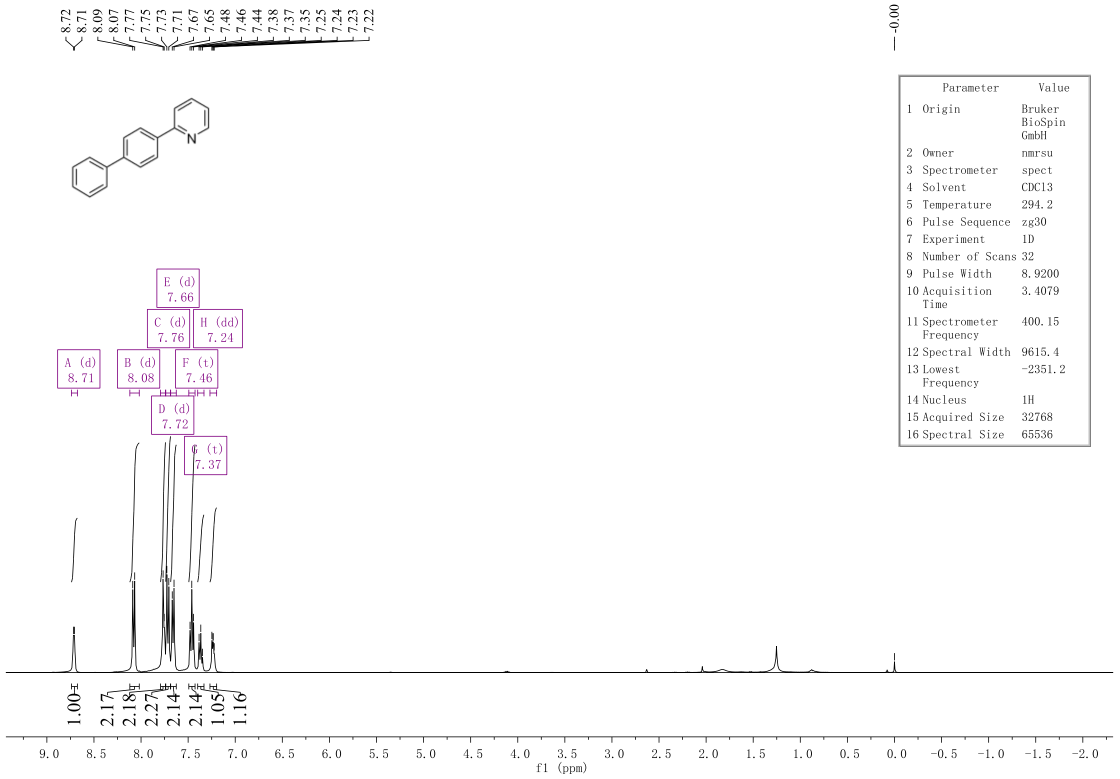

	Parameter	Value
1	Origin	Bruker BioSpin GmbH
2	Owner	nmrsu
3	Spectrometer	spect
4	Solvent	CDCl3
5	Temperature	294.2
6	Pulse Sequence	zg30
7	Experiment	1D
8	Number of Scans	32
9	Pulse Width	8.9200
10	Acquisition Time	3.4079
11	Spectrometer Frequency	400.15
12	Spectral Width	9615.4
13	Lowest Frequency	-2351.2
14	Nucleus	1H
15	Acquired Size	32768
16	Spectral Size	65536

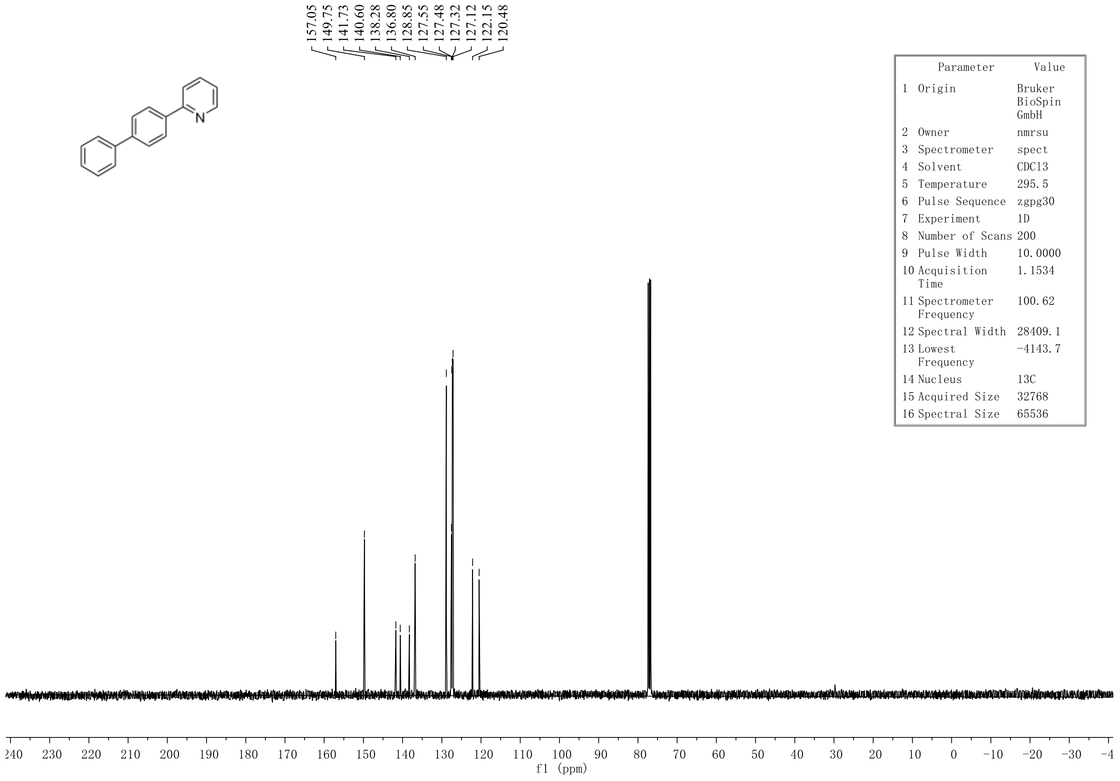

	Parameter	Value
1	Origin	Bruker BioSpin GmbH
2	Owner	nmrsu
3	Spectrometer	spect
4	Solvent	CDCl3
5	Temperature	295.5
6	Pulse Sequence	zgpg30
7	Experiment	1D
8	Number of Scans	200
9	Pulse Width	10.0000
10	Acquisition Time	1.1534
11	Spectrometer Frequency	100.62
12	Spectral Width	28409.1
13	Lowest Frequency	-4143.7
14	Nucleus	13C
15	Acquired Size	32768
16	Spectral Size	65536

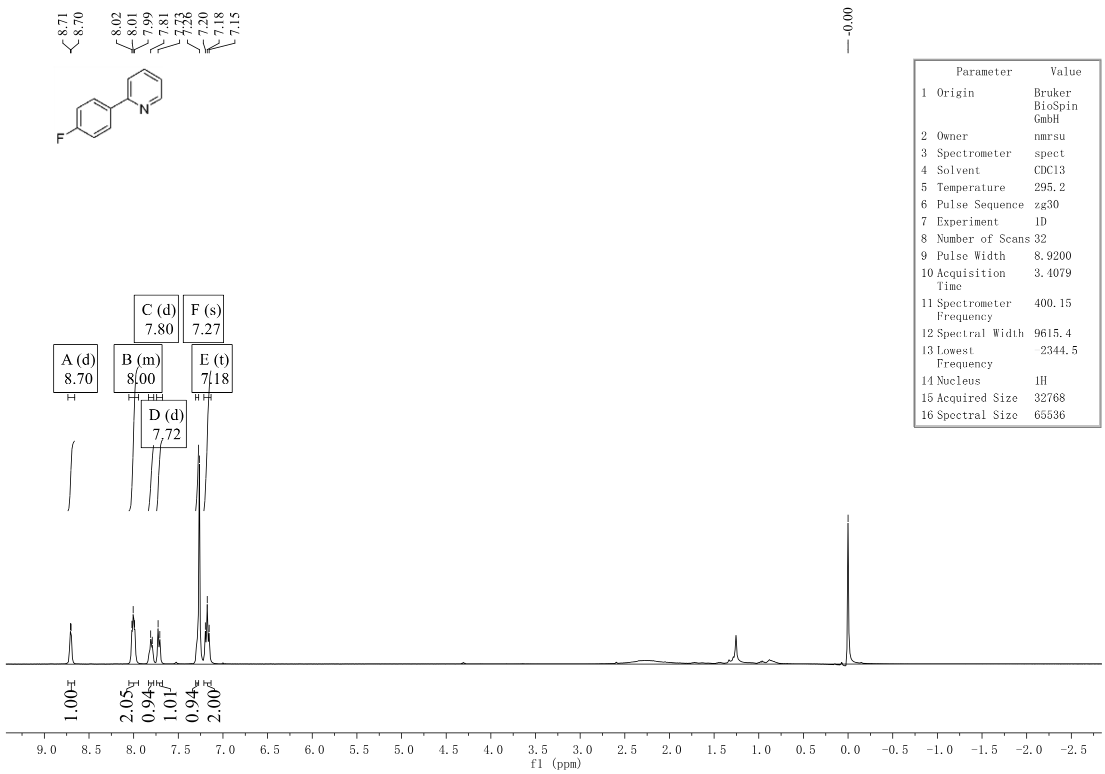

Parameter	Value
1 Origin	Bruker BioSpin GmbH
2 Owner	nmrsu
3 Spectrometer	spect
4 Solvent	CDCl3
5 Temperature	295.2
6 Pulse Sequence	zg30
7 Experiment	1D
8 Number of Scans	32
9 Pulse Width	8.9200
10 Acquisition Time	3.4079
11 Spectrometer Frequency	400.15
12 Spectral Width	9615.4
13 Lowest Frequency	-2344.5
14 Nucleus	1H
15 Acquired Size	32768
16 Spectral Size	65536

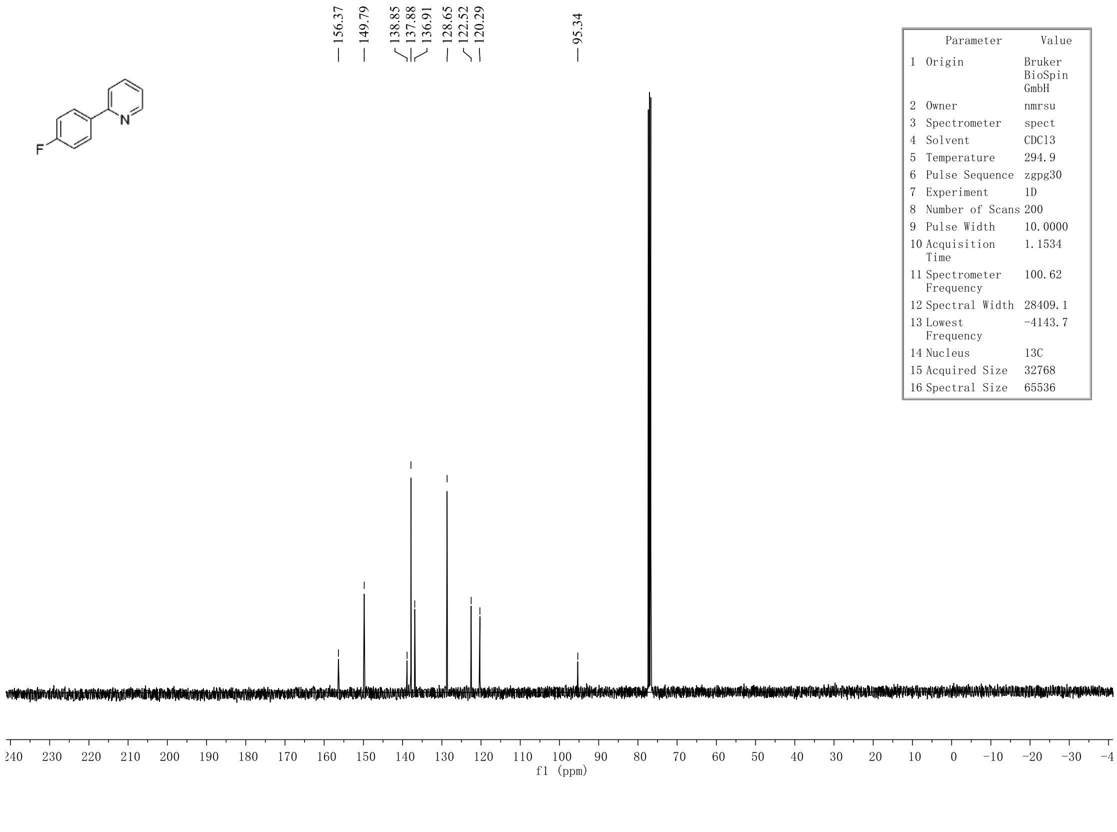

Parameter	Value
1 Origin	Bruker BioSpin GmbH
2 Owner	nmrsu
3 Spectrometer	spect
4 Solvent	CDCl3
5 Temperature	294.9
6 Pulse Sequence	zgpg30
7 Experiment	1D
8 Number of Scans	200
9 Pulse Width	10.0000
10 Acquisition Time	1.1534
11 Spectrometer Frequency	100.62
12 Spectral Width	28409.1
13 Lowest Frequency	-4143.7
14 Nucleus	13C
15 Acquired Size	32768
16 Spectral Size	65536

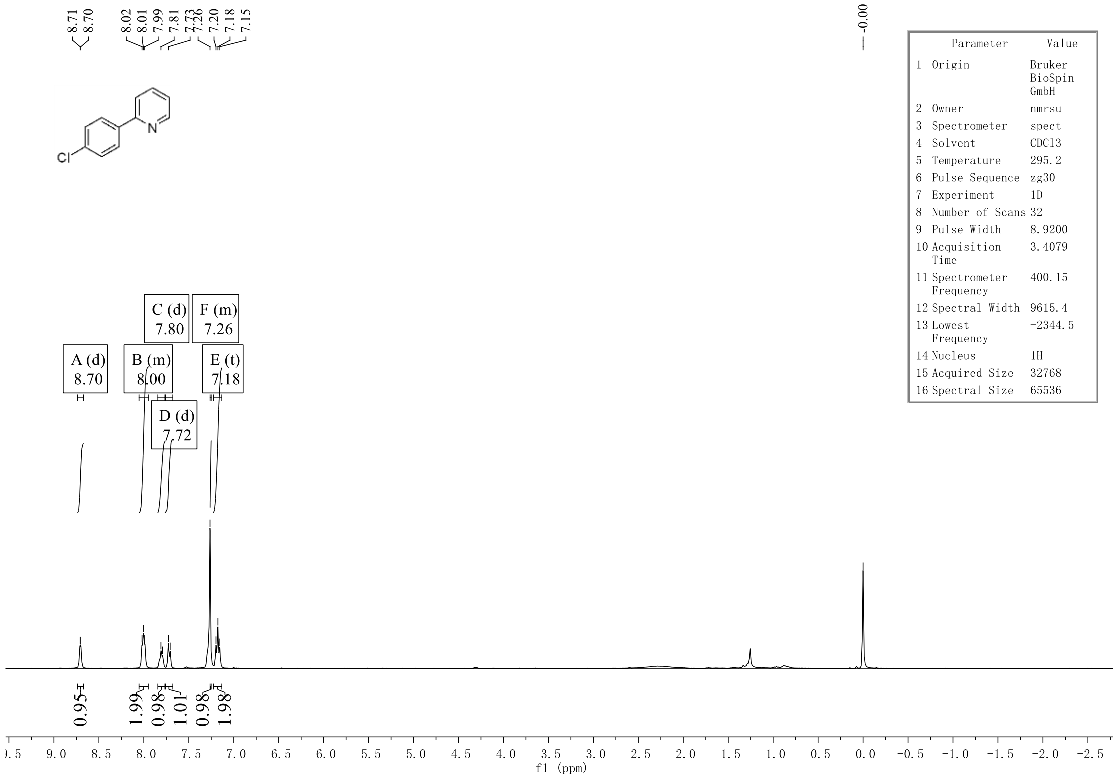

Parameter		Value
1	Origin	Bruker BioSpin GmbH
2	Owner	nmrsu
3	Spectrometer	spect
4	Solvent	CDCl3
5	Temperature	295.2
6	Pulse Sequence	zg30
7	Experiment	1D
8	Number of Scans	32
9	Pulse Width	8.9200
10	Acquisition Time	3.4079
11	Spectrometer Frequency	400.15
12	Spectral Width	9615.4
13	Lowest Frequency	-2344.5
14	Nucleus	1H
15	Acquired Size	32768
16	Spectral Size	65536

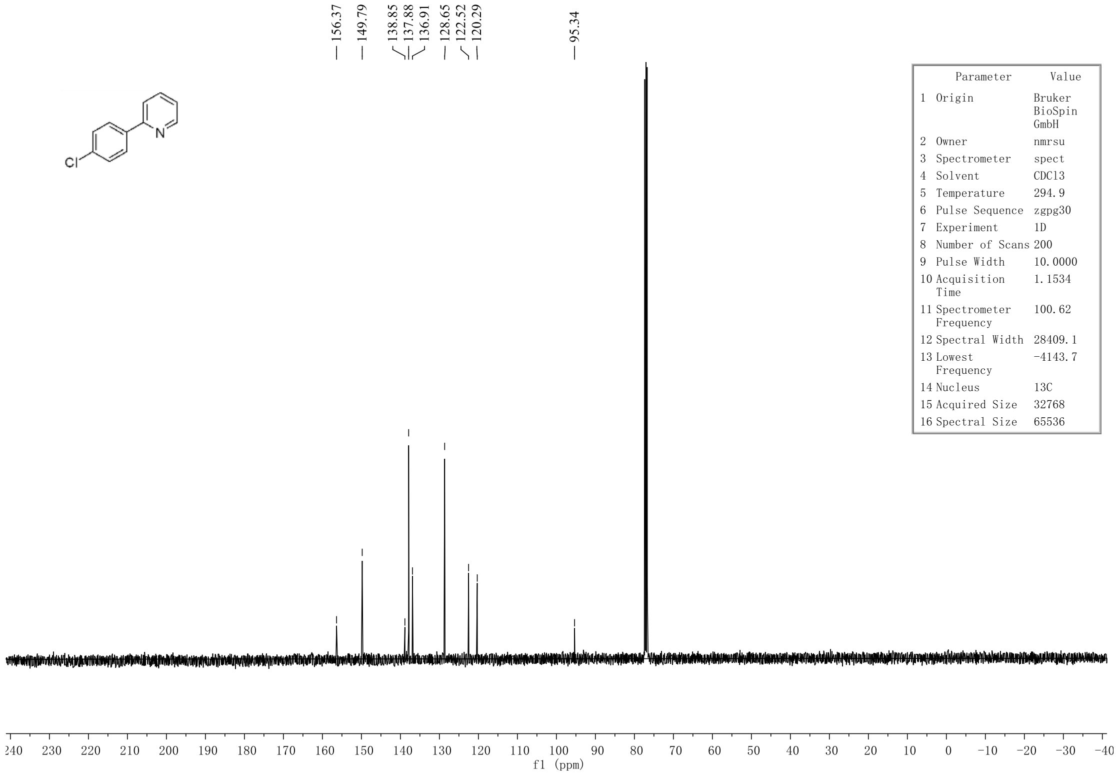

Parameter		Value
1	Origin	Bruker BioSpin GmbH
2	Owner	nmrsu
3	Spectrometer	spect
4	Solvent	CDCl3
5	Temperature	294.9
6	Pulse Sequence	zgpg30
7	Experiment	1D
8	Number of Scans	200
9	Pulse Width	10.0000
10	Acquisition Time	1.1534
11	Spectrometer Frequency	100.62
12	Spectral Width	28409.1
13	Lowest Frequency	-4143.7
14	Nucleus	13C
15	Acquired Size	32768
16	Spectral Size	65536

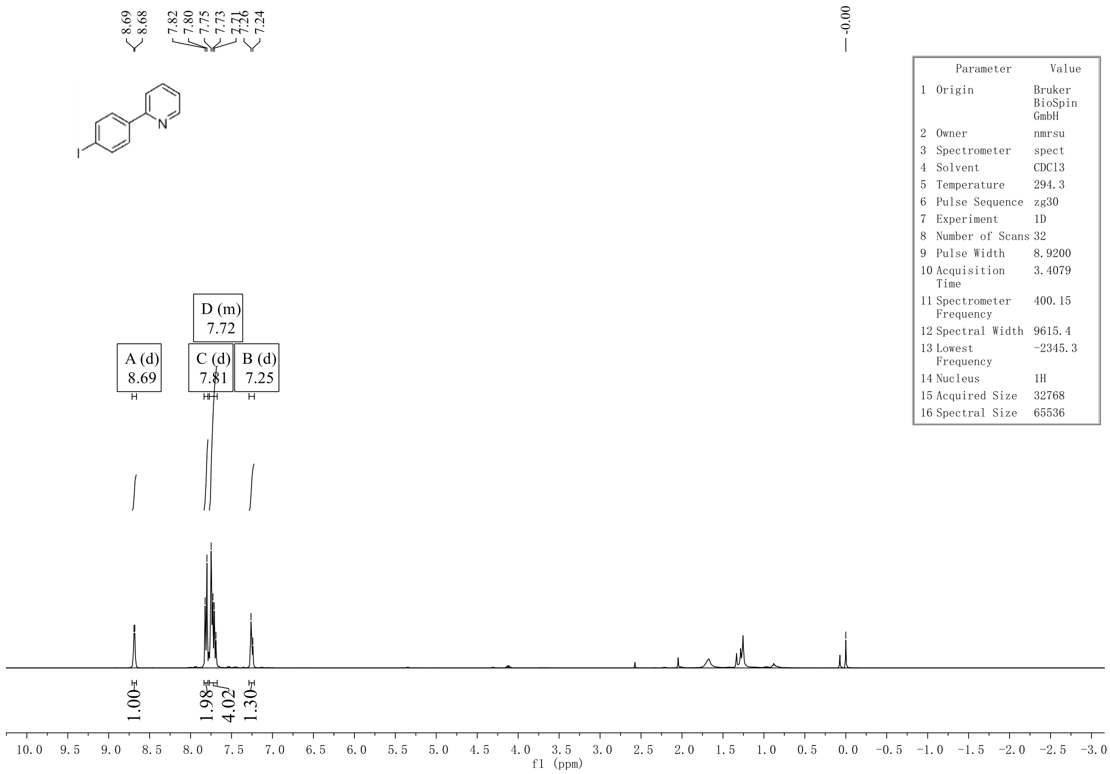

	Parameter	Value
1	Origin	Bruker BioSpin GmbH
2	Owner	nmrsu
3	Spectrometer	spect
4	Solvent	CDCl3
5	Temperature	294.3
6	Pulse Sequence	zg30
7	Experiment	1D
8	Number of Scans	32
9	Pulse Width	8.9200
10	Acquisition Time	3.4079
11	Spectrometer Frequency	400.15
12	Spectral Width	9615.4
13	Lowest Frequency	-2345.3
14	Nucleus	1H
15	Acquired Size	32768
16	Spectral Size	65536

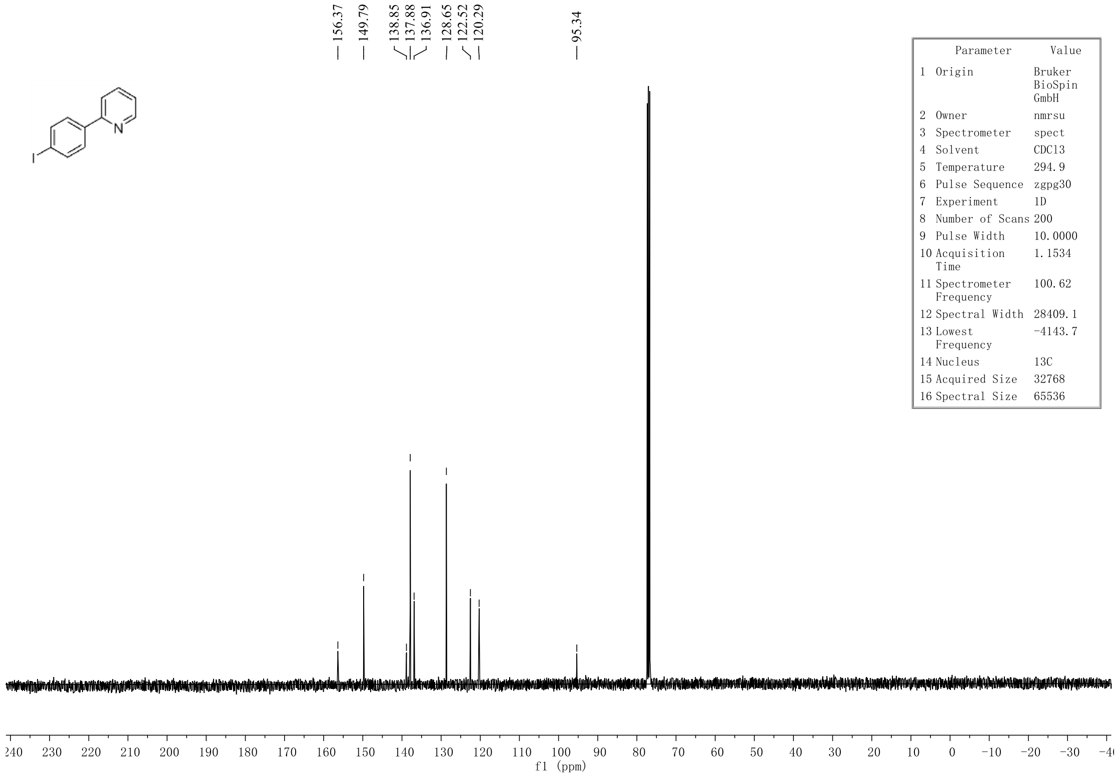

	Parameter	Value
1	Origin	Bruker BioSpin GmbH
2	Owner	nmrsu
3	Spectrometer	spect
4	Solvent	CDCl3
5	Temperature	294.9
6	Pulse Sequence	zgpg30
7	Experiment	1D
8	Number of Scans	200
9	Pulse Width	10.0000
10	Acquisition Time	1.1534
11	Spectrometer Frequency	100.62
12	Spectral Width	28409.1
13	Lowest Frequency	-4143.7
14	Nucleus	13C
15	Acquired Size	32768
16	Spectral Size	65536

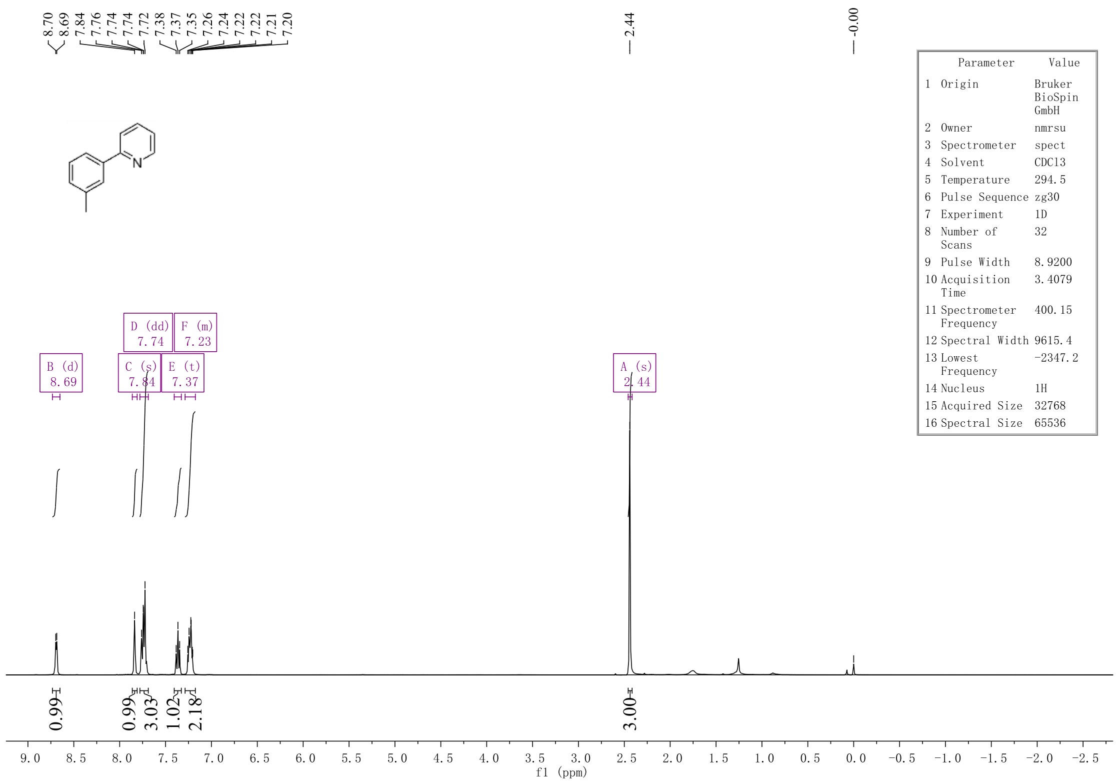

Parameter		Value
1	Origin	Bruker BioSpin GmbH
2	Owner	nmrsu
3	Spectrometer	spect
4	Solvent	CDCl3
5	Temperature	294.5
6	Pulse Sequence	zg30
7	Experiment	1D
8	Number of Scans	32
9	Pulse Width	8.9200
10	Acquisition Time	3.4079
11	Spectrometer Frequency	400.15
12	Spectral Width	9615.4
13	Lowest Frequency	-2347.2
14	Nucleus	1H
15	Acquired Size	32768
16	Spectral Size	65536

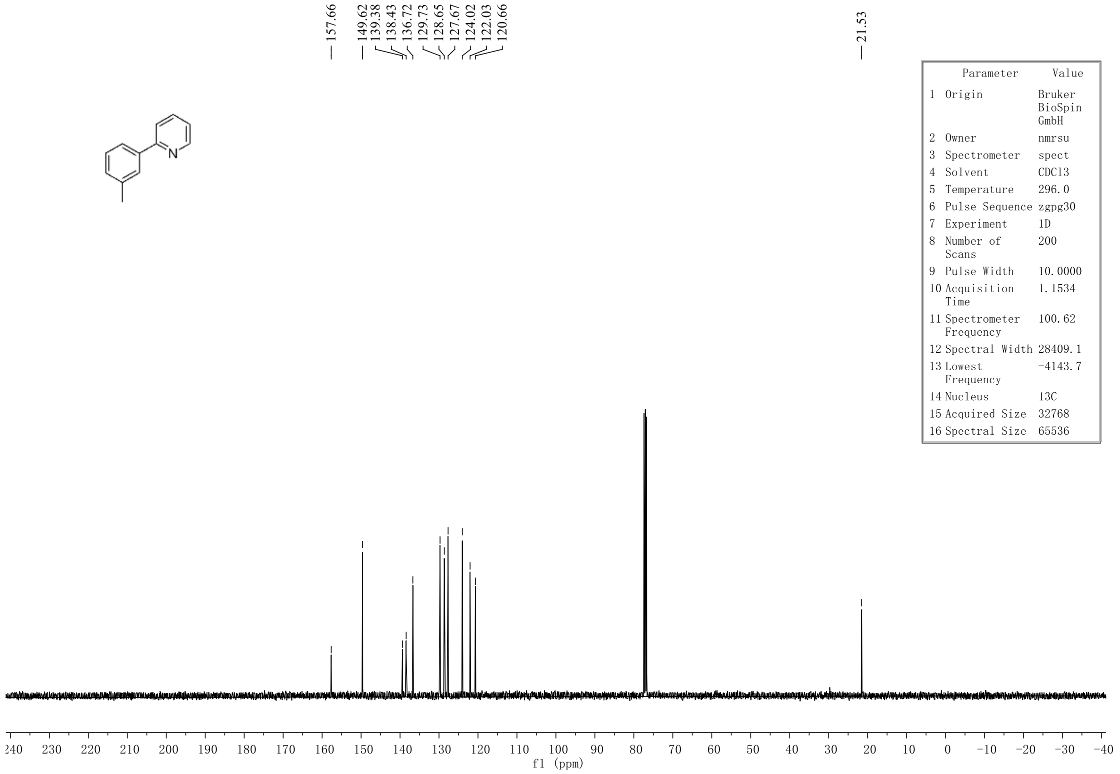

Parameter		Value
1	Origin	Bruker BioSpin GmbH
2	Owner	nmrsu
3	Spectrometer	spect
4	Solvent	CDCl3
5	Temperature	296.0
6	Pulse Sequence	zgpg30
7	Experiment	1D
8	Number of Scans	200
9	Pulse Width	10.0000
10	Acquisition Time	1.1534
11	Spectrometer Frequency	100.62
12	Spectral Width	28409.1
13	Lowest Frequency	-4143.7
14	Nucleus	13C
15	Acquired Size	32768
16	Spectral Size	65536

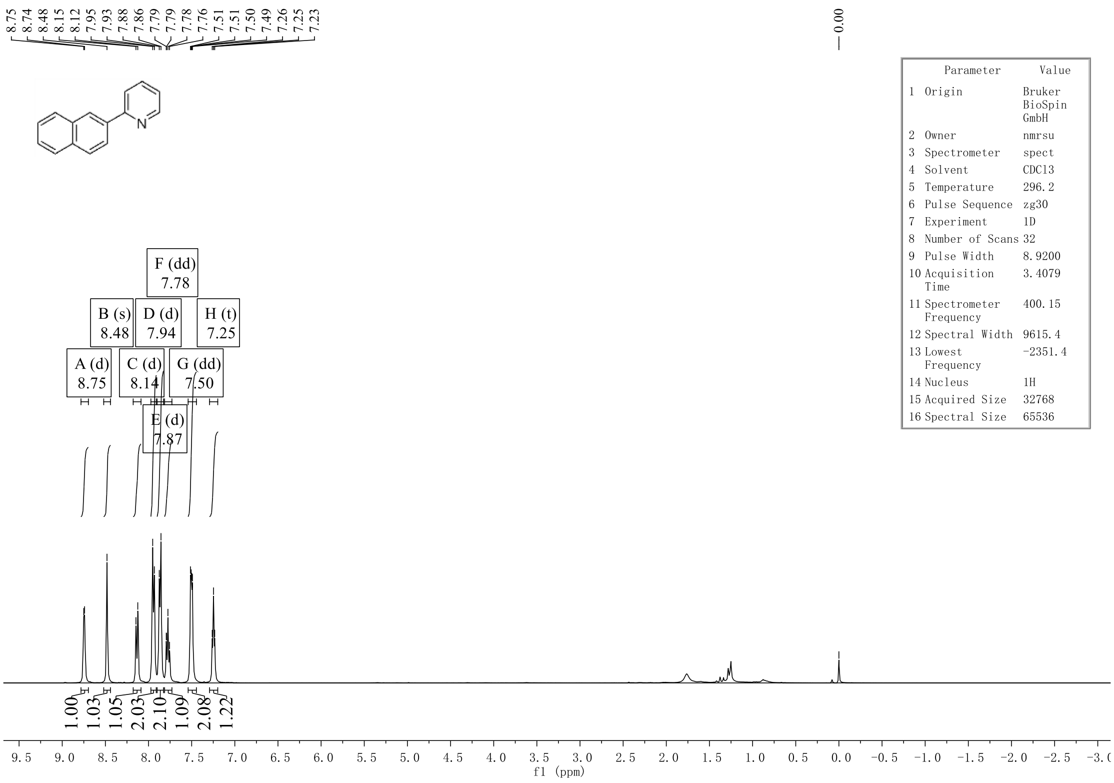

	Parameter	Value
1	Origin	Bruker BioSpin GmbH
2	Owner	nmrsu
3	Spectrometer	spect
4	Solvent	CDCl3
5	Temperature	296.2
6	Pulse Sequence	zg30
7	Experiment	1D
8	Number of Scans	32
9	Pulse Width	8.9200
10	Acquisition Time	3.4079
11	Spectrometer Frequency	400.15
12	Spectral Width	9615.4
13	Lowest Frequency	-2351.4
14	Nucleus	1H
15	Acquired Size	32768
16	Spectral Size	65536

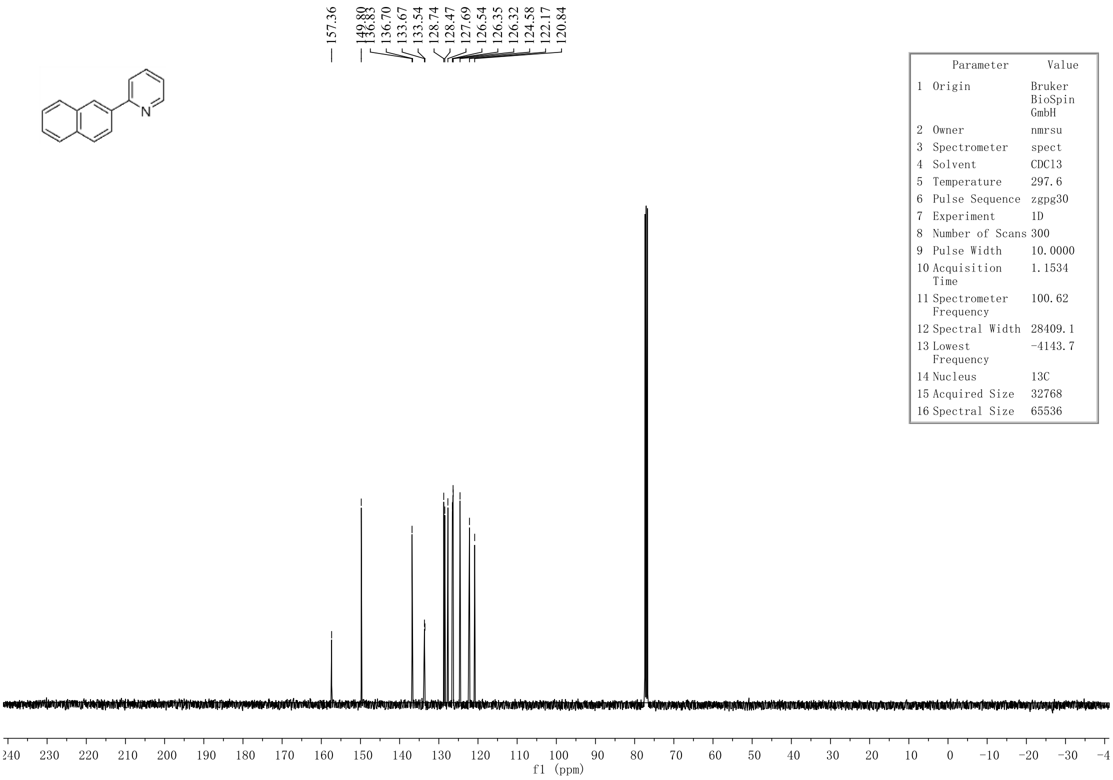

	Parameter	Value
1	Origin	Bruker BioSpin GmbH
2	Owner	nmrsu
3	Spectrometer	spect
4	Solvent	CDCl3
5	Temperature	297.6
6	Pulse Sequence	zgpg30
7	Experiment	1D
8	Number of Scans	300
9	Pulse Width	10.0000
10	Acquisition Time	1.1534
11	Spectrometer Frequency	100.62
12	Spectral Width	28409.1
13	Lowest Frequency	-4143.7
14	Nucleus	13C
15	Acquired Size	32768
16	Spectral Size	65536

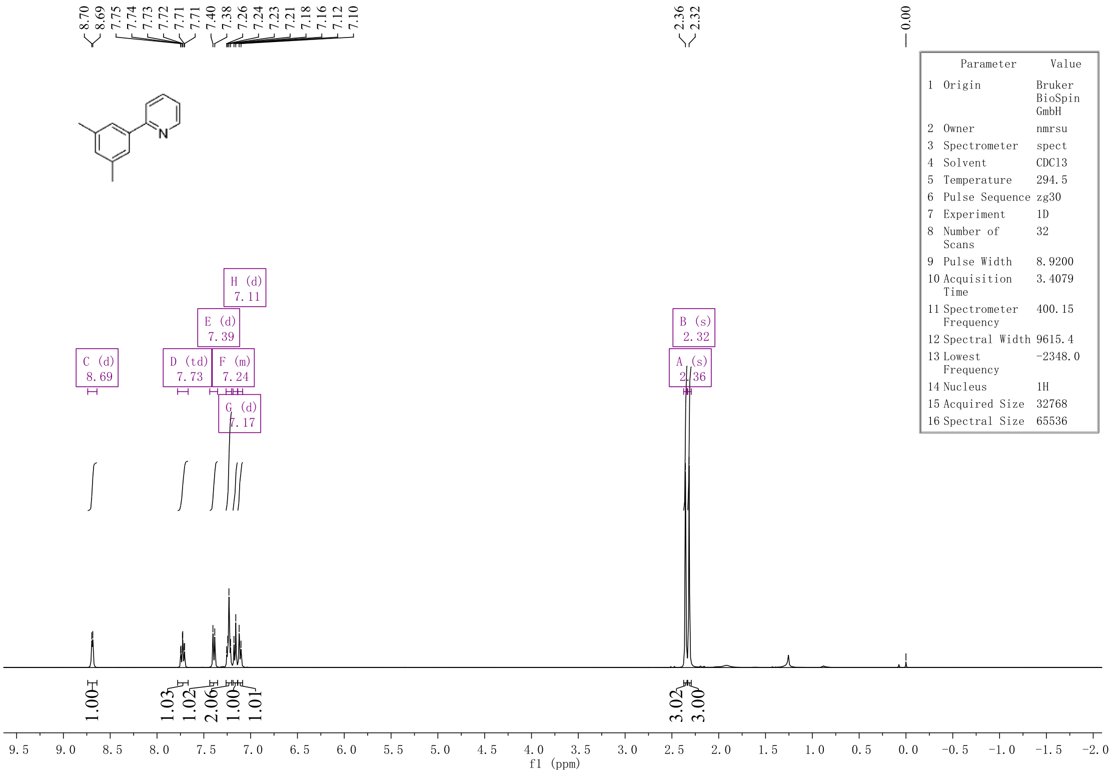

	Parameter	Value
1	Origin	Bruker BioSpin GmbH
2	Owner	nmrsu
3	Spectrometer	spect
4	Solvent	CDCl3
5	Temperature	294.5
6	Pulse Sequence	zg30
7	Experiment	1D
8	Number of Scans	32
9	Pulse Width	8.9200
10	Acquisition Time	3.4079
11	Spectrometer Frequency	400.15
12	Spectral Width	9615.4
13	Lowest Frequency	-2348.0
14	Nucleus	1H
15	Acquired Size	32768
16	Spectral Size	65536

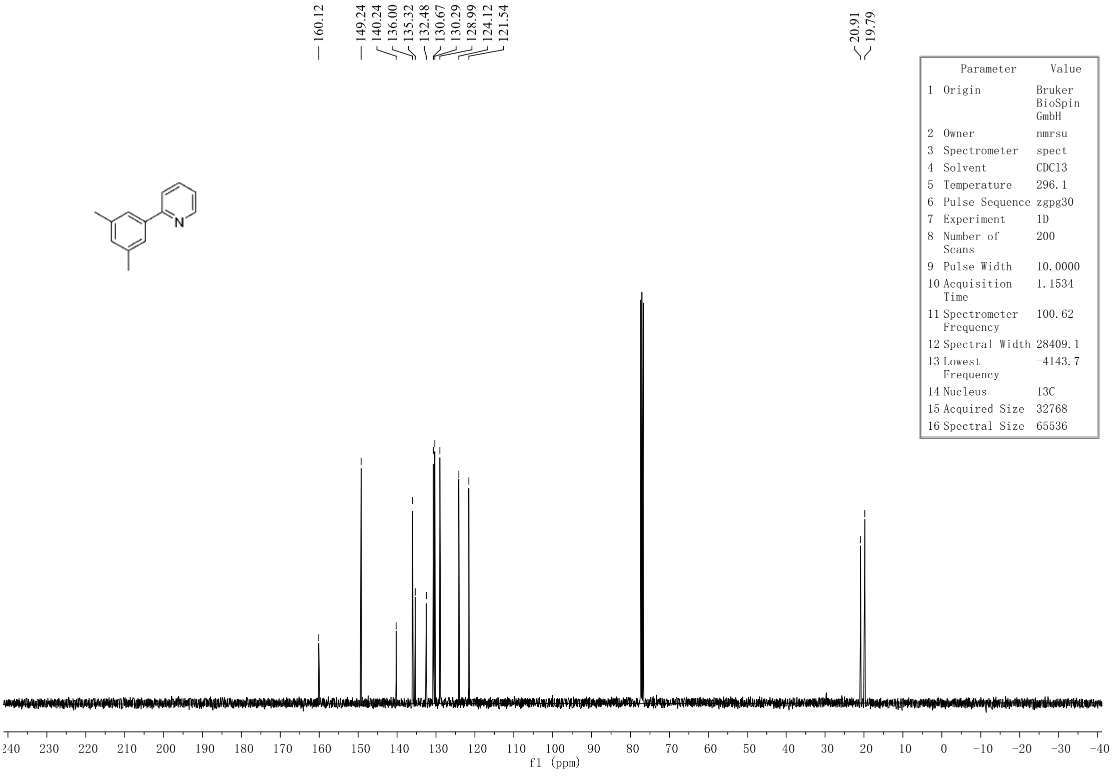

	Parameter	Value
1	Origin	Bruker BioSpin GmbH
2	Owner	nmrsu
3	Spectrometer	spect
4	Solvent	CDCl3
5	Temperature	296.1
6	Pulse Sequence	zgpg30
7	Experiment	1D
8	Number of Scans	200
9	Pulse Width	10.0000
10	Acquisition Time	1.1534
11	Spectrometer Frequency	100.62
12	Spectral Width	28409.1
13	Lowest Frequency	-4143.7
14	Nucleus	13C
15	Acquired Size	32768
16	Spectral Size	65536

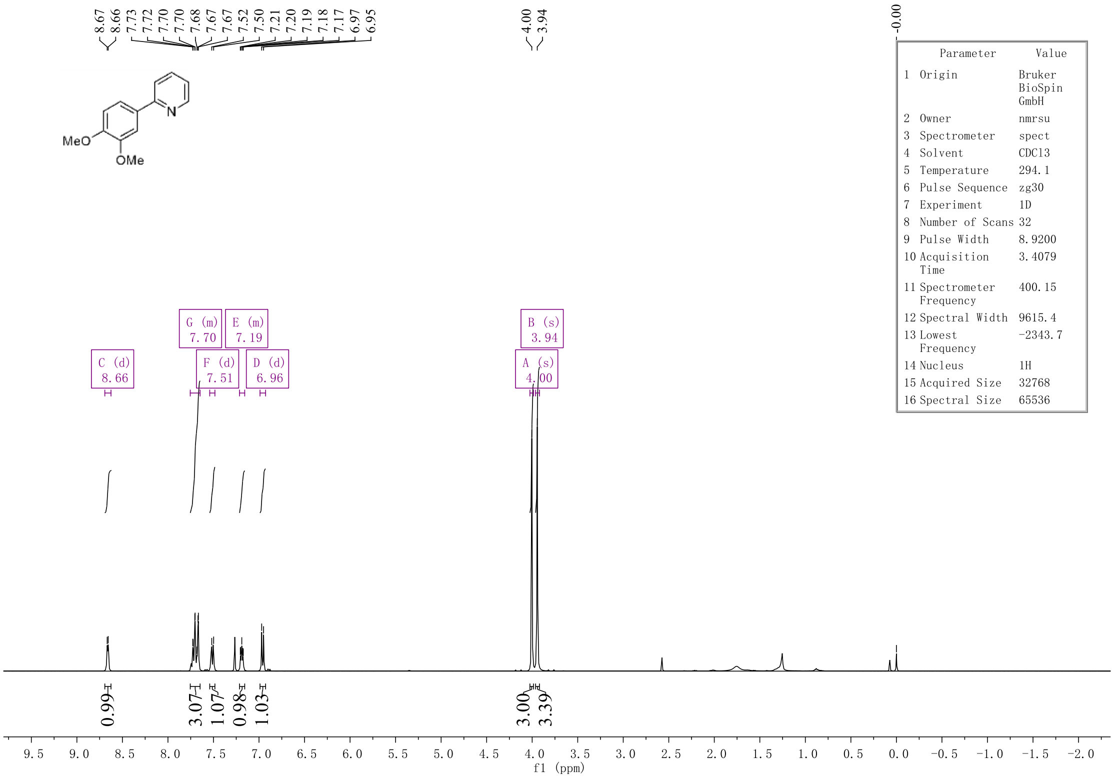

Parameter		Value
1	Origin	Bruker BioSpin GmbH
2	Owner	nmrsu
3	Spectrometer	spect
4	Solvent	CDCl3
5	Temperature	294.1
6	Pulse Sequence	zg30
7	Experiment	1D
8	Number of Scans	32
9	Pulse Width	8.9200
10	Acquisition Time	3.4079
11	Spectrometer Frequency	400.15
12	Spectral Width	9615.4
13	Lowest Frequency	-2343.7
14	Nucleus	1H
15	Acquired Size	32768
16	Spectral Size	65536

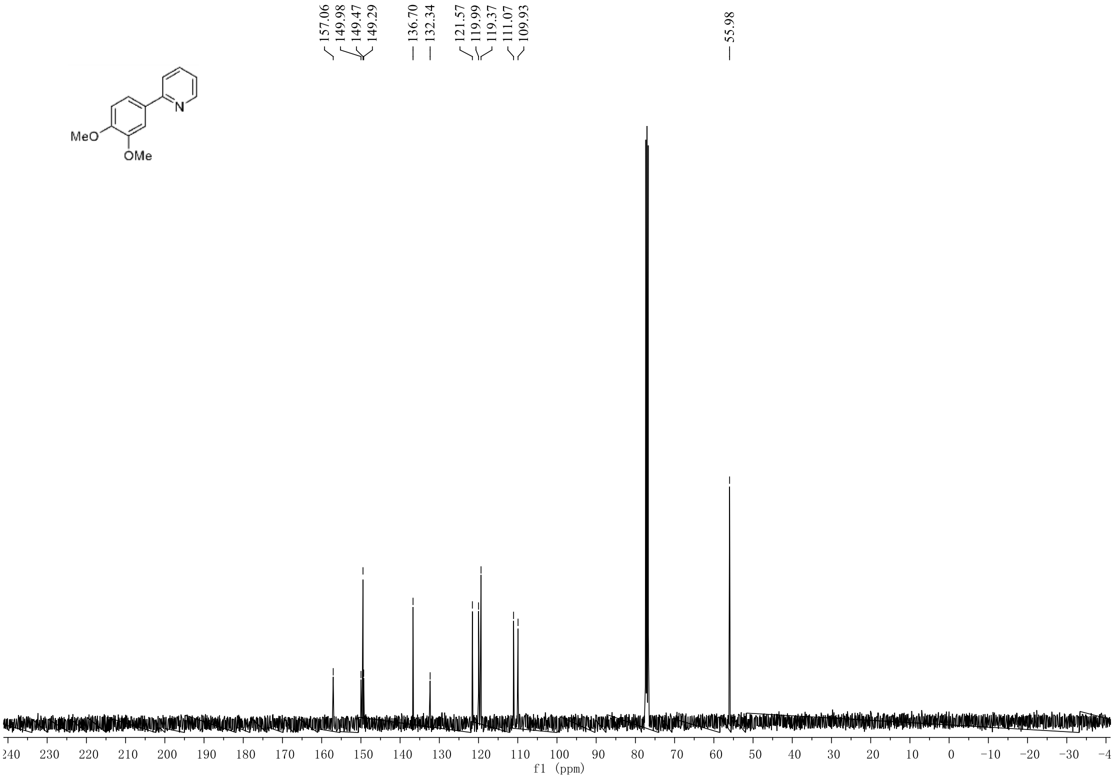

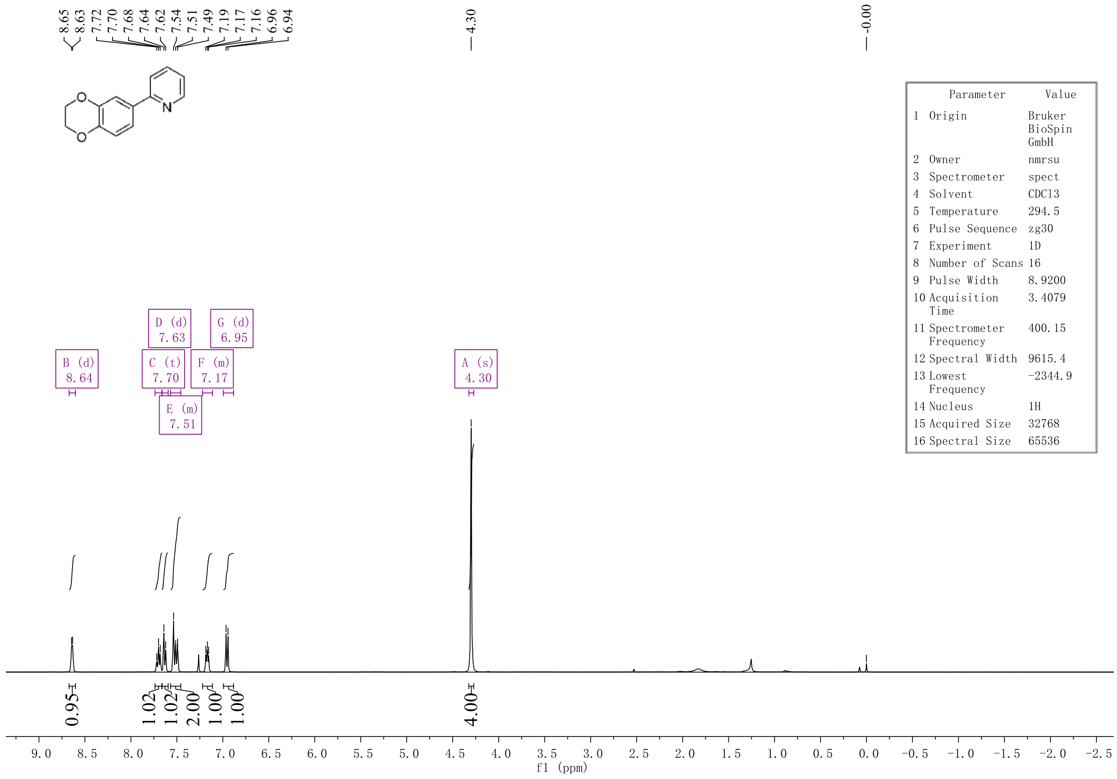

Parameter		Value
1	Origin	Bruker BioSpin GmbH
2	Owner	nmrsu
3	Spectrometer	spect
4	Solvent	CDCl3
5	Temperature	294.5
6	Pulse Sequence	zg30
7	Experiment	1D
8	Number of Scans	16
9	Pulse Width	8.9200
10	Acquisition Time	3.4079
11	Spectrometer Frequency	400.15
12	Spectral Width	9615.4
13	Lowest Frequency	-2344.9
14	Nucleus	1H
15	Acquired Size	32768
16	Spectral Size	65536

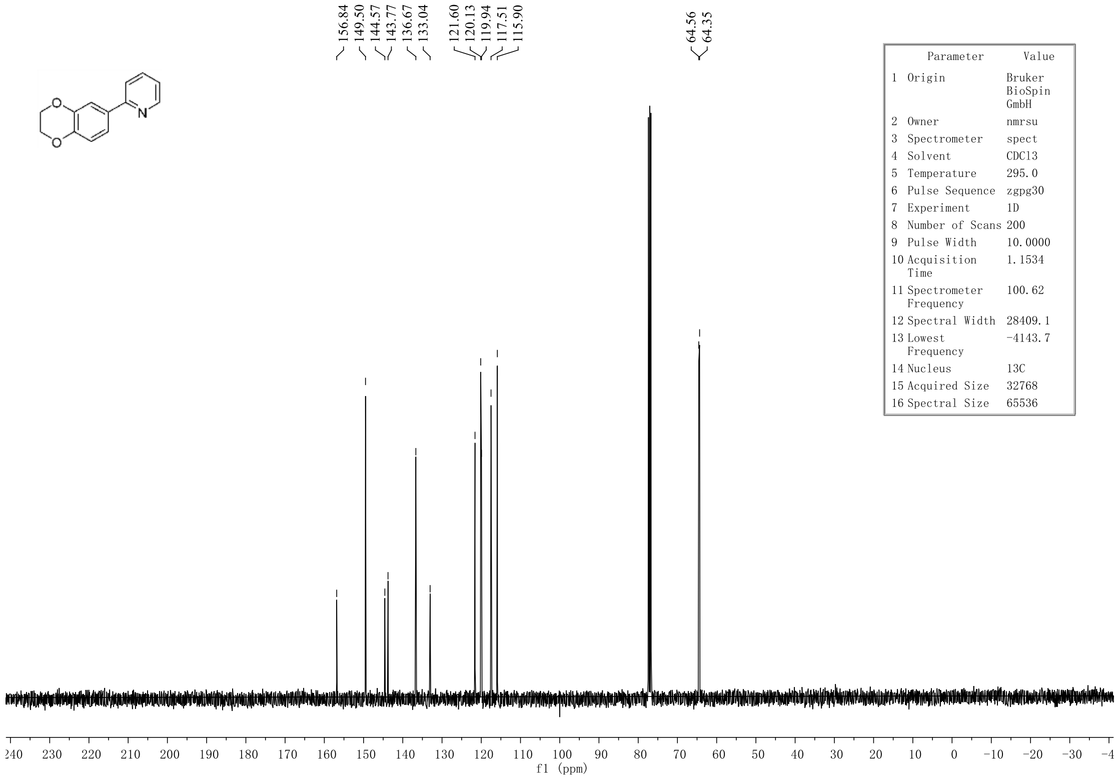

Parameter		Value
1	Origin	Bruker BioSpin GmbH
2	Owner	nmrsu
3	Spectrometer	spect
4	Solvent	CDCl3
5	Temperature	295.0
6	Pulse Sequence	zgpg30
7	Experiment	1D
8	Number of Scans	200
9	Pulse Width	10.0000
10	Acquisition Time	1.1534
11	Spectrometer Frequency	100.62
12	Spectral Width	28409.1
13	Lowest Frequency	-4143.7
14	Nucleus	13C
15	Acquired Size	32768
16	Spectral Size	65536

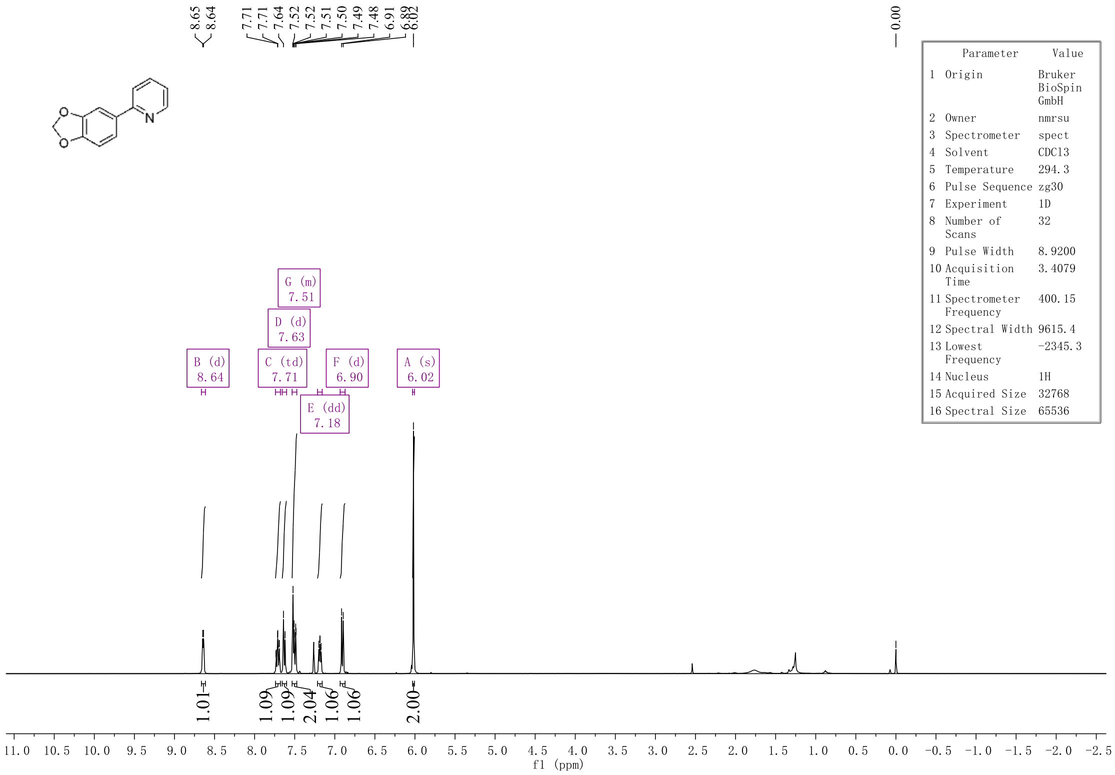

Parameter	Value
1 Origin	Bruker BioSpin GmbH
2 Owner	nmrsu
3 Spectrometer	spect
4 Solvent	CDCl3
5 Temperature	294.3
6 Pulse Sequence	zg30
7 Experiment	1D
8 Number of Scans	32
9 Pulse Width	8.9200
10 Acquisition Time	3.4079
11 Spectrometer Frequency	400.15
12 Spectral Width	9615.4
13 Lowest Frequency	-2345.3
14 Nucleus	1H
15 Acquired Size	32768
16 Spectral Size	65536

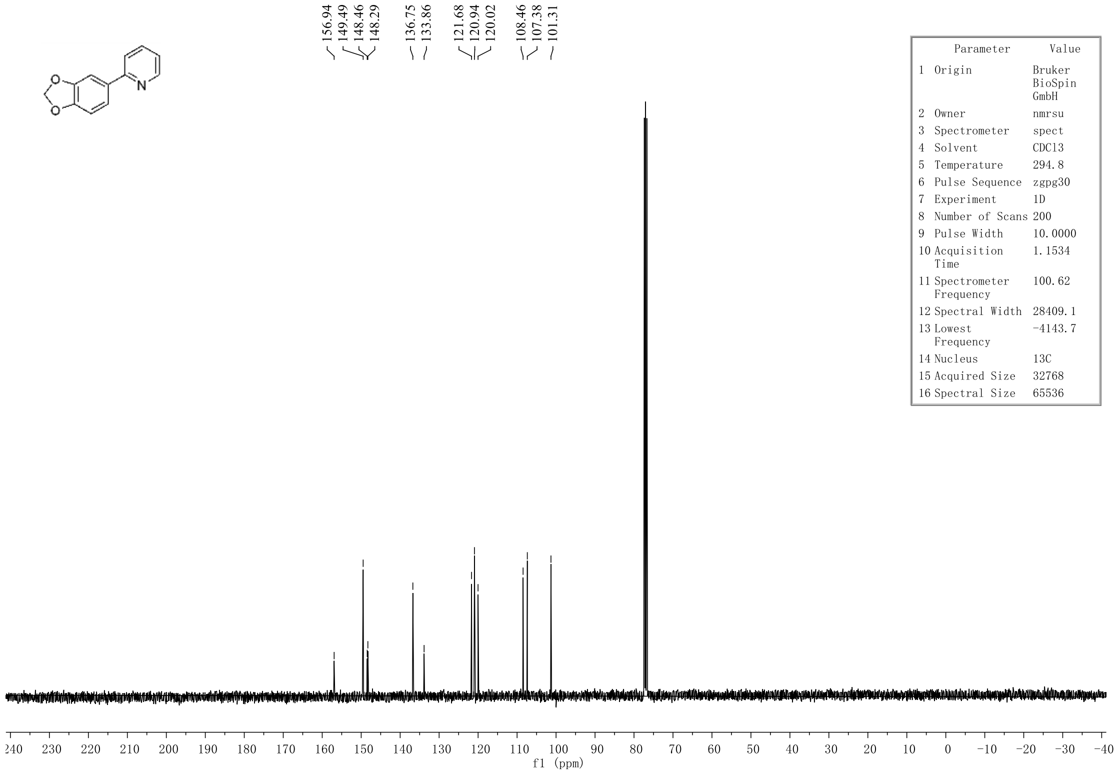

Parameter	Value
1 Origin	Bruker BioSpin GmbH
2 Owner	nmrsu
3 Spectrometer	spect
4 Solvent	CDCl3
5 Temperature	294.8
6 Pulse Sequence	zgpg30
7 Experiment	1D
8 Number of Scans	200
9 Pulse Width	10.0000
10 Acquisition Time	1.1534
11 Spectrometer Frequency	100.62
12 Spectral Width	28409.1
13 Lowest Frequency	-4143.7
14 Nucleus	13C
15 Acquired Size	32768
16 Spectral Size	65536

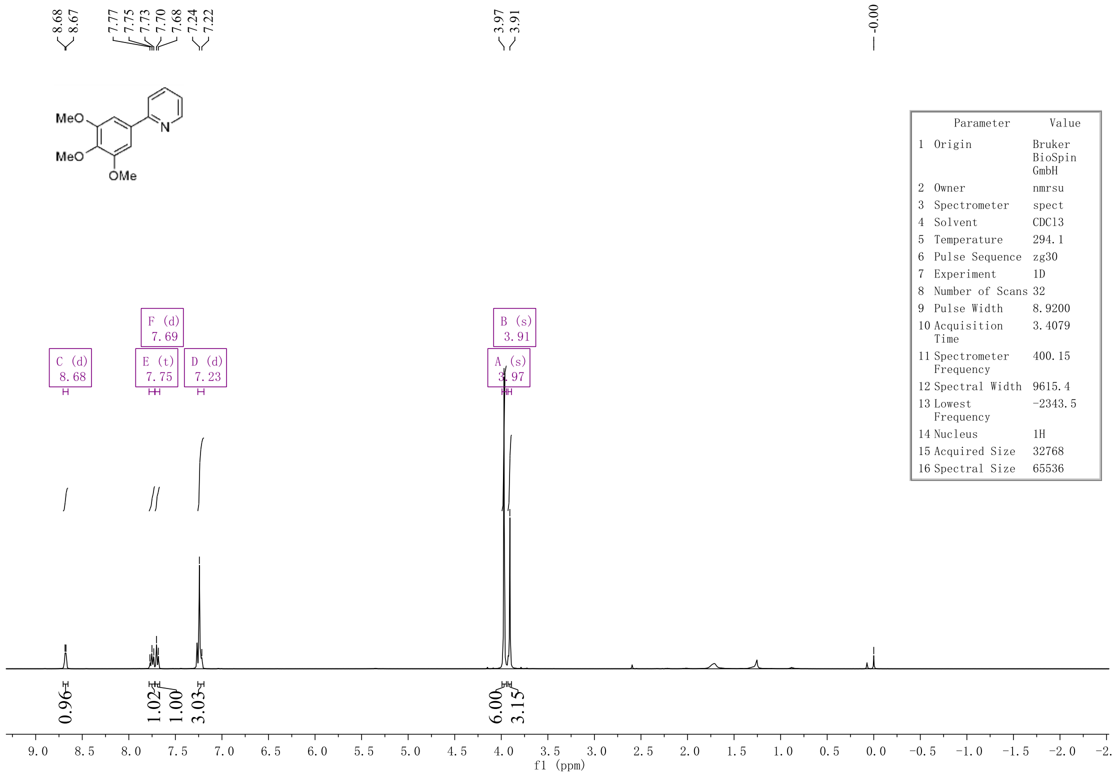

	Parameter	Value
1	Origin	Bruker BioSpin GmbH
2	Owner	nmrsu
3	Spectrometer	spect
4	Solvent	CDCl3
5	Temperature	294.1
6	Pulse Sequence	zg30
7	Experiment	1D
8	Number of Scans	32
9	Pulse Width	8.9200
10	Acquisition Time	3.4079
11	Spectrometer Frequency	400.15
12	Spectral Width	9615.4
13	Lowest Frequency	-2343.5
14	Nucleus	1H
15	Acquired Size	32768
16	Spectral Size	65536

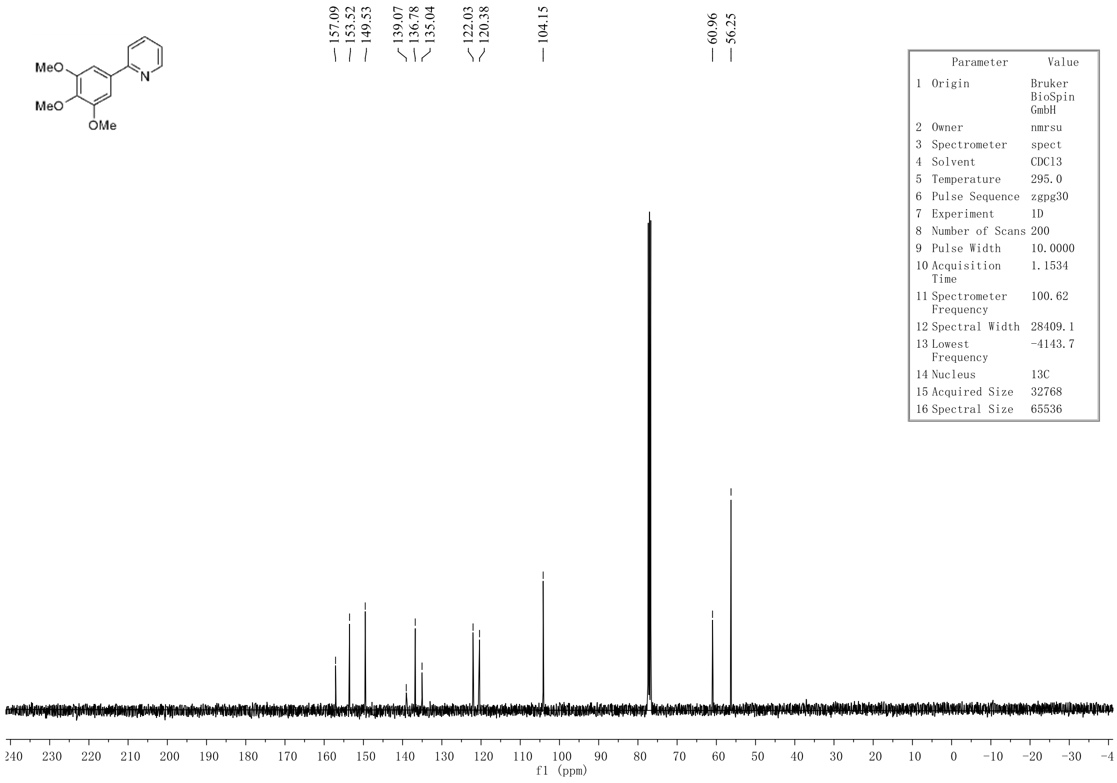

	Parameter	Value
1	Origin	Bruker BioSpin GmbH
2	Owner	nmrsu
3	Spectrometer	spect
4	Solvent	CDCl3
5	Temperature	295.0
6	Pulse Sequence	zgpg30
7	Experiment	1D
8	Number of Scans	200
9	Pulse Width	10.0000
10	Acquisition Time	1.1534
11	Spectrometer Frequency	100.62
12	Spectral Width	28409.1
13	Lowest Frequency	-4143.7
14	Nucleus	13C
15	Acquired Size	32768
16	Spectral Size	65536

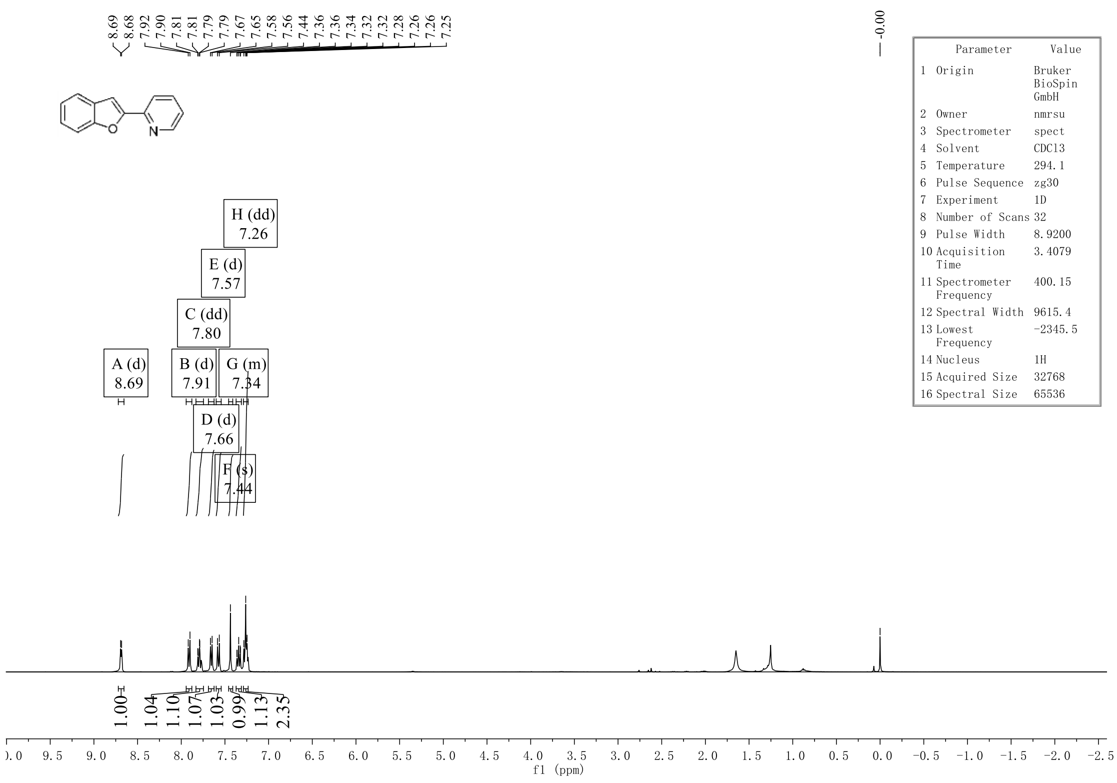
8.69
8.68
7.92
7.90
7.81
7.81
7.79
7.79
7.67
7.65
7.58
7.56
7.44
7.36
7.36
7.34
7.32
7.32
7.28
7.26
7.26
7.25
-0.00
N
O
H (dd) 7.26
E (d) 7.57
C (dd) 7.80
A (d) 8.69
B (d) 7.91
G (m) 7.34
D (d) 7.66
F (s) 7.44
1.00
1.04
1.10
1.07
1.03
0.99
1.13
2.35
f1 (ppm)
Parameter Value
1 Origin Bruker BioSpin GmbH
2 Owner nmrsu
3 Spectrometer spect
4 Solvent CDCl3
5 Temperature 294.1
6 Pulse Sequence zg30
7 Experiment 1D
8 Number of Scans 32
9 Pulse Width 8.9200
10 Acquisition Time 3.4079
11 Spectrometer Frequency 400.15
12 Spectral Width 9615.4
13 Lowest Frequency -2345.5
14 Nucleus 1H
15 Acquired Size 32768
16 Spectral Size 65536

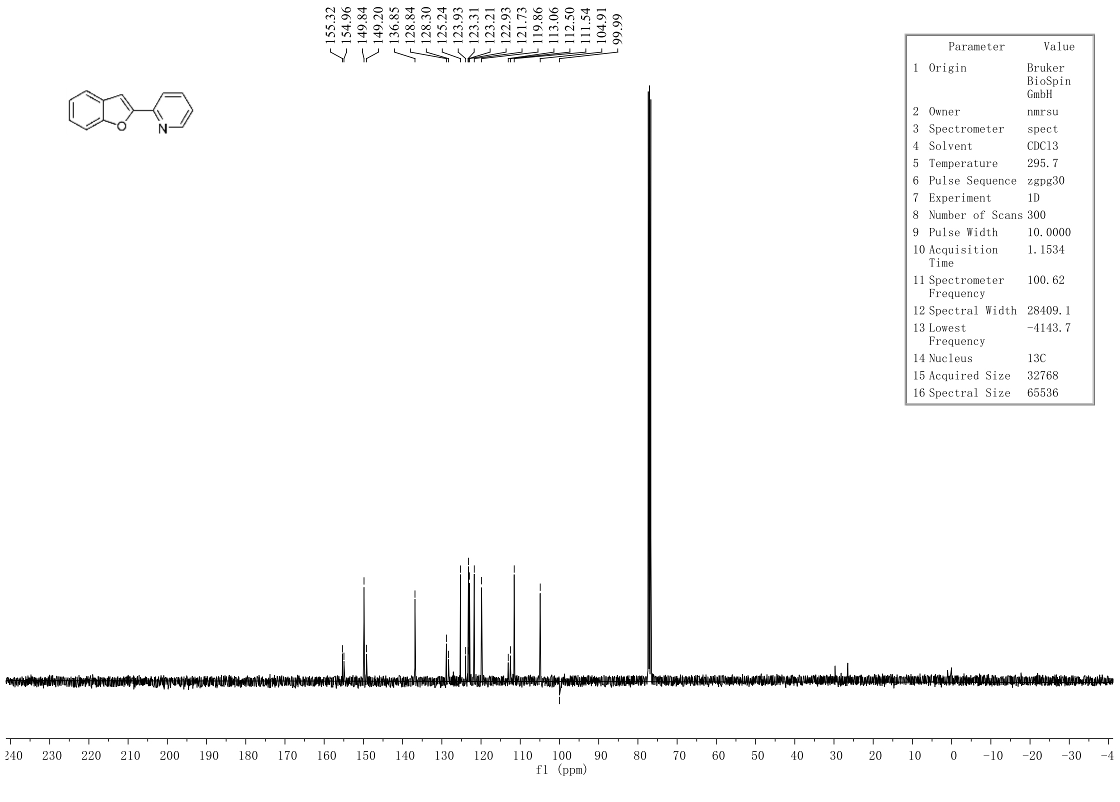
155.32
154.96
149.84
149.20
136.85
128.84
128.30
125.24
123.93
123.31
123.21
122.93
121.73
119.86
113.06
112.50
111.54
104.91
99.99
N
O
f1 (ppm)
Parameter Value
1 Origin Bruker BioSpin GmbH
2 Owner nmrsu
3 Spectrometer spect
4 Solvent CDCl3
5 Temperature 295.7
6 Pulse Sequence zgpg30
7 Experiment 1D
8 Number of Scans 300
9 Pulse Width 10.0000
10 Acquisition Time 1.1534
11 Spectrometer Frequency 100.62
12 Spectral Width 28409.1
13 Lowest Frequency -4143.7
14 Nucleus 13C
15 Acquired Size 32768
16 Spectral Size 65536

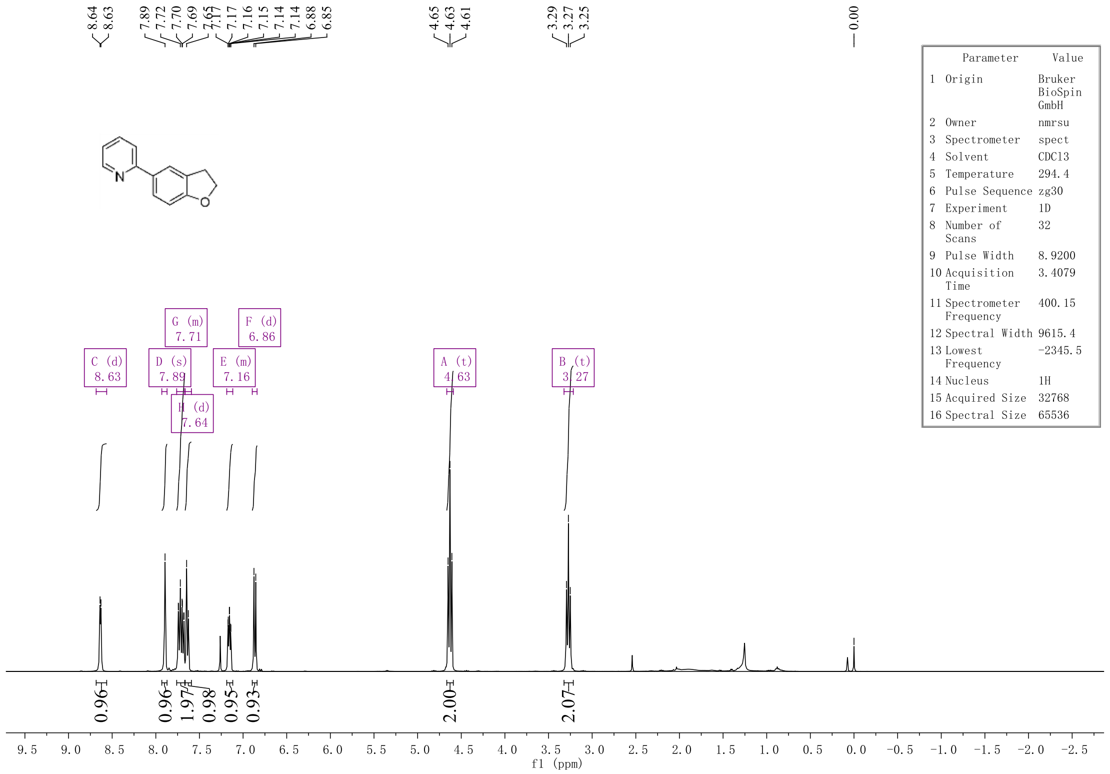

	Parameter	Value
1	Origin	Bruker BioSpin GmbH
2	Owner	nmrsu
3	Spectrometer	spect
4	Solvent	CDCl3
5	Temperature	294.4
6	Pulse Sequence	zg30
7	Experiment	1D
8	Number of Scans	32
9	Pulse Width	8.9200
10	Acquisition Time	3.4079
11	Spectrometer Frequency	400.15
12	Spectral Width	9615.4
13	Lowest Frequency	-2345.5
14	Nucleus	1H
15	Acquired Size	32768
16	Spectral Size	65536

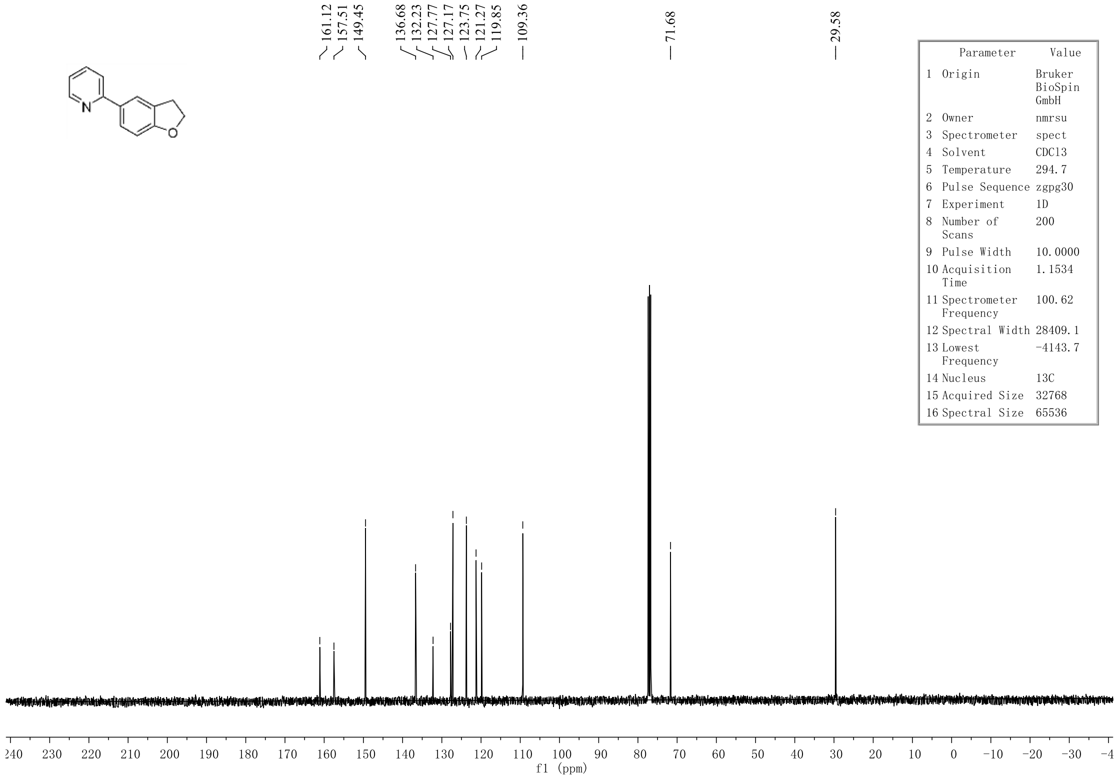

	Parameter	Value
1	Origin	Bruker BioSpin GmbH
2	Owner	nmrsu
3	Spectrometer	spect
4	Solvent	CDCl3
5	Temperature	294.7
6	Pulse Sequence	zgpg30
7	Experiment	1D
8	Number of Scans	200
9	Pulse Width	10.0000
10	Acquisition Time	1.1534
11	Spectrometer Frequency	100.62
12	Spectral Width	28409.1
13	Lowest Frequency	-4143.7
14	Nucleus	13C
15	Acquired Size	32768
16	Spectral Size	65536

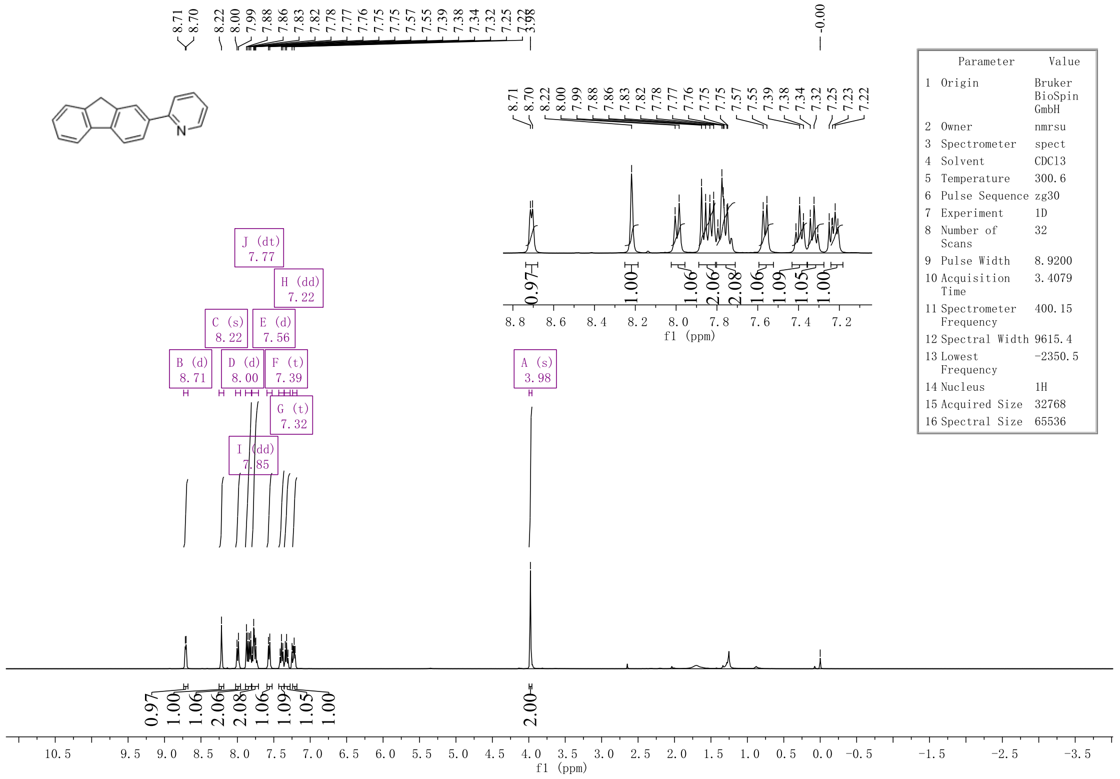
8.71
8.70
8.22
8.00
7.99
7.88
7.86
7.83
7.82
7.78
7.77
7.76
7.75
7.75
7.57
7.55
7.39
7.38
7.34
7.32
7.25
7.23
7.22
3.98
-0.00
N
J (dt) 7.77
H (dd) 7.22
C (s) 8.22
E (d) 7.56
B (d) 8.71
D (d) 8.00
F (t) 7.39
A (s) 3.98
G (t) 7.32
I (dd) 7.85
0.97
1.00
1.06
2.06
2.08
1.06
1.09
1.05
1.00
2.00
f1 (ppm)
Parameter Value
1 Origin Bruker BioSpin GmbH
2 Owner nmrsu
3 Spectrometer spect
4 Solvent CDCl3
5 Temperature 300.6
6 Pulse Sequence zg30
7 Experiment 1D
8 Number of Scans 32
9 Pulse Width 8.9200
10 Acquisition Time 3.4079
11 Spectrometer Frequency 400.15
12 Spectral Width 9615.4
13 Lowest Frequency -2350.5
14 Nucleus 1H
15 Acquired Size 32768
16 Spectral Size 65536

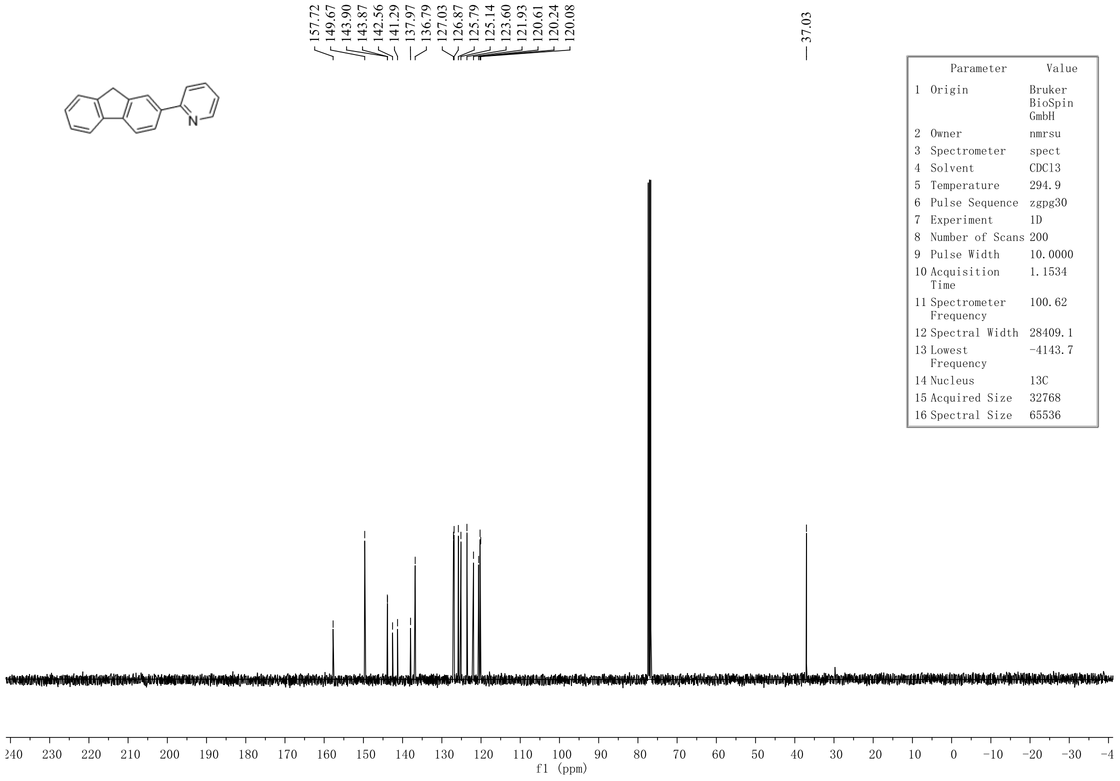
157.72
149.67
143.90
143.87
142.56
141.29
137.97
136.79
127.03
126.87
125.79
125.14
123.60
121.93
120.61
120.24
120.08
37.03
N
f1 (ppm)
Parameter Value
1 Origin Bruker BioSpin GmbH
2 Owner nmrsu
3 Spectrometer spect
4 Solvent CDCl3
5 Temperature 294.9
6 Pulse Sequence zgpg30
7 Experiment 1D
8 Number of Scans 200
9 Pulse Width 10.0000
10 Acquisition Time 1.1534
11 Spectrometer Frequency 100.62
12 Spectral Width 28409.1
13 Lowest Frequency -4143.7
14 Nucleus 13C
15 Acquired Size 32768
16 Spectral Size 65536

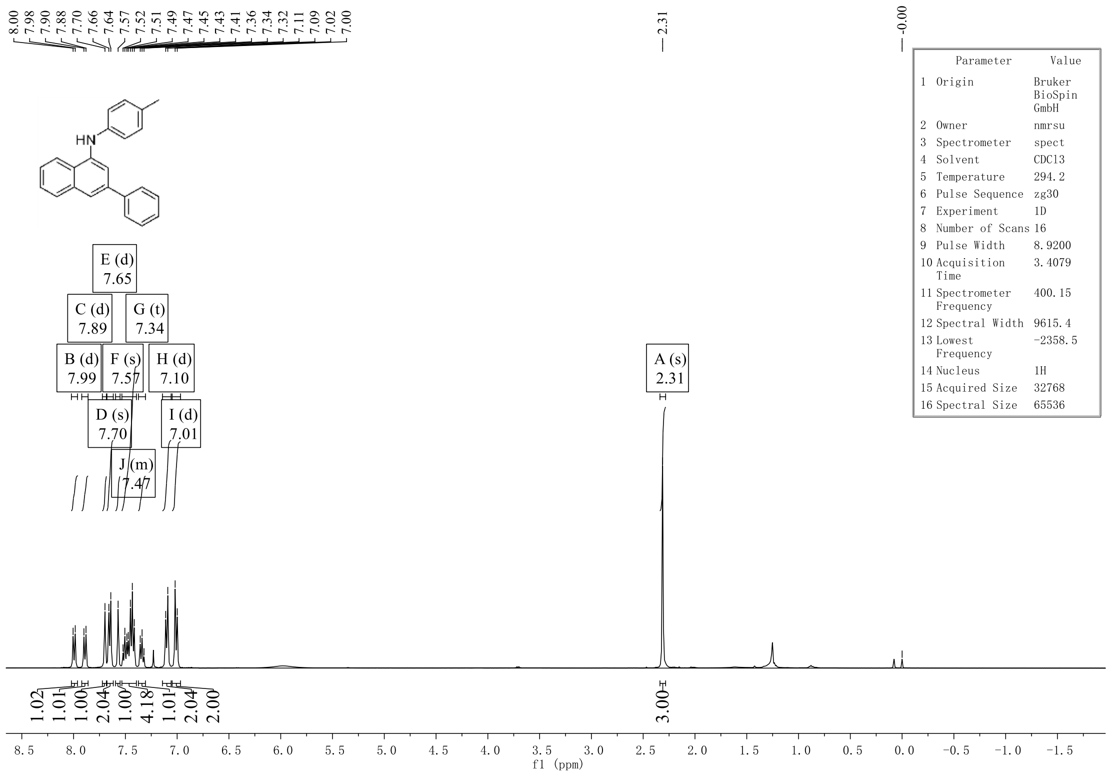

8.00
7.98
7.90
7.88
7.70
7.66
7.64
7.57
7.52
7.51
7.49
7.47
7.45
7.43
7.41
7.36
7.34
7.32
7.11
7.09
7.02
7.00
2.31
-0.00
HN
E (d) 7.65
C (d) 7.89
G (t) 7.34
B (d) 7.99
F (s) 7.57
H (d) 7.10
A (s) 2.31
D (s) 7.70
I (d) 7.01
J (m) 7.47
1.02
1.01
1.00
2.04
1.00
4.18
1.01
2.04
2.00
3.00
f1 (ppm)
Parameter Value
1 Origin Bruker BioSpin GmbH
2 Owner nmrsu
3 Spectrometer spect
4 Solvent CDCl3
5 Temperature 294.2
6 Pulse Sequence zg30
7 Experiment 1D
8 Number of Scans 16
9 Pulse Width 8.9200
10 Acquisition Time 3.4079
11 Spectrometer Frequency 400.15
12 Spectral Width 9615.4
13 Lowest Frequency -2358.5
14 Nucleus 1H
15 Acquired Size 32768
16 Spectral Size 65536

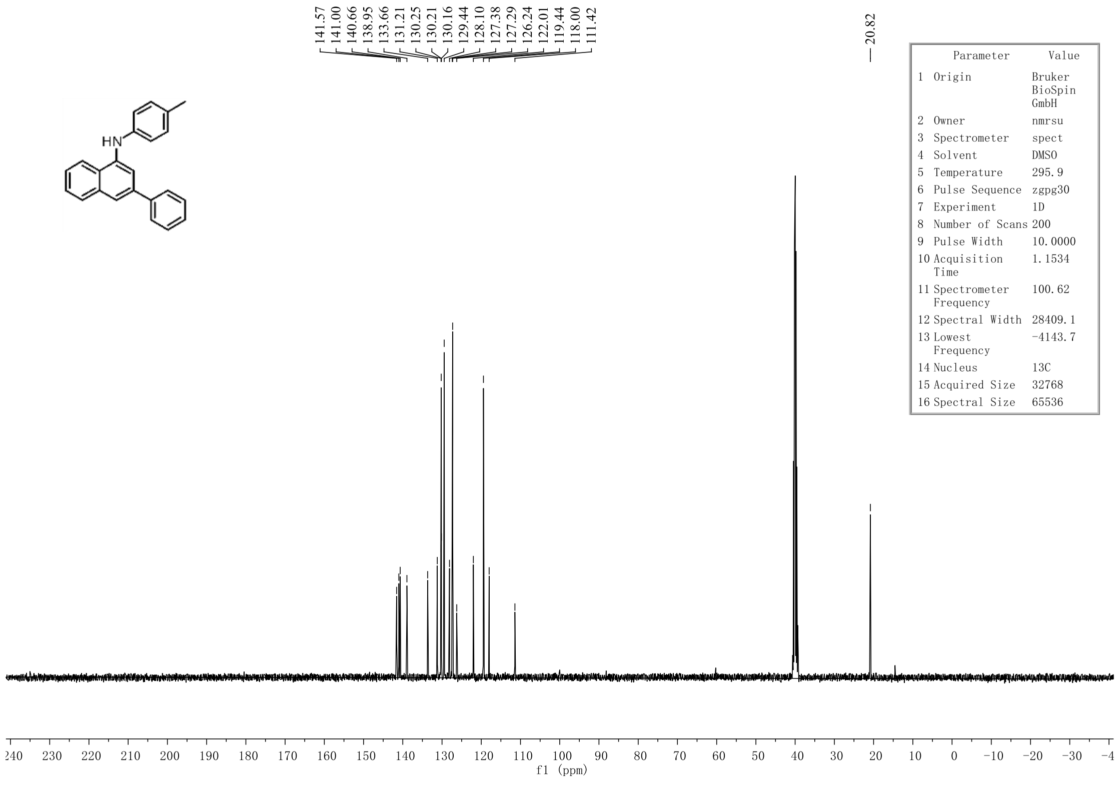

141.57
141.00
140.66
138.95
133.66
131.21
130.25
130.21
130.16
129.44
128.10
127.38
127.29
126.24
122.01
119.44
118.00
111.42
20.82
HN
f1 (ppm)
Parameter Value
1 Origin Bruker BioSpin GmbH
2 Owner nmrsu
3 Spectrometer spect
4 Solvent DMSO
5 Temperature 295.9
6 Pulse Sequence zgpg30
7 Experiment 1D
8 Number of Scans 200
9 Pulse Width 10.0000
10 Acquisition Time 1.1534
11 Spectrometer Frequency 100.62
12 Spectral Width 28409.1
13 Lowest Frequency -4143.7
14 Nucleus 13C
15 Acquired Size 32768
16 Spectral Size 65536

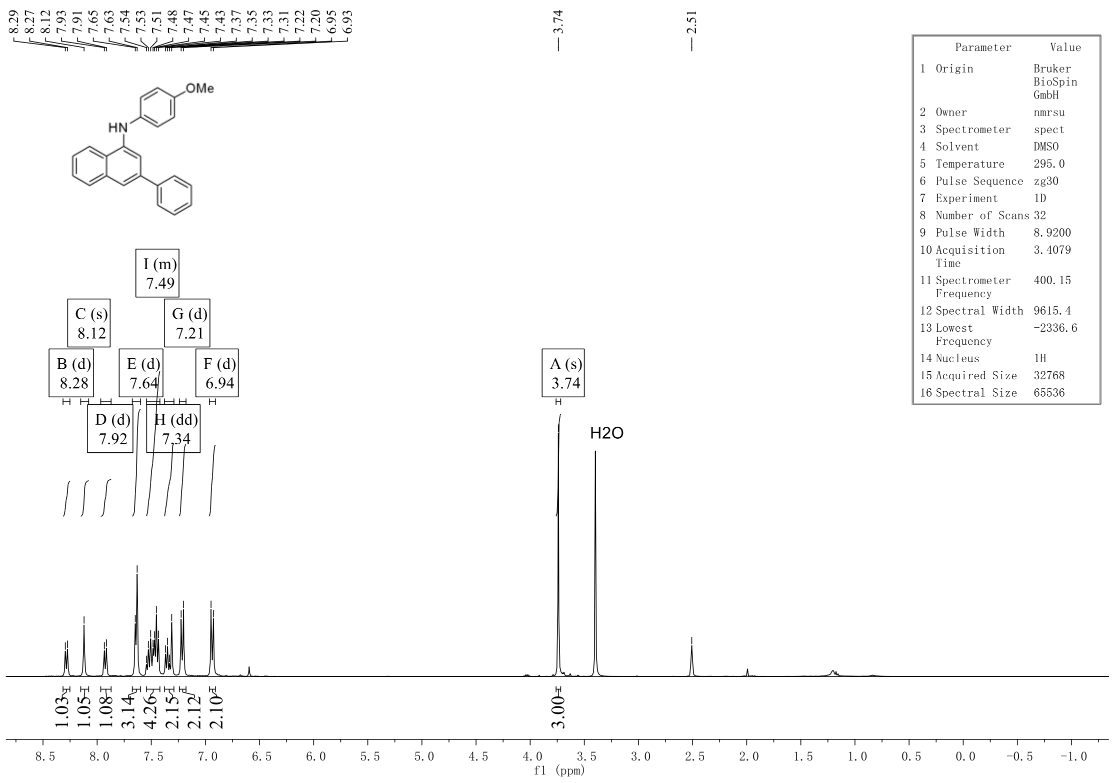

8.29
8.27
8.12
7.93
7.91
7.65
7.63
7.54
7.53
7.51
7.48
7.47
7.45
7.43
7.37
7.35
7.33
7.31
7.22
7.20
6.95
6.93
3.74
2.51
OMe
HN
I (m) 7.49
C (s) 8.12
G (d) 7.21
B (d) 8.28
E (d) 7.64
F (d) 6.94
A (s) 3.74
D (d) 7.92
H (dd) 7.34
H2O
1.03
1.05
1.08
3.14
4.26
2.15
2.12
2.10
3.00
f1 (ppm)
Parameter Value
1 Origin Bruker BioSpin GmbH
2 Owner nmrsu
3 Spectrometer spect
4 Solvent DMSO
5 Temperature 295.0
6 Pulse Sequence zg30
7 Experiment 1D
8 Number of Scans 32
9 Pulse Width 8.9200
10 Acquisition Time 3.4079
11 Spectrometer Frequency 400.15
12 Spectral Width 9615.4
13 Lowest Frequency -2336.6
14 Nucleus 1H
15 Acquired Size 32768
16 Spectral Size 65536

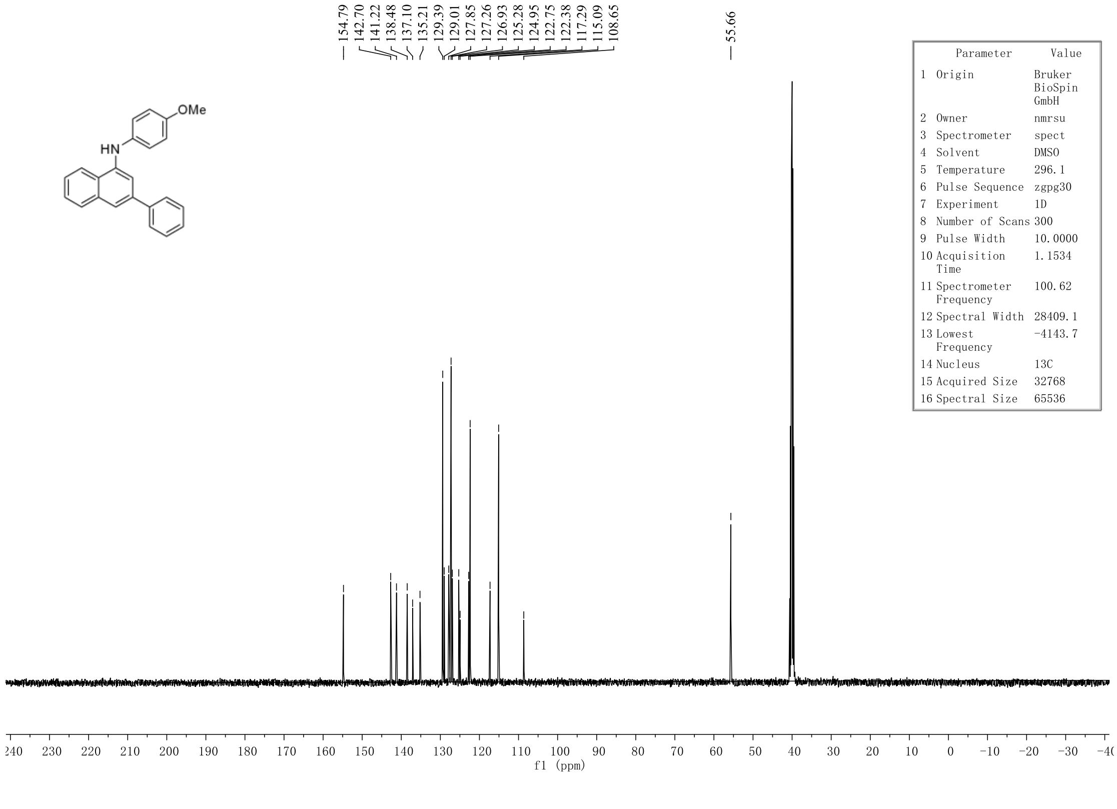

154.79
142.70
141.22
138.48
137.10
135.21
129.39
129.01
127.85
127.26
126.93
125.28
124.95
122.75
122.38
117.29
115.09
108.65
55.66
OMe
HN
f1 (ppm)
Parameter Value
1 Origin Bruker BioSpin GmbH
2 Owner nmrsu
3 Spectrometer spect
4 Solvent DMSO
5 Temperature 296.1
6 Pulse Sequence zgpg30
7 Experiment 1D
8 Number of Scans 300
9 Pulse Width 10.0000
10 Acquisition Time 1.1534
11 Spectrometer Frequency 100.62
12 Spectral Width 28409.1
13 Lowest Frequency -4143.7
14 Nucleus 13C
15 Acquired Size 32768
16 Spectral Size 65536

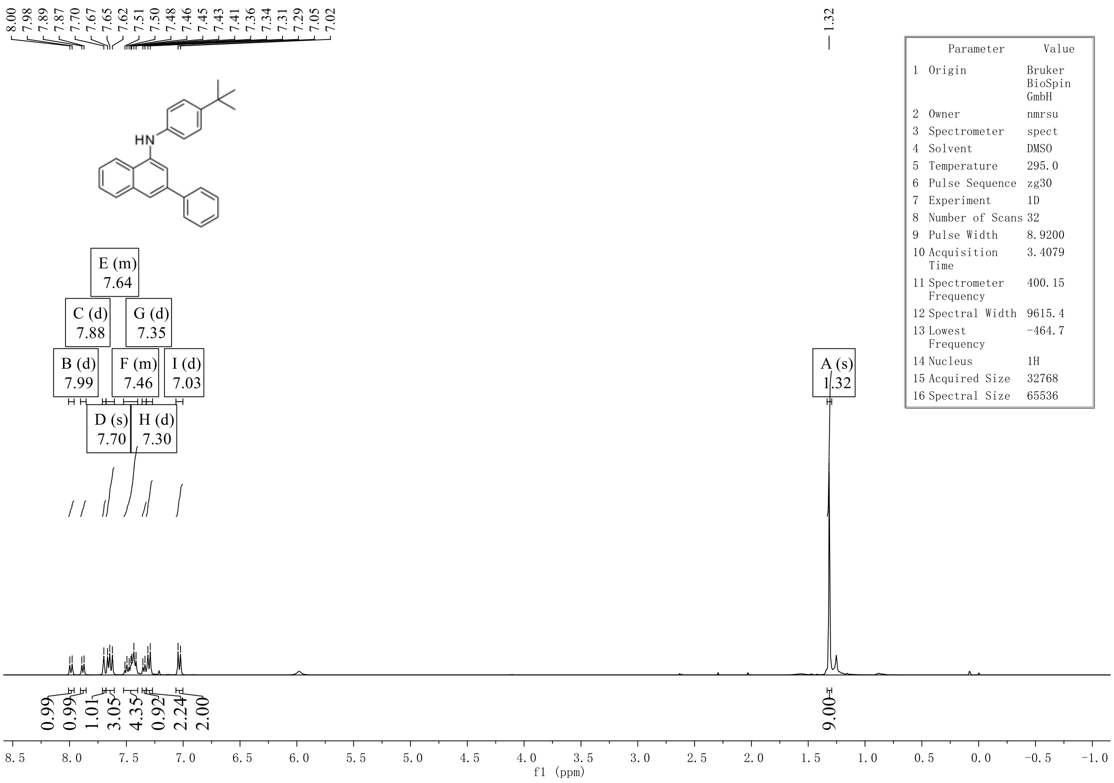

Parameter	Value
1 Origin	Bruker BioSpin GmbH
2 Owner	nmrsu
3 Spectrometer	spect
4 Solvent	DMSO
5 Temperature	295.0
6 Pulse Sequence	zg30
7 Experiment	1D
8 Number of Scans	32
9 Pulse Width	8.9200
10 Acquisition Time	3.4079
11 Spectrometer Frequency	400.15
12 Spectral Width	9615.4
13 Lowest Frequency	-464.7
14 Nucleus	1H
15 Acquired Size	32768
16 Spectral Size	65536

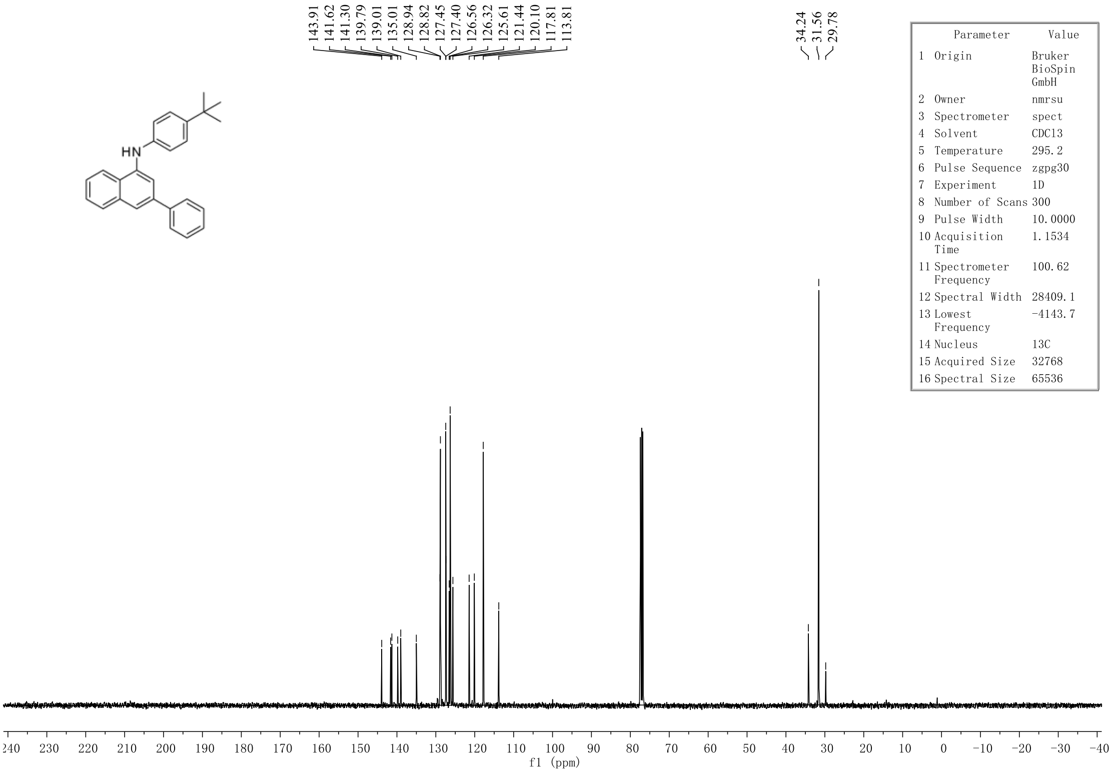

Parameter	Value
1 Origin	Bruker BioSpin GmbH
2 Owner	nmrsu
3 Spectrometer	spect
4 Solvent	CDCl3
5 Temperature	295.2
6 Pulse Sequence	zgpg30
7 Experiment	1D
8 Number of Scans	300
9 Pulse Width	10.0000
10 Acquisition Time	1.1534
11 Spectrometer Frequency	100.62
12 Spectral Width	28409.1
13 Lowest Frequency	-4143.7
14 Nucleus	13C
15 Acquired Size	32768
16 Spectral Size	65536

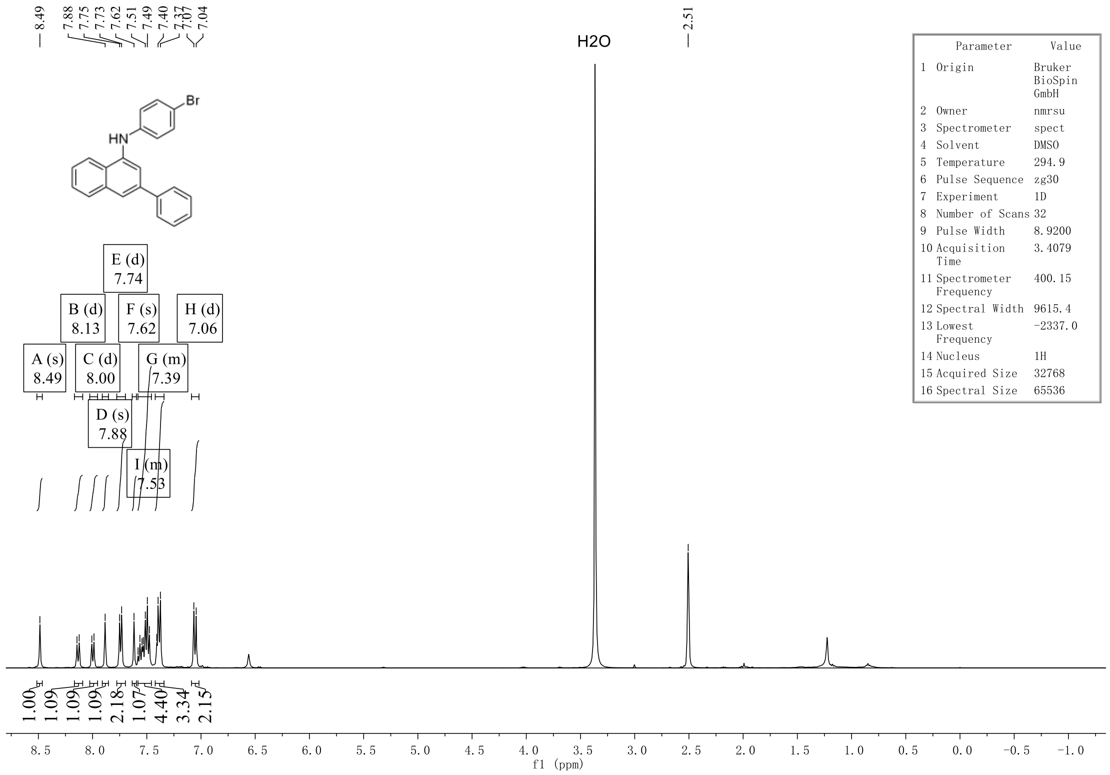

Parameter	Value
1 Origin	Bruker BioSpin GmbH
2 Owner	nmrsu
3 Spectrometer	spect
4 Solvent	DMSO
5 Temperature	294.9
6 Pulse Sequence	zg30
7 Experiment	1D
8 Number of Scans	32
9 Pulse Width	8.9200
10 Acquisition Time	3.4079
11 Spectrometer Frequency	400.15
12 Spectral Width	9615.4
13 Lowest Frequency	-2337.0
14 Nucleus	1H
15 Acquired Size	32768
16 Spectral Size	65536

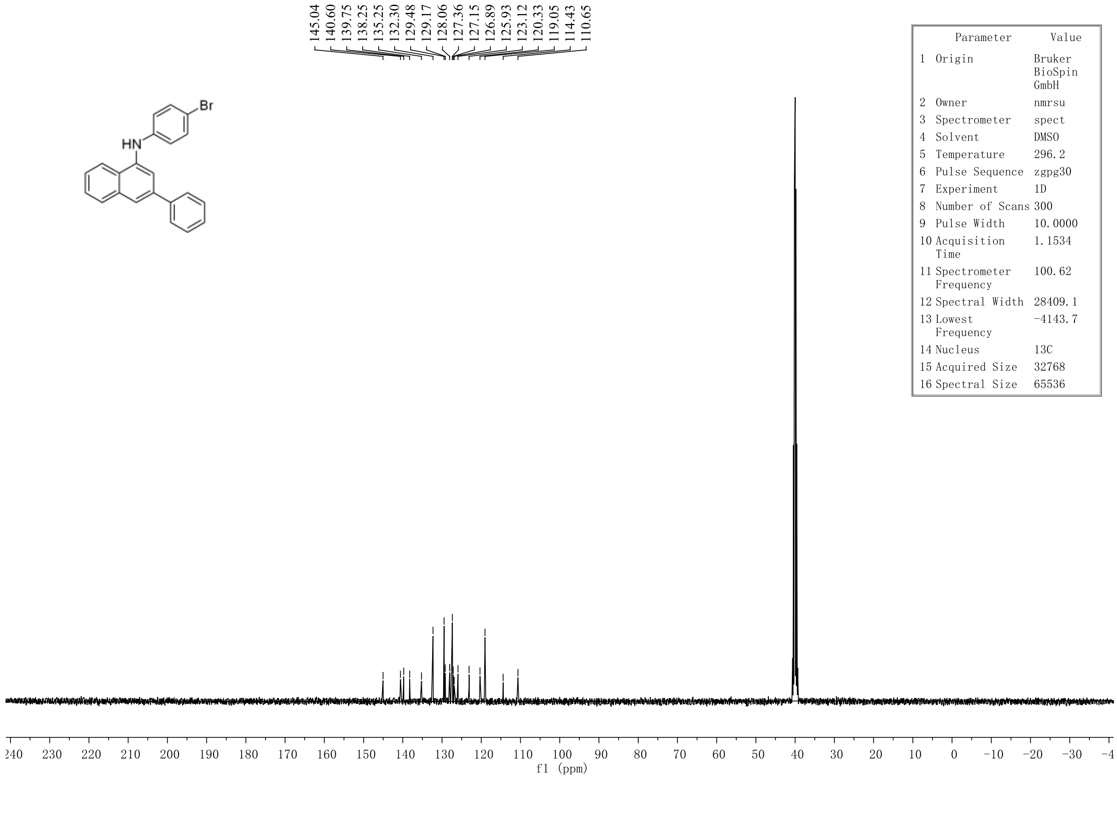

Parameter	Value
1 Origin	Bruker BioSpin GmbH
2 Owner	nmrsu
3 Spectrometer	spect
4 Solvent	DMSO
5 Temperature	296.2
6 Pulse Sequence	zgpg30
7 Experiment	1D
8 Number of Scans	300
9 Pulse Width	10.0000
10 Acquisition Time	1.1534
11 Spectrometer Frequency	100.62
12 Spectral Width	28409.1
13 Lowest Frequency	-4143.7
14 Nucleus	13C
15 Acquired Size	32768
16 Spectral Size	65536

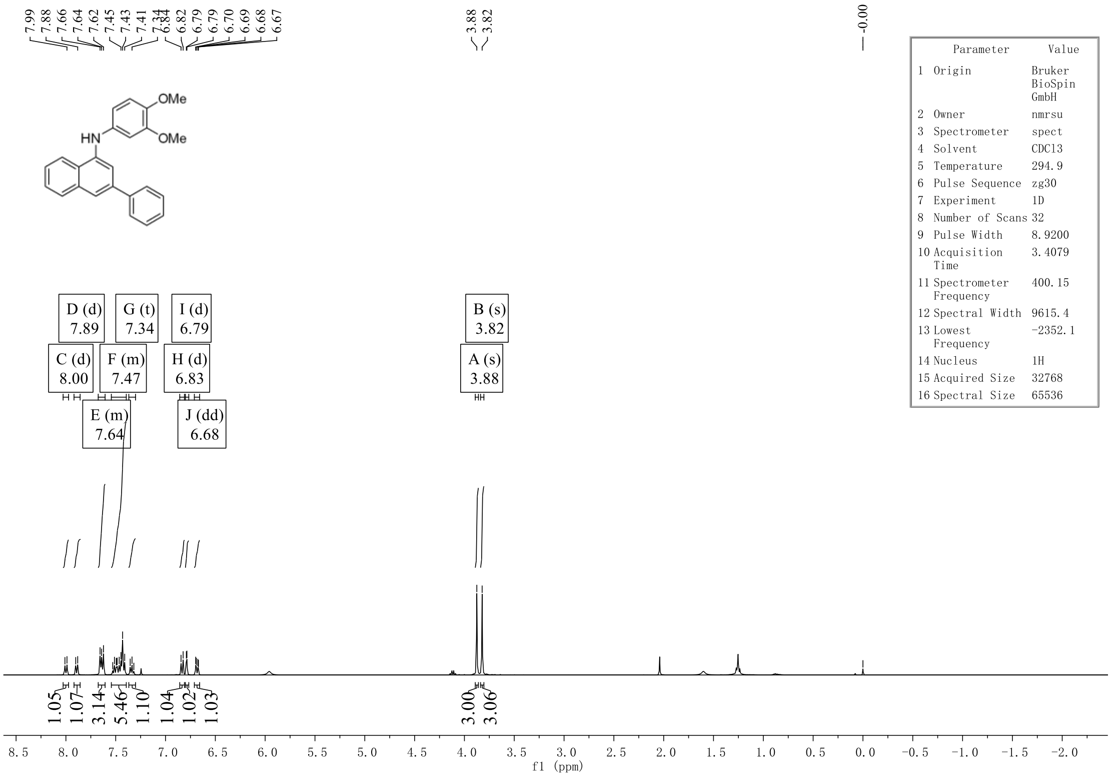

	Parameter	Value
1	Origin	Bruker BioSpin GmbH
2	Owner	nmrsu
3	Spectrometer	spect
4	Solvent	CDCl3
5	Temperature	294.9
6	Pulse Sequence	zg30
7	Experiment	1D
8	Number of Scans	32
9	Pulse Width	8.9200
10	Acquisition Time	3.4079
11	Spectrometer Frequency	400.15
12	Spectral Width	9615.4
13	Lowest Frequency	-2352.1
14	Nucleus	1H
15	Acquired Size	32768
16	Spectral Size	65536

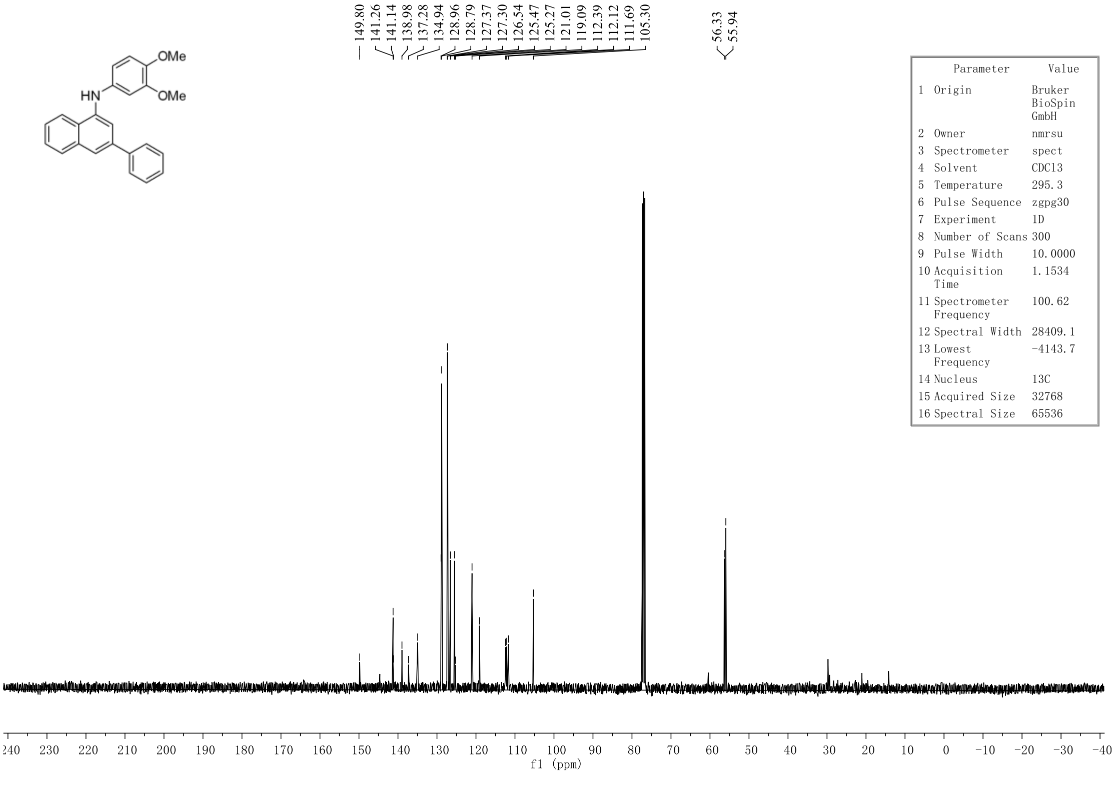

	Parameter	Value
1	Origin	Bruker BioSpin GmbH
2	Owner	nmrsu
3	Spectrometer	spect
4	Solvent	CDCl3
5	Temperature	295.3
6	Pulse Sequence	zgpg30
7	Experiment	1D
8	Number of Scans	300
9	Pulse Width	10.0000
10	Acquisition Time	1.1534
11	Spectrometer Frequency	100.62
12	Spectral Width	28409.1
13	Lowest Frequency	-4143.7
14	Nucleus	13C
15	Acquired Size	32768
16	Spectral Size	65536

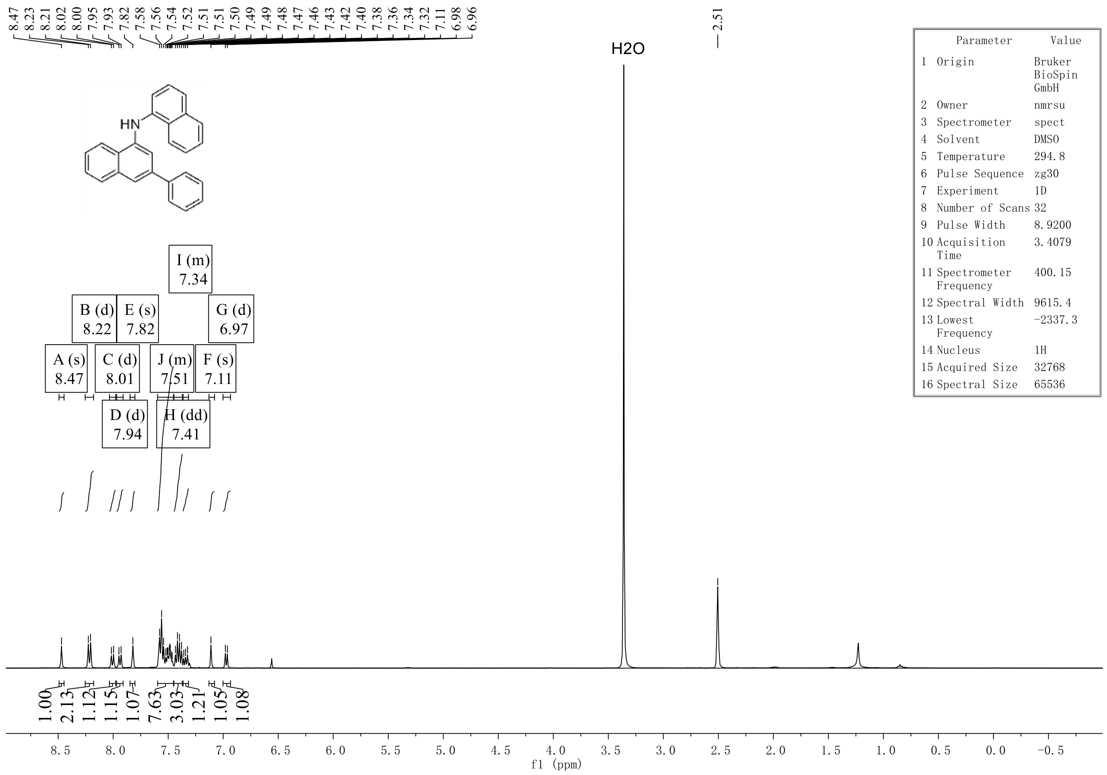

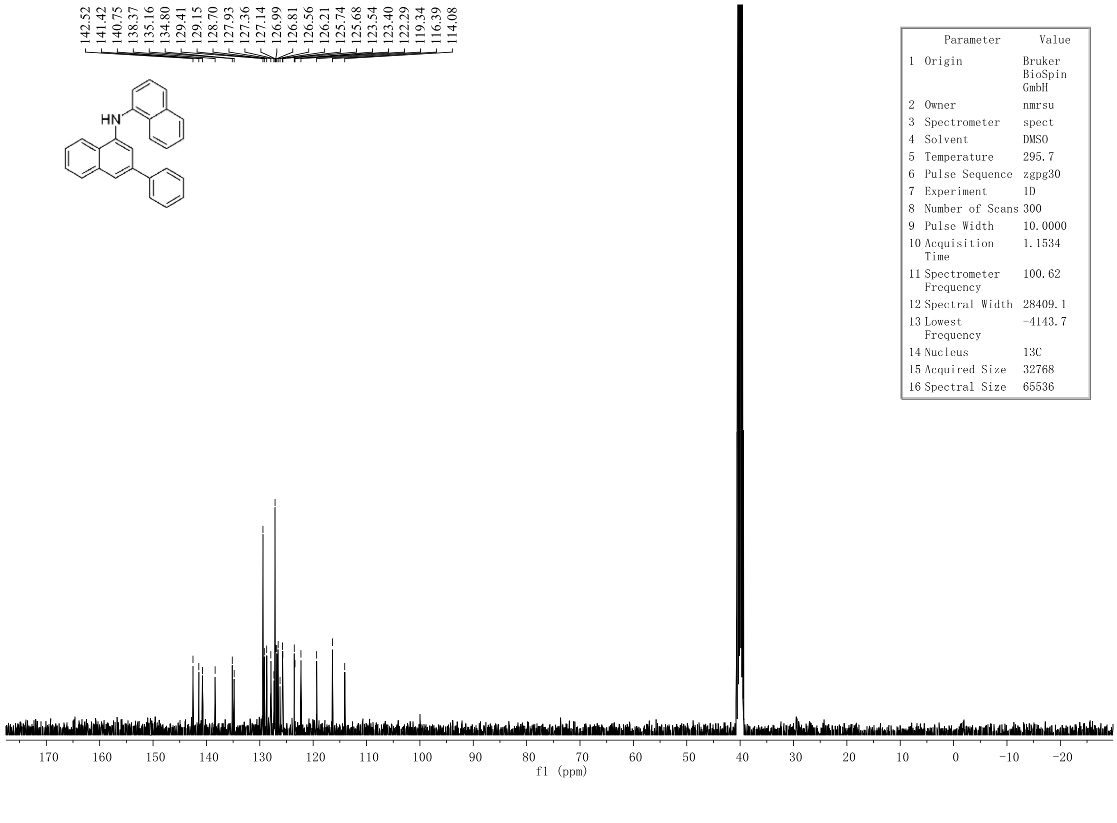

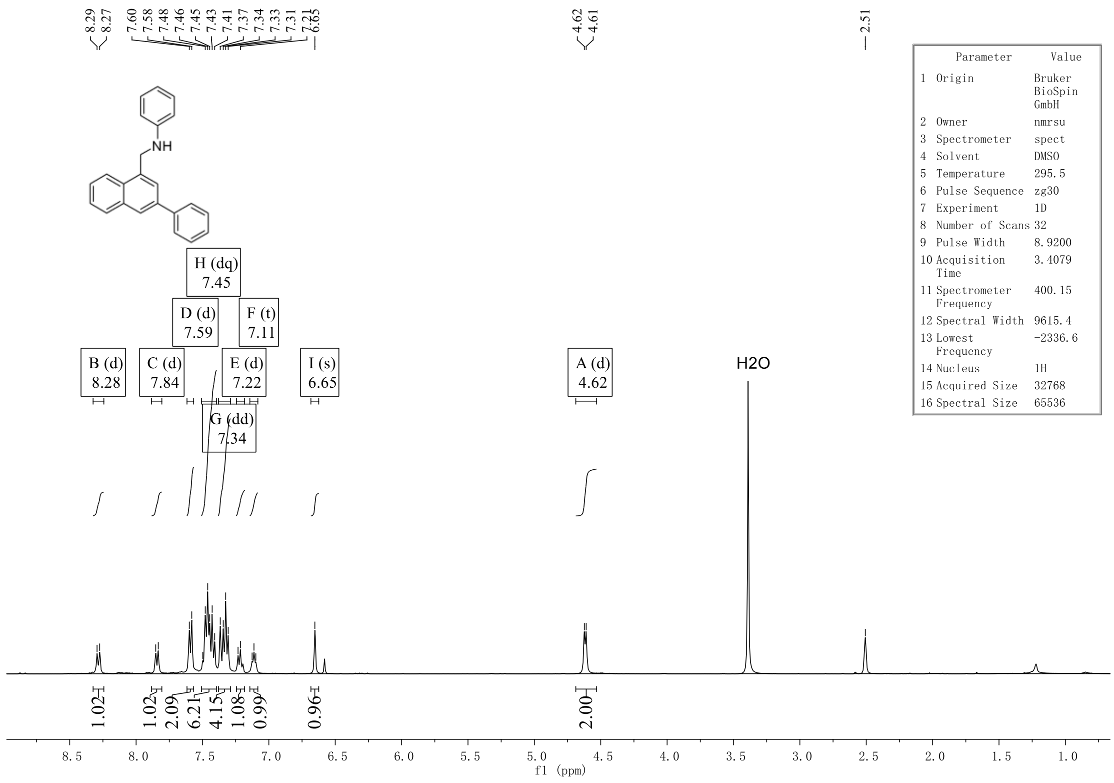

	Parameter	Value
1	Origin	Bruker BioSpin GmbH
2	Owner	nmrsu
3	Spectrometer	spect
4	Solvent	DMSO
5	Temperature	295.5
6	Pulse Sequence	zg30
7	Experiment	1D
8	Number of Scans	32
9	Pulse Width	8.9200
10	Acquisition Time	3.4079
11	Spectrometer Frequency	400.15
12	Spectral Width	9615.4
13	Lowest Frequency	-2336.6
14	Nucleus	1H
15	Acquired Size	32768
16	Spectral Size	65536

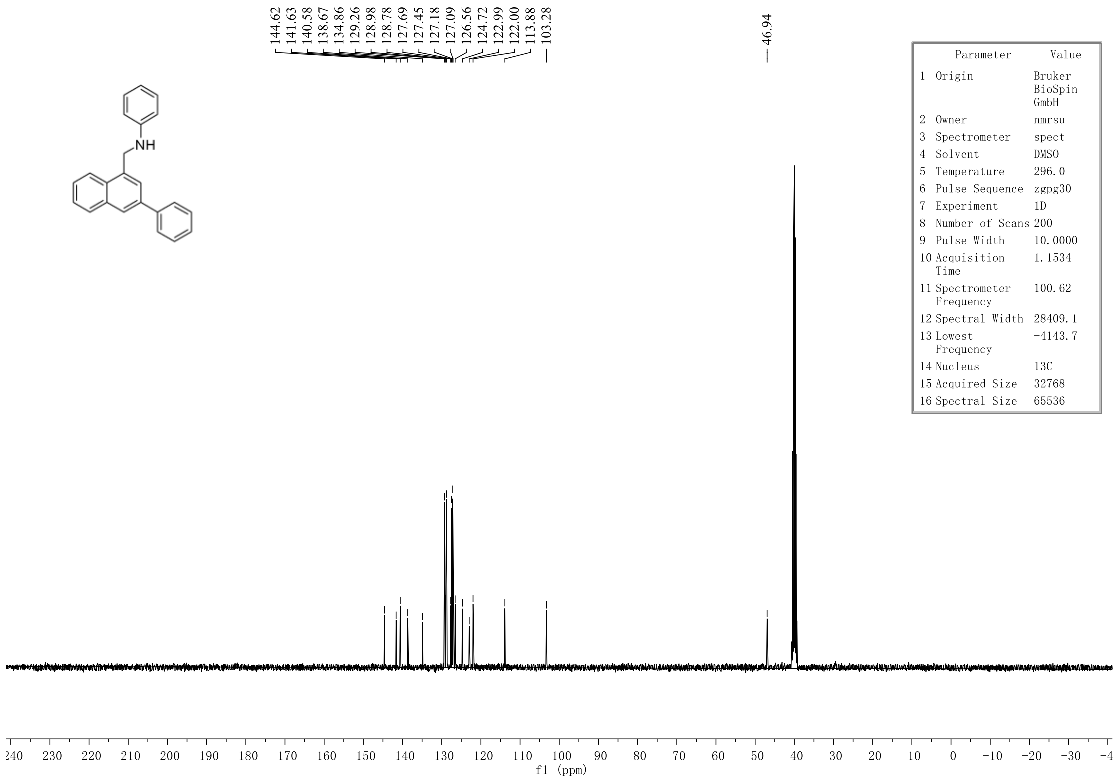

	Parameter	Value
1	Origin	Bruker BioSpin GmbH
2	Owner	nmrsu
3	Spectrometer	spect
4	Solvent	DMSO
5	Temperature	296.0
6	Pulse Sequence	zgpg30
7	Experiment	1D
8	Number of Scans	200
9	Pulse Width	10.0000
10	Acquisition Time	1.1534
11	Spectrometer Frequency	100.62
12	Spectral Width	28409.1
13	Lowest Frequency	-4143.7
14	Nucleus	13C
15	Acquired Size	32768
16	Spectral Size	65536

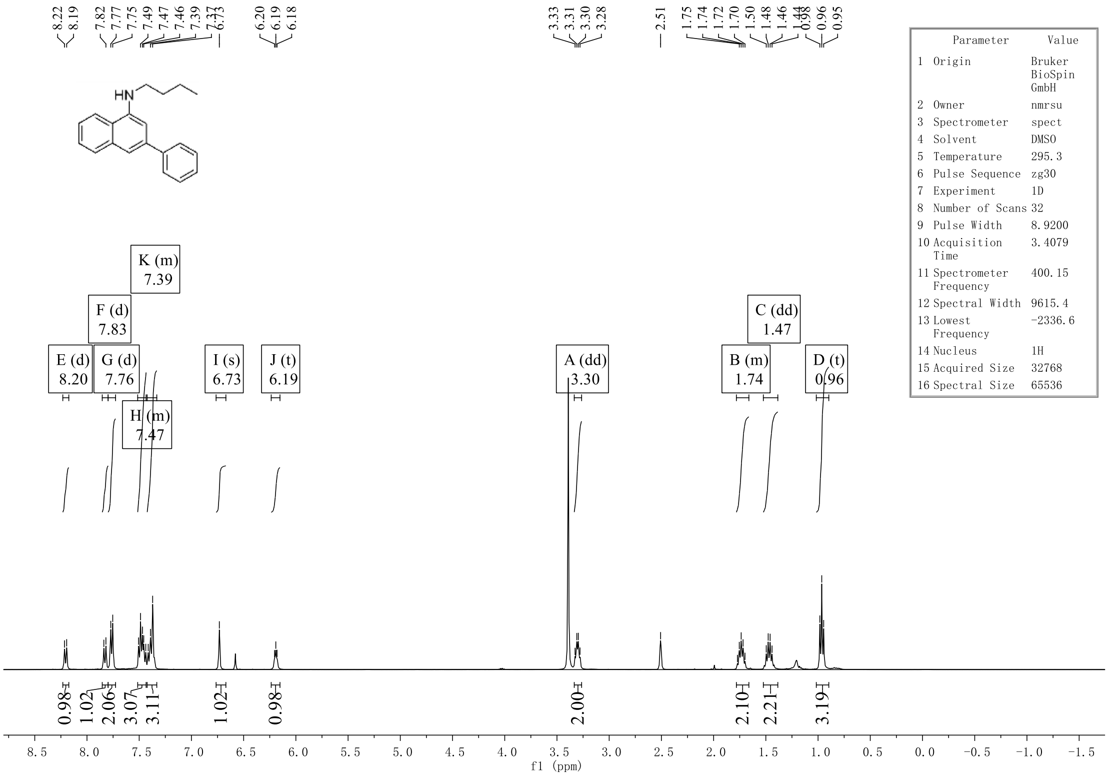

	Parameter	Value
1	Origin	Bruker BioSpin GmbH
2	Owner	nmrsu
3	Spectrometer	spect
4	Solvent	DMSO
5	Temperature	295.3
6	Pulse Sequence	zg30
7	Experiment	1D
8	Number of Scans	32
9	Pulse Width	8.9200
10	Acquisition Time	3.4079
11	Spectrometer Frequency	400.15
12	Spectral Width	9615.4
13	Lowest Frequency	−2336.6
14	Nucleus	1H
15	Acquired Size	32768
16	Spectral Size	65536

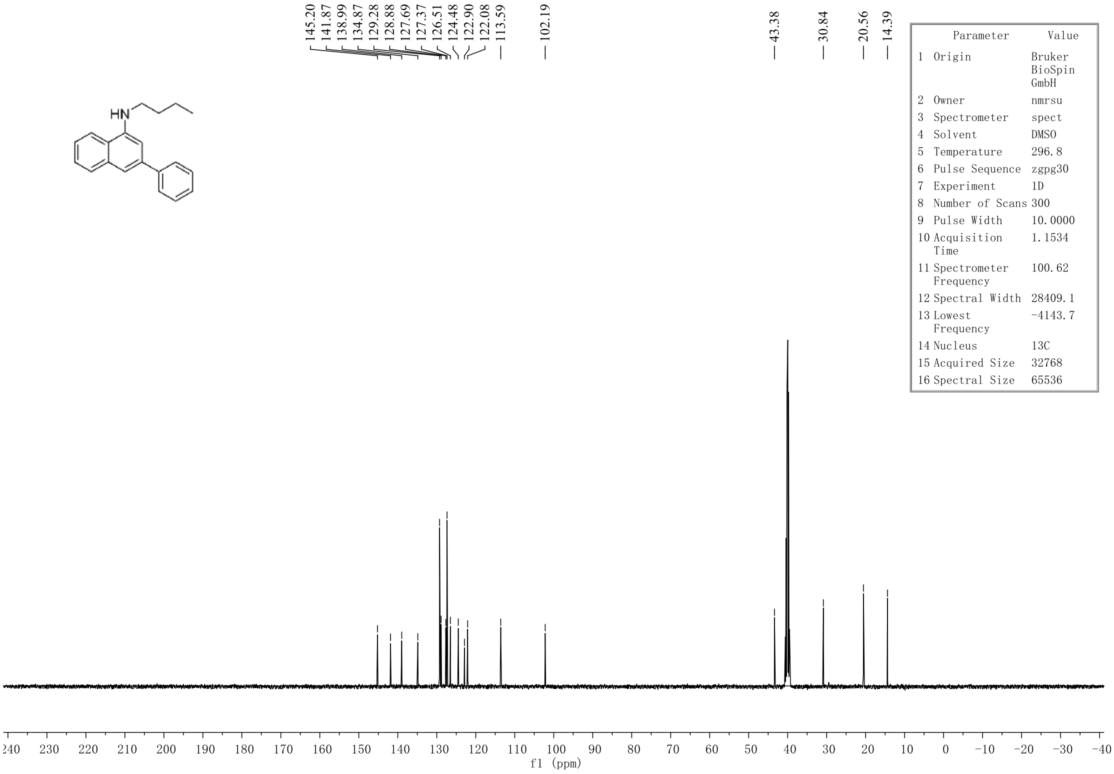

	Parameter	Value
1	Origin	Bruker BioSpin GmbH
2	Owner	nmrsu
3	Spectrometer	spect
4	Solvent	DMSO
5	Temperature	296.8
6	Pulse Sequence	zgpg30
7	Experiment	1D
8	Number of Scans	300
9	Pulse Width	10.0000
10	Acquisition Time	1.1534
11	Spectrometer Frequency	100.62
12	Spectral Width	28409.1
13	Lowest Frequency	−4143.7
14	Nucleus	13C
15	Acquired Size	32768
16	Spectral Size	65536

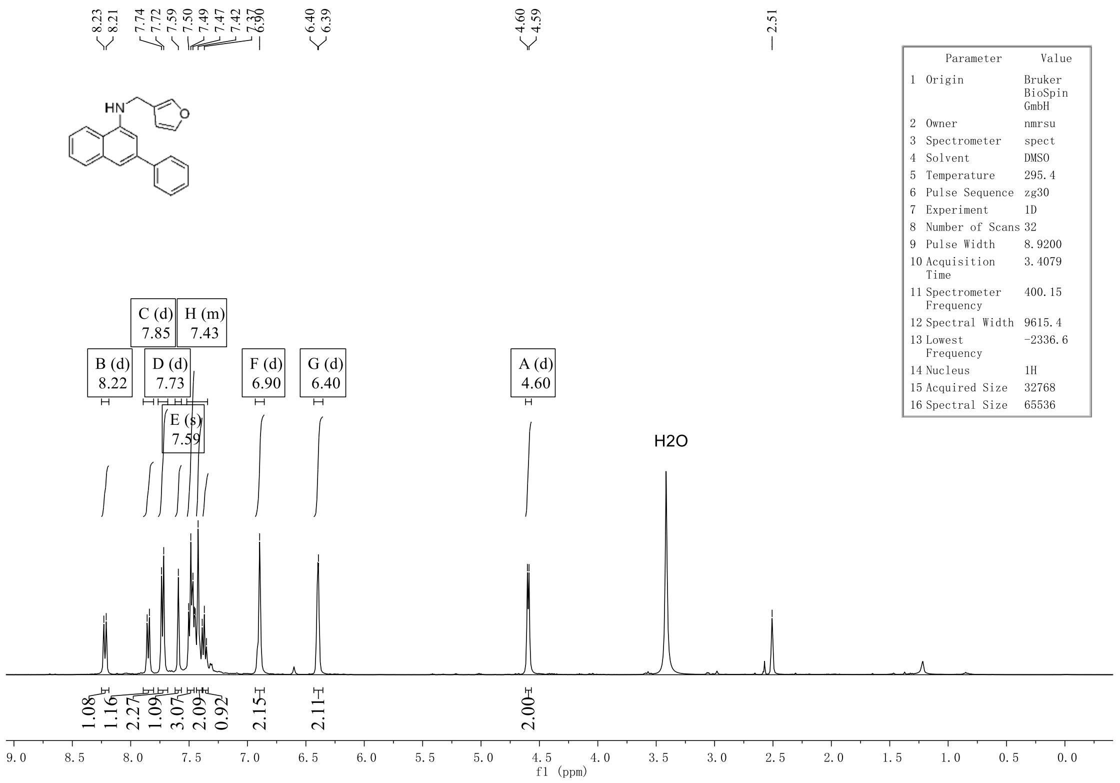

Parameter		Value
1	Origin	Bruker BioSpin GmbH
2	Owner	nmrsu
3	Spectrometer	spect
4	Solvent	DMSO
5	Temperature	295.4
6	Pulse Sequence	zg30
7	Experiment	1D
8	Number of Scans	32
9	Pulse Width	8.9200
10	Acquisition Time	3.4079
11	Spectrometer Frequency	400.15
12	Spectral Width	9615.4
13	Lowest Frequency	-2336.6
14	Nucleus	1H
15	Acquired Size	32768
16	Spectral Size	65536

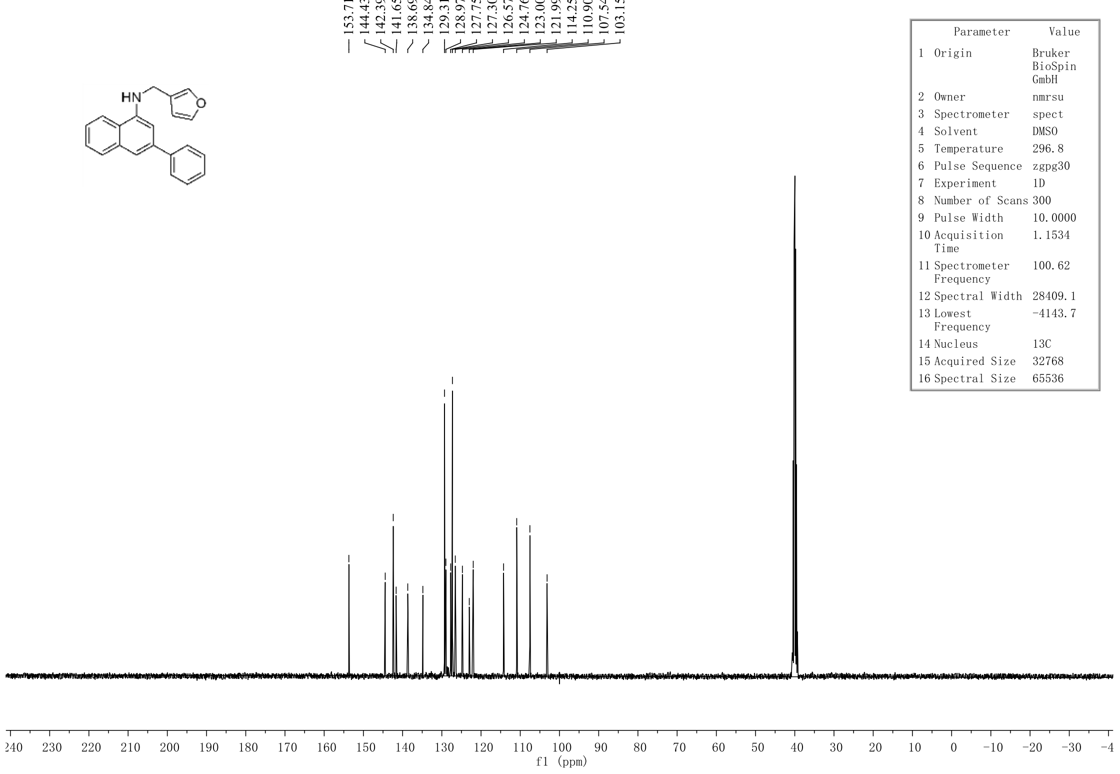

Parameter		Value
1	Origin	Bruker BioSpin GmbH
2	Owner	nmrsu
3	Spectrometer	spect
4	Solvent	DMSO
5	Temperature	296.8
6	Pulse Sequence	zgpg30
7	Experiment	1D
8	Number of Scans	300
9	Pulse Width	10.0000
10	Acquisition Time	1.1534
11	Spectrometer Frequency	100.62
12	Spectral Width	28409.1
13	Lowest Frequency	-4143.7
14	Nucleus	13C
15	Acquired Size	32768
16	Spectral Size	65536

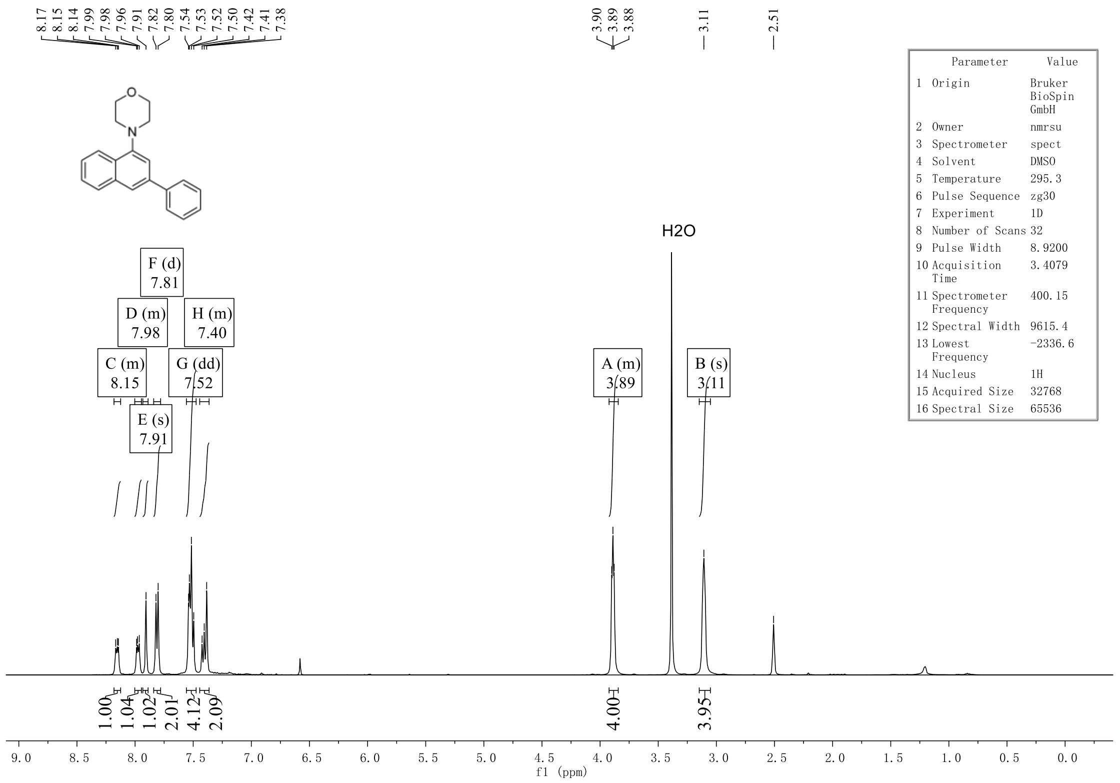
8.17 8.15 8.14 7.99 7.98 7.96 7.91 7.82 7.80 7.54 7.53 7.52 7.50 7.42 7.41 7.38
3.90 3.89 3.88
3.11
2.51
H2O
F (d) 7.81
D (m) 7.98
H (m) 7.40
C (m) 8.15
G (dd) 7.52
E (s) 7.91
A (m) 3.89
B (s) 3.11
1.00 1.04 1.02 2.01 4.12 2.09
4.00
3.95
f1 (ppm)
Parameter Value
1 Origin Bruker BioSpin GmbH
2 Owner nmrsu
3 Spectrometer spect
4 Solvent DMSO
5 Temperature 295.3
6 Pulse Sequence zg30
7 Experiment 1D
8 Number of Scans 32
9 Pulse Width 8.9200
10 Acquisition Time 3.4079
11 Spectrometer Frequency 400.15
12 Spectral Width 9615.4
13 Lowest Frequency -2336.6
14 Nucleus 1H
15 Acquired Size 32768
16 Spectral Size 65536

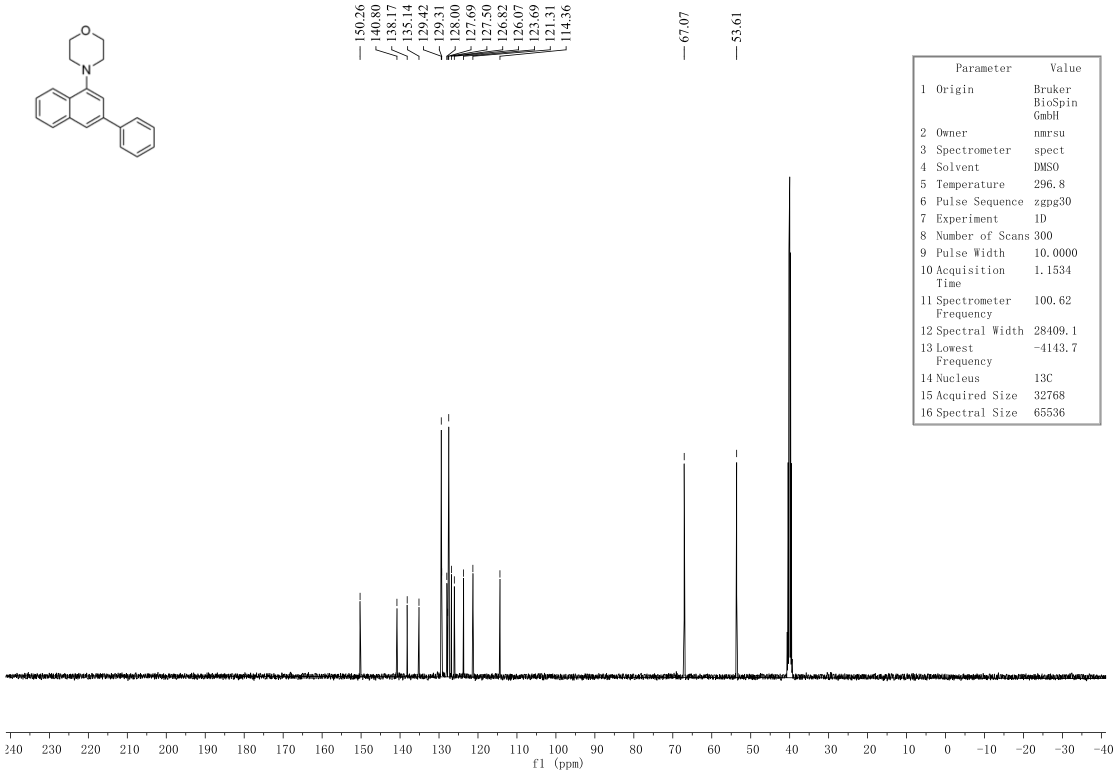
150.26 140.80 138.17 135.14 129.42 129.31 128.00 127.69 127.50 126.82 126.07 123.69 121.31 114.36
67.07
53.61
f1 (ppm)
Parameter Value
1 Origin Bruker BioSpin GmbH
2 Owner nmrsu
3 Spectrometer spect
4 Solvent DMSO
5 Temperature 296.8
6 Pulse Sequence zgpg30
7 Experiment 1D
8 Number of Scans 300
9 Pulse Width 10.0000
10 Acquisition Time 1.1534
11 Spectrometer Frequency 100.62
12 Spectral Width 28409.1
13 Lowest Frequency -4143.7
14 Nucleus 13C
15 Acquired Size 32768
16 Spectral Size 65536

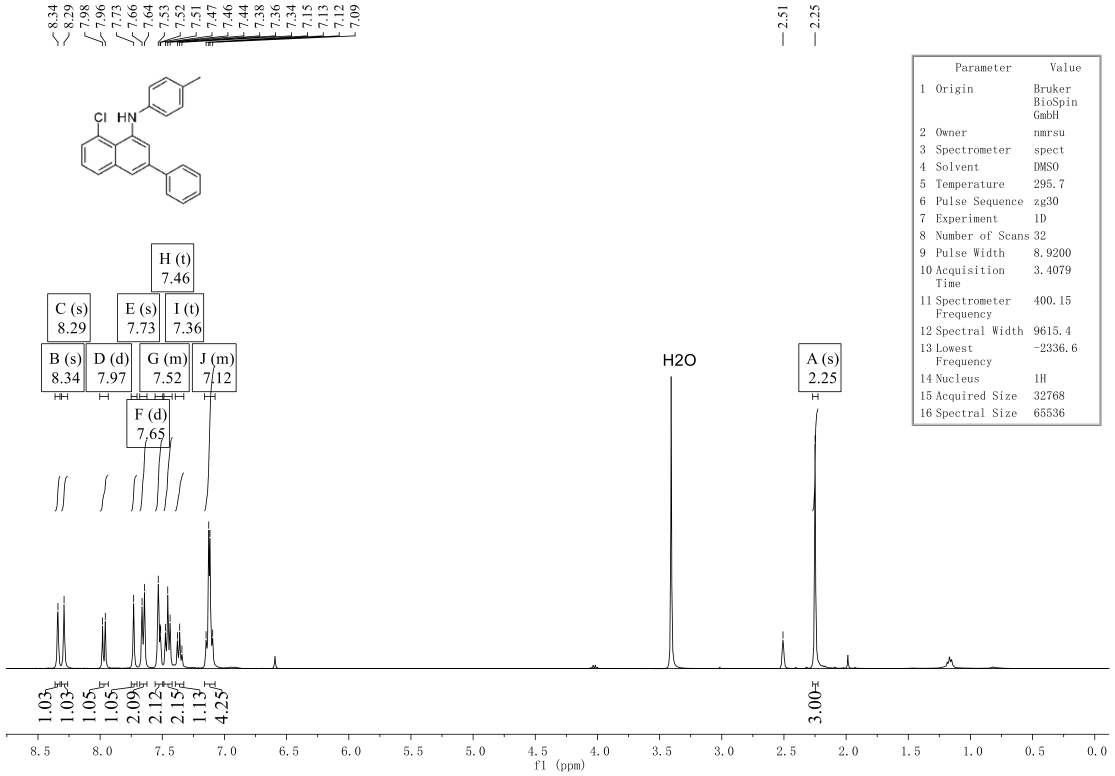
8.34 8.29 7.98 7.96 7.73 7.66 7.64 7.53 7.52 7.51 7.47 7.46 7.44 7.38 7.36 7.34 7.15 7.13 7.12 7.09
2.51
2.25
Cl HN
H (t) 7.46
C (s) 8.29
E (s) 7.73
I (t) 7.36
B (s) 8.34
D (d) 7.97
G (m) 7.52
J (m) 7.12
F (d) 7.65
H2O
A (s) 2.25
1.03 1.03 1.05 1.05 2.09 2.12 2.15 1.13 4.25
3.00
f1 (ppm)
Parameter Value
1 Origin Bruker BioSpin GmbH
2 Owner nmrsu
3 Spectrometer spect
4 Solvent DMSO
5 Temperature 295.7
6 Pulse Sequence zg30
7 Experiment 1D
8 Number of Scans 32
9 Pulse Width 8.9200
10 Acquisition Time 3.4079
11 Spectrometer Frequency 400.15
12 Spectral Width 9615.4
13 Lowest Frequency -2336.6
14 Nucleus 1H
15 Acquired Size 32768
16 Spectral Size 65536

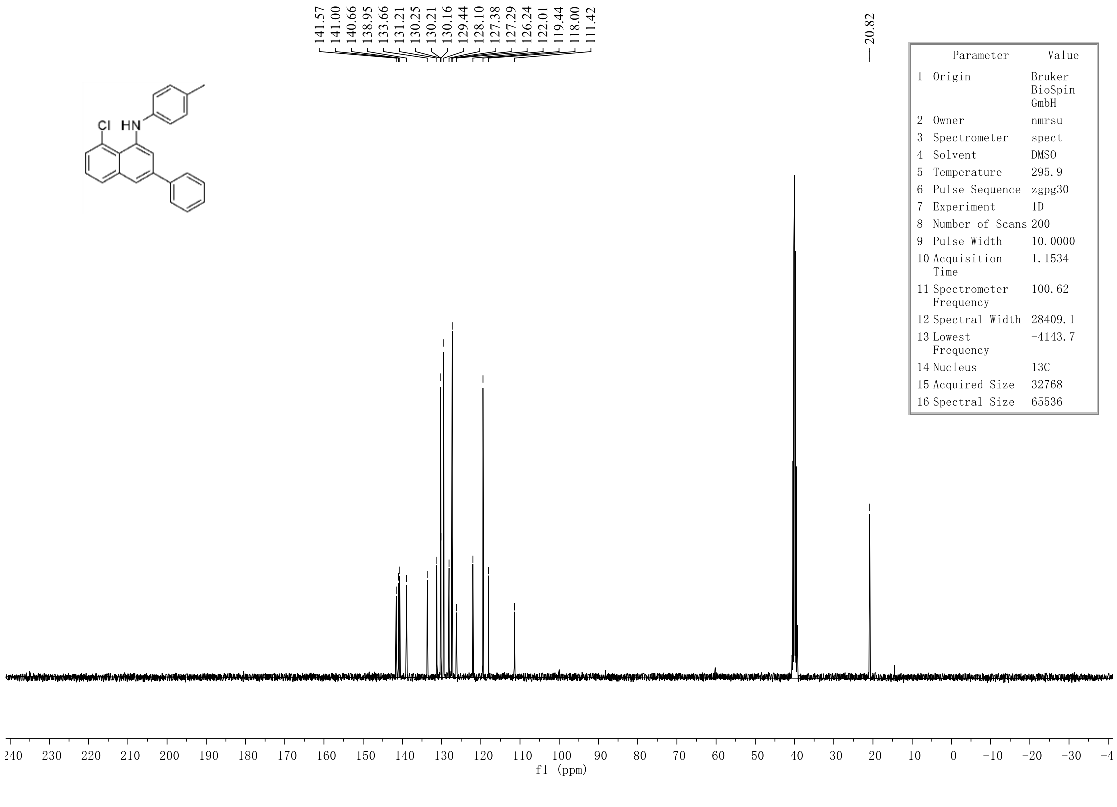
141.57 141.00 140.66 138.95 133.66 131.21 130.25 130.21 130.16 129.44 128.10 127.38 127.29 126.24 122.01 119.44 118.00 111.42
20.82
Cl HN
f1 (ppm)
Parameter Value
1 Origin Bruker BioSpin GmbH
2 Owner nmrsu
3 Spectrometer spect
4 Solvent DMSO
5 Temperature 295.9
6 Pulse Sequence zgpg30
7 Experiment 1D
8 Number of Scans 200
9 Pulse Width 10.0000
10 Acquisition Time 1.1534
11 Spectrometer Frequency 100.62
12 Spectral Width 28409.1
13 Lowest Frequency -4143.7
14 Nucleus 13C
15 Acquired Size 32768
16 Spectral Size 65536

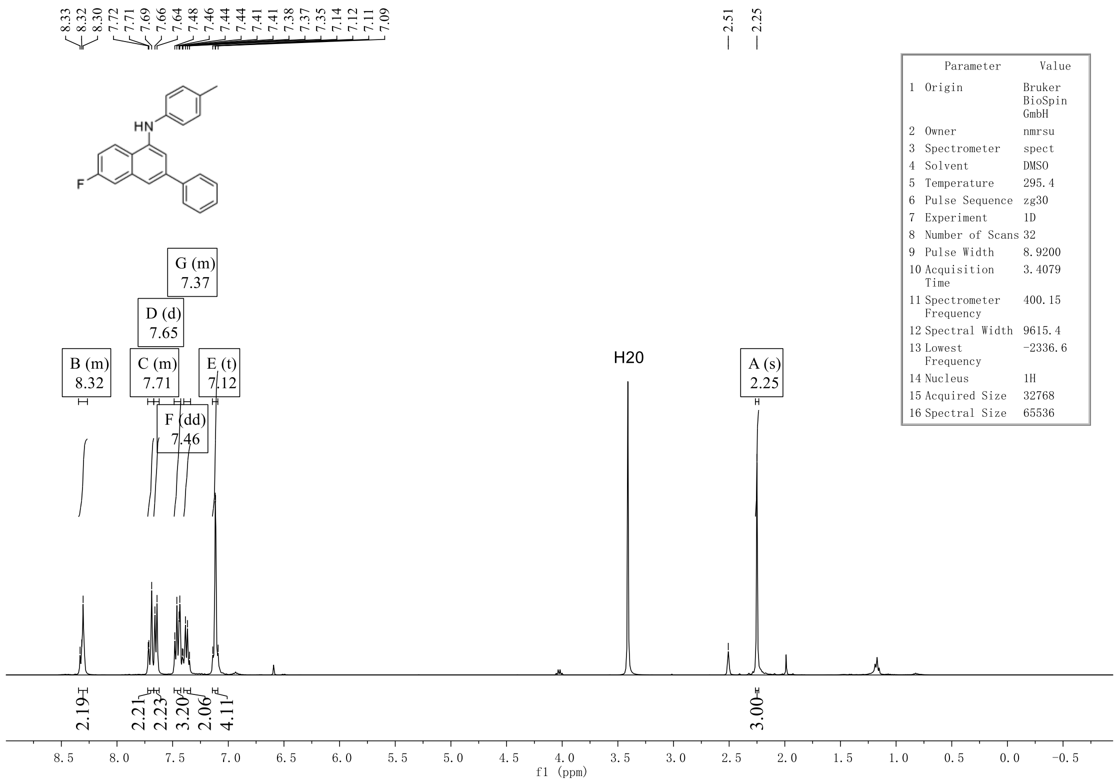
8.33
8.32
8.30
7.72
7.71
7.69
7.66
7.64
7.48
7.46
7.44
7.44
7.41
7.41
7.38
7.37
7.35
7.14
7.12
7.11
7.09
2.51
2.25
HN
F
G (m) 7.37
D (d) 7.65
B (m) 8.32
C (m) 7.71
E (t) 7.12
F (dd) 7.46
H20
A (s) 2.25
2.19
2.21
2.23
3.20
2.06
4.11
3.00
Parameter Value
1 Origin Bruker BioSpin GmbH
2 Owner nmrsu
3 Spectrometer spect
4 Solvent DMSO
5 Temperature 295.4
6 Pulse Sequence zg30
7 Experiment 1D
8 Number of Scans 32
9 Pulse Width 8.9200
10 Acquisition Time 3.4079
11 Spectrometer Frequency 400.15
12 Spectral Width 9615.4
13 Lowest Frequency -2336.6
14 Nucleus 1H
15 Acquired Size 32768
16 Spectral Size 65536
f1 (ppm)

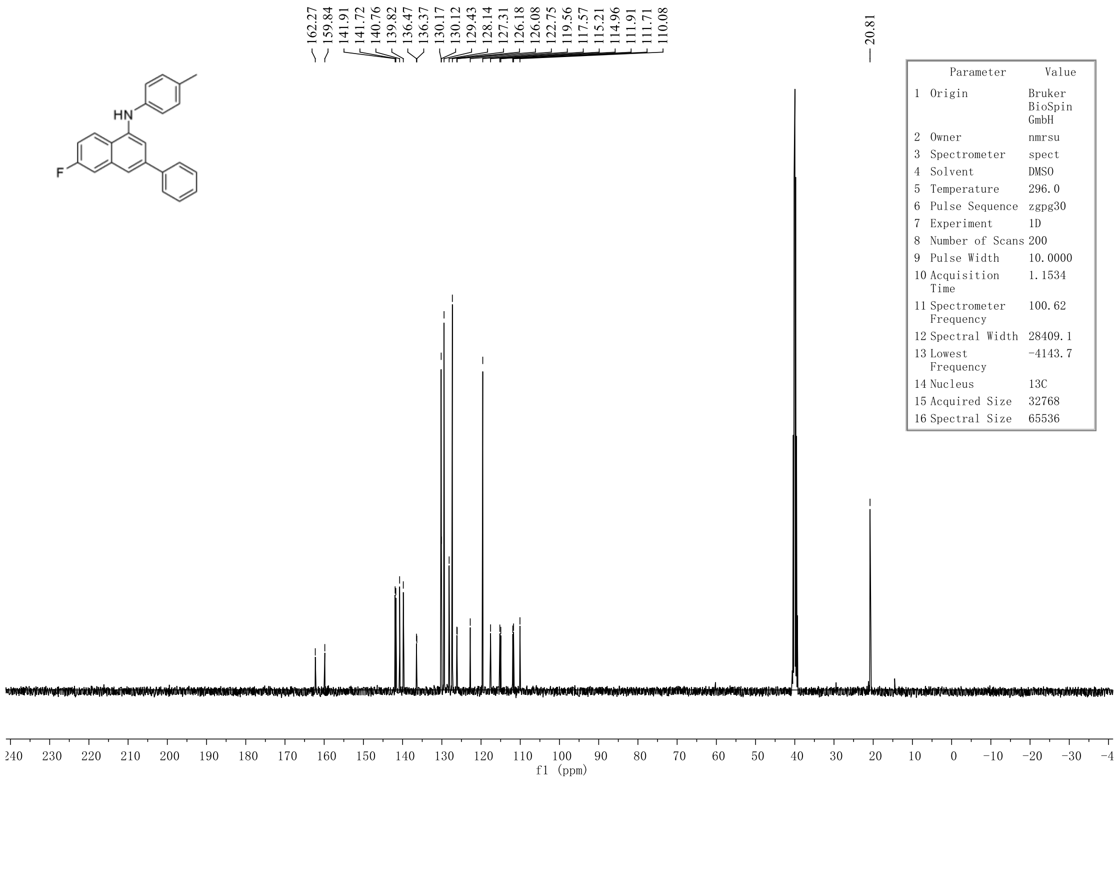
162.27
159.84
141.91
141.72
140.76
139.82
136.47
136.37
130.17
130.12
129.43
128.14
127.31
126.18
126.08
122.75
119.56
117.57
115.21
114.96
111.91
111.71
110.08
20.81
HN
F
Parameter Value
1 Origin Bruker BioSpin GmbH
2 Owner nmrsu
3 Spectrometer spect
4 Solvent DMSO
5 Temperature 296.0
6 Pulse Sequence zgpg30
7 Experiment 1D
8 Number of Scans 200
9 Pulse Width 10.0000
10 Acquisition Time 1.1534
11 Spectrometer Frequency 100.62
12 Spectral Width 28409.1
13 Lowest Frequency -4143.7
14 Nucleus 13C
15 Acquired Size 32768
16 Spectral Size 65536
f1 (ppm)

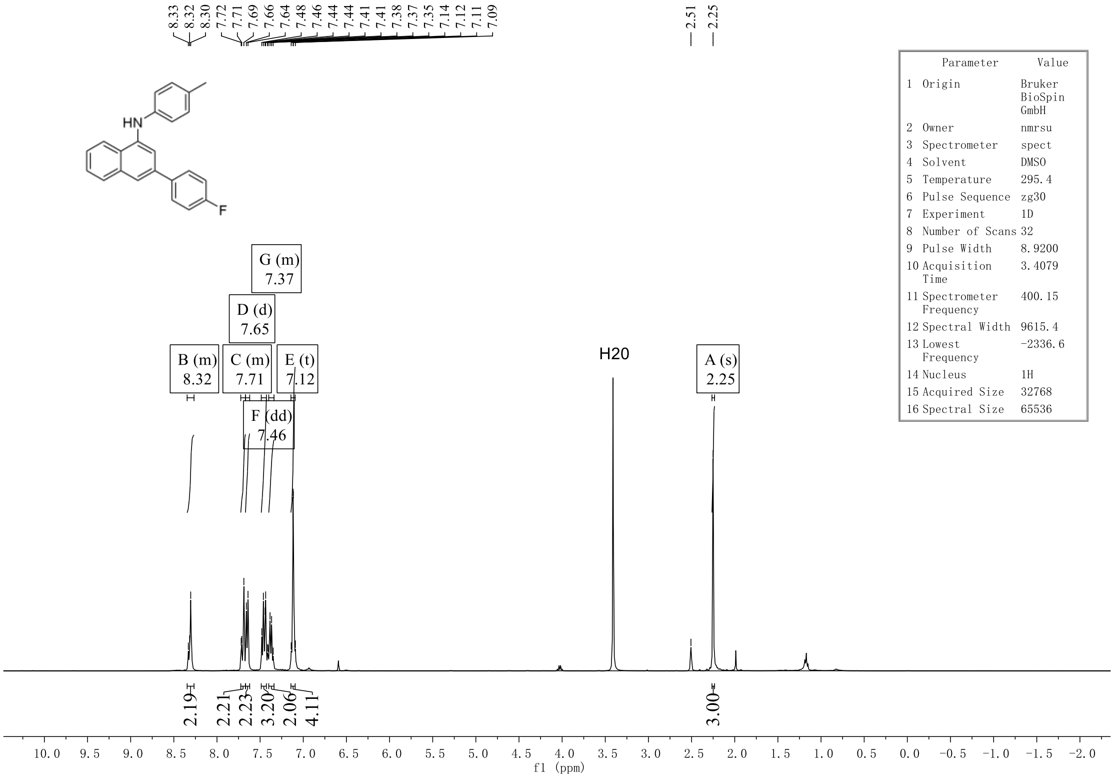
8.33
8.32
8.30
7.72
7.71
7.69
7.66
7.64
7.48
7.46
7.44
7.44
7.41
7.41
7.38
7.37
7.35
7.14
7.12
7.11
7.09
2.51
2.25
HN
F
G (m) 7.37
D (d) 7.65
B (m) 8.32
C (m) 7.71
E (t) 7.12
F (dd) 7.46
H20
A (s) 2.25
2.19
2.21
2.23
3.20
2.06
4.11
3.00
f1 (ppm)
Parameter Value
1 Origin Bruker BioSpin GmbH
2 Owner nmrsu
3 Spectrometer spect
4 Solvent DMSO
5 Temperature 295.4
6 Pulse Sequence zg30
7 Experiment 1D
8 Number of Scans 32
9 Pulse Width 8.9200
10 Acquisition Time 3.4079
11 Spectrometer Frequency 400.15
12 Spectral Width 9615.4
13 Lowest Frequency -2336.6
14 Nucleus 1H
15 Acquired Size 32768
16 Spectral Size 65536

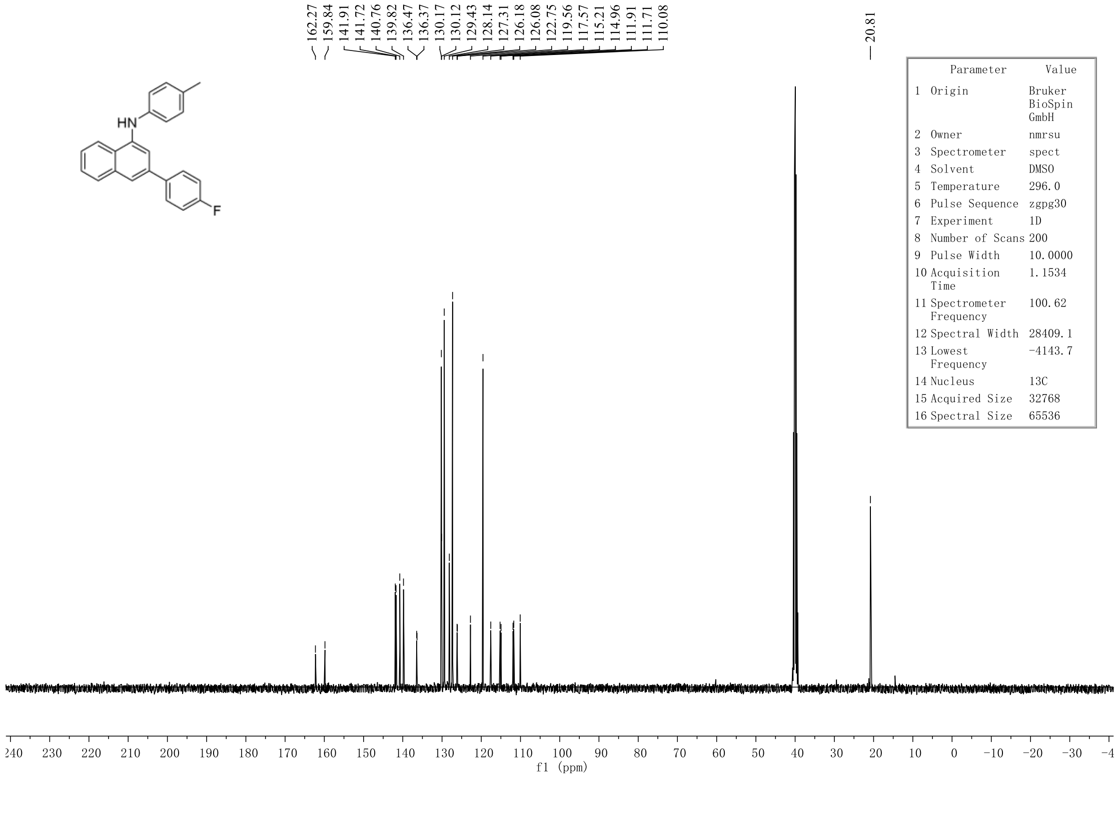
162.27
159.84
141.91
141.72
140.76
139.82
136.47
136.37
130.17
130.12
129.43
128.14
127.31
126.18
126.08
122.75
119.56
117.57
115.21
114.96
111.91
111.71
110.08
20.81
HN
F
f1 (ppm)
Parameter Value
1 Origin Bruker BioSpin GmbH
2 Owner nmrsu
3 Spectrometer spect
4 Solvent DMSO
5 Temperature 296.0
6 Pulse Sequence zgpg30
7 Experiment 1D
8 Number of Scans 200
9 Pulse Width 10.0000
10 Acquisition Time 1.1534
11 Spectrometer Frequency 100.62
12 Spectral Width 28409.1
13 Lowest Frequency -4143.7
14 Nucleus 13C
15 Acquired Size 32768
16 Spectral Size 65536

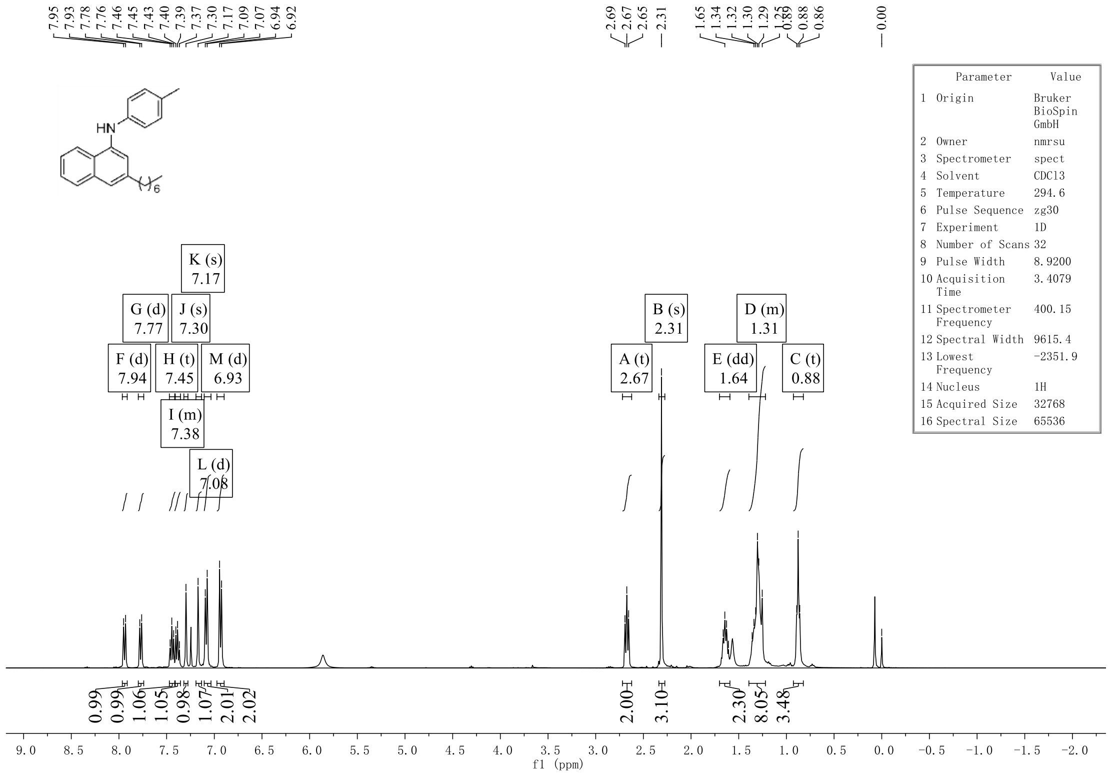
7.95
7.93
7.78
7.76
7.46
7.45
7.43
7.40
7.39
7.37
7.30
7.17
7.09
7.07
6.94
6.92
2.69
2.67
2.65
2.31
1.65
1.34
1.32
1.30
1.29
0.88
0.86
0.00
HN
6
K (s) 7.17
G (d) 7.77
J (s) 7.30
B (s) 2.31
D (m) 1.31
F (d) 7.94
H (t) 7.45
M (d) 6.93
A (t) 2.67
E (dd) 1.64
C (t) 0.88
I (m) 7.38
L (d) 7.08
0.99
0.99
1.06
1.05
0.98
1.07
2.01
2.02
2.00
3.10
2.30
8.05
3.48
f1 (ppm)
Parameter Value
1 Origin Bruker BioSpin GmbH
2 Owner nmrsu
3 Spectrometer spect
4 Solvent CDCl3
5 Temperature 294.6
6 Pulse Sequence zg30
7 Experiment 1D
8 Number of Scans 32
9 Pulse Width 8.9200
10 Acquisition Time 3.4079
11 Spectrometer Frequency 400.15
12 Spectral Width 9615.4
13 Lowest Frequency -2351.9
14 Nucleus 1H
15 Acquired Size 32768
16 Spectral Size 65536

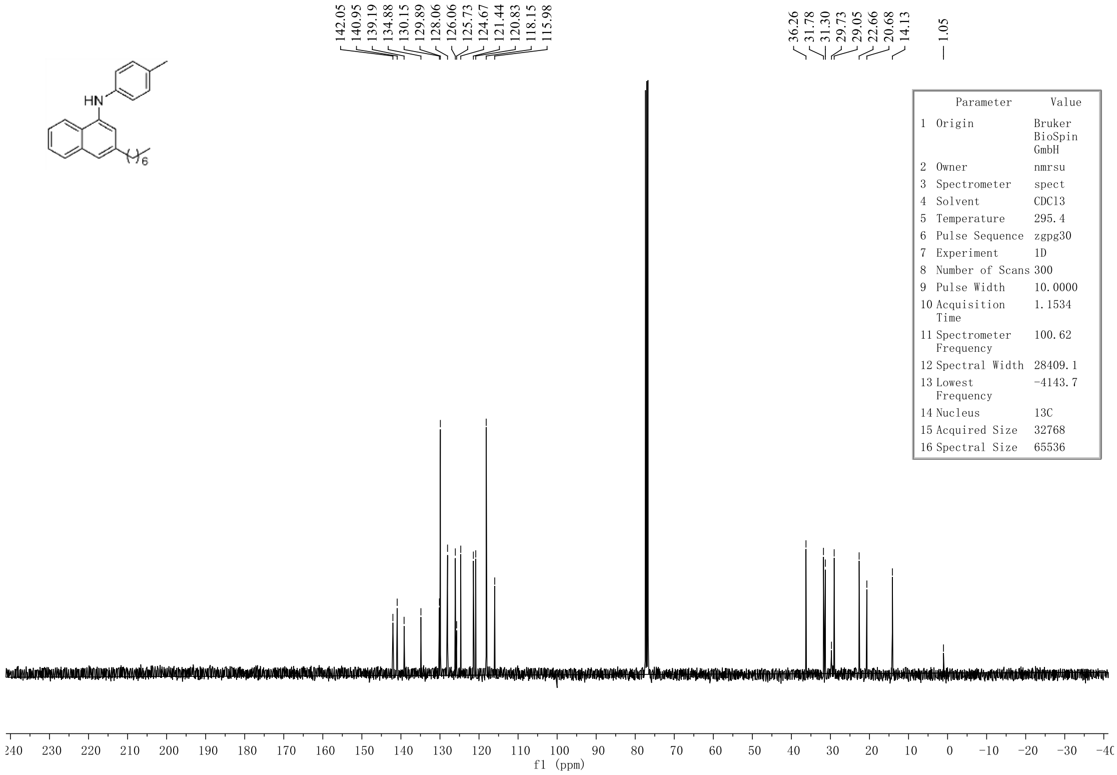
142.05
140.95
139.19
134.88
130.15
129.89
128.06
126.06
125.73
124.67
121.44
120.83
118.15
115.98
36.26
31.78
31.30
29.73
29.05
22.66
20.68
14.13
1.05
HN
6
f1 (ppm)
Parameter Value
1 Origin Bruker BioSpin GmbH
2 Owner nmrsu
3 Spectrometer spect
4 Solvent CDCl3
5 Temperature 295.4
6 Pulse Sequence zgpg30
7 Experiment 1D
8 Number of Scans 300
9 Pulse Width 10.0000
10 Acquisition Time 1.1534
11 Spectrometer Frequency 100.62
12 Spectral Width 28409.1
13 Lowest Frequency -4143.7
14 Nucleus 13C
15 Acquired Size 32768
16 Spectral Size 65536

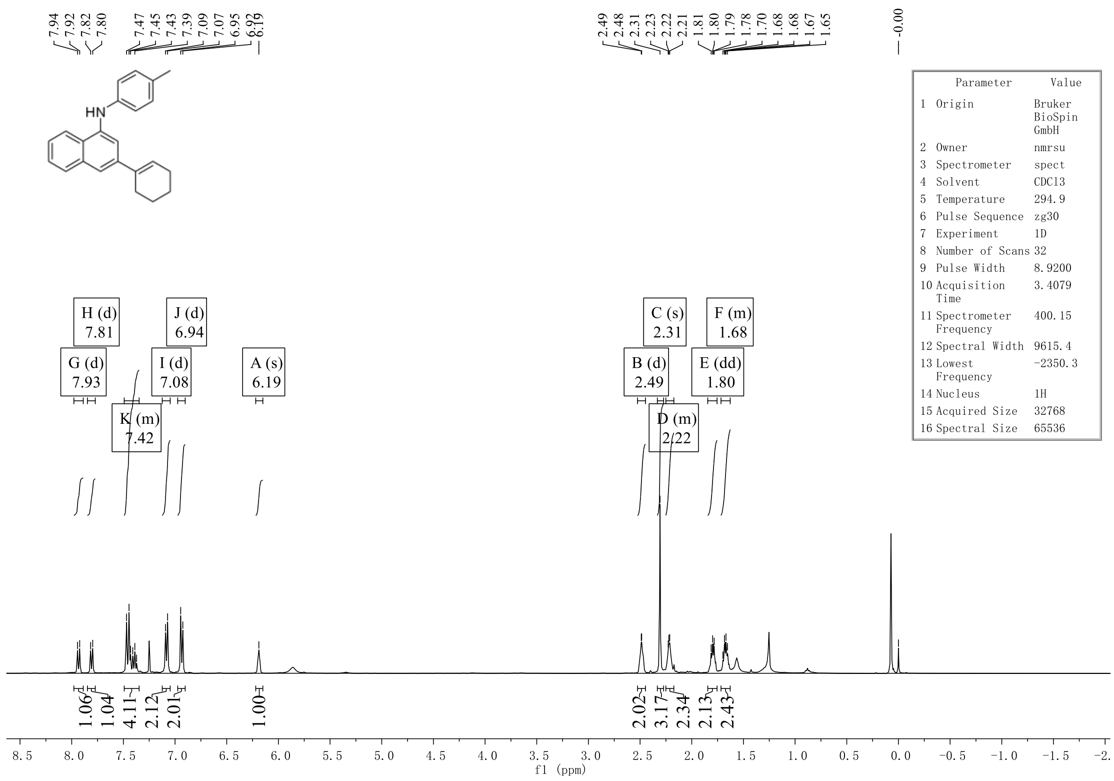

	Parameter	Value
1	Origin	Bruker BioSpin GmbH
2	Owner	nmrsu
3	Spectrometer	spect
4	Solvent	CDCl3
5	Temperature	294.9
6	Pulse Sequence	zg30
7	Experiment	1D
8	Number of Scans	32
9	Pulse Width	8.9200
10	Acquisition Time	3.4079
11	Spectrometer Frequency	400.15
12	Spectral Width	9615.4
13	Lowest Frequency	-2350.3
14	Nucleus	1H
15	Acquired Size	32768
16	Spectral Size	65536

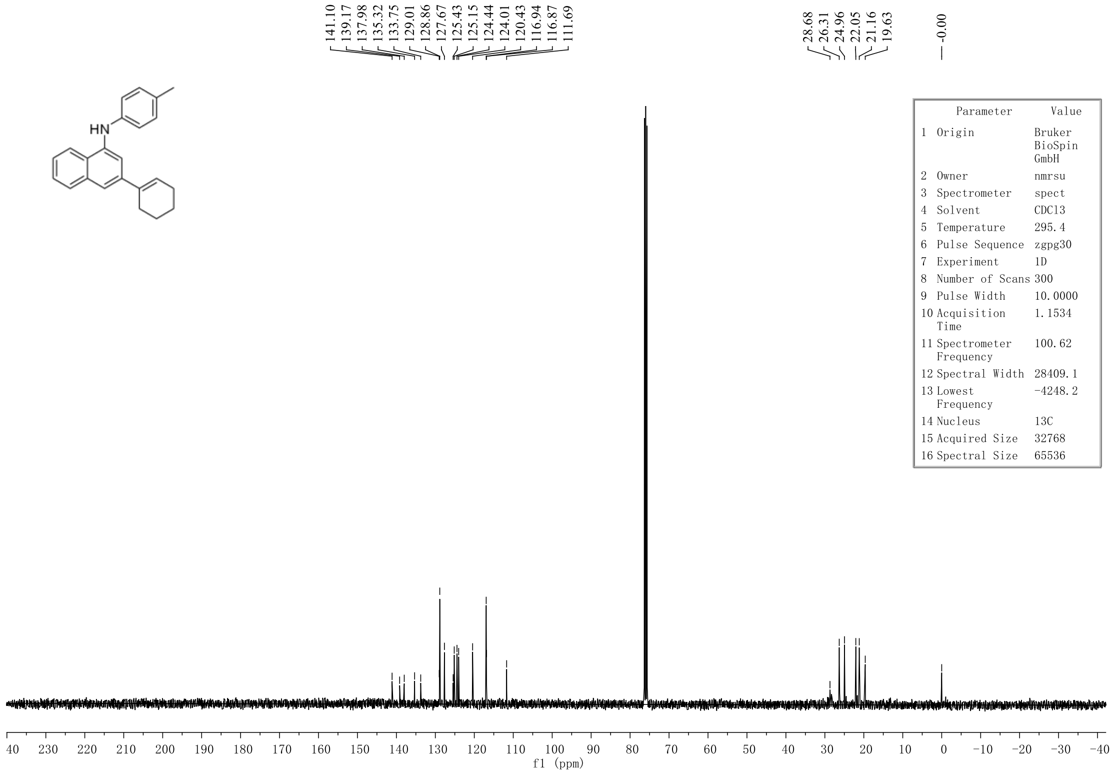

	Parameter	Value
1	Origin	Bruker BioSpin GmbH
2	Owner	nmrsu
3	Spectrometer	spect
4	Solvent	CDCl3
5	Temperature	295.4
6	Pulse Sequence	zgpg30
7	Experiment	1D
8	Number of Scans	300
9	Pulse Width	10.0000
10	Acquisition Time	1.1534
11	Spectrometer Frequency	100.62
12	Spectral Width	28409.1
13	Lowest Frequency	-4248.2
14	Nucleus	13C
15	Acquired Size	32768
16	Spectral Size	65536

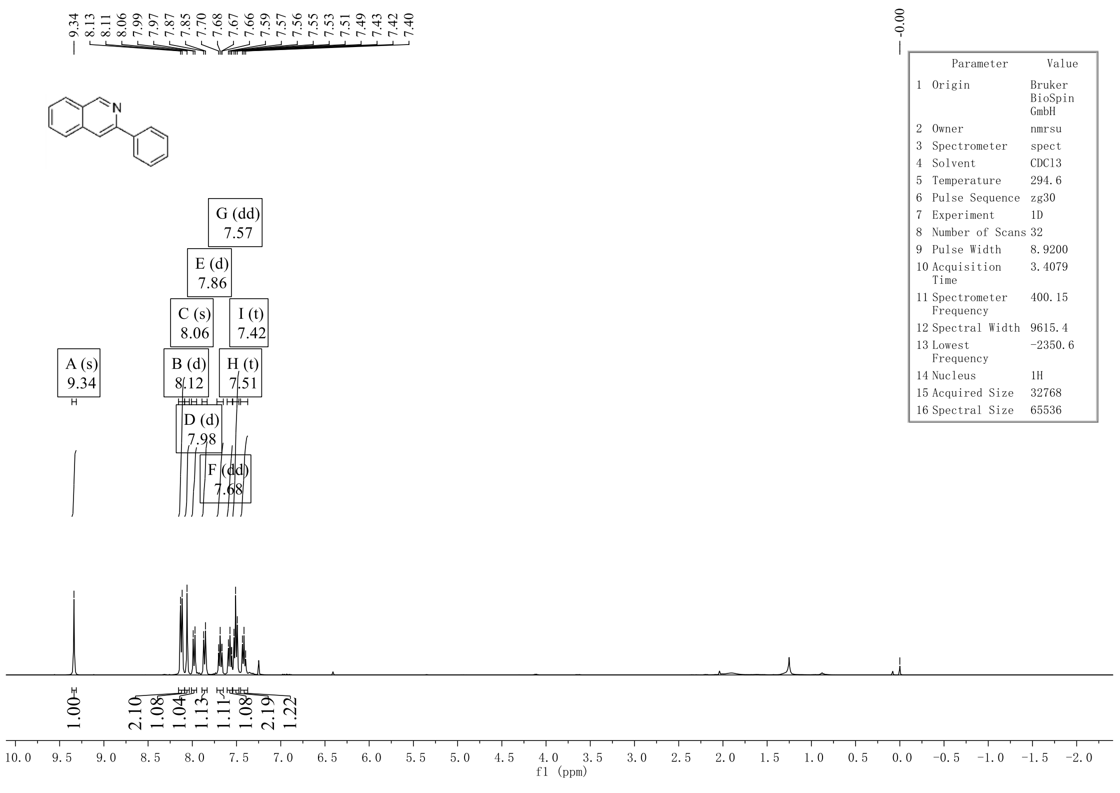
9.34 8.13 8.11 8.06 7.99 7.97 7.87 7.85 7.70 7.68 7.67 7.66 7.59 7.57 7.56 7.55 7.53 7.51 7.49 7.43 7.42 7.40
-0.00
N
A (s) 9.34
B (d) 8.12
C (s) 8.06
D (d) 7.98
E (d) 7.86
F (dd) 7.68
G (dd) 7.57
H (t) 7.51
I (t) 7.42
1.00 2.10 1.08 1.04 1.13 1.11 1.08 2.19 1.22
Parameter Value
1 Origin Bruker BioSpin GmbH
2 Owner nmrsu
3 Spectrometer spect
4 Solvent CDCl3
5 Temperature 294.6
6 Pulse Sequence zg30
7 Experiment 1D
8 Number of Scans 32
9 Pulse Width 8.9200
10 Acquisition Time 3.4079
11 Spectrometer Frequency 400.15
12 Spectral Width 9615.4
13 Lowest Frequency -2350.6
14 Nucleus 1H
15 Acquired Size 32768
16 Spectral Size 65536
10.0 9.5 9.0 8.5 8.0 7.5 7.0 6.5 6.0 5.5 5.0 4.5 4.0 3.5 3.0 2.5 2.0 1.5 1.0 0.5 0.0 -0.5 -1.0 -1.5 -2.0
f1 (ppm)
